# Isoform-specific oxidative modifications of tropoelastin by HOCl and MPO alter protein self-assembly

**DOI:** 10.1080/13510002.2025.2592409

**Published:** 2025-11-28

**Authors:** Karoline Lindgaard Mikkelsen, Tina Nybo, Michael J. Davies, Adelina Rogowska-Wrzesinska

**Affiliations:** a Department of Biochemistry and Molecular Biology and VILLUM Center for Bioanalytical Sciences, University of Southern Denmark, Odense M, Denmark; b Department of Biomedical Sciences, University of Copenhagen, Copenhagen N, Denmark

**Keywords:** Tropoelastin, hypochlorous acid, myeloperoxidase, post-translational modification, oxidation, extracellular matrix, coacervation, cardiovascular disease

## Abstract

**Background:**

Tropoelastin (TE), the soluble precursor of elastin, is critical for the elasticity of arteries, lungs, and skin. Oxidative damage to TE has been implicated in vascular diseases, but the isoform-specific effects remain poorly understood. Hypochlorous acid (HOCl), generated by the enzyme myeloperoxidase (MPO) targets extracellular matrix proteins during inflammatory processes. However, the differential susceptibility and functional consequences in specific TE isoforms are unknown.

**Methods:**

We investigated the effects of HOCl and MPO-derived oxidants on two human TE isoforms, TE2 and TE6. Oxidative modifications were analyzed using high-resolution LC-MS/MS, with site-specific identification of chlorinated tyrosines and oxidized cysteine residues. Functional consequences were assessed using turbidity-based coacervation assays.

**Results:**

TE2 exhibited chlorination at multiple tyrosine residues, particularly 3,5-dichlorotyrosine, while showing minimal cysteine oxidation. In contrast, TE6 was more oxidised at its single disulfide bond, resulting in irreversible sulfonic acid formation. These isoform-specific patterns translated into functional differences: TE2 demonstrated enhanced coacervation , whereas TE6 showed reduced assembly capacity, consistent with structural destabilization.

**Conclusion:**

HOCl and MPO-derived oxidants induce distinct modifications in tropoelastin isoforms, resulting in divergent effects on protein self-assembly. These findings highlight the importance of isoform context in extracellular matrix remodeling under oxidative stress and may have implications for vascular pathologies.

## Introduction

Elastin is the main component of elastic fibers (often >90%) and is a major contributor to the structural stability and flexibility (stretch and recoil) of elastic tissues such as arteries, lungs, and skin. It is highly abundant and constitutes up to 30% of the total dry mass of vascular vessels [1, 2]. Mature elastin is formed by the assembly and cross-linking of its soluble precursor protein tropoelastin (TE). TE is synthesized by elastogenic cells, such as fibroblasts, endothelial cells, and smooth muscle cells (SMCs), and is predominantly expressed during late pre-natal to early postnatal development. The protein exists in multiple isoforms that are tissue and cell-specific, arising from alternative gene splicing. Thus, different TE isoforms appear to have different and specific biological roles [1, 2] though the biological roles of different isoforms is poorly researched and understood [3]. Expression of TE is largely absent in mature tissue under baseline conditions [1, 2], resulting in mature, insoluble elastin being the major form present in healthy tissues [4-6]. However, expression of TE can be induced upon injury to elastic fibers as part of tissue repair and regeneration. Upregulation of TE expression and the presence of soluble TE peptides have been observed in human atherosclerotic plaques [7-10] and in photo-damaged skin, where different isoforms appear to be laid down during tissue healing [11]. Furthermore, it has been reported that multiple isoforms co-exist in mature tissues [12-14]. Elastin-derived peptides have also been detected in plasma from humans with various pathologies consistent with turnover of mature elastin or TE [8, 10, 15]. Although elastogenesis, i.e. the expression, coacervation (TE self-assembly into spherical particles before becoming cross-linked into elastic fibers), cross-linking of TE and its subsequent incorporation into microfibrils, is activated in various diseases, including within human atherosclerotic plaques, the newly formed/repaired elastic fibers often display altered morphology [7-10]. This suggests dysregulation at one or more steps in the complex, multistep process of elastogenesis. Furthermore, there is extensive evidence for a decreased content of elastin and elevated levels of degradation products in abdominal aortic aneurysms, with this loss associated with chronic inflammation, aortic rupture, and high mortality levels [16-18].

Increased oxidative stress is an early and common event in pathologies associated with acute or chronic inflammation, including many cardiovascular pathologies. In both atherosclerosis and abdominal aneurysms, oxidative stress is reported to persist throughout the progression of the disease, making it a key and possibly causal component [19-21]. However, its role in the pathophysiology of these diseases is still not fully elucidated [21] and remains an active area of research [19, 22, 23]. An increasing body of evidence shows that proteins, glycoproteins, and proteoglycans of the extracellular matrix (ECM) can be modified by oxidants generated as a result of the activation of neutrophils, monocytes, and macrophages at sites of inflammation, including within the artery wall [24-26]. In particular, strong evidence has been presented for a critical role for the heme enzyme myeloperoxidase (MPO), which is released by the above cells and generates the reactive chlorinating and oxidizing species, hypochlorous acid (HOCl) (reviewed [27, 28]). This enzyme, and also peroxynitrite/peroxynitrous acid/peroxynitrosocarbonate ($\mathrm{ONO}O^{-}{/\mathrm{ONOOH}/\mathrm{ONOOCO}}_{2}^{-}$, or species derived from these) are potent sources of nitrating species, and subsequent products within atherosclerotic plaques [29, 30]. $\mathrm{ONO}O^{-}/\mathrm{ONOOH}$ are formed by the diffusion-controlled reaction of superoxide anion radicals ($O_{2}^{\cdot -}$; formed by multiple biological processes) with nitric oxide (NO^.^; from either constitutive, eNOS, or induced, iNOS, nitric oxide synthase enzymes), with ONOO^−^ undergoing rapid subsequent addition to CO_2_ to give ${\mathrm{ONOOCO}}_{2}^{-}$ [31]. In several cases, MPO levels and the damage that these systems generate are both diagnostic of disease and can predict adverse outcomes (e.g. [32, 33]).

The hypothesis that oxidation, chlorination, and nitration play important roles in disease development is supported by data showing that ECM proteins form aberrant structures and have perturbed functions after oxidant exposure. This includes ECM glycoproteins such as laminin [34] and fibronectin [35, 36] as well as the heparan sulfate proteoglycan, perlecan [37, 38]. Elastin, and its soluble precursor TE, are also targets, both *in vitro* and *in vivo* [22, 39, 40]. Previous studies have shown that exposure of TE to equimolar or greater concentrations of ONOOH *in vitro* results in the generation of high yields of 3-nitrotyrosine (3-nitroTyr), a biomarker of nitrating species [22]. Exposure to high molar excesses of ONOOH [39, 40] results in impaired assembly of TE into elastic fibers, suggesting that oxidative modification of TE has functional consequences. Whether this is also true for HOCl and the biologically relevant $\mathrm{MPO}\text{–}H_{2}\text{–}O_{2}\text{–}Cl^{-}$ enzyme system is unclear.

In this study, we propose that oxidative modifications induced by hypochlorous acid (HOCl) and the $\mathrm{MPO}\text{–}H_{2}\text{–}O_{2}\text{–}Cl^{-}$ enzymatic system occur in a site-specific and isoform-dependent manner, ultimately altering tropoelastin's ability to self-assemble. We demonstrate that TE6 is highly susceptible to cysteine oxidation and disulfide bond cleavage, which compromises its assembly into elastic fibers. In contrast, TE2 undergoes preferential tyrosine chlorination, enhancing its aggregation. These distinct oxidative signatures highlight isoform-specific vulnerabilities and suggest divergent roles for TE2 and TE6 in preserving extracellular matrix structure under inflammatory conditions.

## Materials and methods

### Materials

Myeloperoxidase (MPO) from human leukocytes (Calbiochem, Catalogue No. 475 911) was used for the $\mathrm{MPO}\text{–}H_{2}\text{–}O_{2}\text{–}Cl^{-}$ system. Sequencing – grade modified trypsin (Promega, Catalogue No. V5111 for lyophilized or V5113 for frozen) and endoproteinase Lys – C (FUJIFILM Wako, Catalogue No. 125-05061) were used for proteolytic digestion. Unless otherwise specified, all chemicals and reagents used in this study were of high-purity LC-MS grade and obtained from Sigma-Aldrich (St. Louis, MO, USA).

### Tropoelastin isoforms and oxidative treatment

Two recombinant human tropoelastin (TE) isoforms were used: TE2, produced and kindly provided by Prof. Anthony Weiss (Univ. of Sydney, Sydney, Australia) [41], and TE6, commercially obtained (CellAdhere™, STEMCELL Technologies, Cat# 07003). These were made, handled, and stored in an identical manner. SDS-PAGE confirmed the purity of the preparations. Oxidative treatments were performed using either 500 µM hypochlorous acid (HOCl) or an enzymatic myeloperoxidase $(\mathrm{MPO})\text{–}H_{2}O_{2}\text{–}Cl^{-}$ system mimicking inflammatory conditions [34, 42].

Oxidative treatments and corresponding controls were designed to distinguish direct HOCl effects from enzymatic myeloperoxidase (MPO)-mediated oxidation. TE isoforms (1 mg mL^−1^ in 100 mM sodium phosphate, pH 7.4) were treated with either 0 µM (control) or 500 µM HOCl for 1 h at 21°C, corresponding to approximately a 30-fold molar excess of oxidant. For enzymatic oxidation, TE was incubated with 0.1 µM MPO, 500 µM H_2_O_2_, and 100 mM NaCl at 37°C. H_2_O_2_ was added in 50 µM portions every 10 min to maintain continuous HOCl formation while preventing MPO inactivation. Control samples were included for each missing reaction component – no MPO, no H_2_O_2_, and control TE (untreated) – to verify that observed modifications originated specifically from HOCl or MPO activity rather than incubation or buffer conditions. HOCl concentrations in stock solutions were quantified spectrophotometrically at 292 nm using a molar extinction coefficient $\left(\epsilon_{\left(292\right)}=350\;M^{-1}\cdot cm^{-1}\right)$ [35, 43]. MPO-derived HOCl was quantified by monitoring the decrease in absorbance at 290 nm of the HOCl-sensitive probe monochlorodimedone (MCD; 100 mM stock in 96% ethanol), using (*ϵ*_290_) of 17,700 M^−1^ cm^−1^ [43].

### Mass spectrometry and quantification of oxidative modifications

After oxidation, TE samples were digested TE (100 µg) in-solution without reduction and alkylation as described previously [44]. Briefly, after oxidant treatment, the protein samples were cleaned up by spin-filter-based buffer exchange using 10 kDa spin-filters (Amicon Ultra-0.5 Ultracel-10 K, Merck Millipore, Ireland) to remove salts and excess reactants. The protein samples were then dissolved in denaturation buffer (4 M urea, 1% sodium deoxycholate (SDC), 50 mM triethylammonium bicarbonate (TEAB), pH 8.5), and incubated for at least 3 h at 30°C. Denaturation was followed by a two-step digestion protocol using Lys-C (1:50 w/w) for 2 h in 4 M urea, and trypsin (1:50 w/w) in 1 M urea for 18 h. The temperature was kept at 30°C to minimize protein carbamylation. SDC was subsequently removed using acidification and ethyl acetate phase transfer. One µg of the peptide mixture was separated on a two-column system using an EASY-nanoLC 1000 instrument (Thermo Fisher Scientific). The system was equipped with two in-house prepared fused silica columns; a pre-column (5 µm particle size, C18 beads, 3 cm × 100 µm ID) coupled to a laser-pulled analytical column (3 µm particle size, C18 beads, 18 cm × 75 µm ID). The peptides were separated at a flow rate of 250 nL min^−1^ over a 60 min gradient using solution A (0.1% formic acid in H_2_O) with 3 to 45% solution B (90% acetonitrile, 0.1% formic acid in H_2_O). Reverse phase-separated peptides were subsequently analyzed on a QExactive HF Quadrupole-Orbitrap (Thermo Fisher Scientific) mass spectrometer in positive ion mode using data-dependent acquisition. Full scans were performed across a range of 350–1600 m/z and recorded at a resolution of 120,000. The MS/MS scan mode selected the 20 most intense ions (charge states of +2 to +4, with dynamic exclusion for 30 s) selected for higher energy collisional dissociation (HCD) fragmentation at a normalized collision energy of 28. Blanks were run between each sample to monitor and prevent carry-over.

Raw MS data were acquired and processed using Xcalibur (Thermo Fisher Scientific). Data files were analyzed using Progenesis QI (Nonlinear Dynamics, USA) for chromatographic alignment and grouped according to their conditions and precursor peak quantification. Data searches were performed using Proteome Discoverer 2.2 for TE2 and 2.3 for TE6, respectively. The data were searched against an isoform database of human TE (downloaded 02/03/2018). For MPO-mediated oxidation, MPO isoform sequences were also included in the database. Following search parameters were used: parent ion tolerance – 4 ppm; fragment ion tolerance – 0.1 Da; trypsin – 2 missed cleavages allowed; fixed modifications – none; variable modifications: chlorination (at Tyr, Y; and tryptophan, W), di-chlorination (Y, W), mono-oxidation (at methionine, M; histidine, H; cysteine, C; and W), di-oxidation (at M, C, W), and tri-oxidation (C). All other parameters were left at their default settings. The Proteome Discoverer data were exported as pepXML files and imported back into Progenesis QI for analysis. Contaminations were removed according to the TE accession number P15502. The peptide abundances and relative occupancy of modification sites were measured by integrating the precursor peak area. Relative site occupancy (RSO) was calculated based on extracted ion chromatograms of modified versus unmodified peptide forms, using Progenesis QI. Chlorinated peptides were verified by manual inspection of peptide fragment ions and isotopic pattern analysis, and the annotated spectra are presented in Figure S2. This quantification approach follows previously established methods [44, 45].

### Statistical analysis

All data are presented as mean ± standard error of the mean from at least three independent biological replicates (*n* = 3). Where applicable, measurements were conducted in technical triplicate to ensure reproducibility. Mass spectrometry data were normalized using median centering, and peptide intensities were log-transformed before statistical analysis. Normality was assessed using the Shapiro–Wilk test, and all statistical analyzes were performed in R version 3.4.4, with significance thresholds set at *p* < 0.05, unless otherwise indicated.

Changes in modified and unmodified peptide abundance were evaluated using a two-sided Student's t-test in R version 3.4.4. For the $(\mathrm{MPO})\text{–}H_{2}O_{2}\text{–}Cl^{-}$ system, a modification was considered significant if its corresponding peptide had a *p*-value < 0.065 between Control TE and MPO-treated samples and a *p*-value < 0.05 in at least one other control condition. This threshold was applied due to the high similarity between control conditions, ensuring robust identification of MPO-specific effects.

Principal Component Analysis (PCA) was used to assess global differences in modification patterns between oxidized and control samples. Variance in principal components was analyzed, and clustering significance was evaluated using permutation tests or ANOVA on principal components, where applicable.

For coacervation kinetics and self-assembly analysis, differences between conditions were evaluated using one-way ANOVA with Tukey's post hoc test to compare treatment groups. Time-dependent changes were analyzed using repeated-measures ANOVA, where applicable.

### Coacervation assay

Coacervation was examined by monitoring the turbidity of the reaction systems by light scattering using a FLUOstar Omega microplate reader (BMG LABTECH). The assay was performed in a 96-well plate with a final protein concentration of 0.25 µg µL^−1^ diluted in sample buffer (0.1 M sodium phosphate, pH 7.4). Three treatment conditions (TE control and 30-fold or 120-fold times molar excesses of HOCl) were employed, and all experiments were carried out in triplicate. 150 mM NaCl was added directly after the oxidant treatment and before transfer to the microplate reader, with the reaction plates kept at 37°C with gentle shaking for the duration of the measurement. The light scattering was monitored by measuring the absorbance at 550 nm over a period of 90 min, with readings taken every 2 min.

## Results

### Characterization of tropoelastin isoforms

**Figure 1.**
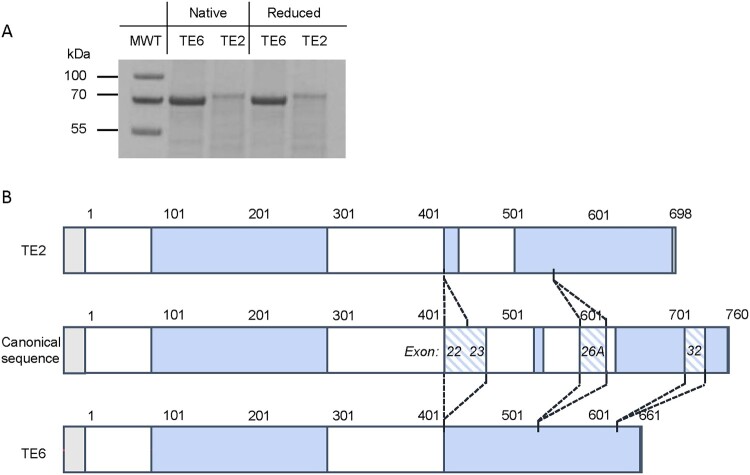
SDS-PAGE and sequence coverage of tropoelastin Isoforms TE2 and TE6. (A) SDS–PAGE analysis showing distinct migration patterns of TE2 and TE6, consistent with differences in exon composition and theoretical molecular masses (62.6 kDa for TE2 and 59.5 kDa for TE6). (B) LC–MS/MS sequence coverage maps. Blue regions indicate peptides detected by MS, white regions correspond to undetected segments, and diagonal stripes mark isoform-unique exons relative to the canonical sequence.

To validate the identity and purity of the TE isoforms, we performed SDS-PAGE and LC-MS/MS analysis. SDS-PAGE revealed single dominant bands in the 60–70 kDa range for both isoforms, with TE2 migrating slightly higher than TE6, consistent with their theoretical molecular masses (62.6 kDa for TE2 and 59.5 kDa for TE6) and exon composition differences, as shown in Figures 1A and 1B.

Peptide mapping by LC-MS/MS confirmed sequence identity and exon-specific differences. TE6 (UniProt: P15502-6) showed 64% coverage of its theoretical sequence and 39% of the canonical reference gene (ELN: HGNC:3327), while TE2 (UniProt: P15502-2) achieved 58% and 45% coverage, respectively. Isoform-specific peptides were identified based on the presence or absence of exons 22, 23, and 32 (e.g. Tyr426-Lys437 for TE2, Tyr426-Lys484 for TE6). Both isoforms lacked exon 26A, as confirmed by sequence coverage in domains 26 and 27.

Regions 1–80 and 300–420 showed poor coverage due to variable lysine and proline content: lysine-rich regions produced undetectably small peptides, while lysine-deficient ones yielded peptides beyond the MS scan range. All peptides within the detectable MS range (m/z 350–1600) were successfully identified and quantified, as detailed in Table S1, ensuring reliable detection of isoform-specific oxidative modifications.

### Identification of oxidative modifications

TE2 and TE6 were treated with HOCl or the $\mathrm{MPO}\text{–}H_{2}\text{–}O_{2}\text{–}Cl^{-}$ enzymatic system to induce oxidative modifications. LC-MS/MS analysis identified a range of site-specific modifications across both isoforms, including 3-chlorotyrosine (3-ClTyr), 3,5-dichlorotyrosine (3,5-Cl_2_Tyr), and cystine oxidation products such as sulfenic (Cys-SOH), sulfinic (Cys-SO_2_H), and sulfonic (Cys-SO_3_H) acids as detailed in Tables S2, S5–S8. All theoretically modifiable cystine residues were detected in both modified and unmodified forms, confirming 100% detection and oxidation. Of the total tyrosine residues, 67% were detected, and all showed evidence of oxidative modification under at least one condition, as shown in Table S1, S2 and S4. These data demonstrate the high analytical sensitivity and specificity of our LC-MS/MS workflow for capturing stable and MS-compatible oxidative proteoforms.

A total of 12 modified sites were identified in TE2 after HOCl treatment, including 10 chlorinated Tyr and 2 oxidized Cys residues. The $\mathrm{MPO}\text{–}H_{2}\text{–}O_{2}\text{–}Cl^{-}$ system produced a nearly identical profile in TE2, with the addition of a uniquely modified Tyr202 detected only after MPO treatment. In TE6, 11 modified residues were detected following HOCl treatment, and 9 involved both tyrosine and cysteine residues with MPO. Nine peptides carried multiple modifications, and some exhibited ambiguous site localization due to poor fragmentation quality; these are indicated in Table S2 and were interpreted with caution. It should be noted that these oxidation systems can also generate other modifications which were not assessed in our analyzes, and this is a weakness of this study. However, it should be noted that TE lacks many of the other residues (e.g. Cys, Trp, Met) which are known (highly reactive) targets of HOCl [42, 46].

Distinct isoform- and oxidant-specific modification patterns were observed. For example, Tyr202 was selectively modified by MPO in TE2, whereas Tyr426, Tyr632, and Cys-656 were exclusively modified by HOCl in TE6, Table S2. These differences likely reflect isoform-specific structural features that modulate the accessibility or reactivity of individual residues.

**Figure 2.**
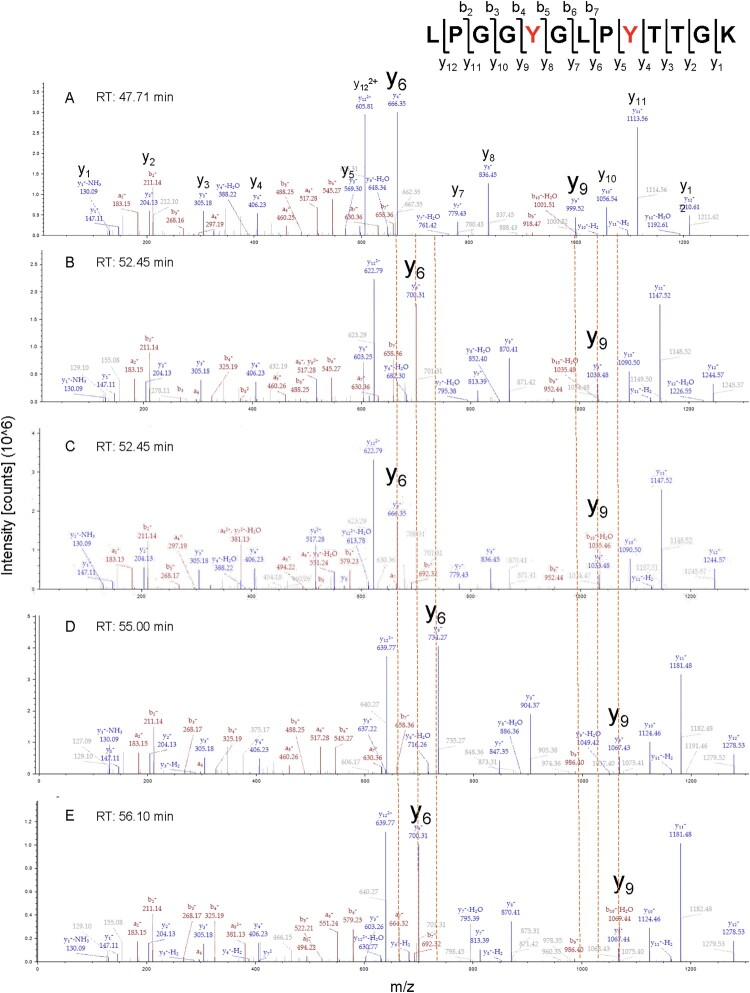
MS/MS spectra of peptide LPGGYGLPYTTGK showing site-specific HOCl-induced chlorination patterns. Higher-energy collisional dissociation (HCD) fragmentation was used to localize site-specific chlorination in tropoelastin isoforms treated with HOCl or the $\mathrm{MPO}\text{–}H_{2}\text{–}O_{2}\text{–}Cl^{-}$ system. The peptide LPGGYGLPYTTGK contains two tyrosine residues (Tyr191 and Tyr195) susceptible to chlorination, generating multiple proteoforms. Panels A–E show annotated y-ion series for: (A) unmodified peptide, (B) monochlorination at Tyr195, (C) monochlorination at Tyr191, (D) dichlorination at Tyr195, and (E) monochlorination at both Tyr191 and Tyr195. Diagnostic mass shifts of +34 Da (monochlorination) and +68 Da (dichlorination) at $y_{6}^{+}$ and $y_{9}^{+}$ ions enabled unambiguous localization of modification sites. Orange dotted lines indicate expected mass shifts relative to the unmodified peptide. Retention time (RT) increased with the number of chlorine atoms: unmodified (47.7 min), singly chlorinated (∼52.4 min), and doubly chlorinated peptides (55.0–56.1 min). The position of modification had minimal effect on RT.

An illustrative example of the high site-level resolution achieved is peptide LPGGYGLPYTTGK, which contains two modifiable tyrosines (Tyr191 and Tyr195). MS/MS spectra confirmed the identity and localization of all mono- and di-chlorinated species across both isoforms and oxidation systems. Representative spectra are shown in Figure 2, and all annotated spectra for modified peptides and residues are provided in Figure S2. Chromatographic analysis further validated these assignments: retention time increased with the number of chlorine substitutions, reflecting increased hydrophobicity as detailed in Table S4. Importantly, both mono-chlorinated forms, whether modified at Tyr191 or Tyr195, exhibited nearly identical retention times, demonstrating that chlorination extent, rather than position, governs retention behavior. This dual validation, by MS/MS fragmentation and chromatographic shift, underscores the robustness of our approach in resolving complex oxidative proteoforms at the site level.

**Figure 3.**
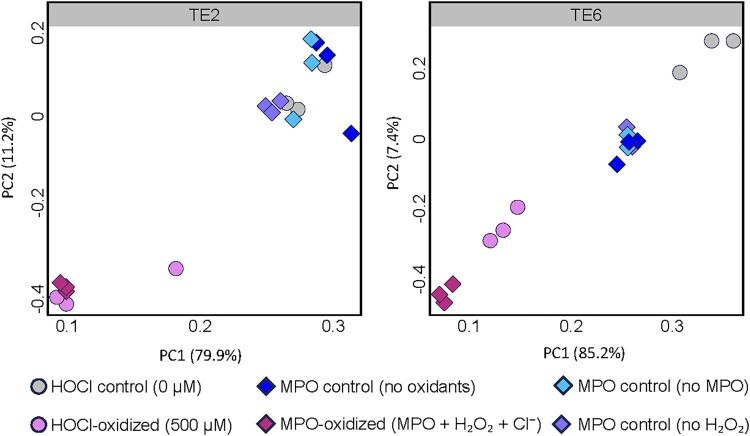
Principal component analysis (PCA) of oxidative modifications in tropoelastin isoforms TE2 and TE6. PCA plots for TE2 (left) and TE6 (right) show clear separation between oxidized samples (pink = 500 µM HOCl; magenta = $\mathrm{MPO}+H_{2}O_{2}+Cl^{-}$) and unoxidized controls (grey = 0 µM HOCl; dark blue = control TE; cyan = no MPO; light blue = no H_2_O_2_). Controls represent conditions in which one or more oxidant components were omitted. The close grouping of control samples demonstrates high reproducibility and minimal background variation, while oxidized samples cluster distinctly along PC1.

Finally, principal component analysis (PCA) confirmed that oxidative treatments led to distinct peptide modification profiles in both isoforms, with tight clustering of biological replicates and clear separation from control samples, as illustrated in Figure 3. MPO-induced modifications were dependent on the presence of H_2_O_2_, as no significant changes were observed in MPO-only or H_2_O_2_-only controls.

### Relative site occupancy and oxidation patterns

**Figure 4.**
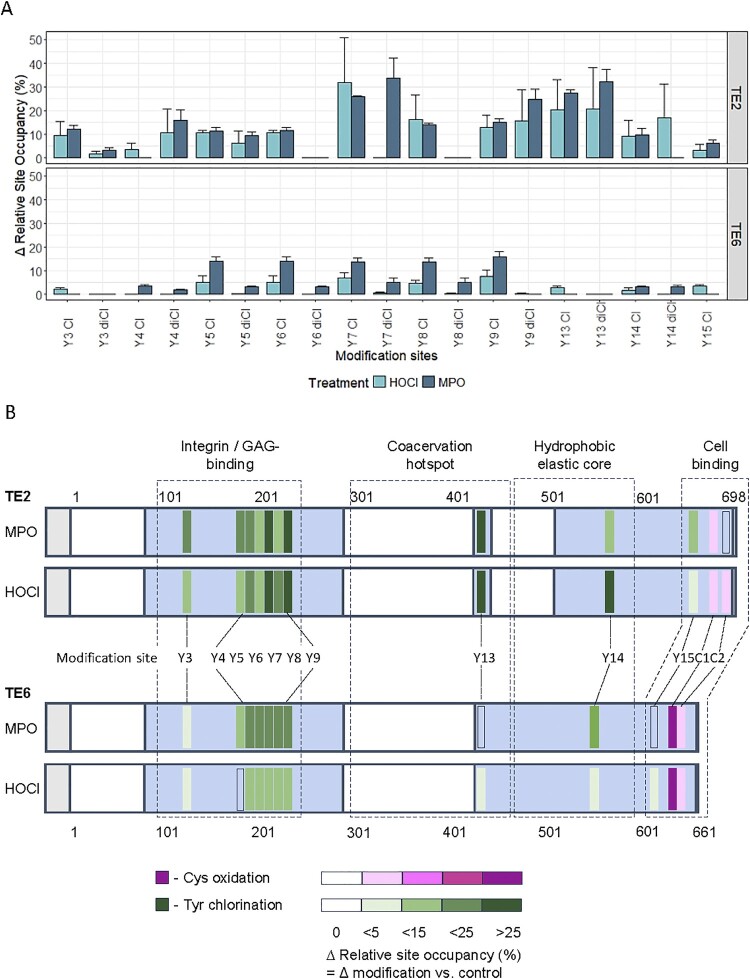
Isoform-specific oxidative modifications in tropoelastin: site occupancy and domain mapping. (A) Relative site occupancy (RSO) of mono- and di-chlorinated tyrosine residues in TE2 and TE6 following HOCl or MPO treatment. TE2 shows consistently higher chlorination levels than TE6, particularly at Tyr7 and Tyr13. Bars represent mean ± SD (*n* = 3). (B) Linear domain maps of TE2 and TE6 showing the location and extent of oxidative modifications. Chlorinated tyrosine (green) and oxidized cysteine (purple) are color-coded by RSO intensity, with white regions indicating segments not covered by LC–MS/MS. Dashed boxes mark major functional domains (Integrin/GAG-binding, Coacervation hotspot, Hydrophobic elastic core, Cell-binding region). These maps highlight isoform-dependent differences in oxidation patterns and their potential functional relevance.

Relative site occupancy (RSO) analysis revealed pronounced isoform- and residue-specific differences in oxidative modifications, as shown in Figures 4A and 4B. TE2 consistently showed higher levels of tyrosine chlorination than TE6, with particularly elevated RSO values at Tyr-7 and Tyr-13, both exceeding 20% under oxidative conditions. Di-chlorination (3,5-Cl_2_Tyr) was especially prevalent in TE2, approximately 88% more frequent than in TE6, indicating a greater propensity for extensive modification at individual tyrosine residues. In contrast, TE6 primarily exhibited mono-chlorination (3-ClTyr), consistent with limited secondary chlorination [42].

Tyr-13, located within the flexible hinge region involved in elastic fiber assembly, was robustly chlorinated in TE2 (RSO ∼20–32%) following both HOCl and MPO treatment, but showed minimal modification in TE6 (RSO ∼3% with HOCl only). This may reflect the absence of exon 23 in TE6, which likely reduces oxidant accessibility to this site. Similarly, Tyr-7 showed ∼70% higher modification in TE2, with an RSO of ∼34% under MPO treatment. Notably, di-chlorination at Tyr-7 was detected only under enzymatic oxidation, suggesting site-specific interactions between TE and MPO, as reported for other ECM proteins such as laminin and fibronectin (Nybo, Cai, et al. 2018, Nybo, Davies, et al. 2019).

The oxidation patterns of the Cys residues (which are all present in the form of disulfides in the parent protein) also differed markedly. This difference in reactivity may be due to the accessibility of the different disulfides, and the amino acids that comprise these, to the oxidizing species. In TE6, Cys-651 was extensively oxidized to sulfonic acid (Cys-SO_3_H), with RSO values of ∼43% for HOCl and ∼17% for MPO. This was accompanied by a significant drop in sulfinic acid (Cys-SO_2_H) (ΔRSO = −42%), consistent with progressive oxidation via halosulfonium and thiosulfinate intermediates leading to disulfide bond cleavage [47, 48]. Sulfonic acid formation is irreversible under biological conditions and may disrupt disulfide-dependent protein folding and function [49]. In contrast, the corresponding Cys-688 in TE2 showed only low-level sulfenic acid formation (∼3% RSO with HOCl) and no detectable oxidation with MPO, suggesting that TE6 is more vulnerable to disulfide bond cleavage.

This susceptibility is functionally relevant, as the single disulfide bond present in each isoform has been shown to be critical for tropoelastin folding, coacervation, and elastic fiber formation [50, 51], and its oxidation can impair TE-cell interactions [40, 52].

Figure 4A presents RSO values for mono- and di-chlorinated tyrosines in TE2 and TE6, highlighting higher chlorination in TE2. Figure 4B maps oxidative modification sites and their RSO intensities, annotated with functional domains. Notably, seven chlorinated tyrosines lie within the first 220 residues of the protein, particularly within hydrophobic domains 9, 12–14. Tyr-13, Tyr-14, and Tyr-15 are positioned at the termini of hydrophilic domains 21, 27, and 31, respectively. The distribution of oxidative modifications, especially within coacervation- and interaction-relevant regions, supports their likely contribution to isoform-specific differences in TE self-assembly.

### Coacervation behavior of oxidized tropoelastin isoforms

To assess the functional consequences of oxidation, we evaluated the coacervation behavior of TE2 and TE6 following HOCl treatment. Coacervation was monitored by light scattering in a turbidity assay under physiological salt and temperature conditions.

Under control conditions, TE2 and TE6 displayed markedly different intrinsic coacervation kinetics: TE6 rapidly reached high turbidity values, indicating a strong inherent propensity for self-assembly, whereas TE2 exhibited only minimal and slow coacervation. These baseline differences reflect isoform-specific structural and sequence features that influence the accessibility of aggregation-prone domains [2, 53]. Consequently, oxidation effects must be interpreted relative to each isoform's native behavior rather than compared directly.

**Figure 5.**
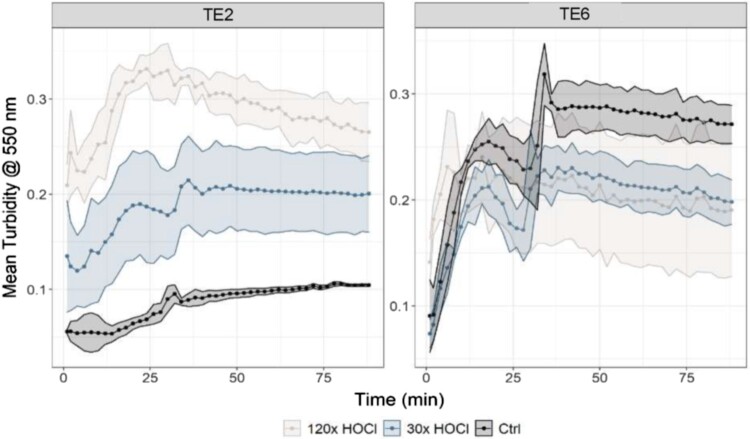
Coacervation Kinetics of Tropoelastin IsoformsTE2 and TE6 After HOCl Exposure. Turbidity measurements at 550 nm track coacervation over a 90-minute period. Coacervation is indicated by an increase in turbidity (0–0.3 A), reflecting the self-association of soluble tropoelastin molecules into coacervates. Under control conditions, TE2 (left panel) and TE6 (right panel) display distinct intrinsic behavior: TE6 assembles rapidly and extensively, whereas TE2 shows minimal and gradual coacervation. HOCl exposure induces a dose-dependent enhancement of TE2 coacervation but reduces TE6 assembly, with effects plateauing between doses. These opposite responses highlight isoform-specific oxidation sensitivity. Shaded regions show standard deviation from biological triplicates (*n* = 3).

In TE2, HOCl exposure induced a clear, dose-dependent enhancement of coacervation compared to untreated controls, which showed minimal assembly under the same conditions, as shown in the left panel of Figure 5. This indicates that tyrosine chlorination promotes TE2 self-assembly into larger aggregates. In contrast, TE6 displayed its highest coacervation capacity in the untreated state, with a slight but non-dose-dependent reduction following HOCl treatment, as illustrated in Figure 5, right panel. This decrease may result from disulfide bond oxidation rather than tyrosine modification, as TE6 exhibited extensive sulfonic acid formation at Cys-651.

Together, these data confirm that oxidative modifications have isoform-specific effects on tropoelastin assembly. Enhanced coacervation in TE2 aligns with high tyrosine chlorination, while reduced assembly in TE6 corresponds with irreversible disulfide bond cleavage, functionally validating the structural oxidation patterns observed by LC-MS/MS.

## Discussion

### Structural impact of oxidation on tropoelastin isoforms

Our findings reveal that oxidative modifications affect TE2 and TE6 differently at both structural and functional levels. TE6 exhibited extensive oxidation at Cys-651, leading to high levels of sulfonic acid formation, indicative of irreversible disulfide bond cleavage. In contrast, TE2 showed only minor, reversible oxidation at the corresponding cysteine (Cys-688), suggesting that its disulfide bond is more protected. This differential vulnerability likely results from local structural differences between the isoforms that affect oxidant accessibility.

Disulfide bond integrity is critical for tropoelastin folding and self-assembly into elastic fibers (Brown, Mecham et al. 1992). Therefore, the pronounced cleavage observed in TE6 may disrupt its structural stability and interfere with subsequent steps in elastogenesis.

### Isoform-specific susceptibility and functional consequences

Previous studies have shown that alternative splicing of key ELN exons (22, 23, 24, 26A, 32) generates tropoelastin isoforms with distinct coacervation kinetics [2, 54]. These exon-dependent variations alter the distribution of hydrophobic and cross-linking domains, thereby shaping self-assembly behavior. In this study, SDS-PAGE and LC-MS/MS confirmed that TE2 and TE6 differ in exon composition, particularly in the presence or absence of exons 22, 23, and 32. These sequence distinctions provide a molecular basis for their markedly different baseline coacervation kinetics: under control conditions, TE6 coacervated rapidly and extensively, whereas TE2 showed only limited and gradual aggregation. Because isoform-specific sequence context defines the intrinsic assembly capacity, oxidation-driven effects should be interpreted relative to each isoform's native behavior rather than compared directly.

TE2 demonstrated a higher extent of tyrosine chlorination, particularly formation of 3,5-dichlorotyrosine, with several sites reaching >20% relative occupancy. These modifications were especially prominent in regions involved in coacervation, including domains 12–14 and 21. In contrast, TE6 showed lower chlorination levels and favored mono-chlorinated species [25].

Functionally, these modifications had opposing effects on self-assembly. In turbidity assays, HOCl treatment enhanced coacervation in TE2, while the same treatment impaired TE6's ability to coacervate. This suggests that the chlorination of Tyr residues may facilitate TE2 aggregation, whereas the disruption of the disulfide bond in TE6 hinders assembly. Notably, the inverse relationship between tyrosine chlorination and disulfide bond oxidation supports the idea of competing modification pathways [42].

### Pathophysiological implications

Tropoelastin plays a key role in maintaining vascular integrity. In conditions such as atherosclerosis and aneurysm, oxidative stress, especially from MPO-derived HOCl, can contribute to ECM remodeling. Our data suggest that distinct TE isoforms may respond differently to oxidative environments, potentially influencing elastic fiber assembly and stability in vivo.

The intrinsic coacervation behavior of the isoforms is likely to have physiological relevance even before oxidation occurs. TE6's rapid self-assembly under physiological conditions may support efficient elastin deposition during tissue growth or repair, but its high susceptibility to oxidative disulfide cleavage suggests that in inflamed or oxidatively stressed tissues, TE6-rich regions could be particularly prone to loss of structural integrity and elasticity. In contrast, TE2's slower baseline coacervation may reflect a more controlled assembly process, but upon chlorination, its enhanced aggregation could lead to aberrant cross-linking or misaligned fiber deposition.

These isoform-specific susceptibilities may help explain the disrupted elastogenesis and altered fiber architecture observed in inflamed or diseased vessels [55], though it should be noted that these data were obtained from mRNA expression data, which may not correlate with the final protein products synthesized.

Taken together, these findings indicate that oxidative stress may not uniformly weaken elastin integrity but instead produce region-specific and isoform-dependent ECM outcomes. Tissues with differing isoform expression profiles may therefore exhibit distinct vulnerabilities to oxidative injury, contributing to the heterogeneous nature of vascular remodeling in disease.

### Outlook and future directions

Understanding isoform-specific oxidative modification patterns offers new insights into elastin regulation in health and disease. These findings support the potential use of tropoelastin oxidation products as biomarkers for vascular damage and highlight MPO as a therapeutic target for preserving ECM integrity.

Future studies should examine how these oxidative signatures correlate with disease severity in patient samples and explore the mechanical consequences of such modifications using 3D ECM models. Expanding this approach to other ECM proteins with isoform diversity, such as collagens, may uncover additional regulatory mechanisms governing matrix remodeling during inflammation and aging.

### Conclusions

This study demonstrates that tropoelastin isoforms TE2 and TE6 exhibit distinct susceptibilities to oxidative stress induced by HOCl and MPO-generated oxidants. TE6 is more prone to disulfide bond cleavage through extensive cysteine oxidation, while TE2 is preferentially chlorinated at tyrosine residues. These isoform-specific modifications alter the protein's self-assembly behavior: TE2 displays enhanced coacervation after oxidation, whereas TE6 shows a reduced capacity.

These findings highlight the importance of considering isoform context when studying extracellular matrix remodeling under inflammatory conditions. Since mature elastin is largely non-renewable, oxidative interference with tropoelastin assembly may have lasting consequences for tissue elasticity and vascular integrity.

Targeting MPO activity or blocking specific oxidative pathways may help preserve elastin structure and prevent ECM destabilization in cardiovascular diseases. Future research should explore whether these isoform-specific modifications serve as biomarkers of vascular pathology and whether modulating elastogenesis could be a viable therapeutic strategy.

## Supplementary Material

Supplemental Material

## Data Availability

The mass spectrometry proteomics data supporting the findings of this study have been deposited in the ProteomeXchange Consortium via the PRIDE partner repository with the dataset identifier PXD02206. All raw data, processed files, and search results associated with the oxidation experiments of tropoelastin isoforms (TE2 and TE6) are included. Supplementary figures and tables containing peptide maps, modification sites, MS/MS spectra, and quantitative data are provided as supplementary material (Figure S1-2, Tables S1–S8) and can be accessed at 10.5281/zenodo.17081172. Additional materials, including experimental protocols, are available from the corresponding author upon request.
