# Supplementary material for: Isoform-specific oxidative modifications of tropoelastin by HOCl and MPO alter protein self-assembly
Source: Redox Rep. 2025 Nov 28;30(1):2592409. doi: 10.1080/13510002.2025.2592409 (PMC12667354; doi:10.1080/13510002.2025.2592409)
Supplement: Supplemental Material [file YRER_A_2592409_SM5240.pdf]

Supplementary Figure S2. Annotated MS/MS Spectra of Oxidatively Modified Peptides in Tropoelastin Isoforms TE2 and TE6.

Isoform-specific oxidative modifications of tropoelastin by HOCl and MPO alter protein self-assembly

Karoline Lindgaard Mikkelsen[1], Tina Nybo[2], Michael J. Davies[2], Adelina Rogowska-Wrzesinska[1]*

[1]Department of Biochemistry and Molecular Biology and VILLUM Center for Bioanalytical Sciences, University of Southern Denmark, Campusvej 55, DK-5230, Odense M, Denmark

[2]Department of Biomedical Sciences, University of Copenhagen, Blegdamsvej 3, 2200 Copenhagen N, Denmark

•*Corresponding author: adelinar@bmb.sdu.dk, Department of Biochemistry and Molecular Biology and VILLUM Center for Bioanalytical Sciences, University of Southern Denmark, Campusvej 55, DK-5230, Odense M, Denmark

- **Supplementary Figure S2. Annotated MS/MS Spectra of Oxidatively Modified Peptides in Tropoelastin Isoforms TE2 and TE6.** This figure presents high-resolution MS/MS spectra for all peptides identified with oxidative modifications in TE2 and TE6 following treatment with HOCl or the MPO–H_2O_2–Cl^- system. Fragmentation spectra include site-specific annotations of chlorinated tyrosines (3-ClTyr, 3,5-Cl_2Tyr) and oxidized cysteines (Cys–SOH, –SO_2H, –SO_3H). Assigned b- and y-ions are labeled, and diagnostic mass shifts corresponding to each modification are highlighted. Isotopic patterns were used to confirm chlorination events, and all spectra were manually validated. This dataset supports confident localization of oxidative modifications at single-residue resolution.

Tropoelastin Isoform 2

Treated with HOCl

VPGVGLPGVYPGGVLPGAR

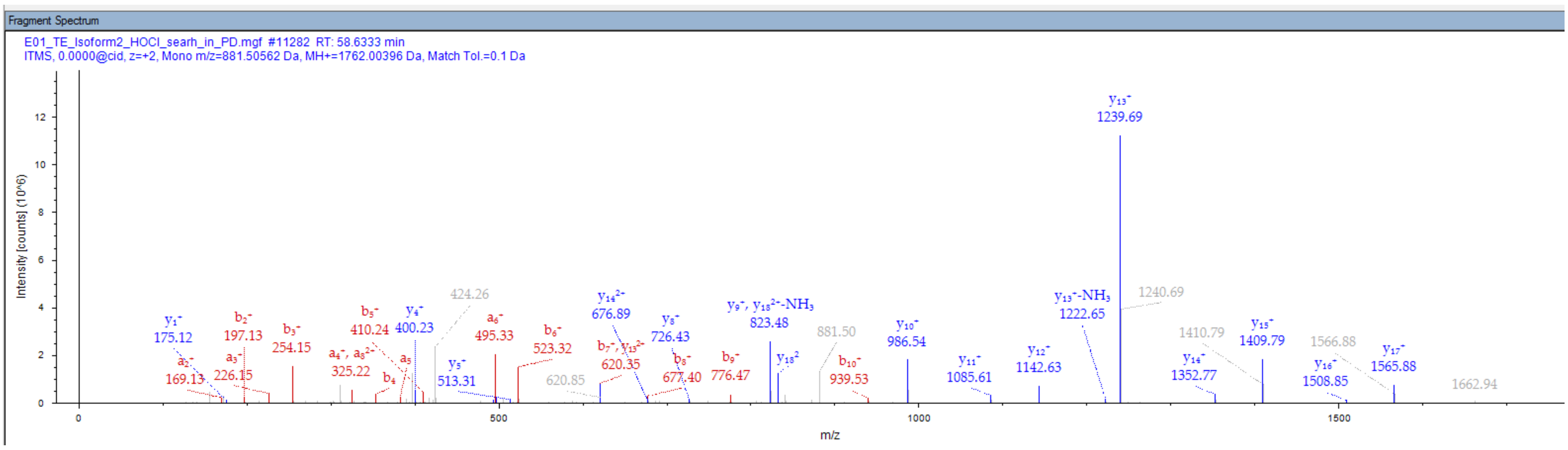

#1	a⁺	a²⁺	b⁺	b²⁺	Seq.	y⁺	y²⁺	#2
1	72.08078	36.54403	100.07569	50.54148	V			19
2	169.13354	85.07041	197.12845	99.06787	P	1662.93260	831.96994	18
3	226.15500	113.58114	254.14992	127.57860	G	1565.87984	783.44356	17
4	325.22342	163.11535	353.21833	177.11280	V	1508.85837	754.93283	16
5	382.24488	191.62608	410.23980	205.62354	G	1409.78996	705.39862	15
6	495.32894	248.16811	523.32386	262.16557	L	1352.76850	676.88789	14
7	592.38171	296.69449	620.37662	310.69195	P	1239.68443	620.34585	13
8	649.40317	325.20522	677.39809	339.20268	G	1142.63167	571.81947	12
9	748.47159	374.73943	776.46650	388.73689	V	1085.61020	543.30874	11
10	911.53491	456.27110	939.52983	470.26855	Y	986.54179	493.77453	10
11	1008.58768	504.79748	1036.58259	518.79493	P	823.47846	412.24287	9
12	1065.60914	533.30821	1093.60406	547.30567	G	726.42570	363.71649	8
13	1122.63061	561.81894	1150.62552	575.81640	G	669.40423	335.20576	7
14	1221.69902	611.35315	1249.69393	625.35061	V	612.38277	306.69502	6
15	1334.78308	667.89518	1362.77800	681.89264	L	513.31436	257.16082	5
16	1431.83585	716.42156	1459.83076	730.41902	P	400.23029	200.61879	4
17	1488.85731	744.93229	1516.85223	758.92975	G	303.17753	152.09240	3
18	1559.89442	780.45085	1587.88934	794.44831	A	246.15607	123.58167	2
19					R	175.11895	88.06311	1

VPGVGLPGVYPGGVLPGAR, Y10-Chlorination (33.96103 Da)

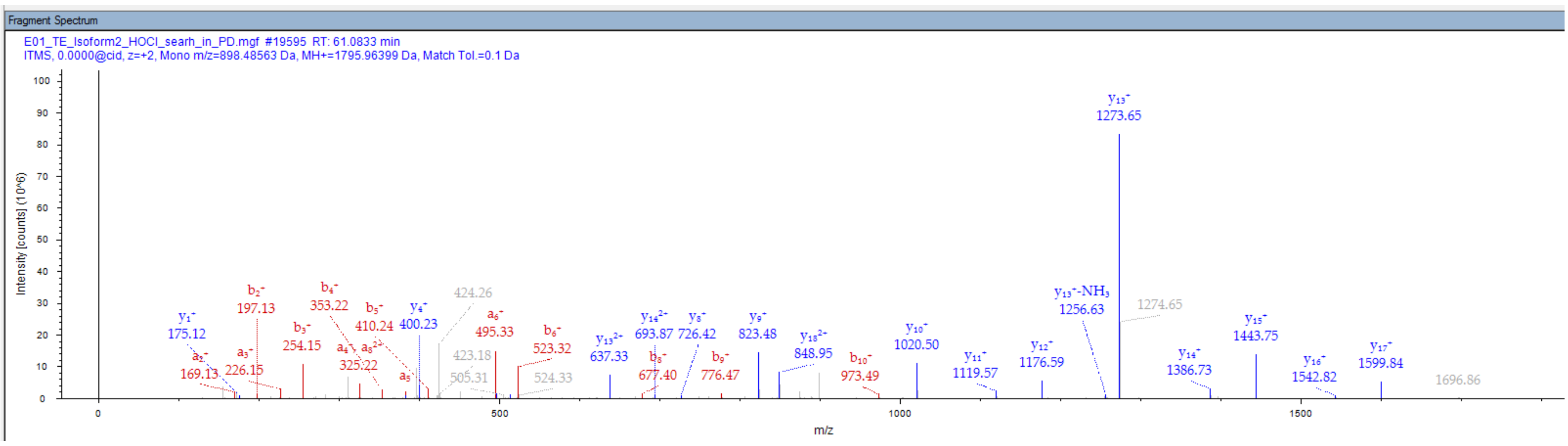

VPGVGLPGVYPGGVLPGAR, Y10-dichlorination (67.92206 Da)

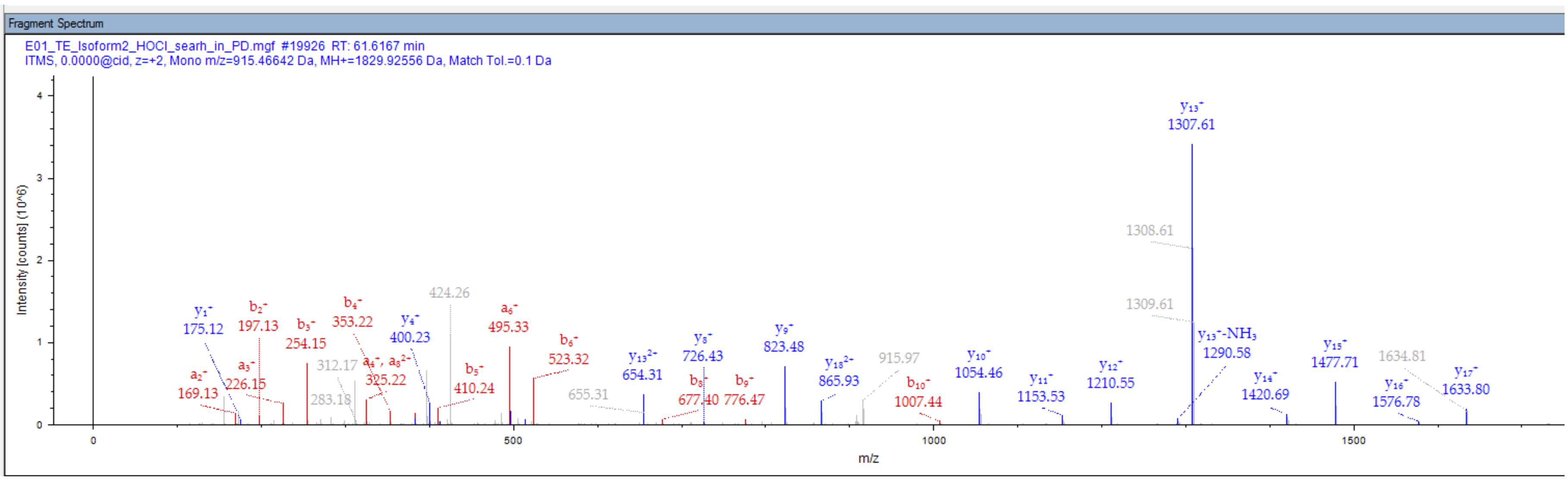

Ion Series | Neutral Losses | Precursor Ions | Internal Fragments

#1	a⁺	a²⁺	b⁺	b²⁺	Seq.	y⁺	y²⁺	#2
1	72.08078	36.54403	100.07569	50.54148	V			19
2	169.13354	85.07041	197.12845	99.06787	P	1730.85466	865.93097	18
3	226.15500	113.58114	254.14992	127.57860	G	1633.80189	817.40458	17
4	325.22342	163.11535	353.21833	177.11280	V	1576.78043	788.89385	16
5	382.24488	191.62608	410.23980	205.62354	G	1477.71201	739.35965	15
6	495.32894	248.16811	523.32386	262.16557	L	1420.69055	710.84891	14
7	592.38171	296.69449	620.37662	310.69195	P	1307.60649	654.30688	13
8	649.40317	325.20522	677.39809	339.20268	G	1210.55372	605.78050	12
9	748.47159	374.73943	776.46650	388.73689	V	1153.53226	577.26977	11
10	979.45697	490.23212	1007.45188	504.22958	Y-dichlorin...	1054.46385	527.73556	10
11	1076.50973	538.75851	1104.50465	552.75596	P	823.47846	412.24287	9
12	1133.53120	567.26924	1161.52611	581.26669	G	726.42570	363.71649	8
13	1190.55266	595.77997	1218.54758	609.77743	G	669.40423	335.20576	7
14	1289.62107	645.31418	1317.61599	659.31163	V	612.38277	306.69502	6
15	1402.70514	701.85621	1430.70005	715.85366	L	513.31436	257.16082	5
16	1499.75790	750.38259	1527.75282	764.38005	P	400.23029	200.61879	4
17	1556.77937	778.89332	1584.77428	792.89078	G	303.17753	152.09240	3
18	1627.81648	814.41188	1655.81139	828.40934	A	246.15607	123.58167	2
19					R	175.11895	88.06311	1

APGVGGAFAGIPGVGPFGGPQPGVPLG YPIK

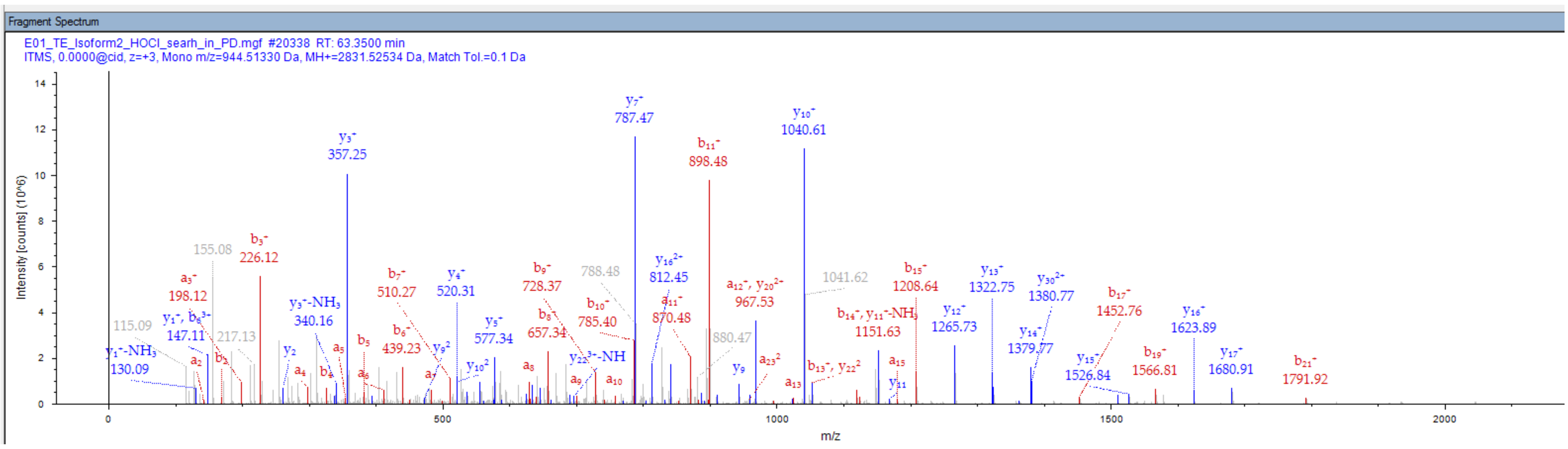

Ion Series | Neutral Losses | Precursor Ions | Internal Fragments

#1	a⁺	a²⁺	a³⁺	b⁺	b²⁺	b³⁺	Seq.	y⁺	y²⁺	y³⁺	#2
1	44.04948	22.52838	15.35468	72.04439	36.52583	24.68631	A				31
2	141.10224	71.05476	47.70560	169.09715	85.05222	57.03724	P	2760.48718	1380.74723	920.83391	30
3	198.12370	99.56549	66.71275	226.11862	113.56295	76.04439	G	2663.43442	1332.22085	888.48299	29
4	297.19212	149.09970	99.73556	325.18703	163.09715	109.06720	V	2606.41295	1303.71012	869.47584	28
5	354.21358	177.61043	118.74271	382.20850	191.60789	128.07435	G	2507.34454	1254.17591	836.45303	27
6	411.23504	206.12116	137.74987	439.22996	220.11862	147.08150	G	2450.32308	1225.66518	817.44588	26
7	482.27216	241.63972	161.42890	510.26707	255.63717	170.76054	A	2393.30161	1197.15445	798.43872	25
8	629.34057	315.17392	210.45171	657.33549	329.17138	219.78335	F	2322.26450	1161.63589	774.75968	24
9	700.37769	350.69248	234.13075	728.37260	364.68994	243.46238	A	2175.19609	1088.10168	725.73688	23
10	757.39915	379.20321	253.13790	785.39406	393.20067	262.46954	G	2104.15897	1052.58312	702.05784	22
11	870.48321	435.74525	290.83259	898.47813	449.74270	300.16423	I	2047.13751	1024.07239	683.05069	21
12	967.53598	484.27163	323.18351	995.53089	498.26908	332.51515	P	1934.05344	967.53036	645.35600	20
13	1024.55744	512.78236	342.19066	1052.55236	526.77982	351.52230	G	1837.00068	919.00398	613.00508	19
14	1123.62585	562.31657	375.21347	1151.62077	576.31402	384.54511	V	1779.97922	890.49325	593.99792	18
15	1180.64732	590.82730	394.22062	1208.64223	604.82475	403.55226	G	1680.91080	840.95904	560.97512	17
16	1277.70008	639.35368	426.57155	1305.69500	653.35114	435.90318	P	1623.88934	812.44831	541.96796	16
17	1424.76850	712.88789	475.59435	1452.76341	726.88534	484.92599	F	1526.83658	763.92193	509.61704	15
18	1481.78996	741.39862	494.60150	1509.78487	755.39608	503.93314	G	1379.76816	690.38772	460.59424	14
19	1538.81142	769.90935	513.60866	1566.80634	783.90681	522.94030	G	1322.74670	661.87699	441.58708	13
20	1635.86419	818.43573	545.95958	1663.85910	832.43319	555.29122	P	1265.72523	633.36626	422.57993	12
21	1763.92276	882.46502	588.64577	1791.91768	896.46248	597.97741	Q	1168.67247	584.83987	390.22901	11
22	1860.97553	930.99140	620.99669	1888.97044	944.98886	630.32833	P	1040.61389	520.81058	347.54282	10
23	1917.99699	959.50213	640.00385	1945.99191	973.49959	649.33549	G	943.56113	472.28420	315.19189	9
24	2017.06541	1009.03634	673.02665	2045.06032	1023.03380	682.35829	V	886.53967	443.77347	296.18474	8
25	2114.11817	1057.56272	705.37757	2142.11308	1071.56018	714.70921	P	787.47125	394.23926	263.16194	7
26	2227.20223	1114.10476	743.07226	2255.19715	1128.10221	752.40390	L	690.41849	345.71288	230.81101	6
27	2284.22370	1142.61549	762.07942	2312.21861	1156.61294	771.41106	G	577.33442	289.17085	193.11633	5
28	2447.28703	1224.14715	816.43386	2475.28194	1238.14461	825.76550	Y	520.31296	260.66012	174.10917	4
29	2544.33979	1272.67353	848.78478	2572.33470	1286.67099	858.11642	P	357.24963	179.12845	119.75473	3
30	2657.42385	1329.21557	886.47947	2685.41877	1343.21302	895.81111	I	260.19687	130.60207	87.40381	2
31							K	147.11280	74.06004	49.70912	1

APGVGGAFAGIPGVGPFGGPQPGVPLGYPIK, Y28-Chlorination (33.96103 Da)

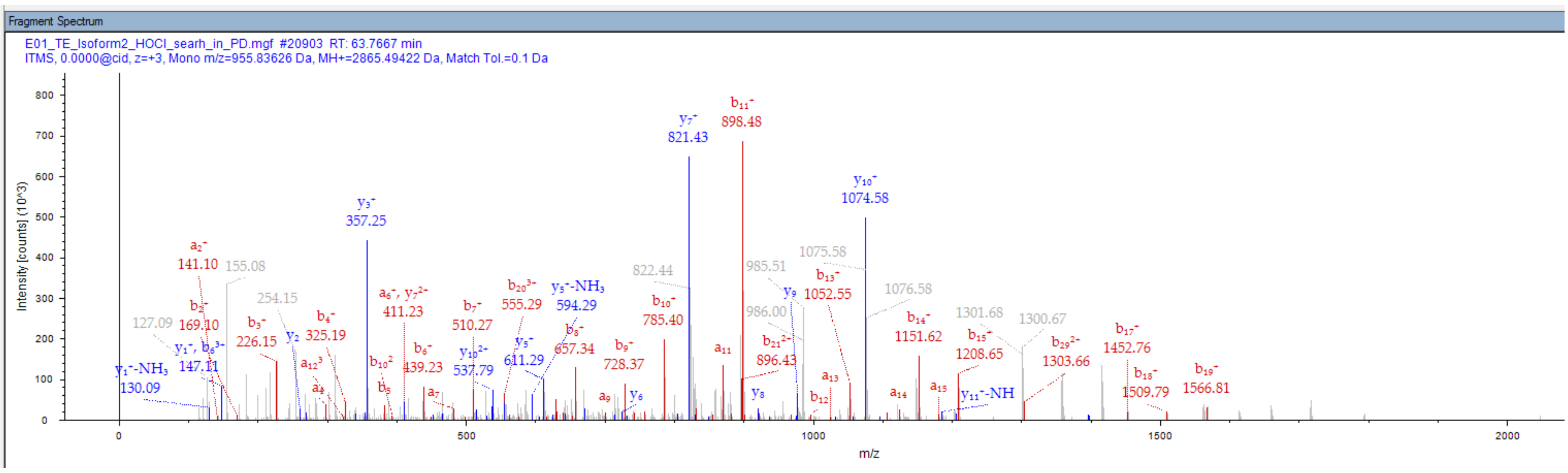

Fragment Matches

Value Type: Theo. Mass [Da]

Ion Series | Neutral Losses | Precursor Ions | Internal Fragments

#1	a⁺	a²⁺	a³⁺	b⁺	b²⁺	b³⁺	Seq.	y⁺	y²⁺	y³⁺	#2
1	44.04948	22.52838	15.35468	72.04439	36.52583	24.68631	A				31
2	141.10224	71.05476	47.70560	169.09715	85.05222	57.03724	P	2794.44821	1397.72774	932.15425	30
3	198.12370	99.56549	66.71275	226.11862	113.56295	76.04439	G	2697.39545	1349.20136	899.80333	29
4	297.19212	149.09970	99.73556	325.18703	163.09715	109.06720	V	2640.37398	1320.69063	880.79618	28
5	354.21358	177.61043	118.74271	382.20850	191.60789	128.07435	G	2541.30557	1271.15642	847.77337	27
6	411.23504	206.12116	137.74987	439.22996	220.11862	147.08150	G	2484.28411	1242.64569	828.76622	26
7	482.27216	241.63972	161.42890	510.26707	255.63717	170.76054	A	2427.26264	1214.13496	809.75906	25
8	629.34057	315.17392	210.45171	657.33549	329.17138	219.78335	F	2356.22553	1178.61640	786.08003	24
9	700.37769	350.69248	234.13075	728.37260	364.68994	243.46238	A	2209.15711	1105.08220	737.05722	23
10	757.39915	379.20321	253.13790	785.39406	393.20067	262.46954	G	2138.12000	1069.56364	713.37818	22
11	870.48321	435.74525	290.83259	898.47813	449.74270	300.16423	I	2081.09854	1041.05291	694.37103	21
12	967.53598	484.27163	323.18351	995.53089	498.26908	332.51515	P	1968.01447	984.51087	656.67634	20
13	1024.55744	512.78236	342.19066	1052.55236	526.77982	351.52230	G	1870.96171	935.98449	624.32542	19
14	1123.62585	562.31657	375.21347	1151.62077	576.31402	384.54511	V	1813.94025	907.47376	605.31827	18
15	1180.64732	590.82730	394.22062	1208.64223	604.82475	403.55226	G	1714.87183	857.93955	572.29546	17
16	1277.70008	639.35368	426.57155	1305.69500	653.35114	435.90318	P	1657.85037	829.42882	553.28831	16
17	1424.76850	712.88789	475.59435	1452.76341	726.88534	484.92599	F	1560.79760	780.90244	520.93739	15
18	1481.78996	741.39862	494.60150	1509.78487	755.39608	503.93314	G	1413.72919	707.36823	471.91458	14
19	1538.81142	769.90935	513.60866	1566.80634	783.90681	522.94030	G	1356.70773	678.85750	452.90743	13
20	1635.86419	818.43573	545.95958	1663.85910	832.43319	555.29122	P	1299.68626	650.34677	433.90027	12
21	1763.92276	882.46502	588.64577	1791.91768	896.46248	597.97741	Q	1202.63350	601.82039	401.54935	11
22	1860.97553	930.99140	620.99669	1888.97044	944.98886	630.32833	P	1074.57492	537.79110	358.86316	10
23	1917.99699	959.50213	640.00385	1945.99191	973.49959	649.33549	G	977.52216	489.26472	326.51224	9
24	2017.06541	1009.03634	673.02665	2045.06032	1023.03380	682.35829	V	920.50069	460.75399	307.50508	8
25	2114.11817	1057.56272	705.37757	2142.11308	1071.56018	714.70921	P	821.43228	411.21978	274.48228	7
26	2227.20223	1114.10476	743.07226	2255.19715	1128.10221	752.40390	L	724.37952	362.69340	242.13136	6
27	2284.22370	1142.61549	762.07942	2312.21861	1156.61294	771.41106	G	611.29545	306.15136	204.43667	5
28	2481.24805	1241.12767	827.75420	2509.24297	1255.12512	837.08584	Y-Chlorinat...	554.27399	277.64063	185.42951	4
29	2578.30082	1289.65405	860.10512	2606.29573	1303.65150	869.43676	P	357.24963	179.12845	119.75473	3
30	2691.38488	1346.19608	897.79981	2719.37980	1360.19354	907.13145	I	260.19687	130.60207	87.40381	2
31							K	147.11280	74.06004	49.70912	1

APGVGGAFAGIPGVGPFGGPQPGVPLGYPIK, Y28-dichlorination (67.92206 Da)

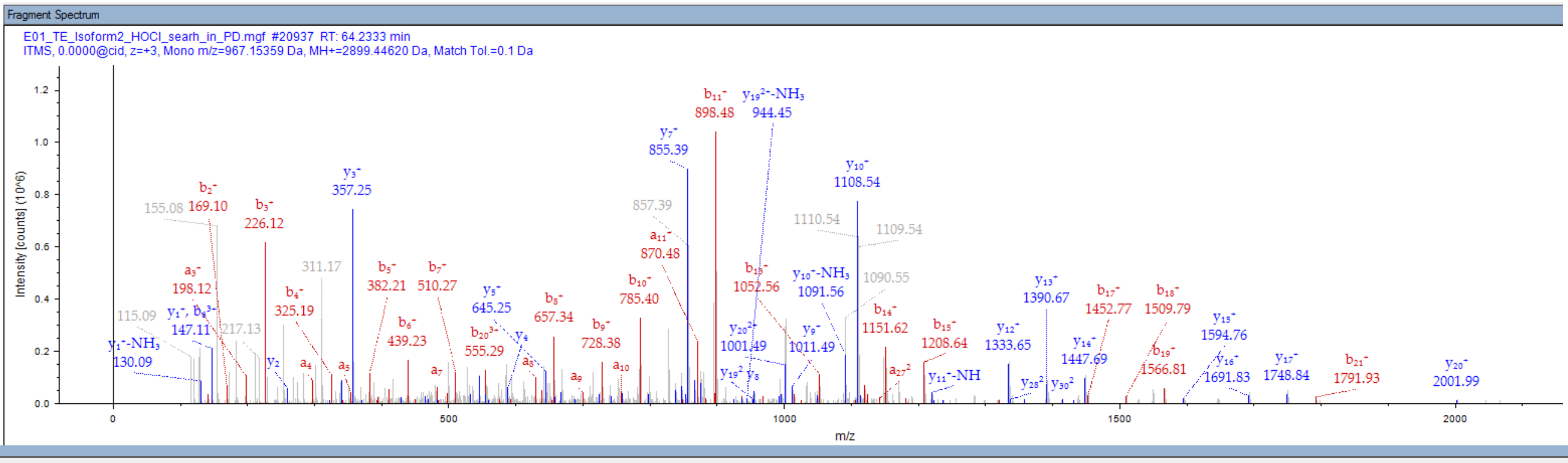

Value Type: Theo. Mass [Da]

Ion Series | Neutral Losses | Precursor Ions | Internal Fragments

#1	a^{+}	a^{2+}	a^{3+}	b^{+}	b^{2+}	b^{3+}	Seq.	y^{+}	y^{2+}	y^{3+}	#2
1	44.04948	22.52838	15.35468	72.04439	36.52583	24.68631	A				31
2	141.10224	71.05476	47.70560	169.09715	85.05222	57.03724	P	2828.40924	1414.70826	943.47460	30
3	198.12370	99.56549	66.71275	226.11862	113.56295	76.04439	G	2731.35647	1366.18188	911.12368	29
4	297.19212	149.09970	99.73556	325.18703	163.09715	109.06720	V	2674.33501	1337.67114	892.11652	28
5	354.21358	177.61043	118.74271	382.20850	191.60789	128.07435	G	2575.26660	1288.13694	859.09372	27
6	411.23504	206.12116	137.74987	439.22996	220.11862	147.08150	G	2518.24513	1259.62620	840.08656	26
7	482.27216	241.63972	161.42890	510.26707	255.63717	170.76054	A	2461.22367	1231.11547	821.07941	25
8	629.34057	315.17392	210.45171	657.33549	329.17138	219.78335	F	2390.18655	1195.59692	797.40037	24
9	700.37769	350.69248	234.13075	728.37260	364.68994	243.46238	A	2243.11814	1122.06271	748.37756	23
10	757.39915	379.20321	253.13790	785.39406	393.20067	262.46954	G	2172.08103	1086.54415	724.69853	22
11	870.48321	435.74525	290.83259	898.47813	449.74270	300.16423	I	2115.05956	1058.03342	705.69137	21
12	967.53598	484.27163	323.18351	995.53089	498.26908	332.51515	P	2001.97550	1001.49139	667.99668	20
13	1024.55744	512.78236	342.19066	1052.55236	526.77982	351.52230	G	1904.92274	952.96501	635.64576	19
14	1123.62585	562.31657	375.21347	1151.62077	576.31402	384.54511	V	1847.90127	924.45427	616.63861	18
15	1180.64732	590.82730	394.22062	1208.64223	604.82475	403.55226	G	1748.83286	874.92007	583.61580	17
16	1277.70008	639.35368	426.57155	1305.69500	653.35114	435.90318	P	1691.81139	846.40934	564.60865	16
17	1424.76850	712.88789	475.59435	1452.76341	726.88534	484.92599	F	1594.75863	797.88295	532.25773	15
18	1481.78996	741.39862	494.60150	1509.78487	755.39608	503.93314	G	1447.69022	724.34875	483.23492	14
19	1538.81142	769.90935	513.60866	1566.80634	783.90681	522.94030	G	1390.66875	695.83801	464.22777	13
20	1635.86419	818.43573	545.95958	1663.85910	832.43319	555.29122	P	1333.64729	667.32728	445.22061	12
21	1763.92276	882.46502	588.64577	1791.91768	896.46248	597.97741	Q	1236.59453	618.80090	412.86969	11
22	1860.97553	930.99140	620.99669	1888.97044	944.98886	630.32833	P	1108.53595	554.77161	370.18350	10
23	1917.99699	959.50213	640.00385	1945.99191	973.49959	649.33549	G	1011.48318	506.24523	337.83258	9
24	2017.06541	1009.03634	673.02665	2045.06032	1023.03380	682.35829	V	954.46172	477.73450	318.82542	8
25	2114.11817	1057.56272	705.37757	2142.11308	1071.56018	714.70921	P	855.39331	428.20029	285.80262	7
26	2227.20223	1114.10476	743.07226	2255.19715	1128.10221	752.40390	L	758.34054	379.67391	253.45170	6
27	2284.22370	1142.61549	762.07942	2312.21861	1156.61294	771.41106	G	645.25648	323.13188	215.75701	5
28	2515.20908	1258.10818	839.07454	2543.20400	1272.10564	848.40618	Y-dichlorin...	588.23502	294.62115	196.74986	4
29	2612.26184	1306.63456	871.42547	2640.25676	1320.63202	880.75710	P	357.24963	179.12845	119.75473	3
30	2725.34591	1363.17659	909.12015	2753.34082	1377.17405	918.45179	I	260.19687	130.60207	87.40381	2
31							K	147.11280	74.06004	49.70912	1

LPGGYGLPYTTGK,

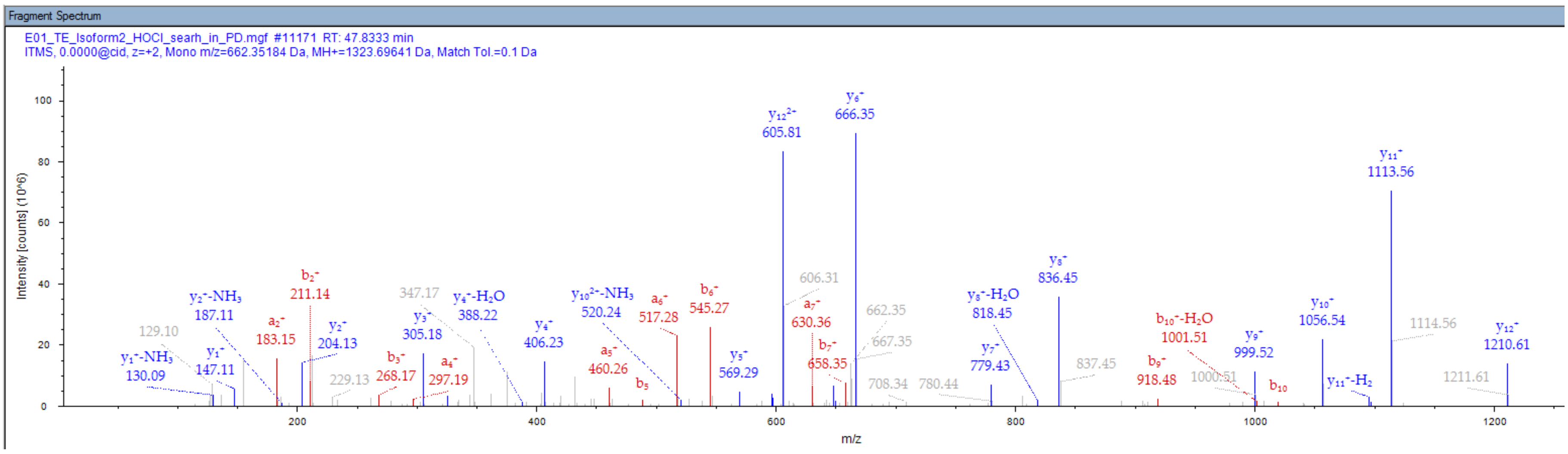

Ion Series | Neutral Losses | Precursor Ions | Internal Fragments

#1	a⁺	a²⁺	b⁺	b²⁺	Seq.	y⁺	y²⁺	#2
1	86.09643	43.55185	114.09134	57.54931	L			13
2	183.14919	92.07823	211.14410	106.07569	P	1210.61026	605.80877	12
3	240.17065	120.58897	268.16557	134.58642	G	1113.55750	557.28239	11
4	297.19212	149.09970	325.18703	163.09715	G	1056.53604	528.77166	10
5	460.25545	230.63136	488.25036	244.62882	Y	999.51457	500.26092	9
6	517.27691	259.14209	545.27182	273.13955	G	836.45124	418.72926	8
7	630.36097	315.68412	658.35589	329.68158	L	779.42978	390.21853	7
8	727.41374	364.21051	755.40865	378.20796	P	666.34572	333.67650	6
9	890.47707	445.74217	918.47198	459.73963	Y	569.29295	285.15011	5
10	991.52474	496.26601	1019.51966	510.26347	T	406.22962	203.61845	4
11	1092.57242	546.78985	1120.56734	560.78731	T	305.18195	153.09461	3
12	1149.59389	575.30058	1177.58880	589.29804	G	204.13427	102.57077	2
13					K	147.11280	74.06004	1

LPGGYGLPYTTGK, Y5-Chlorination (33.96103 Da)

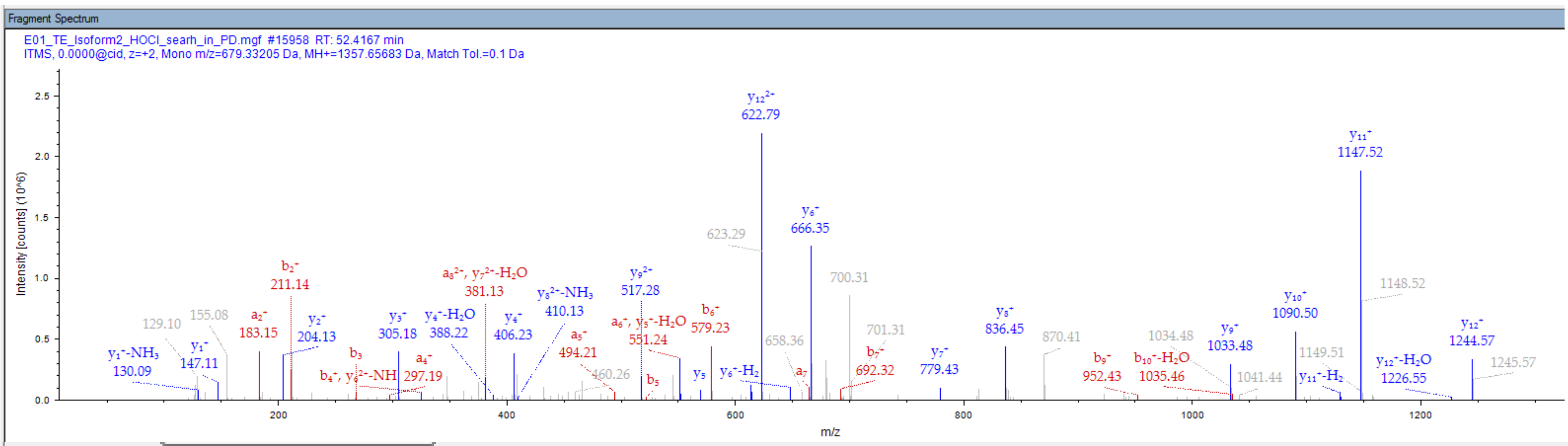

Ion Series | Neutral Losses | Precursor Ions | Internal Fragments

#1	a⁺	a²⁺	b⁺	b²⁺	Seq.	y⁺	y²⁺	#2
1	86.09643	43.55185	114.09134	57.54931	L			13
2	183.14919	92.07823	211.14410	106.07569	P	1244.57129	622.78928	12
3	240.17065	120.58897	268.16557	134.58642	G	1147.51853	574.26290	11
4	297.19212	149.09970	325.18703	163.09715	G	1090.49706	545.75217	10
5	494.21647	247.61188	522.21139	261.60933	Y-Chlorinat...	1033.47560	517.24144	9
6	551.23794	276.12261	579.23285	290.12006	G	836.45124	418.72926	8
7	664.32200	332.66464	692.31692	346.66210	L	779.42978	390.21853	7
8	761.37477	381.19102	789.36968	395.18848	P	666.34572	333.67650	6
9	924.43809	462.72269	952.43301	476.72014	Y	569.29295	285.15011	5
10	1025.48577	513.24652	1053.48069	527.24398	T	406.22962	203.61845	4
11	1126.53345	563.77036	1154.52836	577.76782	T	305.18195	153.09461	3
12	1183.55491	592.28110	1211.54983	606.27855	G	204.13427	102.57077	2
13					K	147.11280	74.06004	1

LPGGYGLPYTTGK, Y9-Chlorination (33.96103 Da)

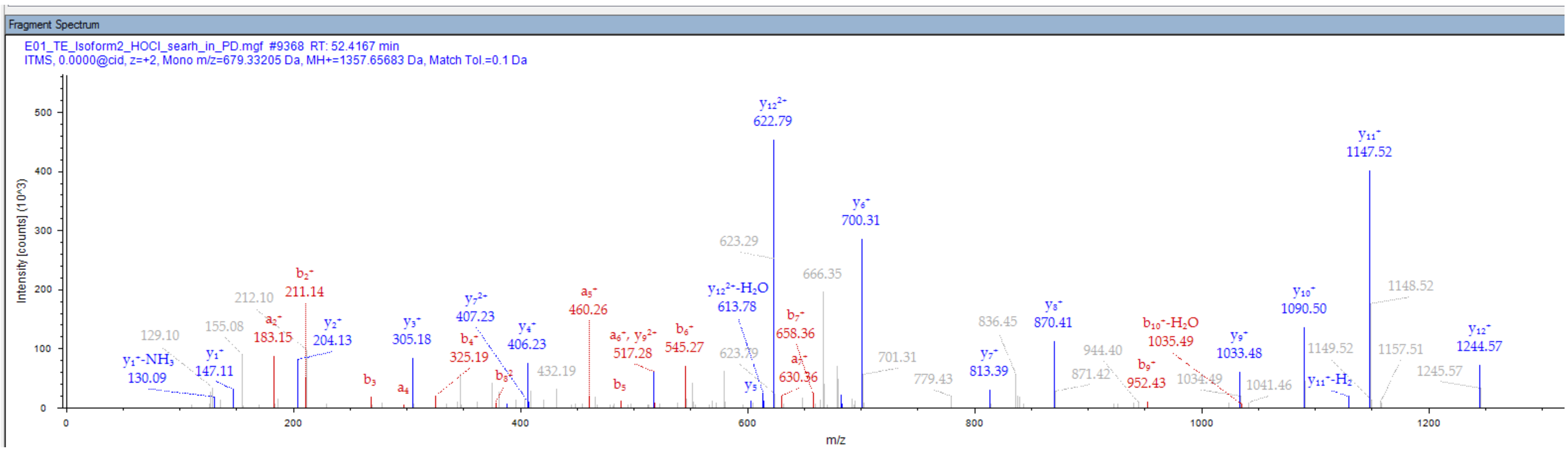

Ion Series | Neutral Losses | Precursor Ions | Internal Fragments

#1	a⁺	a²⁺	b⁺	b²⁺	Seq.	y⁺	y²⁺	#2
1	86.09643	43.55185	114.09134	57.54931	L			13
2	183.14919	92.07823	211.14410	106.07569	P	1244.57129	622.78928	12
3	240.17065	120.58897	268.16557	134.58642	G	1147.51853	574.26290	11
4	297.19212	149.09970	325.18703	163.09715	G	1090.49706	545.75217	10
5	460.25545	230.63136	488.25036	244.62882	Y	1033.47560	517.24144	9
6	517.27691	259.14209	545.27182	273.13955	G	870.41227	435.70977	8
7	630.36097	315.68412	658.35589	329.68158	L	813.39081	407.19904	7
8	727.41374	364.21051	755.40865	378.20796	P	700.30675	350.65701	6
9	924.43809	462.72269	952.43301	476.72014	Y-Chlorinat...	603.25398	302.13063	5
10	1025.48577	513.24652	1053.48069	527.24398	T	406.22962	203.61845	4
11	1126.53345	563.77036	1154.52836	577.76782	T	305.18195	153.09461	3
12	1183.55491	592.28110	1211.54983	606.27855	G	204.13427	102.57077	2
13					K	147.11280	74.06004	1

LPGGYGLPYTTGK, Y9-dichlorination (67.92206 Da)

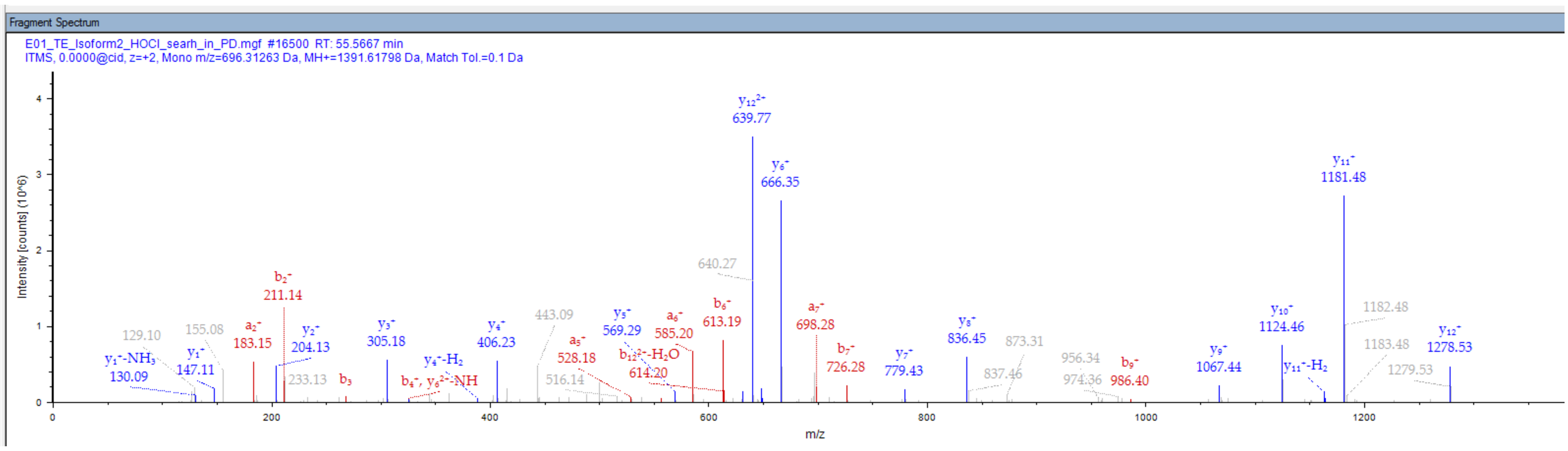

Ion Series | Neutral Losses | Precursor Ions | Internal Fragments

#1	a⁺	a²⁺	b⁺	b²⁺	Seq.	y⁺	y²⁺	#2
1	86.09643	43.55185	114.09134	57.54931	L			13
2	183.14919	92.07823	211.14410	106.07569	P	1278.53232	639.76980	12
3	240.17065	120.58897	268.16557	134.58642	G	1181.47956	591.24342	11
4	297.19212	149.09970	325.18703	163.09715	G	1124.45809	562.73268	10
5	528.17750	264.59239	556.17242	278.58985	Y-dichlorin...	1067.43663	534.22195	9
6	585.19896	293.10312	613.19388	307.10058	G	836.45124	418.72926	8
7	698.28303	349.64515	726.27794	363.64261	L	779.42978	390.21853	7
8	795.33579	398.17153	823.33071	412.16899	P	666.34572	333.67650	6
9	958.39912	479.70320	986.39404	493.70066	Y	569.29295	285.15011	5
10	1059.44680	530.22704	1087.44171	544.22450	T	406.22962	203.61845	4
11	1160.49448	580.75088	1188.48939	594.74833	T	305.18195	153.09461	3
12	1217.51594	609.26161	1245.51086	623.25907	G	204.13427	102.57077	2
13					K	147.11280	74.06004	1

LPGGYGLPYTTGK, Y5-Chlorination (33.96103 Da), Y9-Chlorination (33.96103 Da)

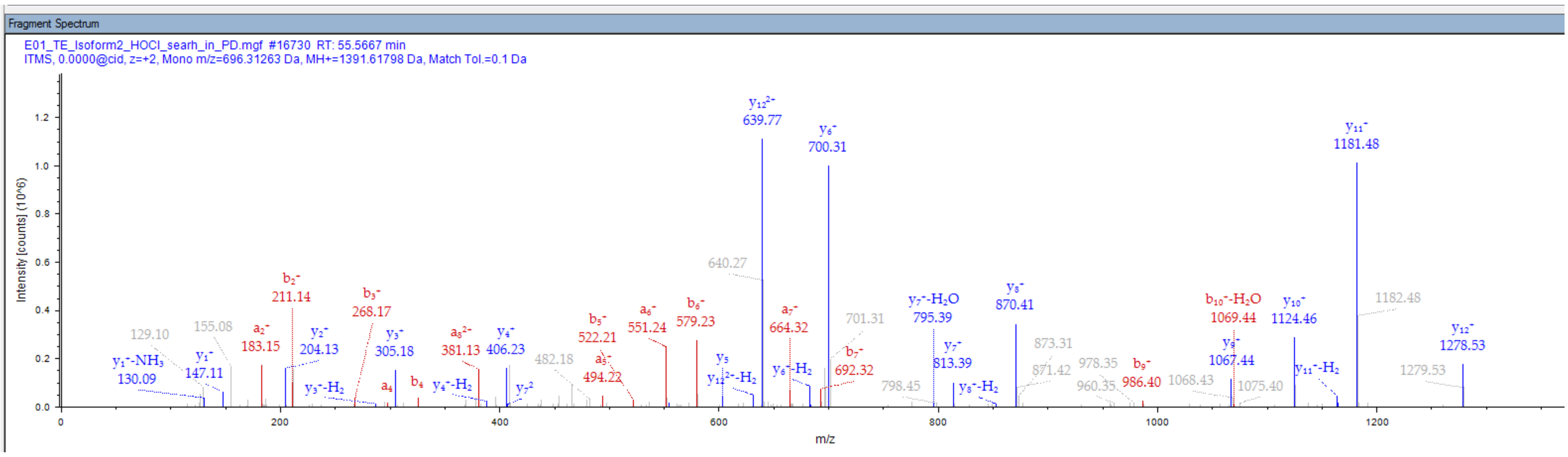

Ion Series | Neutral Losses | Precursor Ions | Internal Fragments

#1	a⁺	a²⁺	b⁺	b²⁺	Seq.	y⁺	y²⁺	#2
1	86.09643	43.55185	114.09134	57.54931	L			13
2	183.14919	92.07823	211.14410	106.07569	P	1278.53232	639.76980	12
3	240.17065	120.58897	268.16557	134.58642	G	1181.47956	591.24342	11
4	297.19212	149.09970	325.18703	163.09715	G	1124.45809	562.73268	10
5	494.21647	247.61188	522.21139	261.60933	Y-Chlorinat...	1067.43663	534.22195	9
6	551.23794	276.12261	579.23285	290.12006	G	870.41227	435.70977	8
7	664.32200	332.66464	692.31692	346.66210	L	813.39081	407.19904	7
8	761.37477	381.19102	789.36968	395.18848	P	700.30675	350.65701	6
9	958.39912	479.70320	986.39404	493.70066	Y-Chlorinat...	603.25398	302.13063	5
10	1059.44680	530.22704	1087.44171	544.22450	T	406.22962	203.61845	4
11	1160.49448	580.75088	1188.48939	594.74833	T	305.18195	153.09461	3
12	1217.51594	609.26161	1245.51086	623.25907	G	204.13427	102.57077	2
13					K	147.11280	74.06004	1

LPYGYGPGGVAGAAGK,

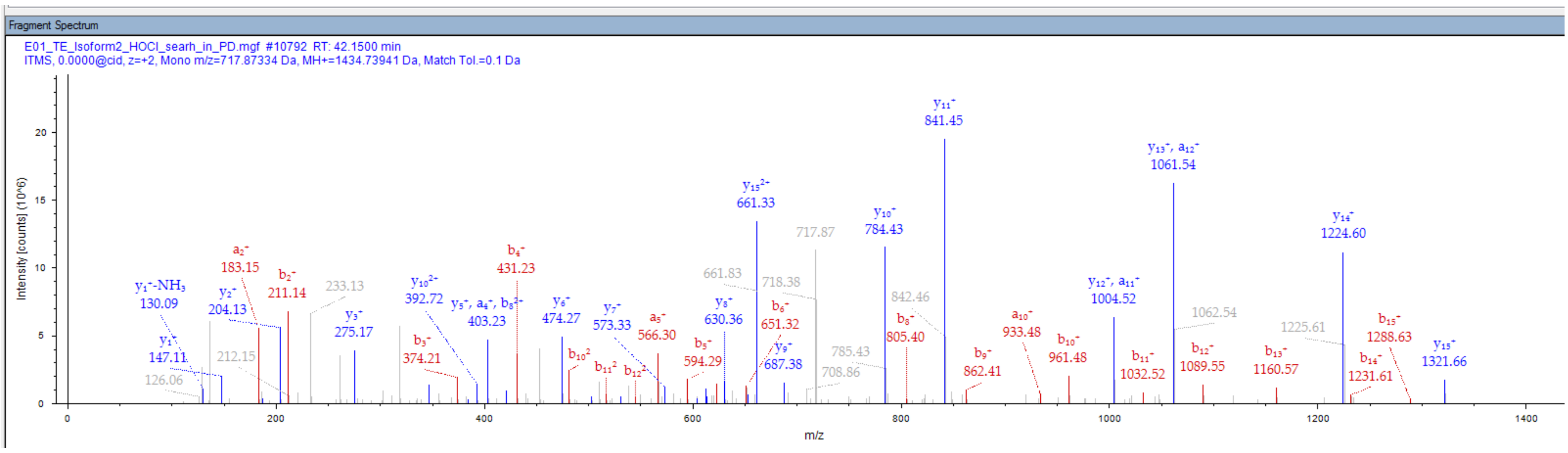

Ion Series | Neutral Losses | Precursor Ions | Internal Fragments

#1	a⁺	a²⁺	b⁺	b²⁺	Seq.	y⁺	y²⁺	#2
1	86.09643	43.55185	114.09134	57.54931	L			16
2	183.14919	92.07823	211.14410	106.07569	P	1321.65353	661.33040	15
3	346.21252	173.60990	374.20743	187.60735	Y	1224.60076	612.80402	14
4	403.23398	202.12063	431.22890	216.11809	G	1061.53743	531.27236	13
5	566.29731	283.65229	594.29223	297.64975	Y	1004.51597	502.76162	12
6	623.31877	312.16303	651.31369	326.16048	G	841.45264	421.22996	11
7	720.37154	360.68941	748.36645	374.68686	P	784.43118	392.71923	10
8	777.39300	389.20014	805.38792	403.19760	G	687.37841	344.19285	9
9	834.41447	417.71087	862.40938	431.70833	G	630.35695	315.68211	8
10	933.48288	467.24508	961.47779	481.24254	V	573.33549	287.17138	7
11	1004.51999	502.76363	1032.51491	516.76109	A	474.26707	237.63717	6
12	1061.54146	531.27437	1089.53637	545.27182	G	403.22996	202.11862	5
13	1132.57857	566.79292	1160.57348	580.79038	A	346.20850	173.60789	4
14	1203.61568	602.31148	1231.61060	616.30894	A	275.17138	138.08933	3
15	1260.63715	630.82221	1288.63206	644.81967	G	204.13427	102.57077	2
16					K	147.11280	74.06004	1

LPYGYGPGGVAGAAGK, Y3-Chlorination (33.96103 Da)

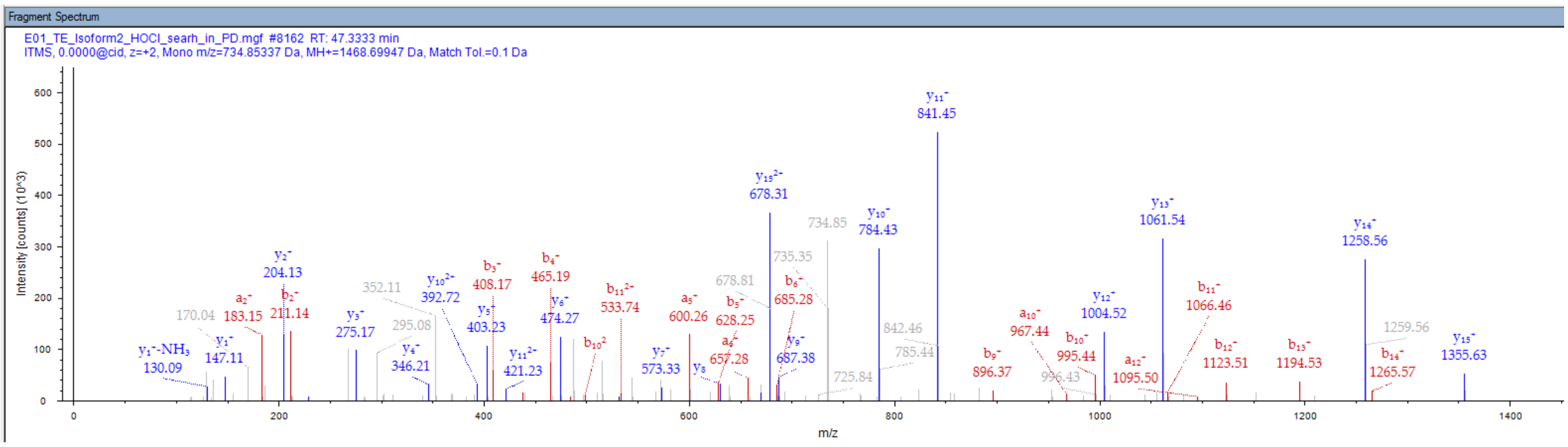

Ion Series | Neutral Losses | Precursor Ions | Internal Fragments

#1	a⁺	a²⁺	b⁺	b²⁺	Seq.	y⁺	y²⁺	#2
1	86.09643	43.55185	114.09134	57.54931	L			16
2	183.14919	92.07823	211.14410	106.07569	P	1355.61455	678.31092	15
3	380.17355	190.59041	408.16846	204.58787	Y-Chlorinat...	1258.56179	629.78453	14
4	437.19501	219.10114	465.18992	233.09860	G	1061.53743	531.27236	13
5	600.25834	300.63281	628.25325	314.63026	Y	1004.51597	502.76162	12
6	657.27980	329.14354	685.27472	343.14100	G	841.45264	421.22996	11
7	754.33257	377.66992	782.32748	391.66738	P	784.43118	392.71923	10
8	811.35403	406.18065	839.34894	420.17811	G	687.37841	344.19285	9
9	868.37549	434.69138	896.37041	448.68884	G	630.35695	315.68211	8
10	967.44391	484.22559	995.43882	498.22305	V	573.33549	287.17138	7
11	1038.48102	519.74415	1066.47594	533.74161	A	474.26707	237.63717	6
12	1095.50248	548.25488	1123.49740	562.25234	G	403.22996	202.11862	5
13	1166.53960	583.77344	1194.53451	597.77089	A	346.20850	173.60789	4
14	1237.57671	619.29199	1265.57163	633.28945	A	275.17138	138.08933	3
15	1294.59818	647.80273	1322.59309	661.80018	G	204.13427	102.57077	2
16					K	147.11280	74.06004	1

LPYGYGPGGVAGAAGK, Y5-Chlorination (33.96103 Da)

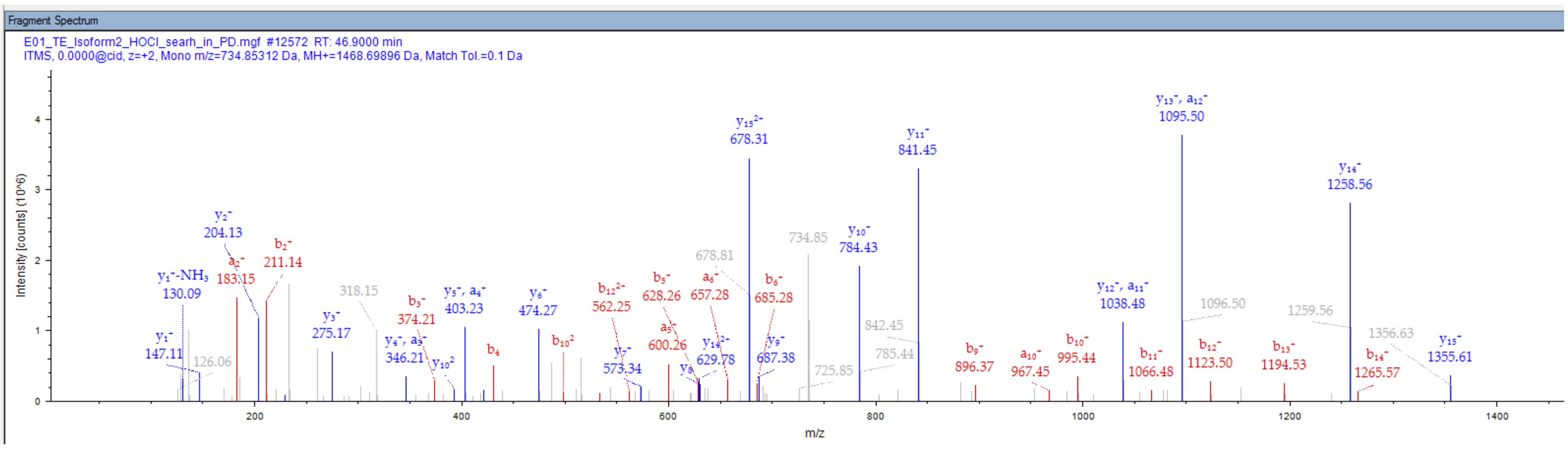

Ion Series | Neutral Losses | Precursor Ions | Internal Fragments

#1	a+	a2+	b+	b2+	Seq.	y+	y2+	#2
1	86.09643	43.55185	114.09134	57.54931	L			16
2	183.14919	92.07823	211.14410	106.07569	P	1355.61455	678.31092	15
3	346.21252	173.60990	374.20743	187.60735	Y	1258.56179	629.78453	14
4	403.23398	202.12063	431.22890	216.11809	G	1095.49846	548.25287	13
5	600.25834	300.63281	628.25325	314.63026	Y-Chlorinat...	1038.47700	519.74214	12
6	657.27980	329.14354	685.27472	343.14100	G	841.45264	421.22996	11
7	754.33257	377.66992	782.32748	391.66738	P	784.43118	392.71923	10
8	811.35403	406.18065	839.34894	420.17811	G	687.37841	344.19285	9
9	868.37549	434.69138	896.37041	448.68884	G	630.35695	315.68211	8
10	967.44391	484.22559	995.43882	498.22305	V	573.33549	287.17138	7
11	1038.48102	519.74415	1066.47594	533.74161	A	474.26707	237.63717	6
12	1095.50248	548.25488	1123.49740	562.25234	G	403.22996	202.11862	5
13	1166.53960	583.77344	1194.53451	597.77089	A	346.20850	173.60789	4
14	1237.57671	619.29199	1265.57163	633.28945	A	275.17138	138.08933	3
15	1294.59818	647.80273	1322.59309	661.80018	G	204.13427	102.57077	2
16					K	147.11280	74.06004	1

AGYPTGTGVGPQAAAAAAK

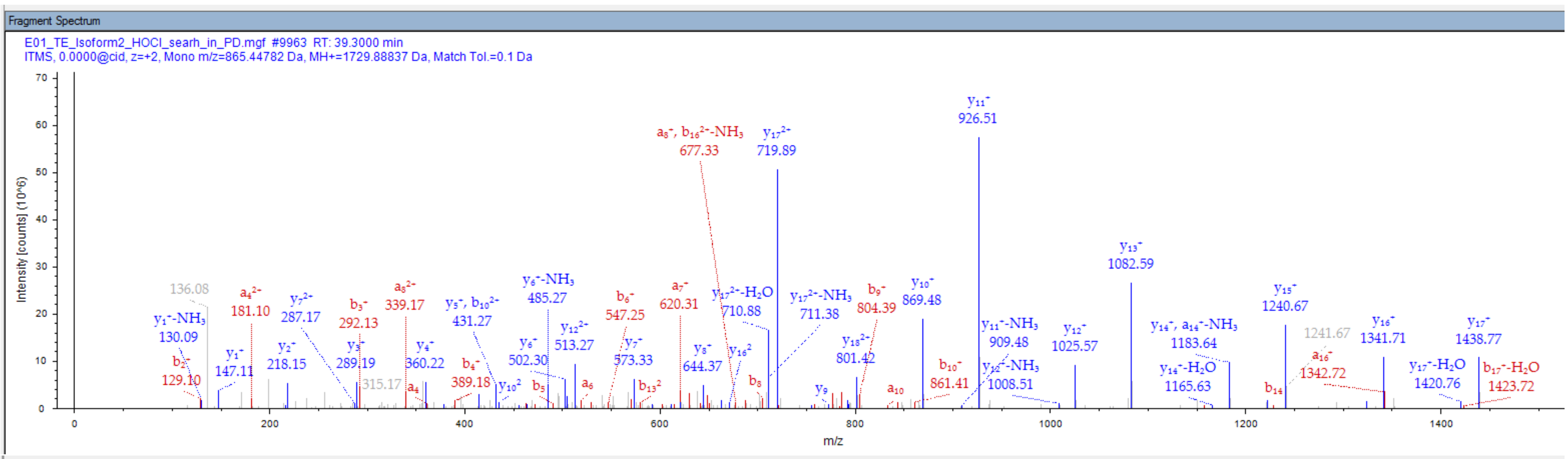

Ion Series | Neutral Losses | Precursor Ions | Internal Fragments

#1	a⁺	a²⁺	b⁺	b²⁺	Seq.	y⁺	y²⁺	#2
1	44.04948	22.52838	72.04439	36.52583	A			20
2	101.07094	51.03911	129.06585	65.03657	G	1658.84966	829.92847	19
3	264.13427	132.57077	292.12918	146.56823	Y	1601.82820	801.41774	18
4	361.18703	181.09715	389.18195	195.09461	P	1438.76487	719.88607	17
5	462.23471	231.62099	490.22962	245.61845	T	1341.71210	671.35969	16
6	519.25617	260.13173	547.25109	274.12918	G	1240.66443	620.83585	15
7	620.30385	310.65556	648.29877	324.65302	T	1183.64296	592.32512	14
8	677.32532	339.16630	705.32023	353.16375	G	1082.59528	541.80128	13
9	776.39373	388.70050	804.38864	402.69796	V	1025.57382	513.29055	12
10	833.41519	417.21124	861.41011	431.20869	G	926.50541	463.75634	11
11	930.46796	465.73762	958.46287	479.73507	P	869.48394	435.24561	10
12	1058.52653	529.76691	1086.52145	543.76436	Q	772.43118	386.71923	9
13	1129.56365	565.28546	1157.55856	579.28292	A	644.37260	322.68994	8
14	1200.60076	600.80402	1228.59568	614.80148	A	573.33549	287.17138	7
15	1271.63788	636.32258	1299.63279	650.32003	A	502.29837	251.65282	6
16	1342.67499	671.84113	1370.66990	685.83859	A	431.26126	216.13427	5
17	1413.71210	707.35969	1441.70702	721.35715	A	360.22415	180.61571	4
18	1484.74922	742.87825	1512.74413	756.87570	A	289.18703	145.09715	3
19	1555.78633	778.39680	1583.78125	792.39426	A	218.14992	109.57860	2
20					K	147.11280	74.06004	1

AGYPTGTGVGPQAAAAAAAK, Y3-Chlorination (33.96103 Da)

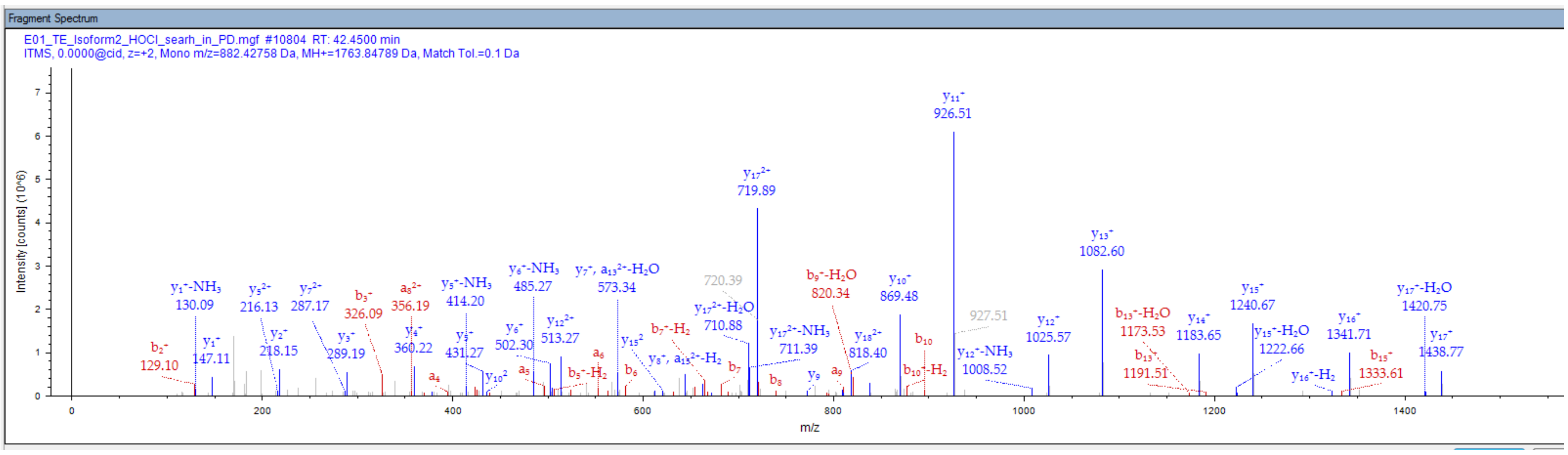

Ion Series | Neutral Losses | Precursor Ions | Internal Fragments

#1	a⁺	a²⁺	b⁺	b²⁺	Seq.	y⁺	y²⁺	#2
1	44.04948	22.52838	72.04439	36.52583	A			20
2	101.07094	51.03911	129.06585	65.03657	G	1692.81069	846.90898	19
3	298.09530	149.55129	326.09021	163.54874	Y-Chlorinat...	1635.78922	818.39825	18
4	395.14806	198.07767	423.14297	212.07513	P	1438.76487	719.88607	17
5	496.19574	248.60151	524.19065	262.59896	T	1341.71210	671.35969	16
6	553.21720	277.11224	581.21212	291.10970	G	1240.66443	620.83585	15
7	654.26488	327.63608	682.25979	341.63354	T	1183.64296	592.32512	14
8	711.28634	356.14681	739.28126	370.14427	G	1082.59528	541.80128	13
9	810.35476	405.68102	838.34967	419.67847	V	1025.57382	513.29055	12
10	867.37622	434.19175	895.37114	448.18921	G	926.50541	463.75634	11
11	964.42899	482.71813	992.42390	496.71559	P	869.48394	435.24561	10
12	1092.48756	546.74742	1120.48248	560.74488	Q	772.43118	386.71923	9
13	1163.52468	582.26598	1191.51959	596.26343	A	644.37260	322.68994	8
14	1234.56179	617.78453	1262.55670	631.78199	A	573.33549	287.17138	7
15	1305.59890	653.30309	1333.59382	667.30055	A	502.29837	251.65282	6
16	1376.63602	688.82165	1404.63093	702.81910	A	431.26126	216.13427	5
17	1447.67313	724.34020	1475.66805	738.33766	A	360.22415	180.61571	4
18	1518.71025	759.85876	1546.70516	773.85622	A	289.18703	145.09715	3
19	1589.74736	795.37732	1617.74227	809.37478	A	218.14992	109.57860	2
20					K	147.11280	74.06004	1

AGYPTGTGVGPQAAAAAAAK, Y3-dichlorination (67.92206 Da)

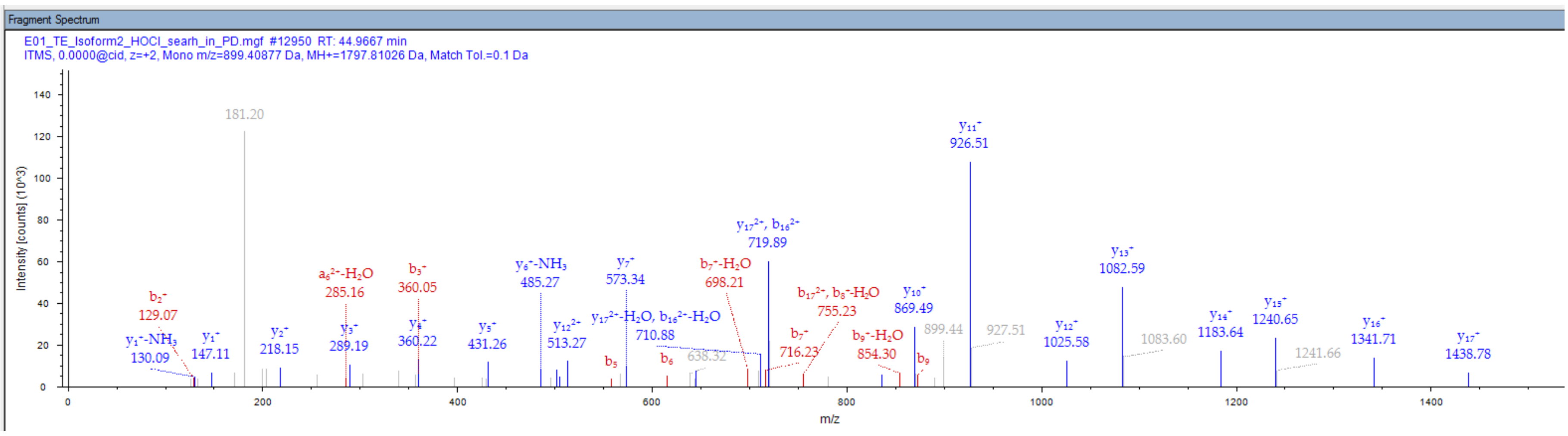

Ion Series | Neutral Losses | Precursor Ions | Internal Fragments

#1	a⁺	a²⁺	b⁺	b²⁺	Seq.	y⁺	y²⁺	#2
1	44.04948	22.52838	72.04439	36.52583	A			20
2	101.07094	51.03911	129.06585	65.03657	G	1726.77171	863.88950	19
3	332.05632	166.53180	360.05124	180.52926	Y-dichlorin...	1669.75025	835.37876	18
4	429.10909	215.05818	457.10400	229.05564	P	1438.76487	719.88607	17
5	530.15677	265.58202	558.15168	279.57948	T	1341.71210	671.35969	16
6	587.17823	294.09275	615.17314	308.09021	G	1240.66443	620.83585	15
7	688.22591	344.61659	716.22082	358.61405	T	1183.64296	592.32512	14
8	745.24737	373.12732	773.24229	387.12478	G	1082.59528	541.80128	13
9	844.31578	422.66153	872.31070	436.65899	V	1025.57382	513.29055	12
10	901.33725	451.17226	929.33216	465.16972	G	926.50541	463.75634	11
11	998.39001	499.69864	1026.38493	513.69610	P	869.48394	435.24561	10
12	1126.44859	563.72793	1154.44350	577.72539	Q	772.43118	386.71923	9
13	1197.48570	599.24649	1225.48062	613.24395	A	644.37260	322.68994	8
14	1268.52282	634.76505	1296.51773	648.76250	A	573.33549	287.17138	7
15	1339.55993	670.28360	1367.55485	684.28106	A	502.29837	251.65282	6
16	1410.59704	705.80216	1438.59196	719.79962	A	431.26126	216.13427	5
17	1481.63416	741.32072	1509.62907	755.31817	A	360.22415	180.61571	4
18	1552.67127	776.83927	1580.66619	790.83673	A	289.18703	145.09715	3
19	1623.70839	812.35783	1651.70330	826.35529	A	218.14992	109.57860	2
20					K	147.11280	74.06004	1

AGYPTGTGVGPQAAAAAAK, Y3-dichlorination (67.92206 Da)

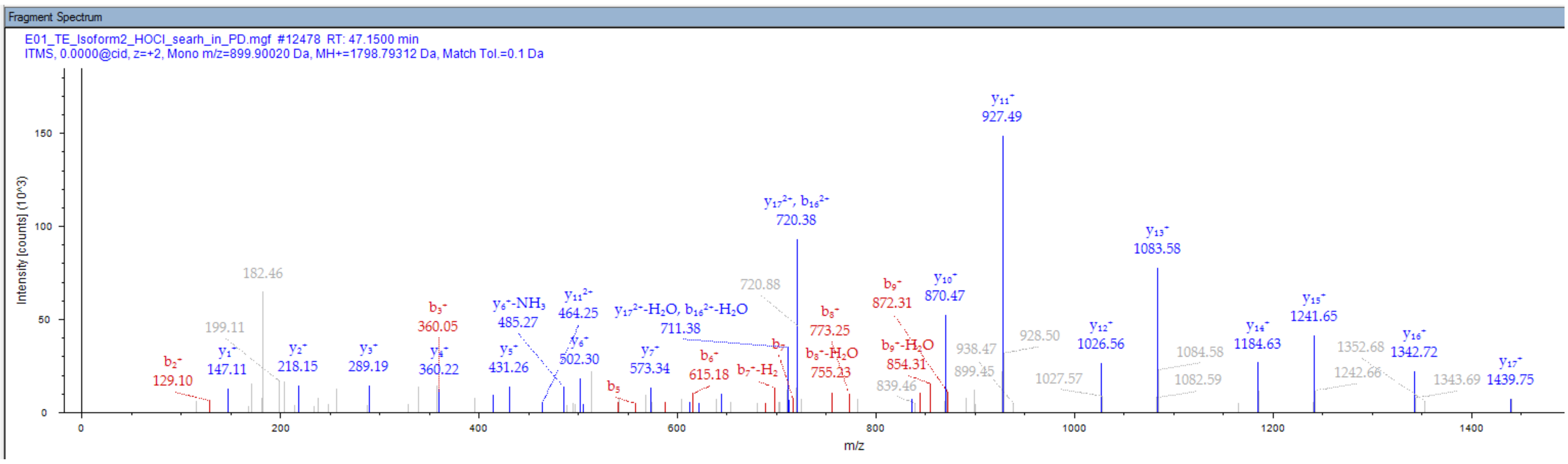

Ion Series | Neutral Losses | Precursor Ions | Internal Fragments

#1	a+	a2+	b+	b2+	Seq.	y+	y2+	#2
1	44.04948	22.52838	72.04439	36.52583	A			20
2	101.07094	51.03911	129.06585	65.03657	G	1727.75573	864.38150	19
3	332.05632	166.53180	360.05124	180.52926	Y-dichlorin...	1670.73427	835.87077	18
4	429.10909	215.05818	457.10400	229.05564	P	1439.74888	720.37808	17
5	530.15677	265.58202	558.15168	279.57948	T	1342.69612	671.85170	16
6	587.17823	294.09275	615.17314	308.09021	G	1241.64844	621.32786	15
7	688.22591	344.61659	716.22082	358.61405	T	1184.62698	592.81713	14
8	745.24737	373.12732	773.24229	387.12478	G	1083.57930	542.29329	13
9	844.31578	422.66153	872.31070	436.65899	V	1026.55784	513.78256	12
10	901.33725	451.17226	929.33216	465.16972	G	927.48942	464.24835	11
11	998.39001	499.69864	1026.38493	513.69610	P	870.46796	435.73762	10
12	1127.43261	564.21994	1155.42752	578.21740	Q-Deamid...	773.41519	387.21124	9
13	1198.46972	599.73850	1226.46463	613.73596	A	644.37260	322.68994	8
14	1269.50683	635.25705	1297.50175	649.25451	A	573.33549	287.17138	7
15	1340.54395	670.77561	1368.53886	684.77307	A	502.29837	251.65282	6
16	1411.58106	706.29417	1439.57598	720.29163	A	431.26126	216.13427	5
17	1482.61817	741.81273	1510.61309	755.81018	A	360.22415	180.61571	4
18	1553.65529	777.33128	1581.65020	791.32874	A	289.18703	145.09715	3
19	1624.69240	812.84984	1652.68732	826.84730	A	218.14992	109.57860	2
20					K	147.11280	74.06004	1

YGVGTPAAAAK

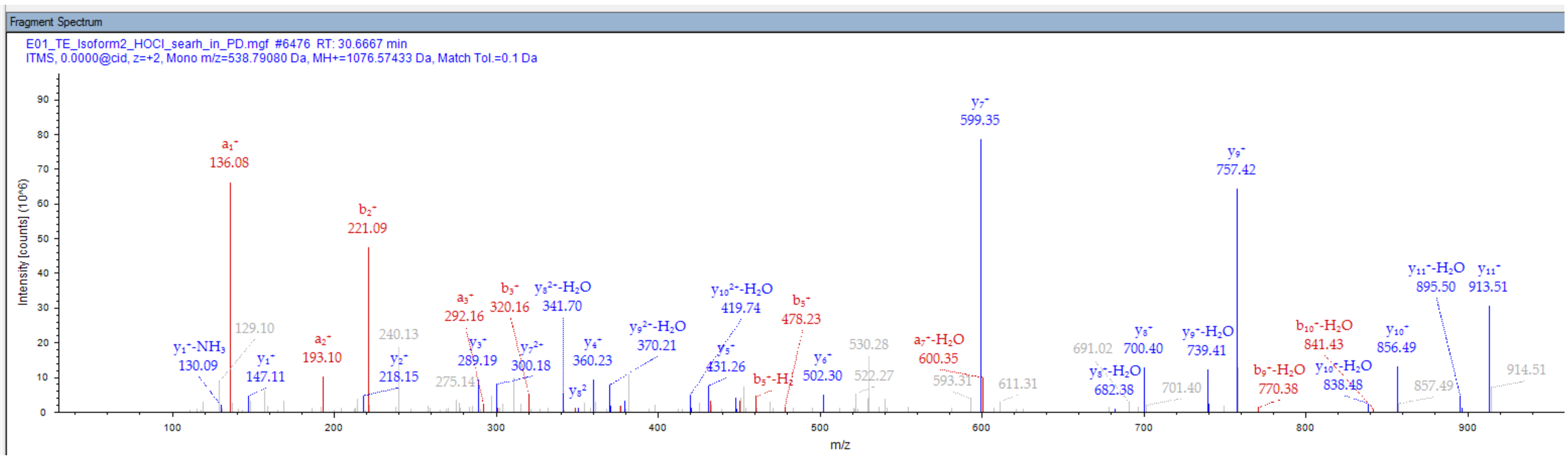

Ion Series | Neutral Losses | Precursor Ions | Internal Fragments

#1	a^+	a^{2+}	b^+	b^{2+}	Seq.	y^+	y^{2+}	#2
1	136.07569	68.54148	164.07061	82.53894	Y			12
2	193.09715	97.05222	221.09207	111.04967	G	913.51016	457.25872	11
3	292.16557	146.58642	320.16048	160.58388	V	856.48869	428.74798	10
4	349.18703	175.09715	377.18195	189.09461	G	757.42028	379.21378	9
5	450.23471	225.62099	478.22962	239.61845	T	700.39882	350.70305	8
6	547.28747	274.14738	575.28239	288.14483	P	599.35114	300.17921	7
7	618.32459	309.66593	646.31950	323.66339	A	502.29837	251.65282	6
8	689.36170	345.18449	717.35662	359.18195	A	431.26126	216.13427	5
9	760.39882	380.70305	788.39373	394.70050	A	360.22415	180.61571	4
10	831.43593	416.22160	859.43084	430.21906	A	289.18703	145.09715	3
11	902.47304	451.74016	930.46796	465.73762	A	218.14992	109.57860	2
12					K	147.11280	74.06004	1

YGVGTPAAAAK, Y1-Chlorination (33.96103 Da)

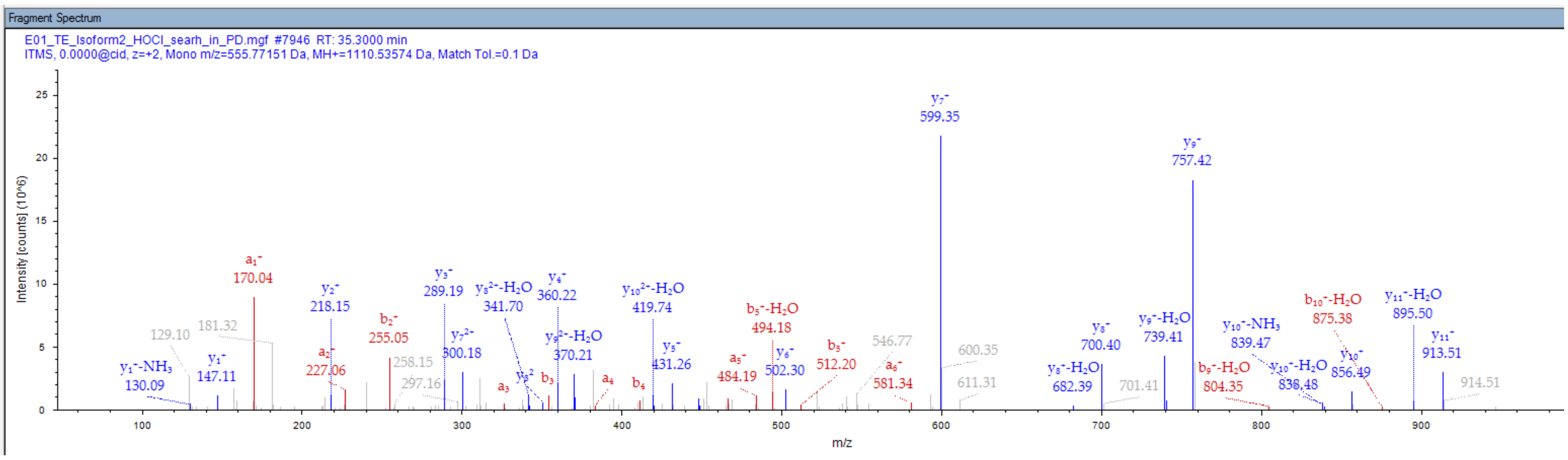

Ion Series	Neutral Losses	Precursor Ions	Internal Fragments

#1	a+	a2+	b+	b2+	Seq.	y+	y2+	#2
1	170.03672	85.52200	198.03163	99.51945	Y-Chlorinat...			12
2	227.05818	114.03273	255.05310	128.03019	G	913.51016	457.25872	11
3	326.12660	163.56694	354.12151	177.56439	V	856.48869	428.74798	10
4	383.14806	192.07767	411.14297	206.07513	G	757.42028	379.21378	9
5	484.19574	242.60151	512.19065	256.59896	T	700.39882	350.70305	8
6	581.24850	291.12789	609.24342	305.12535	P	599.35114	300.17921	7
7	652.28562	326.64645	680.28053	340.64390	A	502.29837	251.65282	6
8	723.32273	362.16500	751.31764	376.16246	A	431.26126	216.13427	5
9	794.35984	397.68356	822.35476	411.68102	A	360.22415	180.61571	4
10	865.39696	433.20212	893.39187	447.19957	A	289.18703	145.09715	3
11	936.43407	468.72067	964.42899	482.71813	A	218.14992	109.57860	2
12					K	147.11280	74.06004	1

YGVGTPAAAAAK, Y1-dichlorination (67.92206 Da)

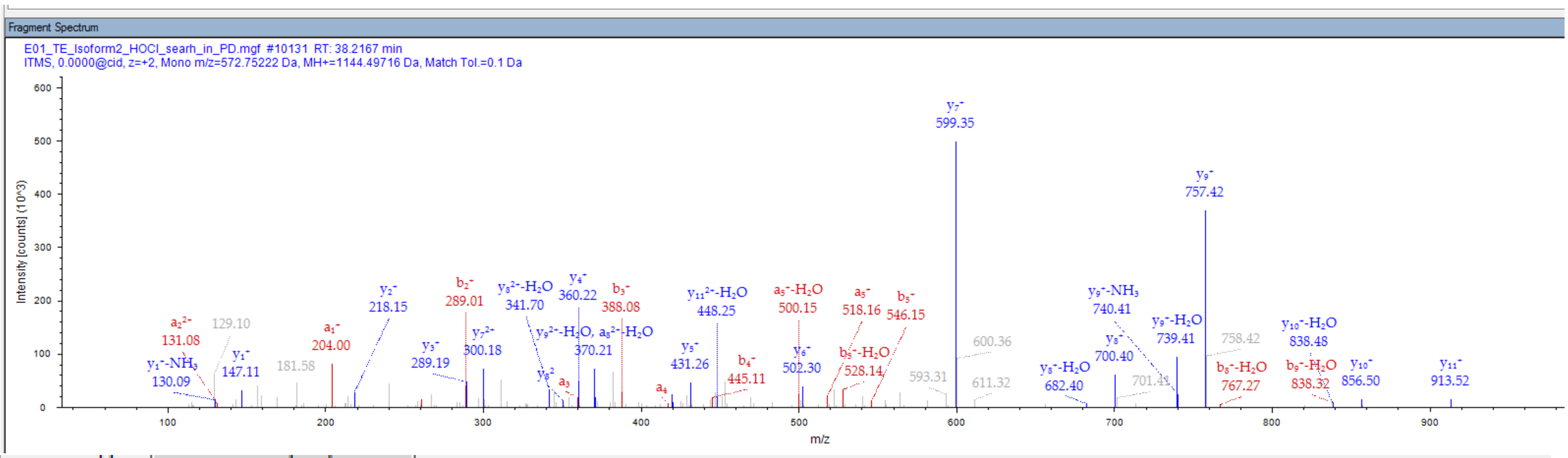

Ion Series | Neutral Losses | Precursor Ions | Internal Fragments

#1	a⁺	a²⁺	b⁺	b²⁺	Seq.	y⁺	y²⁺	#2
1	203.99775	102.50251	231.99266	116.49997	Y-dichlorin...			12
2	261.01921	131.01324	289.01412	145.01070	G	913.51016	457.25872	11
3	360.08762	180.54745	388.08254	194.54491	V	856.48869	428.74798	10
4	417.10909	209.05818	445.10400	223.05564	G	757.42028	379.21378	9
5	518.15677	259.58202	546.15168	273.57948	T	700.39882	350.70305	8
6	615.20953	308.10840	643.20444	322.10586	P	599.35114	300.17921	7
7	686.24664	343.62696	714.24156	357.62442	A	502.29837	251.65282	6
8	757.28376	379.14552	785.27867	393.14297	A	431.26126	216.13427	5
9	828.32087	414.66407	856.31578	428.66153	A	360.22415	180.61571	4
10	899.35798	450.18263	927.35290	464.18009	A	289.18703	145.09715	3
11	970.39510	485.70119	998.39001	499.69864	A	218.14992	109.57860	2
12					K	147.11280	74.06004	1

YGAAVPGVLGGLGALGGVGIPGGVVGA GPAAAAAAK

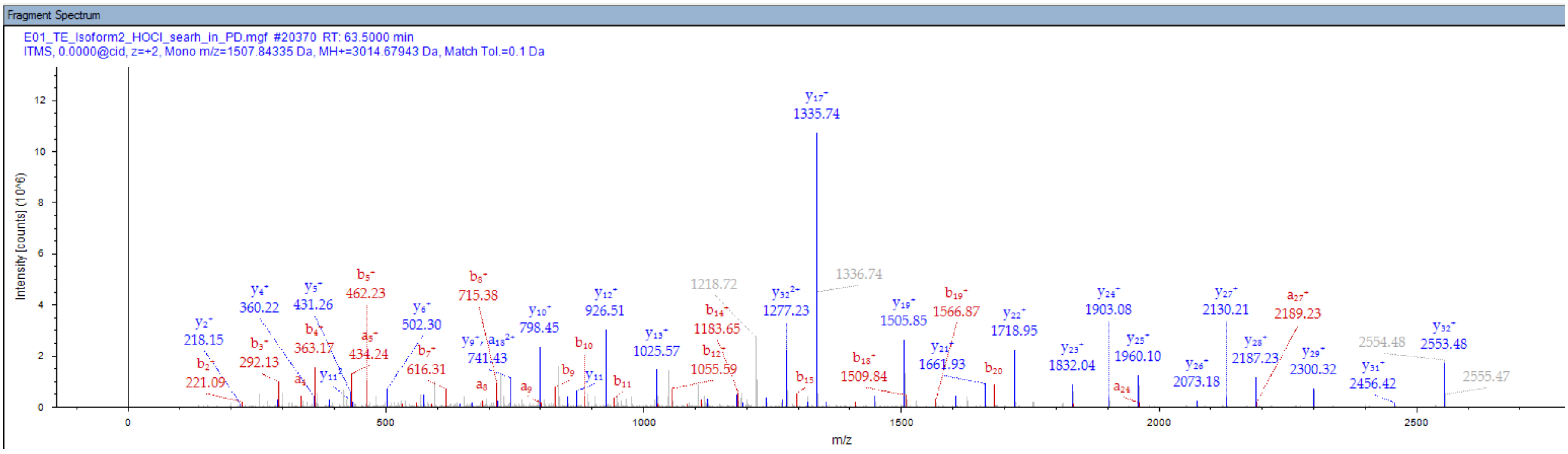

Ion Series | Neutral Losses | Precursor Ions | Internal Fragments

#1	a⁺	a²⁺	b⁺	b²⁺	Seq.	y⁺	y²⁺	#2
1	136.07569	68.54148	164.07061	82.53894	Y			37
2	193.09715	97.05222	221.09207	111.04967	G	2851.61524	1426.31126	36
3	264.13427	132.57077	292.12918	146.56823	A	2794.59377	1397.80052	35
4	335.17138	168.08933	363.16630	182.08679	A	2723.55666	1362.28197	34
5	434.23980	217.62354	462.23471	231.62099	V	2652.51955	1326.76341	33
6	531.29256	266.14992	559.28747	280.14738	P	2553.45113	1277.22920	32
7	588.31402	294.66065	616.30894	308.65811	G	2456.39837	1228.70282	31
8	687.38244	344.19486	715.37735	358.19231	V	2399.37690	1200.19209	30
9	800.46650	400.73689	828.46142	414.73435	L	2300.30849	1150.65788	29
10	857.48796	429.24762	885.48288	443.24508	G	2187.22443	1094.11585	28
11	914.50943	457.75835	942.50434	471.75581	G	2130.20296	1065.60512	27
12	1027.59349	514.30038	1055.58841	528.29784	L	2073.18150	1037.09439	26
13	1084.61496	542.81112	1112.60987	556.80857	G	1960.09743	980.55236	25
14	1155.65207	578.32967	1183.64698	592.32713	A	1903.07597	952.04162	24
15	1268.73613	634.87171	1296.73105	648.86916	L	1832.03886	916.52307	23
16	1325.75760	663.38244	1353.75251	677.37989	G	1718.95479	859.98103	22
17	1382.77906	691.89317	1410.77398	705.89063	G	1661.93333	831.47030	21
18	1481.84747	741.42738	1509.84239	755.42483	V	1604.91187	802.95957	20
19	1538.86894	769.93811	1566.86385	783.93556	G	1505.84345	753.42536	19
20	1651.95300	826.48014	1679.94792	840.47760	I	1448.82199	724.91463	18
21	1749.00577	875.00652	1777.00068	889.00398	P	1335.73792	668.37260	17
22	1806.02723	903.51725	1834.02214	917.51471	G	1238.68516	619.84622	16
23	1863.04869	932.02799	1891.04361	946.02544	G	1181.66370	591.33549	15
24	1962.11711	981.56219	1990.11202	995.55965	V	1124.64223	562.82475	14
25	2061.18552	1031.09640	2089.18044	1045.09386	V	1025.57382	513.29055	13
26	2118.20699	1059.60713	2146.20190	1073.60459	G	926.50541	463.75634	12
27	2189.24410	1095.12569	2217.23901	1109.12315	A	869.48394	435.24561	11
28	2246.26556	1123.63642	2274.26048	1137.63388	G	798.44683	399.72705	10
29	2343.31833	1172.16280	2371.31324	1186.16026	P	741.42536	371.21632	9
30	2414.35544	1207.68136	2442.35035	1221.67882	A	644.37260	322.68994	8
31	2485.39255	1243.19992	2513.38747	1257.19737	A	573.33549	287.17138	7
32	2556.42967	1278.71847	2584.42458	1292.71593	A	502.29837	251.65282	6
33	2627.46678	1314.23703	2655.46170	1328.23449	A	431.26126	216.13427	5
34	2698.50390	1349.75559	2726.49881	1363.75304	A	360.22415	180.61571	4
35	2769.54101	1385.27414	2797.53592	1399.27160	A	289.18703	145.09715	3
36	2840.57812	1420.79270	2868.57304	1434.79016	A	218.14992	109.57860	2
37					K	147.11280	74.06004	1

YGAAVPGVLGGLGALGGVGIPGGVVGA GPAAAAAAK, Y1-Chlorination (33.96103 Da)

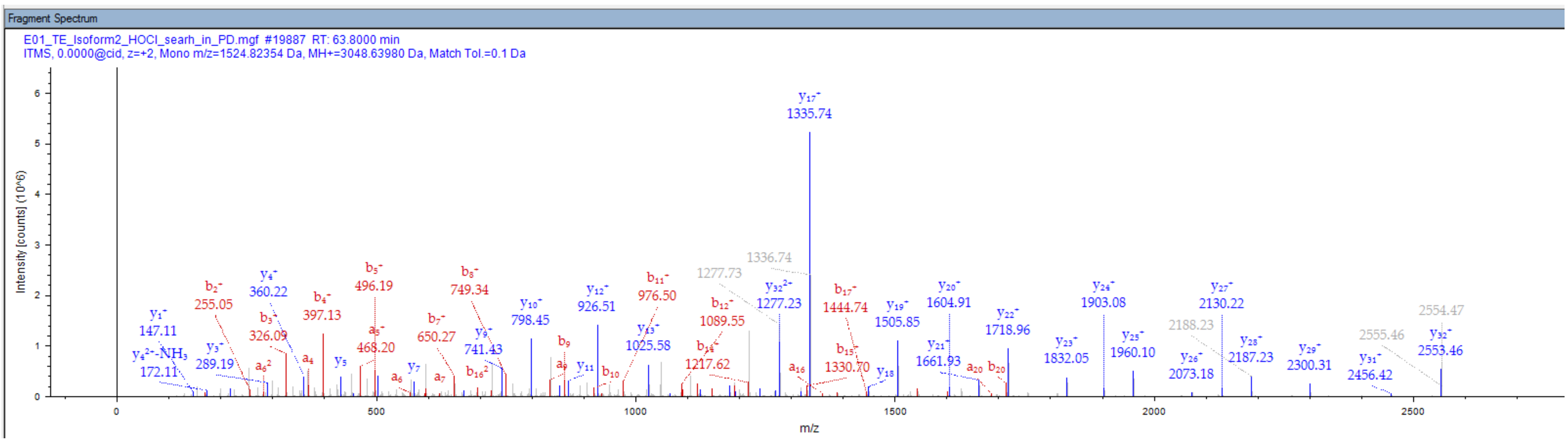

Ion Series | Neutral Losses | Precursor Ions | Internal Fragments

#1	a⁺	a²⁺	b⁺	b²⁺	Seq.	y⁺	y²⁺	#2
1	170.03672	85.52200	198.03163	99.51945	Y-Chlorinat...			37
2	227.05818	114.03273	255.05310	128.03019	G	2851.61524	1426.31126	36
3	298.09530	149.55129	326.09021	163.54874	A	2794.59377	1397.80052	35
4	369.13241	185.06984	397.12732	199.06730	A	2723.55666	1362.28197	34
5	468.20082	234.60405	496.19574	248.60151	V	2652.51955	1326.76341	33
6	565.25359	283.13043	593.24850	297.12789	P	2553.45113	1277.22920	32
7	622.27505	311.64116	650.26997	325.63862	G	2456.39837	1228.70282	31
8	721.34346	361.17537	749.33838	375.17283	V	2399.37690	1200.19209	30
9	834.42753	417.71740	862.42244	431.71486	L	2300.30849	1150.65788	29
10	891.44899	446.22813	919.44391	460.22559	G	2187.22443	1094.11585	28
11	948.47046	474.73887	976.46537	488.73632	G	2130.20296	1065.60512	27
12	1061.55452	531.28090	1089.54943	545.27836	L	2073.18150	1037.09439	26
13	1118.57598	559.79163	1146.57090	573.78909	G	1960.09743	980.55236	25
14	1189.61310	595.31019	1217.60801	609.30764	A	1903.07597	952.04162	24
15	1302.69716	651.85222	1330.69208	665.84968	L	1832.03886	916.52307	23
16	1359.71863	680.36295	1387.71354	694.36041	G	1718.95479	859.98103	22
17	1416.74009	708.87368	1444.73500	722.87114	G	1661.93333	831.47030	21
18	1515.80850	758.40789	1543.80342	772.40535	V	1604.91187	802.95957	20
19	1572.82997	786.91862	1600.82488	800.91608	G	1505.84345	753.42536	19
20	1685.91403	843.46065	1713.90895	857.45811	I	1448.82199	724.91463	18
21	1782.96679	891.98704	1810.96171	905.98449	P	1335.73792	668.37260	17
22	1839.98826	920.49777	1867.98317	934.49522	G	1238.68516	619.84622	16
23	1897.00972	949.00850	1925.00464	963.00596	G	1181.66370	591.33549	15
24	1996.07814	998.54271	2024.07305	1012.54016	V	1124.64223	562.82475	14
25	2095.14655	1048.07691	2123.14146	1062.07437	V	1025.57382	513.29055	13
26	2152.16801	1076.58764	2180.16293	1090.58510	G	926.50541	463.75634	12
27	2223.20513	1112.10620	2251.20004	1126.10366	A	869.48394	435.24561	11
28	2280.22659	1140.61693	2308.22151	1154.61439	G	798.44683	399.72705	10
29	2377.27935	1189.14332	2405.27427	1203.14077	P	741.42536	371.21632	9
30	2448.31647	1224.66187	2476.31138	1238.65933	A	644.37260	322.68994	8
31	2519.35358	1260.18043	2547.34850	1274.17789	A	573.33549	287.17138	7
32	2590.39070	1295.69899	2618.38561	1309.69644	A	502.29837	251.65282	6
33	2661.42781	1331.21754	2689.42272	1345.21500	A	431.26126	216.13427	5
34	2732.46492	1366.73610	2760.45984	1380.73356	A	360.22415	180.61571	4
35	2803.50204	1402.25466	2831.49695	1416.25211	A	289.18703	145.09715	3
36	2874.53915	1437.77321	2902.53407	1451.77067	A	218.14992	109.57860	2
37					K	147.11280	74.06004	1

YGAAVPGVLGGLGALGGVGIPGGVVGA GPAAAAAAAK, Y1-dichlorination (67.92206 Da)

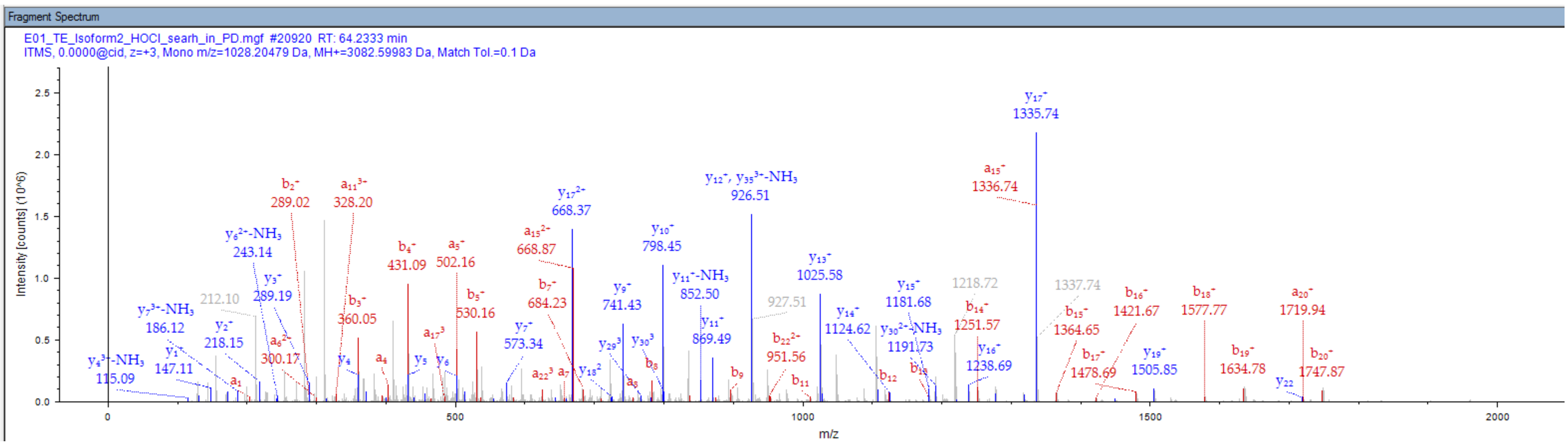

#1	a⁺	a²⁺	a³⁺	b⁺	b²⁺	b³⁺	Seq.	y⁺	y²⁺	y³⁺	#2
1	203.99775	102.50251	68.67077	231.99266	116.49997	78.00240	Y-dichlorin...				37
2	261.01921	131.01324	87.67792	289.01412	145.01070	97.00956	G	2851.61524	1426.31126	951.20993	36
3	332.05632	166.53180	111.35696	360.05124	180.52926	120.68860	A	2794.59377	1397.80052	932.20278	35
4	403.09344	202.05036	135.03600	431.08835	216.04781	144.36763	A	2723.55666	1362.28197	908.52374	34
5	502.16185	251.58456	168.05880	530.15677	265.58202	177.39044	V	2652.51955	1326.76341	884.84470	33
6	599.21461	300.11095	200.40972	627.20953	314.10840	209.74136	P	2553.45113	1277.22920	851.82189	32
7	656.23608	328.62168	219.41688	684.23099	342.61913	228.74852	G	2456.39837	1228.70282	819.47097	31
8	755.30449	378.15588	252.43968	783.29941	392.15334	261.77132	V	2399.37690	1200.19209	800.46382	30
9	868.38856	434.69792	290.13437	896.38347	448.69537	299.46601	L	2300.30849	1150.65788	767.44101	29
10	925.41002	463.20865	309.14152	953.40493	477.20611	318.47316	G	2187.22443	1094.11585	729.74633	28
11	982.43148	491.71938	328.14868	1010.42640	505.71684	337.48032	G	2130.20296	1065.60512	710.73917	27
12	1095.51555	548.26141	365.84337	1123.51046	562.25887	375.17501	L	2073.18150	1037.09439	691.73202	26
13	1152.53701	576.77214	384.85052	1180.53193	590.76960	394.18216	G	1960.09743	980.55236	654.03733	25
14	1223.57412	612.29070	408.52956	1251.56904	626.28816	417.86120	A	1903.07597	952.04162	635.03017	24
15	1336.65819	668.83273	446.22425	1364.65310	682.83019	455.55589	L	1832.03886	916.52307	611.35114	23
16	1393.67965	697.34346	465.23140	1421.67457	711.34092	474.56304	G	1718.95479	859.98103	573.65645	22
17	1450.70112	725.85420	484.23856	1478.69603	739.85165	493.57019	G	1661.93333	831.47030	554.64929	21
18	1549.76953	775.38840	517.26136	1577.76444	789.38586	526.59300	V	1604.91187	802.95957	535.64214	20
19	1606.79099	803.89914	536.26852	1634.78591	817.89659	545.60015	G	1505.84345	753.42536	502.61934	19
20	1719.87506	860.44117	573.96320	1747.86997	874.43862	583.29484	I	1448.82199	724.91463	483.61218	18
21	1816.92782	908.96755	606.31412	1844.92274	922.96501	615.64576	P	1335.73792	668.37260	445.91749	17
22	1873.94928	937.47828	625.32128	1901.94420	951.47574	634.65292	G	1238.68516	619.84622	413.56657	16
23	1930.97075	965.98901	644.32843	1958.96566	979.98647	653.66007	G	1181.66370	591.33549	394.55942	15
24	2030.03916	1015.52322	677.35124	2058.03408	1029.52068	686.68288	V	1124.64223	562.82475	375.55226	14
25	2129.10758	1065.05743	710.37404	2157.10249	1079.05488	719.70568	V	1025.57382	513.29055	342.52946	13
26	2186.12904	1093.56816	729.38120	2214.12395	1107.56562	738.71284	G	926.50541	463.75634	309.50665	12
27	2257.16615	1129.08672	753.06024	2285.16107	1143.08417	762.39187	A	869.48394	435.24561	290.49950	11
28	2314.18762	1157.59745	772.06739	2342.18253	1171.59490	781.39903	G	798.44683	399.72705	266.82046	10
29	2411.24038	1206.12383	804.41831	2439.23530	1220.12129	813.74995	P	741.42536	371.21632	247.81331	9
30	2482.27750	1241.64239	828.09735	2510.27241	1255.63984	837.42899	A	644.37260	322.68994	215.46238	8
31	2553.31461	1277.16094	851.77639	2581.30952	1291.15840	861.10803	A	573.33549	287.17138	191.78335	7
32	2624.35172	1312.67950	875.45543	2652.34664	1326.67696	884.78706	A	502.29837	251.65282	168.10431	6
33	2695.38884	1348.19806	899.13446	2723.38375	1362.19551	908.46610	A	431.26126	216.13427	144.42527	5
34	2766.42595	1383.71661	922.81350	2794.42086	1397.71407	932.14514	A	360.22415	180.61571	120.74623	4
35	2837.46306	1419.23517	946.49254	2865.45798	1433.23263	955.82418	A	289.18703	145.09715	97.06720	3
36	2908.50018	1454.75373	970.17158	2936.49509	1468.75118	979.50322	A	218.14992	109.57860	73.38816	2
37							K	147.11280	74.06004	49.70912	1

YGAAVPGVLGGLGALGGVGIPGGVVGA GPAAAAAAK

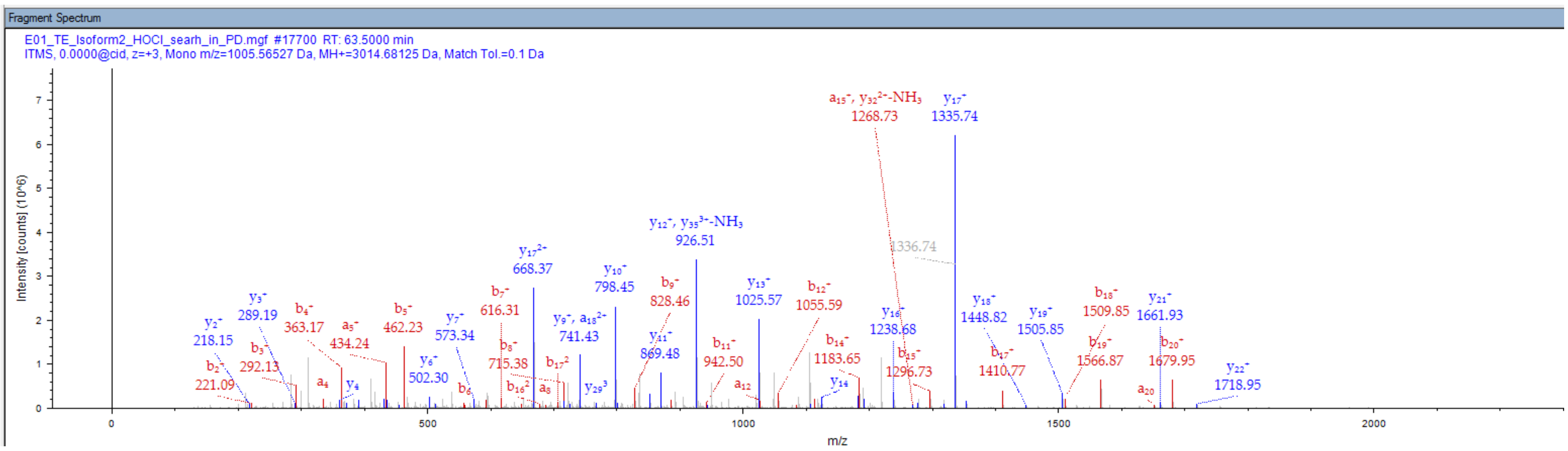

Ion Series | Neutral Losses | Precursor Ions | Internal Fragments

#1	a+	a2+	a3+	b+	b2+	b3+	Seq.	y+	y2+	y3+	#2
1	136.07569	68.54148	46.03008	164.07061	82.53894	55.36172	Y				37
2	193.09715	97.05222	65.03724	221.09207	111.04967	74.36887	G	2851.61524	1426.31126	951.20993	36
3	264.13427	132.57077	88.71627	292.12918	146.56823	98.04791	A	2794.59377	1397.80052	932.20278	35
4	335.17138	168.08933	112.39531	363.16630	182.08679	121.72695	A	2723.55666	1362.28197	908.52374	34
5	434.23980	217.62354	145.41812	462.23471	231.62099	154.74975	V	2652.51955	1326.76341	884.84470	33
6	531.29256	266.14992	177.76904	559.28747	280.14738	187.10068	P	2553.45113	1277.22920	851.82189	32
7	588.31402	294.66065	196.77619	616.30894	308.65811	206.10783	G	2456.39837	1228.70282	819.47097	31
8	687.38244	344.19486	229.79900	715.37735	358.19231	239.13063	V	2399.37690	1200.19209	800.46382	30
9	800.46650	400.73689	267.49368	828.46142	414.73435	276.82532	L	2300.30849	1150.65788	767.44101	29
10	857.48796	429.24762	286.50084	885.48288	443.24508	295.83248	G	2187.22443	1094.11585	729.74633	28
11	914.50943	457.75835	305.50799	942.50434	471.75581	314.83963	G	2130.20296	1065.60512	710.73917	27
12	1027.59349	514.30038	343.20268	1055.58841	528.29784	352.53432	L	2073.18150	1037.09439	691.73202	26
13	1084.61496	542.81112	362.20984	1112.60987	556.80857	371.54147	G	1960.09743	980.55236	654.03733	25
14	1155.65207	578.32967	385.88887	1183.64698	592.32713	395.22051	A	1903.07597	952.04162	635.03017	24
15	1268.73613	634.87171	423.58356	1296.73105	648.86916	432.91520	L	1832.03886	916.52307	611.35114	23
16	1325.75760	663.38244	442.59072	1353.75251	677.37989	451.92236	G	1718.95479	859.98103	573.65645	22
17	1382.77906	691.89317	461.59787	1410.77398	705.89063	470.92951	G	1661.93333	831.47030	554.64929	21
18	1481.84747	741.42738	494.62068	1509.84239	755.42483	503.95231	V	1604.91187	802.95957	535.64214	20
19	1538.86894	769.93811	513.62783	1566.86385	783.93556	522.95947	G	1505.84345	753.42536	502.61934	19
20	1651.95300	826.48014	551.32252	1679.94792	840.47760	560.65416	I	1448.82199	724.91463	483.61218	18
21	1749.00577	875.00652	583.67344	1777.00068	889.00398	593.00508	P	1335.73792	668.37260	445.91749	17
22	1806.02723	903.51725	602.68059	1834.02214	917.51471	612.01223	G	1238.68516	619.84622	413.56657	16
23	1863.04869	932.02799	621.68775	1891.04361	946.02544	631.01939	G	1181.66370	591.33549	394.55942	15
24	1962.11711	981.56219	654.71055	1990.11202	995.55965	664.04219	V	1124.64223	562.82475	375.55226	14
25	2061.18552	1031.09640	687.73336	2089.18044	1045.09386	697.06500	V	1025.57382	513.29055	342.52946	13
26	2118.20699	1059.60713	706.74051	2146.20190	1073.60459	716.07215	G	926.50541	463.75634	309.50665	12
27	2189.24410	1095.12569	730.41955	2217.23901	1109.12315	739.75119	A	869.48394	435.24561	290.49950	11
28	2246.26556	1123.63642	749.42671	2274.26048	1137.63388	758.75834	G	798.44683	399.72705	266.82046	10
29	2343.31833	1172.16280	781.77763	2371.31324	1186.16026	791.10926	P	741.42536	371.21632	247.81331	9
30	2414.35544	1207.68136	805.45666	2442.35035	1221.67882	814.78830	A	644.37260	322.68994	215.46238	8
31	2485.39255	1243.19992	829.13570	2513.38747	1257.19737	838.46734	A	573.33549	287.17138	191.78335	7
32	2556.42967	1278.71847	852.81474	2584.42458	1292.71593	862.14638	A	502.29837	251.65282	168.10431	6
33	2627.46678	1314.23703	876.49378	2655.46170	1328.23449	885.82542	A	431.26126	216.13427	144.42527	5
34	2698.50390	1349.75559	900.17282	2726.49881	1363.75304	909.50445	A	360.22415	180.61571	120.74623	4
35	2769.54101	1385.27414	923.85185	2797.53592	1399.27160	933.18349	A	289.18703	145.09715	97.06720	3
36	2840.57812	1420.79270	947.53089	2868.57304	1434.79016	956.86253	A	218.14992	109.57860	73.38816	2
37							K	147.11280	74.06004	49.70912	1

YGAAGLGGVLGGAGQFPLGGVAARPGFGLSPIFPGGACLGKACGR, C43-Oxidation (15.99492 Da), Y1-Chlorination (33.96103 Da)

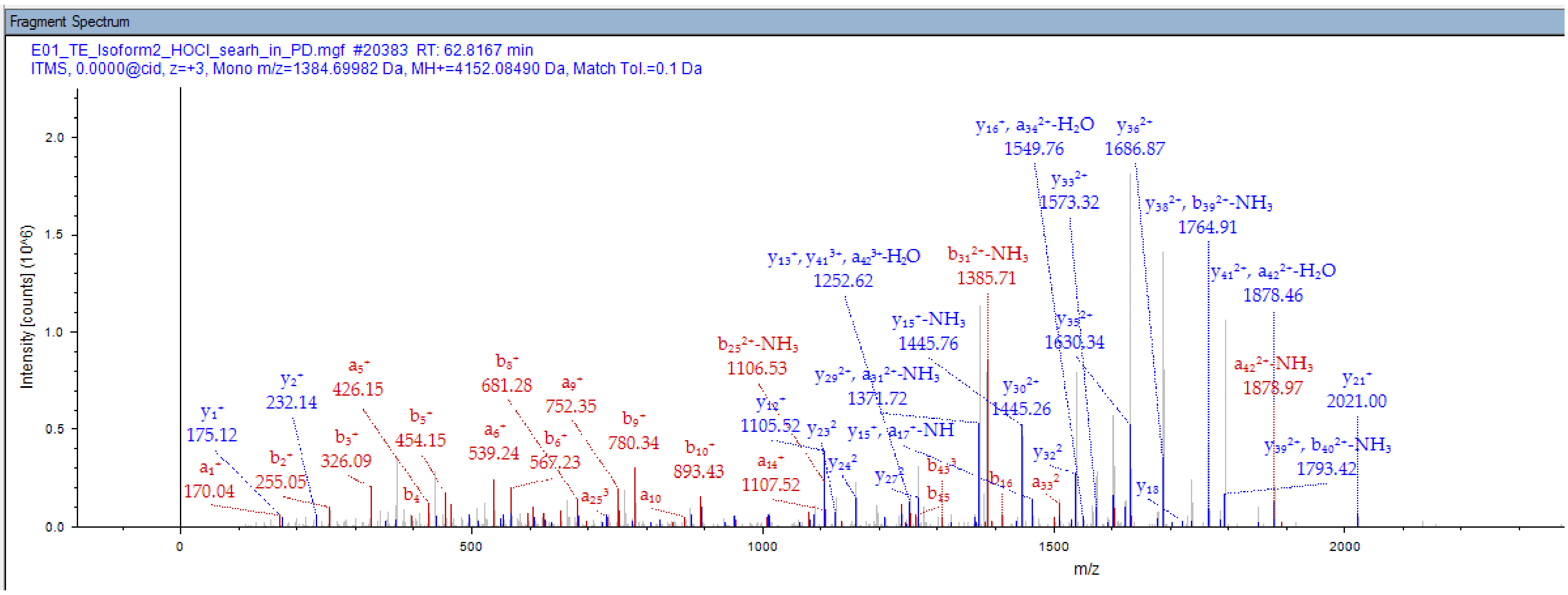

Ion Series | Neutral Losses | Precursor Ions | Internal Fragments

#1	a^+	a^{2+}	a^{3+}	b^+	b^{2+}	b^{3+}	Seq.	y^+	y^{2+}	y^{3+}	#2
1	170.03672	85.52200	57.35042	198.03163	99.51945	66.68206	Y-Chlorinat...				45
2	227.05818	114.03273	76.35758	255.05310	128.03019	85.68922	G	3955.05818	1978.03273	1319.02424	44
3	298.09530	149.55129	100.03662	326.09021	163.54874	109.36825	A	3898.03671	1949.52199	1300.01709	43
4	369.13241	185.06984	123.71565	397.12732	199.06730	133.04729	A	3826.99960	1914.00344	1276.33805	42
5	426.15387	213.58058	142.72281	454.14879	227.57803	152.05445	G	3755.96248	1878.48488	1252.65901	41
6	539.23794	270.12261	180.41750	567.23285	284.12006	189.74914	L	3698.94102	1849.97415	1233.65186	40
7	596.25940	298.63334	199.42465	624.25432	312.63080	208.75629	G	3585.85696	1793.43212	1195.95717	39
8	653.28086	327.14407	218.43181	681.27578	341.14153	227.76344	G	3528.83549	1764.92138	1176.95002	38
9	752.34928	376.67828	251.45461	780.34419	390.67573	260.78625	V	3471.81403	1736.41065	1157.94286	37
10	865.43334	433.22031	289.14930	893.42826	447.21777	298.48094	L	3372.74562	1686.87645	1124.92006	36
11	922.45481	461.73104	308.15645	950.44972	475.72850	317.48809	G	3259.66155	1630.33441	1087.22537	35
12	979.47627	490.24177	327.16361	1007.47118	504.23923	336.49525	G	3202.64009	1601.82368	1068.21821	34
13	1050.51338	525.76033	350.84265	1078.50830	539.75779	360.17428	A	3145.61862	1573.31295	1049.21106	33
14	1107.53485	554.27106	369.84980	1135.52976	568.26852	379.18144	G	3074.58151	1537.79439	1025.53202	32
15	1235.59342	618.30035	412.53599	1263.58834	632.29781	421.86763	Q	3017.56005	1509.28366	1006.52487	31
16	1382.66184	691.83456	461.55880	1410.65675	705.83201	470.89044	F	2889.50147	1445.25437	963.83867	30
17	1479.71460	740.36094	493.90972	1507.70952	754.35840	503.24136	P	2742.43306	1371.72017	914.81587	29
18	1592.79867	796.90297	531.60441	1620.79358	810.90043	540.93604	L	2645.38029	1323.19378	882.46495	28
19	1649.82013	825.41370	550.61156	1677.81504	839.41116	559.94320	G	2532.29623	1266.65175	844.77026	27
20	1706.84159	853.92444	569.61872	1734.83651	867.92189	578.95035	G	2475.27476	1238.14102	825.76311	26
21	1805.91001	903.45864	602.64152	1833.90492	917.45610	611.97316	V	2418.25330	1209.63029	806.75595	25
22	1876.94712	938.97720	626.32056	1904.94204	952.97466	635.65220	A	2319.18489	1160.09608	773.73315	24
23	1947.98424	974.49576	649.99960	1975.97915	988.49321	659.33123	A	2248.14777	1124.57752	750.05411	23
24	2104.08535	1052.54631	702.03330	2132.08026	1066.54377	711.36494	R	2177.11066	1089.05897	726.37507	22
25	2201.13811	1101.07269	734.38422	2229.13302	1115.07015	743.71586	P	2021.00955	1011.00841	674.34137	21
26	2258.15957	1129.58343	753.39138	2286.15449	1143.58088	762.72301	G	1923.95678	962.48203	641.99045	20
27	2405.22799	1203.11763	802.41418	2433.22290	1217.11509	811.74582	F	1866.93532	933.97130	622.98329	19
28	2462.24945	1231.62836	821.42133	2490.24437	1245.62582	830.75297	G	1719.86691	860.43709	573.96049	18
29	2575.33352	1288.17040	859.11602	2603.32843	1302.16785	868.44766	L	1662.84544	831.92636	554.95333	17
30	2662.36554	1331.68641	888.12670	2690.36046	1345.68387	897.45834	S	1549.76138	775.38433	517.25864	16
31	2759.41831	1380.21279	920.47762	2787.41322	1394.21025	929.80926	P	1462.72935	731.86831	488.24797	15
32	2872.50237	1436.75482	958.17231	2900.49729	1450.75228	967.50395	I	1365.67659	683.34193	455.89705	14
33	3019.57079	1510.28903	1007.19511	3047.56570	1524.28649	1016.52675	F	1252.59252	626.79990	418.20236	13
34	3116.62355	1558.81541	1039.54603	3144.61846	1572.81287	1048.87767	P	1105.52411	553.26569	369.17955	12
35	3173.64501	1587.32614	1058.55319	3201.63993	1601.32360	1067.88483	G	1008.47135	504.73931	336.82863	11
36	3230.66648	1615.83688	1077.56034	3258.66139	1629.83433	1086.89198	G	951.44988	476.22858	317.82148	10
37	3301.70359	1651.35543	1101.23938	3329.69850	1665.35289	1110.57102	A	894.42842	447.71785	298.81432	9
38	3404.71277	1702.86003	1135.57578	3432.70769	1716.85748	1144.90741	C	823.39130	412.19929	275.13529	8
39	3517.79684	1759.40206	1173.27046	3545.79175	1773.39951	1182.60210	L	720.38212	360.69470	240.79889	7
40	3574.81830	1787.91279	1192.27762	3602.81322	1801.91025	1201.60926	G	607.29806	304.15267	203.10420	6
41	3702.91326	1851.96027	1234.97594	3730.90818	1865.95773	1244.30758	K	550.27659	275.64193	184.09705	5
42	3773.95038	1887.47883	1258.65498	3801.94529	1901.47628	1267.98662	A	422.18163	211.59445	141.39873	4
43	3892.95448	1946.98088	1298.32301	3920.94939	1960.97833	1307.65465	C-Oxidation	351.14452	176.07590	117.71969	3
44	3949.97594	1975.49161	1317.33017	3977.97086	1989.48907	1326.66180	G	232.14042	116.57385	78.05166	2
45							R	175.11895	88.06311	59.04450	1

YGAAGLGGVLGGAGQFPLGGVAARPGFGLSPIFPGGACLGKACGR, C38-Oxidation (15.99492 Da), Y1-Chlorination (33.96103 Da)

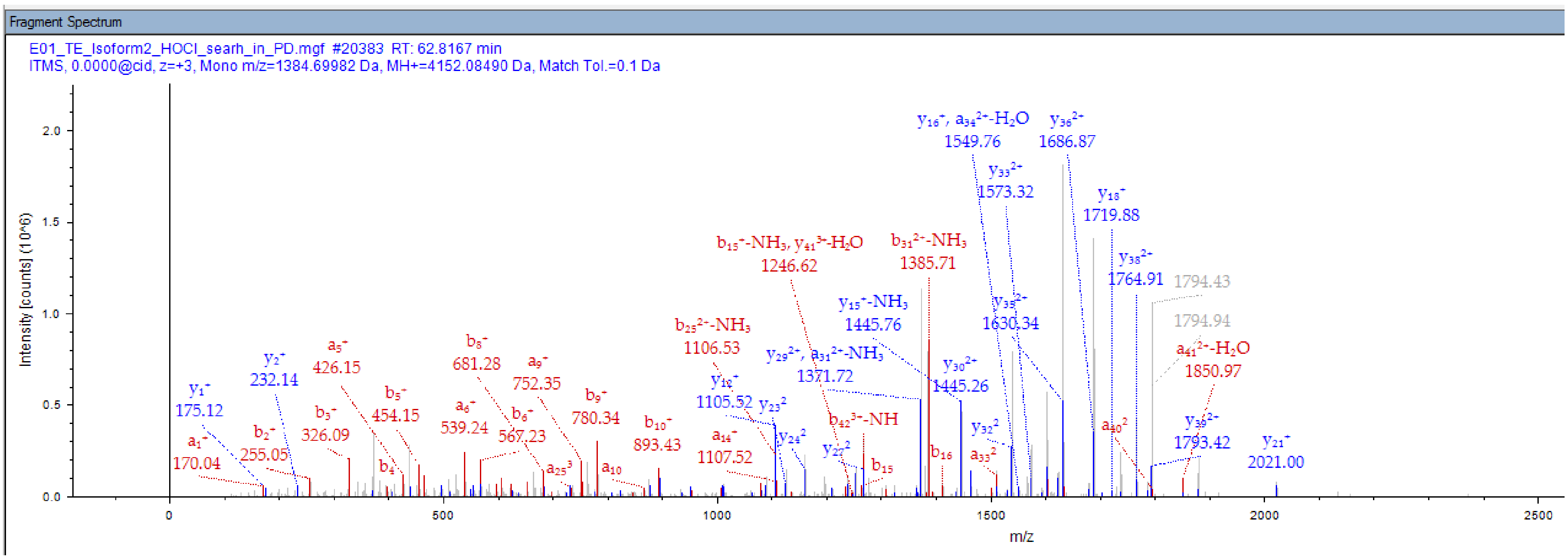

#1	a⁺	a²⁺	a³⁺	b⁺	b²⁺	b³⁺	Seq.	y⁺	y²⁺	y³⁺	#2
1	170.03672	85.52200	57.35042	198.03163	99.51945	66.68206	Y-Chlorinat...				45
2	227.05818	114.03273	76.35758	255.05310	128.03019	85.68922	G	3955.05818	1978.03273	1319.02424	44
3	298.09530	149.55129	100.03662	326.09021	163.54874	109.36825	A	3898.03671	1949.52199	1300.01709	43
4	369.13241	185.06984	123.71565	397.12732	199.06730	133.04729	A	3826.99960	1914.00344	1276.33805	42
5	426.15387	213.58058	142.72281	454.14879	227.57803	152.05445	G	3755.96248	1878.48488	1252.65901	41
6	539.23794	270.12261	180.41750	567.23285	284.12006	189.74914	L	3698.94102	1849.97415	1233.65186	40
7	596.25940	298.63334	199.42465	624.25432	312.63080	208.75629	G	3585.85696	1793.43212	1195.95717	39
8	653.28086	327.14407	218.43181	681.27578	341.14153	227.76344	G	3528.83549	1764.92138	1176.95002	38
9	752.34928	376.67828	251.45461	780.34419	390.67573	260.78625	V	3471.81403	1736.41065	1157.94286	37
10	865.43334	433.22031	289.14930	893.42826	447.21777	298.48094	L	3372.74562	1686.87645	1124.92006	36
11	922.45481	461.73104	308.15645	950.44972	475.72850	317.48809	G	3259.66155	1630.33441	1087.22537	35
12	979.47627	490.24177	327.16361	1007.47118	504.23923	336.49525	G	3202.64009	1601.82368	1068.21821	34
13	1050.51338	525.76033	350.84265	1078.50830	539.75779	360.17428	A	3145.61862	1573.31295	1049.21106	33
14	1107.53485	554.27106	369.84980	1135.52976	568.26852	379.18144	G	3074.58151	1537.79439	1025.53202	32
15	1235.59342	618.30035	412.53599	1263.58834	632.29781	421.86763	Q	3017.56005	1509.28366	1006.52487	31
16	1382.66184	691.83456	461.55880	1410.65675	705.83201	470.89044	F	2889.50147	1445.25437	963.83867	30
17	1479.71460	740.36094	493.90972	1507.70952	754.35840	503.24136	P	2742.43306	1371.72017	914.81587	29
18	1592.79867	796.90297	531.60441	1620.79358	810.90043	540.93604	L	2645.38029	1323.19378	882.46495	28
19	1649.82013	825.41370	550.61156	1677.81504	839.41116	559.94320	G	2532.29623	1266.65175	844.77026	27
20	1706.84159	853.92444	569.61872	1734.83651	867.92189	578.95035	G	2475.27476	1238.14102	825.76311	26
21	1805.91001	903.45864	602.64152	1833.90492	917.45610	611.97316	V	2418.25330	1209.63029	806.75595	25
22	1876.94712	938.97720	626.32056	1904.94204	952.97466	635.65220	A	2319.18489	1160.09608	773.73315	24
23	1947.98424	974.49576	649.99960	1975.97915	988.49321	659.33123	A	2248.14777	1124.57752	750.05411	23
24	2104.08535	1052.54631	702.03330	2132.08026	1066.54377	711.36494	R	2177.11066	1089.05897	726.37507	22
25	2201.13811	1101.07269	734.38422	2229.13302	1115.07015	743.71586	P	2021.00955	1011.00841	674.34137	21
26	2258.15957	1129.58343	753.39138	2286.15449	1143.58088	762.72301	G	1923.95678	962.48203	641.99045	20
27	2405.22799	1203.11763	802.41418	2433.22290	1217.11509	811.74582	F	1866.93532	933.97130	622.98329	19
28	2462.24945	1231.62836	821.42133	2490.24437	1245.62582	830.75297	G	1719.86691	860.43709	573.96049	18
29	2575.33352	1288.17040	859.11602	2603.32843	1302.16785	868.44766	L	1662.84544	831.92636	554.95333	17
30	2662.36554	1331.68641	888.12670	2690.36046	1345.68387	897.45834	S	1549.76138	775.38433	517.25864	16
31	2759.41831	1380.21279	920.47762	2787.41322	1394.21025	929.80926	P	1462.72935	731.86831	488.24797	15
32	2872.50237	1436.75482	958.17231	2900.49729	1450.75228	967.50395	I	1365.67659	683.34193	455.89705	14
33	3019.57079	1510.28903	1007.19511	3047.56570	1524.28649	1016.52675	F	1252.59252	626.79990	418.20236	13
34	3116.62355	1558.81541	1039.54603	3144.61846	1572.81287	1048.87767	P	1105.52411	553.26569	369.17955	12
35	3173.64501	1587.32614	1058.55319	3201.63993	1601.32360	1067.88483	G	1008.47135	504.73931	336.82863	11
36	3230.66648	1615.83688	1077.56034	3258.66139	1629.83433	1086.89198	G	951.44988	476.22858	317.82148	10
37	3301.70359	1651.35543	1101.23938	3329.69850	1665.35289	1110.57102	A	894.42842	447.71785	298.81432	9
38	3420.70769	1710.85748	1140.90741	3448.70260	1724.85494	1150.23905	C-Oxidation	823.39130	412.19929	275.13529	8
39	3533.79175	1767.39951	1178.60210	3561.78667	1781.39697	1187.93374	L	704.38720	352.69724	235.46725	7
40	3590.81322	1795.91025	1197.60926	3618.80813	1809.90770	1206.94089	G	591.30314	296.15521	197.77256	6
41	3718.90818	1859.95773	1240.30758	3746.90309	1873.95519	1249.63922	K	534.28168	267.64448	178.76541	5
42	3789.94529	1895.47629	1263.98662	3817.94021	1909.47374	1273.31825	A	406.18671	203.59700	136.06709	4
43	3892.95448	1946.98088	1298.32301	3920.94939	1960.97833	1307.65465	C	335.14960	168.07844	112.38805	3
44	3949.97594	1975.49161	1317.33017	3977.97086	1989.48907	1326.66180	G	232.14042	116.57385	78.05166	2
45							R	175.11895	88.06311	59.04450	1

YGAAGLGGVLGGAGQFPLGGVAARPGFGLSPIFPGGACLGKACGR, C43-Oxidation (15.99492 Da)

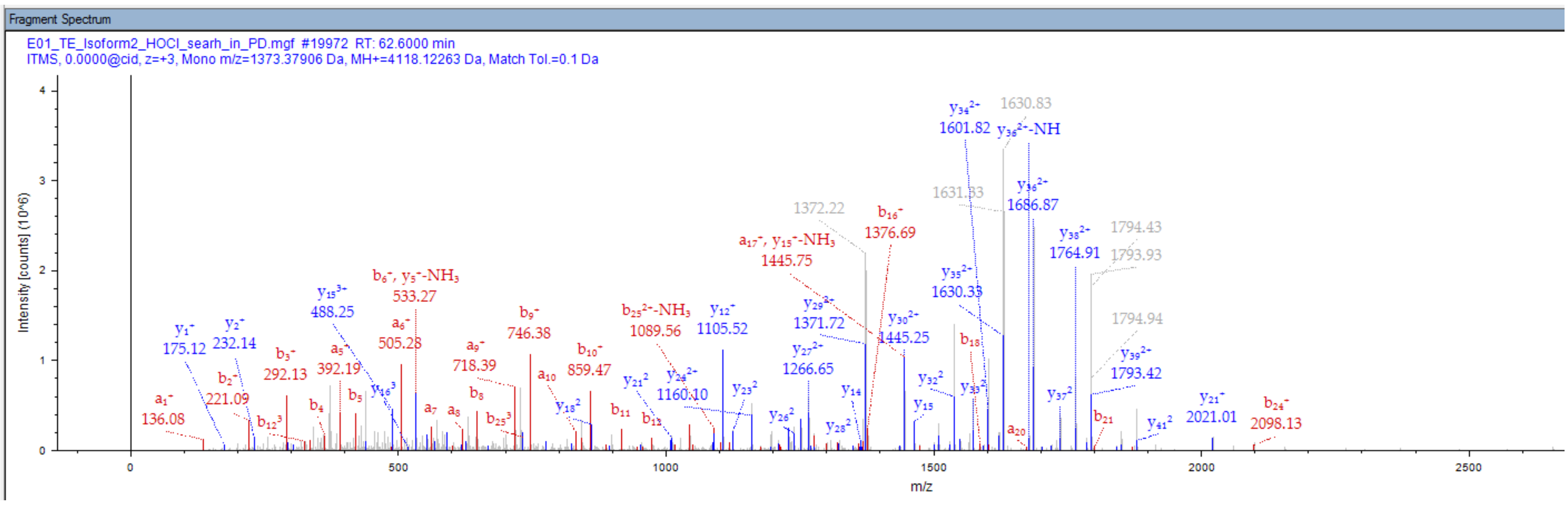

Ion Series | Neutral Losses | Precursor Ions | Internal Fragments

#1	a⁺	a²⁺	a³⁺	b⁺	b²⁺	b³⁺	Seq.	y⁺	y²⁺	y³⁺	#2
1	136.07569	68.54148	46.03008	164.07061	82.53894	55.36172	Y				45
2	193.09715	97.05222	65.03724	221.09207	111.04967	74.36887	G	3955.05818	1978.03273	1319.02424	44
3	264.13427	132.57077	88.71627	292.12918	146.56823	98.04791	A	3898.03671	1949.52199	1300.01709	43
4	335.17138	168.08933	112.39531	363.16630	182.08679	121.72695	A	3826.99960	1914.00344	1276.33805	42
5	392.19285	196.60006	131.40247	420.18776	210.59752	140.73410	G	3755.96248	1878.48488	1252.65901	41
6	505.27691	253.14209	169.09715	533.27182	267.13955	178.42879	L	3698.94102	1849.97415	1233.65186	40
7	562.29837	281.65282	188.10431	590.29329	295.65028	197.43595	G	3585.85696	1793.43212	1195.95717	39
8	619.31984	310.16356	207.11146	647.31475	324.16101	216.44310	G	3528.83549	1764.92138	1176.95002	38
9	718.38825	359.69776	240.13427	746.38317	373.69522	249.46591	V	3471.81403	1736.41065	1157.94286	37
10	831.47231	416.23980	277.82896	859.46723	430.23725	287.16059	L	3372.74562	1686.87645	1124.92006	36
11	888.49378	444.75053	296.83611	916.48869	458.74798	306.16775	G	3259.66155	1630.33441	1087.22537	35
12	945.51524	473.26126	315.84327	973.51016	487.25872	325.17490	G	3202.64009	1601.82368	1068.21821	34
13	1016.55236	508.77982	339.52230	1044.54727	522.77727	348.85394	A	3145.61862	1573.31295	1049.21106	33
14	1073.57382	537.29055	358.52946	1101.56873	551.28801	367.86110	G	3074.58151	1537.79439	1025.53202	32
15	1201.63240	601.31984	401.21565	1229.62731	615.31729	410.54729	Q	3017.56005	1509.28366	1006.52487	31
16	1348.70081	674.85404	450.23845	1376.69573	688.85150	459.57009	F	2889.50147	1445.25437	963.83867	30
17	1445.75357	723.38043	482.58938	1473.74849	737.37788	491.92101	P	2742.43306	1371.72017	914.81587	29
18	1558.83764	779.92246	520.28406	1586.83255	793.91991	529.61570	L	2645.38029	1323.19378	882.46495	28
19	1615.85910	808.43319	539.29122	1643.85402	822.43065	548.62286	G	2532.29623	1266.65175	844.77026	27
20	1672.88057	836.94392	558.29837	1700.87548	850.94138	567.63001	G	2475.27476	1238.14102	825.76311	26
21	1771.94898	886.47813	591.32118	1799.94389	900.47559	600.65282	V	2418.25330	1209.63029	806.75595	25
22	1842.98609	921.99668	615.00022	1870.98101	935.99414	624.33185	A	2319.18489	1160.09608	773.73315	24
23	1914.02321	957.51524	638.67925	1942.01812	971.51270	648.01089	A	2248.14777	1124.57752	750.05411	23
24	2070.12432	1035.56580	690.71296	2098.11923	1049.56325	700.04460	R	2177.11066	1089.05897	726.37507	22
25	2167.17708	1084.09218	723.06388	2195.17200	1098.08964	732.39552	P	2021.00955	1011.00841	674.34137	21
26	2224.19855	1112.60291	742.07103	2252.19346	1126.60037	751.40267	G	1923.95678	962.48203	641.99045	20
27	2371.26696	1186.13712	791.09384	2399.26187	1200.13458	800.42548	F	1866.93532	933.97130	622.98329	19
28	2428.28842	1214.64785	810.10099	2456.28334	1228.64531	819.43263	G	1719.86691	860.43709	573.96049	18
29	2541.37249	1271.18988	847.79568	2569.36740	1285.18734	857.12732	L	1662.84544	831.92636	554.95333	17
30	2628.40452	1314.70590	876.80636	2656.39943	1328.70335	886.13799	S	1549.76138	775.38433	517.25864	16
31	2725.45728	1363.23228	909.15728	2753.45219	1377.22974	918.48892	P	1462.72935	731.86831	488.24797	15
32	2838.54134	1419.77431	946.85197	2866.53626	1433.77177	956.18360	I	1365.67659	683.34193	455.89705	14
33	2985.60976	1493.30852	995.87477	3013.60467	1507.30597	1005.20641	F	1252.59252	626.79990	418.20236	13
34	3082.66252	1541.83490	1028.22569	3110.65744	1555.83236	1037.55733	P	1105.52411	553.26569	369.17955	12
35	3139.68398	1570.34563	1047.23285	3167.67890	1584.34309	1056.56448	G	1008.47135	504.73931	336.82863	11
36	3196.70545	1598.85636	1066.24000	3224.70036	1612.85382	1075.57164	G	951.44988	476.22858	317.82148	10
37	3267.74256	1634.37492	1089.91904	3295.73748	1648.37238	1099.25068	A	894.42842	447.71785	298.81432	9
38	3370.75175	1685.87951	1124.25543	3398.74666	1699.87697	1133.58707	C	823.39130	412.19929	275.13529	8
39	3483.83581	1742.42154	1161.95012	3511.83072	1756.41900	1171.28176	L	720.38212	360.69470	240.79889	7
40	3540.85727	1770.93228	1180.95728	3568.85219	1784.92973	1190.28891	G	607.29806	304.15267	203.10420	6
41	3668.95224	1834.97976	1223.65560	3696.94715	1848.97721	1232.98723	K	550.27659	275.64193	184.09705	5
42	3739.98935	1870.49831	1247.33463	3767.98427	1884.49577	1256.66627	A	422.18163	211.59445	141.39873	4
43	3858.99345	1930.00036	1287.00267	3886.98836	1943.99782	1296.33431	C-Oxidation	351.14452	176.07590	117.71969	3
44	3916.01491	1958.51110	1306.00982	3944.00983	1972.50855	1315.34146	G	232.14042	116.57385	78.05166	2
45							R	175.11895	88.06311	59.04450	1

YGAAGLGGVLGGAGQFPLGGVAARPGFGLSPIFPGGACLGKACGR, C38-Oxidation (15.99492 Da)

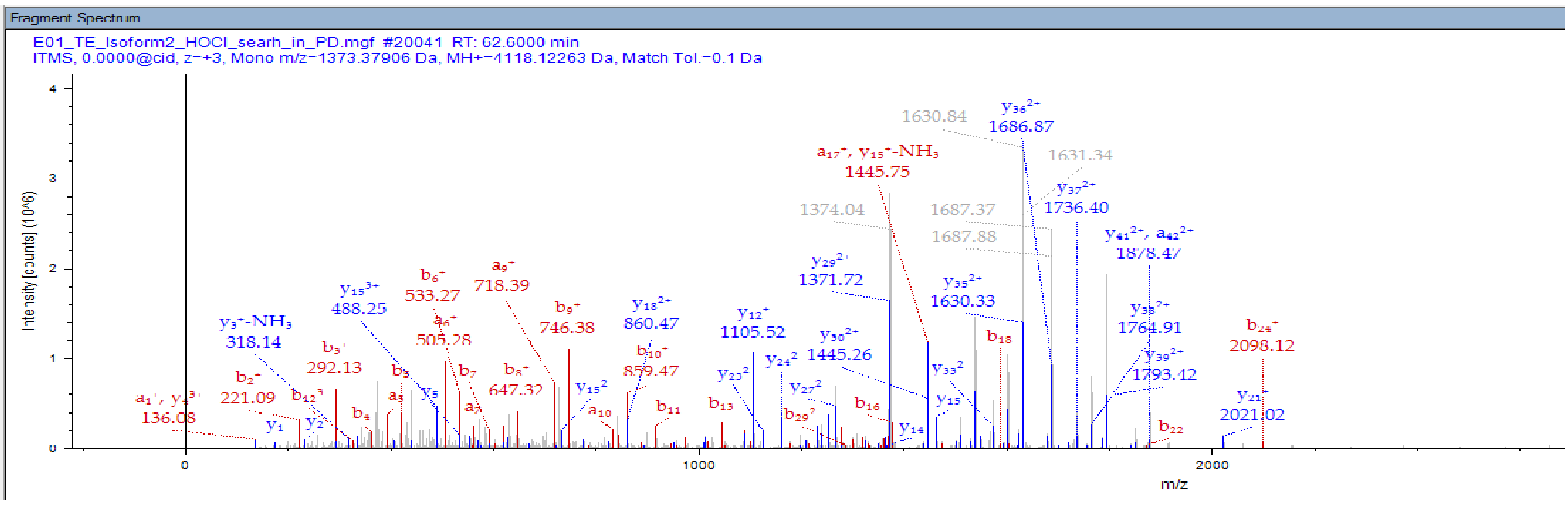

Ion Series | Neutral Losses | Precursor Ions | Internal Fragments

#1	a+	a2+	a3+	b+	b2+	b3+	Seq.	y+	y2+	y3+	#2
1	136.07569	68.54148	46.03008	164.07061	82.53894	55.36172	Y				45
2	193.09715	97.05222	65.03724	221.09207	111.04967	74.36887	G	3955.05818	1978.03273	1319.02424	44
3	264.13427	132.57077	88.71627	292.12918	146.56823	98.04791	A	3898.03671	1949.52199	1300.01709	43
4	335.17138	168.08933	112.39531	363.16630	182.08679	121.72695	A	3826.99960	1914.00344	1276.33805	42
5	392.19285	196.60006	131.40247	420.18776	210.59752	140.73410	G	3755.96248	1878.48488	1252.65901	41
6	505.27691	253.14209	169.09715	533.27182	267.13955	178.42879	L	3698.94102	1849.97415	1233.65186	40
7	562.29837	281.65282	188.10431	590.29329	295.65028	197.43595	G	3585.85696	1793.43212	1195.95717	39
8	619.31984	310.16356	207.11146	647.31475	324.16101	216.44310	G	3528.83549	1764.92138	1176.95002	38
9	718.38825	359.69776	240.13427	746.38317	373.69522	249.46591	V	3471.81403	1736.41065	1157.94286	37
10	831.47231	416.23980	277.82896	859.46723	430.23725	287.16059	L	3372.74562	1686.87645	1124.92006	36
11	888.49378	444.75053	296.83611	916.48869	458.74798	306.16775	G	3259.66155	1630.33441	1087.22537	35
12	945.51524	473.26126	315.84327	973.51016	487.25872	325.17490	G	3202.64009	1601.82368	1068.21821	34
13	1016.55236	508.77982	339.52230	1044.54727	522.77727	348.85394	A	3145.61862	1573.31295	1049.21106	33
14	1073.57382	537.29055	358.52946	1101.56873	551.28801	367.86110	G	3074.58151	1537.79439	1025.53202	32
15	1201.63240	601.31984	401.21565	1229.62731	615.31729	410.54729	Q	3017.56005	1509.28366	1006.52487	31
16	1348.70081	674.85404	450.23845	1376.69573	688.85150	459.57009	F	2889.50147	1445.25437	963.83867	30
17	1445.75357	723.38043	482.58938	1473.74849	737.37788	491.92101	P	2742.43306	1371.72017	914.81587	29
18	1558.83764	779.92246	520.28406	1586.83255	793.91991	529.61570	L	2645.38029	1323.19378	882.46495	28
19	1615.85910	808.43319	539.29122	1643.85402	822.43065	548.62286	G	2532.29623	1266.65175	844.77026	27
20	1672.88057	836.94392	558.29837	1700.87548	850.94138	567.63001	G	2475.27476	1238.14102	825.76311	26
21	1771.94898	886.47813	591.32118	1799.94389	900.47559	600.65282	V	2418.25330	1209.63029	806.75595	25
22	1842.98609	921.99668	615.00022	1870.98101	935.99414	624.33185	A	2319.18489	1160.09608	773.73315	24
23	1914.02321	957.51524	638.67925	1942.01812	971.51270	648.01089	A	2248.14777	1124.57752	750.05411	23
24	2070.12432	1035.56580	690.71296	2098.11923	1049.56325	700.04460	R	2177.11066	1089.05897	726.37507	22
25	2167.17708	1084.09218	723.06388	2195.17200	1098.08964	732.39552	P	2021.00955	1011.00841	674.34137	21
26	2224.19855	1112.60291	742.07103	2252.19346	1126.60037	751.40267	G	1923.95678	962.48203	641.99045	20
27	2371.26696	1186.13712	791.09384	2399.26187	1200.13458	800.42548	F	1866.93532	933.97130	622.98329	19
28	2428.28842	1214.64785	810.10099	2456.28334	1228.64531	819.43263	G	1719.86691	860.43709	573.96049	18
29	2541.37249	1271.18988	847.79568	2569.36740	1285.18734	857.12732	L	1662.84544	831.92636	554.95333	17
30	2628.40452	1314.70590	876.80636	2656.39943	1328.70335	886.13799	S	1549.76138	775.38433	517.25864	16
31	2725.45728	1363.23228	909.15728	2753.45219	1377.22974	918.48892	P	1462.72935	731.86831	488.24797	15
32	2838.54134	1419.77431	946.85197	2866.53626	1433.77177	956.18360	I	1365.67659	683.34193	455.89705	14
33	2985.60976	1493.30852	995.87477	3013.60467	1507.30597	1005.20641	F	1252.59252	626.79990	418.20236	13
34	3082.66252	1541.83490	1028.22569	3110.65744	1555.83236	1037.55733	P	1105.52411	553.26569	369.17955	12
35	3139.68398	1570.34563	1047.23285	3167.67890	1584.34309	1056.56448	G	1008.47135	504.73931	336.82863	11
36	3196.70545	1598.85636	1066.24000	3224.70036	1612.85382	1075.57164	G	951.44988	476.22858	317.82148	10
37	3267.74256	1634.37492	1089.91904	3295.73748	1648.37238	1099.25068	A	894.42842	447.71785	298.81432	9
38	3386.74666	1693.87697	1129.58707	3414.74158	1707.87443	1138.91871	C-Oxidation	823.39130	412.19929	275.13529	8
39	3499.83073	1750.41900	1167.28176	3527.82564	1764.41646	1176.61340	L	704.38720	352.69724	235.46725	7
40	3556.85219	1778.92973	1186.28891	3584.84710	1792.92719	1195.62055	G	591.30314	296.15521	197.77256	6
41	3684.94715	1842.97721	1228.98724	3712.94207	1856.97467	1238.31887	K	534.28168	267.64448	178.76541	5
42	3755.98427	1878.49577	1252.66627	3783.97918	1892.49323	1261.99791	A	406.18671	203.59700	136.06709	4
43	3858.99345	1930.00036	1287.00267	3886.98836	1943.99782	1296.33431	C	335.14960	168.07844	112.38805	3
44	3916.01491	1958.51110	1306.00982	3944.00983	1972.50855	1315.34146	G	232.14042	116.57385	78.05166	2
45							R	175.11895	88.06311	59.04450	1

Tropoelastin Isofrom 2

Treated with MPO

AAAGLGAGIPGLGVGVGVPGLGVGAGV PGLGVGAGVPGFGAVPGALAAAK,

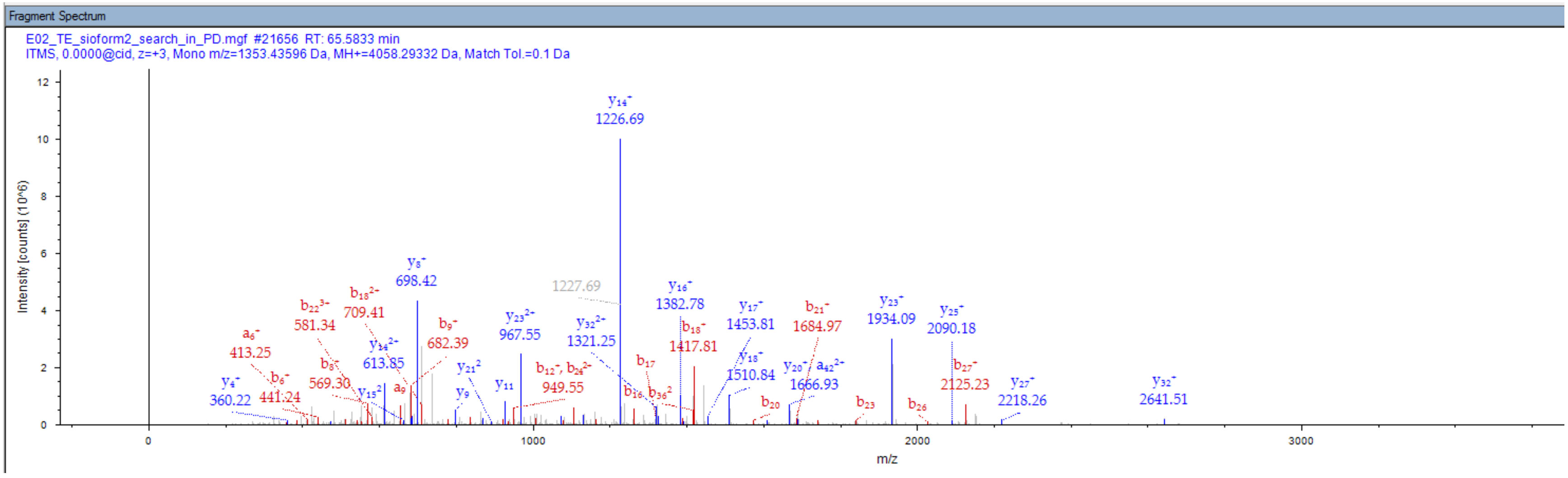

#1	a+	a2+	a3+	b+	b2+	b3+	Seq.	y+	y2+	y3+	#2
1	44.04948	22.52838	15.35468	72.04439	36.52583	24.68631	A				50
2	115.08659	58.04693	39.03371	143.08150	72.04439	48.36535	A	3987.25422	1994.13075	1329.75626	49
3	186.12370	93.56549	62.71275	214.11862	107.56295	72.04439	A	3916.21710	1958.61219	1306.07722	48
4	243.14517	122.07622	81.71991	271.14008	136.07368	91.05154	G	3845.17999	1923.09363	1282.39818	47
5	356.22923	178.61825	119.41459	384.22415	192.61571	128.74623	L	3788.15852	1894.58290	1263.39103	46
6	413.25069	207.12899	138.42175	441.24561	221.12644	147.75339	G	3675.07446	1838.04087	1225.69634	45
7	484.28781	242.64754	162.10079	512.28272	256.64500	171.43243	A	3618.05300	1809.53014	1206.68918	44
8	541.30927	271.15827	181.10794	569.30419	285.15573	190.43958	G	3547.01588	1774.01158	1183.01015	43
9	654.39334	327.70031	218.80263	682.38825	341.69776	228.13427	I	3489.99442	1745.50085	1164.00299	42
10	751.44610	376.22669	251.15355	779.44101	390.22415	260.48519	P	3376.91036	1688.95882	1126.30830	41
11	808.46756	404.73742	270.16071	836.46248	418.73488	279.49234	G	3279.85759	1640.43243	1093.95738	40
12	921.55163	461.27945	307.85539	949.54654	475.27691	317.18703	L	3222.83613	1611.92170	1074.95023	39
13	978.57309	489.79018	326.86255	1006.56801	503.78764	336.19419	G	3109.75206	1555.37967	1037.25554	38
14	1077.64150	539.32439	359.88535	1105.63642	553.32185	369.21699	V	3052.73060	1526.86894	1018.24838	37
15	1134.66297	567.83512	378.89251	1162.65788	581.83258	388.22415	G	2953.66219	1477.33473	985.22558	36
16	1233.73138	617.36933	411.91531	1261.72630	631.36679	421.24695	V	2896.64072	1448.82400	966.21843	35
17	1290.75285	645.88006	430.92247	1318.74776	659.87752	440.25410	G	2797.57231	1399.28979	933.19562	34
18	1389.82126	695.41427	463.94527	1417.81617	709.41173	473.27691	V	2740.55085	1370.77906	914.18847	33
19	1486.87402	743.94065	496.29619	1514.86894	757.93811	505.62783	P	2641.48243	1321.24485	881.16566	32
20	1543.89549	772.45138	515.30335	1571.89040	786.44884	524.63499	G	2544.42967	1272.71847	848.81474	31
21	1656.97955	828.99341	552.99803	1684.97447	842.99087	562.32967	L	2487.40820	1244.20774	829.80759	30
22	1714.00102	857.50415	572.00519	1741.99593	871.50160	581.33683	G	2374.32414	1187.66571	792.11290	29
23	1813.06943	907.03835	605.02799	1841.06434	921.03581	614.35963	V	2317.30268	1159.15498	773.10574	28
24	1870.09089	935.54908	624.03515	1898.08581	949.54654	633.36679	G	2218.23426	1109.62077	740.08294	27
25	1941.12801	971.06764	647.71419	1969.12292	985.06510	657.04582	A	2161.21280	1081.11004	721.07578	26
26	1998.14947	999.57837	666.72134	2026.14438	1013.57583	676.05298	G	2090.17568	1045.59148	697.39675	25
27	2097.21788	1049.11258	699.74415	2125.21280	1063.11004	709.07578	V	2033.15422	1017.08075	678.38959	24
28	2194.27065	1097.63896	732.09507	2222.26556	1111.63642	741.42671	P	1934.08581	967.54654	645.36679	23
29	2251.29211	1126.14969	751.10222	2279.28703	1140.14715	760.43386	G	1837.03304	919.02016	613.01587	22
30	2364.37618	1182.69173	788.79691	2392.37109	1196.68918	798.12855	L	1780.01158	890.50943	594.00871	21
31	2421.39764	1211.20246	807.80406	2449.39255	1225.19992	817.13570	G	1666.92752	833.96740	556.31402	20
32	2520.46605	1260.73666	840.82687	2548.46097	1274.73412	850.15851	V	1609.90605	805.45666	537.30687	19
33	2577.48752	1289.24740	859.83402	2605.48243	1303.24485	869.16566	G	1510.83764	755.92246	504.28406	18
34	2648.52463	1324.76595	883.51306	2676.51955	1338.76341	892.84470	A	1453.81617	727.41173	485.27691	17
35	2705.54609	1353.27669	902.52022	2733.54101	1367.27414	911.85185	G	1382.77906	691.89317	461.59787	16
36	2804.61451	1402.81089	935.54302	2832.60942	1416.80835	944.87466	V	1325.75760	663.38244	442.59072	15
37	2901.66727	1451.33727	967.89394	2929.66219	1465.33473	977.22558	P	1226.68918	613.84823	409.56791	14
38	2958.68874	1479.84801	986.90110	2986.68365	1493.84546	996.23273	G	1129.63642	565.32185	377.21699	13
39	3105.75715	1553.38221	1035.92390	3133.75206	1567.37967	1045.25554	F	1072.61496	536.81112	358.20984	12
40	3162.77861	1581.89294	1054.93106	3190.77353	1595.89040	1064.26269	G	925.54654	463.27691	309.18703	11
41	3233.81573	1617.41150	1078.61009	3261.81064	1631.40896	1087.94173	A	868.52508	434.76618	290.17988	10
42	3332.88414	1666.94571	1111.63290	3360.87906	1680.94317	1120.96454	V	797.48796	399.24762	266.50084	9
43	3429.93690	1715.47209	1143.98382	3457.93182	1729.46955	1153.31546	P	698.41955	349.71341	233.47803	8
44	3486.95837	1743.98282	1162.99097	3514.95328	1757.98028	1172.32261	G	601.36679	301.18703	201.12711	7
45	3557.99548	1779.50138	1186.67001	3585.99040	1793.49884	1196.00165	A	544.34532	272.67630	182.11996	6
46	3671.07955	1836.04341	1224.36470	3699.07446	1850.04087	1233.69634	L	473.30821	237.15774	158.44092	5
47	3742.11666	1871.56197	1248.04374	3770.11157	1885.55943	1257.37538	A	360.22415	180.61571	120.74623	4
48	3813.15377	1907.08053	1271.72278	3841.14869	1921.07798	1281.05441	A	289.18703	145.09715	97.06720	3
49	3884.19089	1942.59908	1295.40181	3912.18580	1956.59654	1304.73345	A	218.14992	109.57860	73.38816	2
50							K	147.11280	74.06004	49.70912	1

AAKYGAAVPGVLGGLGALGGVGIPGGVVGAGPAAAAAAAK, Y4-Chlorination (33.

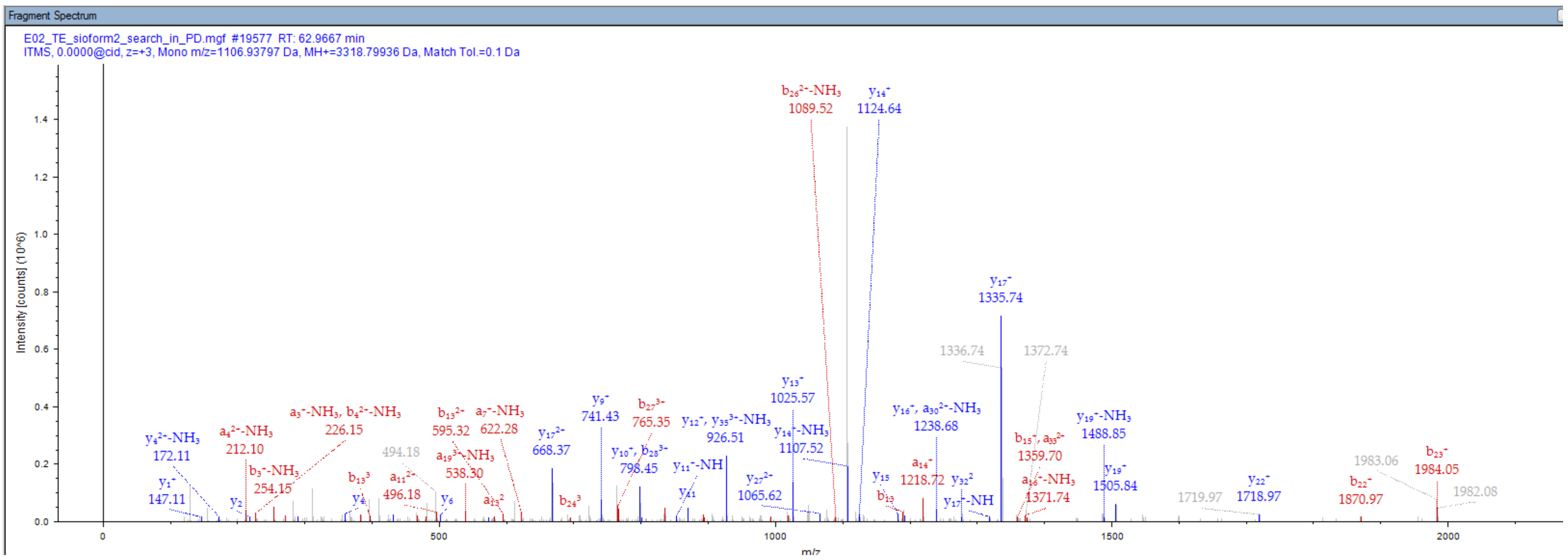

#1	a⁺	a²⁺	a³⁺	b⁺	b²⁺	b³⁺	Seq.	y⁺	y²⁺	y³⁺	#2
2	115.08659	58.04693	39.03371	143.08150	72.04439	48.36535	A	3247.77167	1624.38947	1083.26207	39
3	243.18155	122.09441	81.73204	271.17647	136.09187	91.06367	K	3176.73456	1588.87092	1059.58304	38
4	440.20591	220.60659	147.40682	468.20082	234.60405	156.73846	Y-Chlorinat...	3048.63959	1524.82343	1016.88472	37
5	497.22737	249.11732	166.41398	525.22229	263.11478	175.74561	G	2851.61524	1426.31126	951.20993	36
6	568.26449	284.63588	190.09301	596.25940	298.63334	199.42465	A	2794.59377	1397.80052	932.20278	35
7	639.30160	320.15444	213.77205	667.29651	334.15190	223.10369	A	2723.55666	1362.28197	908.52374	34
8	738.37001	369.68865	246.79486	766.36493	383.68610	256.12649	V	2652.51955	1326.76341	884.84470	33
9	835.42278	418.21503	279.14578	863.41769	432.21248	288.47742	P	2553.45113	1277.22920	851.82189	32
10	892.44424	446.72576	298.15293	920.43916	460.72322	307.48457	G	2456.39837	1228.70282	819.47097	31
11	991.51266	496.25997	331.17574	1019.50757	510.25742	340.50737	V	2399.37690	1200.19209	800.46382	30
12	1104.59672	552.80200	368.87042	1132.59163	566.79946	378.20206	L	2300.30849	1150.65788	767.44101	29
13	1161.61818	581.31273	387.87758	1189.61310	595.31019	397.20922	G	2187.22443	1094.11585	729.74633	28
14	1218.63965	609.82346	406.88473	1246.63456	623.82092	416.21637	G	2130.20296	1065.60512	710.73917	27
15	1331.72371	666.36549	444.57942	1359.71863	680.36295	453.91106	L	2073.18150	1037.09439	691.73202	26
16	1388.74517	694.87623	463.58658	1416.74009	708.87368	472.91821	G	1960.09743	980.55236	654.03733	25
17	1459.78229	730.39478	487.26561	1487.77720	744.39224	496.59725	A	1903.07597	952.04162	635.03017	24
18	1572.86635	786.93681	524.96030	1600.86127	800.93427	534.29194	L	1832.03886	916.52307	611.35114	23
19	1629.88782	815.44755	543.96746	1657.88273	829.44500	553.29909	G	1718.95479	859.98103	573.65645	22
20	1686.90928	843.95828	562.97461	1714.90419	857.95574	572.30625	G	1661.93333	831.47030	554.64929	21
21	1785.97769	893.49248	595.99742	1813.97261	907.48994	605.32905	V	1604.91187	802.95957	535.64214	20
22	1842.99916	922.00322	615.00457	1870.99407	936.00067	624.33621	G	1505.84345	753.42536	502.61934	19
23	1956.08322	978.54525	652.69926	1984.07814	992.54271	662.03090	I	1448.82199	724.91463	483.61218	18
24	2053.13598	1027.07163	685.05018	2081.13090	1041.06909	694.38182	P	1335.73792	668.37260	445.91749	17
25	2110.15745	1055.58236	704.05733	2138.15236	1069.57982	713.38897	G	1238.68516	619.84622	413.56657	16
26	2167.17891	1084.09309	723.06449	2195.17383	1098.09055	732.39613	G	1181.66370	591.33549	394.55942	15
27	2266.24733	1133.62730	756.08729	2294.24224	1147.62476	765.41893	V	1124.64223	562.82475	375.55226	14
28	2365.31574	1183.16151	789.11010	2393.31065	1197.15897	798.44174	V	1025.57382	513.29055	342.52946	13
29	2422.33720	1211.67224	808.11725	2450.33212	1225.66970	817.44889	G	926.50541	463.75634	309.50665	12
30	2493.37432	1247.19080	831.79629	2521.36923	1261.18825	841.12793	A	869.48394	435.24561	290.49950	11
31	2550.39578	1275.70153	850.80344	2578.39070	1289.69899	860.13508	G	798.44683	399.72705	266.82046	10
32	2647.44854	1324.22791	883.15437	2675.44346	1338.22537	892.48600	P	741.42536	371.21632	247.81331	9
33	2718.48566	1359.74647	906.83340	2746.48057	1373.74392	916.16504	A	644.37260	322.68994	215.46238	8
34	2789.52277	1395.26502	930.51244	2817.51769	1409.26248	939.84408	A	573.33549	287.17138	191.78335	7
35	2860.55989	1430.78358	954.19148	2888.55480	1444.78104	963.52312	A	502.29837	251.65282	168.10431	6
36	2931.59700	1466.30214	977.87052	2959.59191	1480.29960	987.20216	A	431.26126	216.13427	144.42527	5
37	3002.63411	1501.82070	1001.54956	3030.62903	1515.81815	1010.88119	A	360.22415	180.61571	120.74623	4
38	3073.67123	1537.33925	1025.22859	3101.66614	1551.33671	1034.56023	A	289.18703	145.09715	97.06720	3
39	3144.70834	1572.85781	1048.90763	3172.70326	1586.85527	1058.23927	A	218.14992	109.57860	73.38816	2
40							K	147.11280	74.06004	49.70912	1

AAKYGVGTPAAAAK, Y4-dichlorination

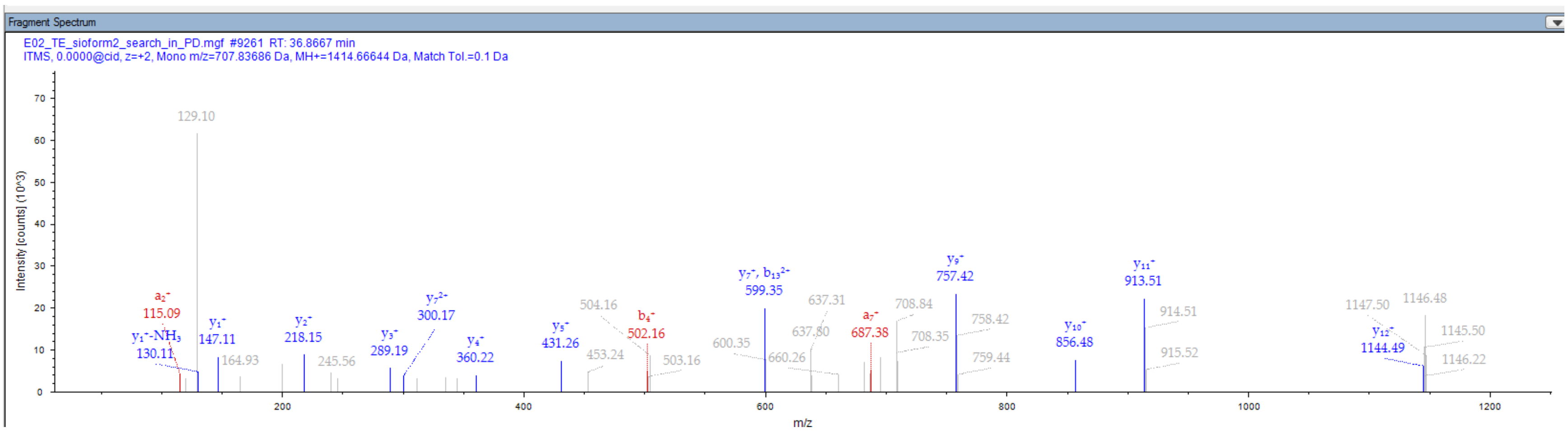

#1	a^+	a^{2+}	b^+	b^{2+}	Seq.	y^+	y^{2+}	#2
1	44.04948	22.52838	72.04439	36.52583	A			15
2	115.08659	58.04693	143.08150	72.04439	A	1343.62762	672.31745	14
3	243.18155	122.09441	271.17647	136.09187	K	1272.59050	636.79889	13
4	474.16694	237.58711	502.16185	251.58456	Y-dichlorin...	1144.49554	572.75141	12
5	531.18840	266.09784	559.18331	280.09530	G	913.51016	457.25872	11
6	630.25681	315.63205	658.25173	329.62950	V	856.48869	428.74798	10
7	687.27828	344.14278	715.27319	358.14023	G	757.42028	379.21378	9
8	788.32596	394.66662	816.32087	408.66407	T	700.39882	350.70305	8
9	885.37872	443.19300	913.37363	457.19046	P	599.35114	300.17921	7
10	956.41583	478.71155	984.41075	492.70901	A	502.29837	251.65282	6
11	1027.45295	514.23011	1055.44786	528.22757	A	431.26126	216.13427	5
12	1098.49006	549.74867	1126.48498	563.74613	A	360.22415	180.61571	4
13	1169.52717	585.26723	1197.52209	599.26468	A	289.18703	145.09715	3
14	1240.56429	620.78578	1268.55920	634.78324	A	218.14992	109.57860	2
15					K	147.11280	74.06004	1

AGYPTGTGVGPQAAAAAAAK

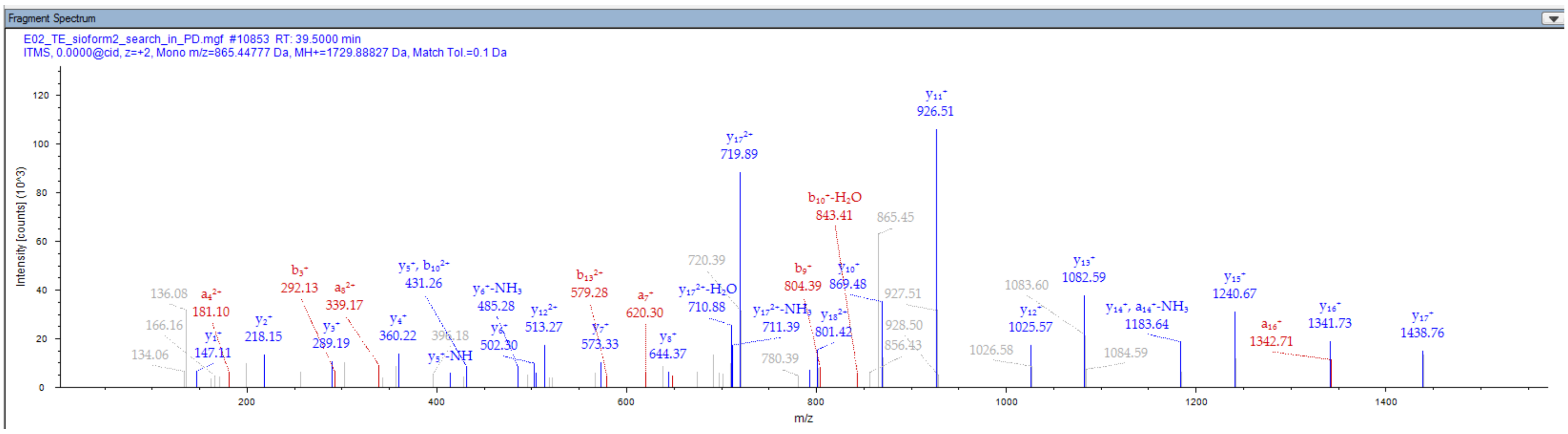

Ion Series | Neutral Losses | Precursor Ions | Internal Fragments

#1	a^+	a^{2+}	b^+	b^{2+}	Seq.	y^+	y^{2+}	#2
1	44.04948	22.52838	72.04439	36.52583	A			20
2	101.07094	51.03911	129.06585	65.03657	G	1658.84966	829.92847	19
3	264.13427	132.57077	292.12918	146.56823	Y	1601.82820	801.41774	18
4	361.18703	181.09715	389.18195	195.09461	P	1438.76487	719.88607	17
5	462.23471	231.62099	490.22962	245.61845	T	1341.71210	671.35969	16
6	519.25617	260.13173	547.25109	274.12918	G	1240.66443	620.83585	15
7	620.30385	310.65556	648.29877	324.65302	T	1183.64296	592.32512	14
8	677.32532	339.16630	705.32023	353.16375	G	1082.59528	541.80128	13
9	776.39373	388.70050	804.38864	402.69796	V	1025.57382	513.29055	12
10	833.41519	417.21124	861.41011	431.20869	G	926.50541	463.75634	11
11	930.46796	465.73762	958.46287	479.73507	P	869.48394	435.24561	10
12	1058.52653	529.76691	1086.52145	543.76436	Q	772.43118	386.71923	9
13	1129.56365	565.28546	1157.55856	579.28292	A	644.37260	322.68994	8
14	1200.60076	600.80402	1228.59568	614.80148	A	573.33549	287.17138	7
15	1271.63788	636.32258	1299.63279	650.32003	A	502.29837	251.65282	6
16	1342.67499	671.84113	1370.66990	685.83859	A	431.26126	216.13427	5
17	1413.71210	707.35969	1441.70702	721.35715	A	360.22415	180.61571	4
18	1484.74922	742.87825	1512.74413	756.87570	A	289.18703	145.09715	3
19	1555.78633	778.39680	1583.78125	792.39426	A	218.14992	109.57860	2
20					K	147.11280	74.06004	1

AGYPTGTGVGPQAAAAAAAK, Y3-Chlorination (33.96103 Da)

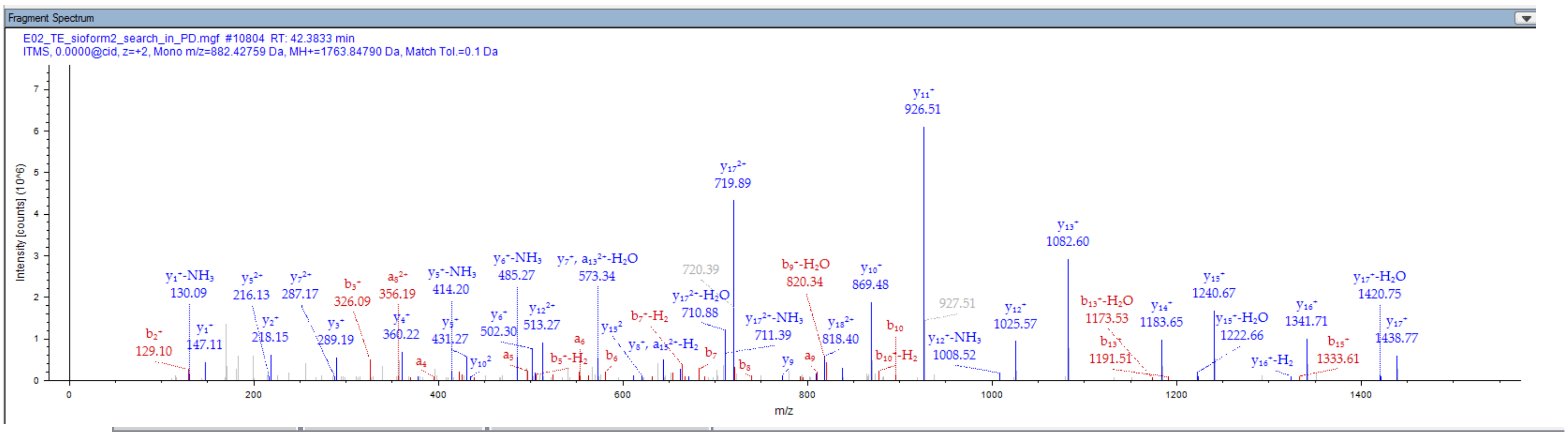

#1	a⁺	a²⁺	b⁺	b²⁺	Seq.	y⁺	y²⁺	#2
1	44.04948	22.52838	72.04439	36.52583	A			20
2	101.07094	51.03911	129.06585	65.03657	G	1692.81069	846.90898	19
3	298.09530	149.55129	326.09021	163.54874	Y-Chlorinat...	1635.78922	818.39825	18
4	395.14806	198.07767	423.14297	212.07513	P	1438.76487	719.88607	17
5	496.19574	248.60151	524.19065	262.59896	T	1341.71210	671.35969	16
6	553.21720	277.11224	581.21212	291.10970	G	1240.66443	620.83585	15
7	654.26488	327.63608	682.25979	341.63354	T	1183.64296	592.32512	14
8	711.28634	356.14681	739.28126	370.14427	G	1082.59528	541.80128	13
9	810.35476	405.68102	838.34967	419.67847	V	1025.57382	513.29055	12
10	867.37622	434.19175	895.37114	448.18921	G	926.50541	463.75634	11
11	964.42899	482.71813	992.42390	496.71559	P	869.48394	435.24561	10
12	1092.48756	546.74742	1120.48248	560.74488	Q	772.43118	386.71923	9
13	1163.52468	582.26598	1191.51959	596.26343	A	644.37260	322.68994	8
14	1234.56179	617.78453	1262.55670	631.78199	A	573.33549	287.17138	7
15	1305.59890	653.30309	1333.59382	667.30055	A	502.29837	251.65282	6
16	1376.63602	688.82165	1404.63093	702.81910	A	431.26126	216.13427	5
17	1447.67313	724.34020	1475.66805	738.33766	A	360.22415	180.61571	4
18	1518.71025	759.85876	1546.70516	773.85622	A	289.18703	145.09715	3
19	1589.74736	795.37732	1617.74227	809.37478	A	218.14992	109.57860	2
20					K	147.11280	74.06004	1

AGYPTGTGVGPQAAAAAAK, Q12-Deamidated (0.98402 Da), Y3-Chlorination (33.96103 Da)

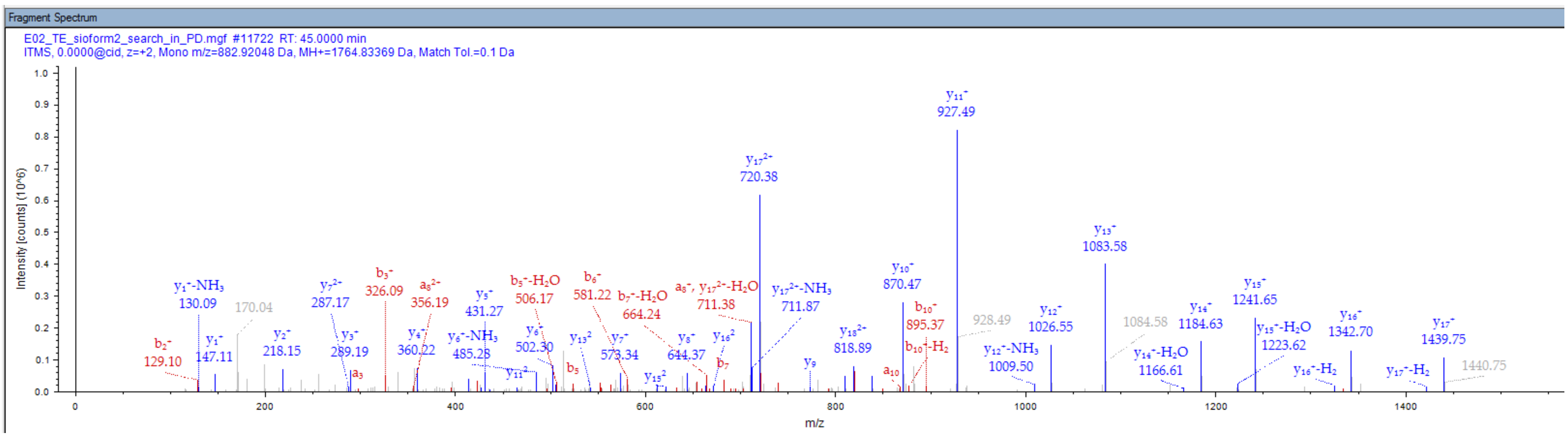

#1	a⁺	a²⁺	b⁺	b²⁺	Seq.	y⁺	y²⁺	#2
1	44.04948	22.52838	72.04439	36.52583	A			20
2	101.07094	51.03911	129.06585	65.03657	G	1693.79470	847.40099	19
3	298.09530	149.55129	326.09021	163.54874	Y-Chlorinat...	1636.77324	818.89026	18
4	395.14806	198.07767	423.14297	212.07513	P	1439.74888	720.37808	17
5	496.19574	248.60151	524.19065	262.59896	T	1342.69612	671.85170	16
6	553.21720	277.11224	581.21212	291.10970	G	1241.64844	621.32786	15
7	654.26488	327.63608	682.25979	341.63354	T	1184.62698	592.81713	14
8	711.28634	356.14681	739.28126	370.14427	G	1083.57930	542.29329	13
9	810.35476	405.68102	838.34967	419.67847	V	1026.55784	513.78256	12
10	867.37622	434.19175	895.37114	448.18921	G	927.48942	464.24835	11
11	964.42899	482.71813	992.42390	496.71559	P	870.46796	435.73762	10
12	1093.47158	547.23943	1121.46649	561.23688	Q-Deamid...	773.41519	387.21124	9
13	1164.50869	582.75798	1192.50361	596.75544	A	644.37260	322.68994	8
14	1235.54581	618.27654	1263.54072	632.27400	A	573.33549	287.17138	7
15	1306.58292	653.79510	1334.57783	667.79256	A	502.29837	251.65282	6
16	1377.62003	689.31366	1405.61495	703.31111	A	431.26126	216.13427	5
17	1448.65715	724.83221	1476.65206	738.82967	A	360.22415	180.61571	4
18	1519.69426	760.35077	1547.68918	774.34823	A	289.18703	145.09715	3
19	1590.73138	795.86933	1618.72629	809.86678	A	218.14992	109.57860	2
20					K	147.11280	74.06004	1

AGYPTGTGVGPQAAAAAAAK, Y3-dichlorination (67.92206 Da)

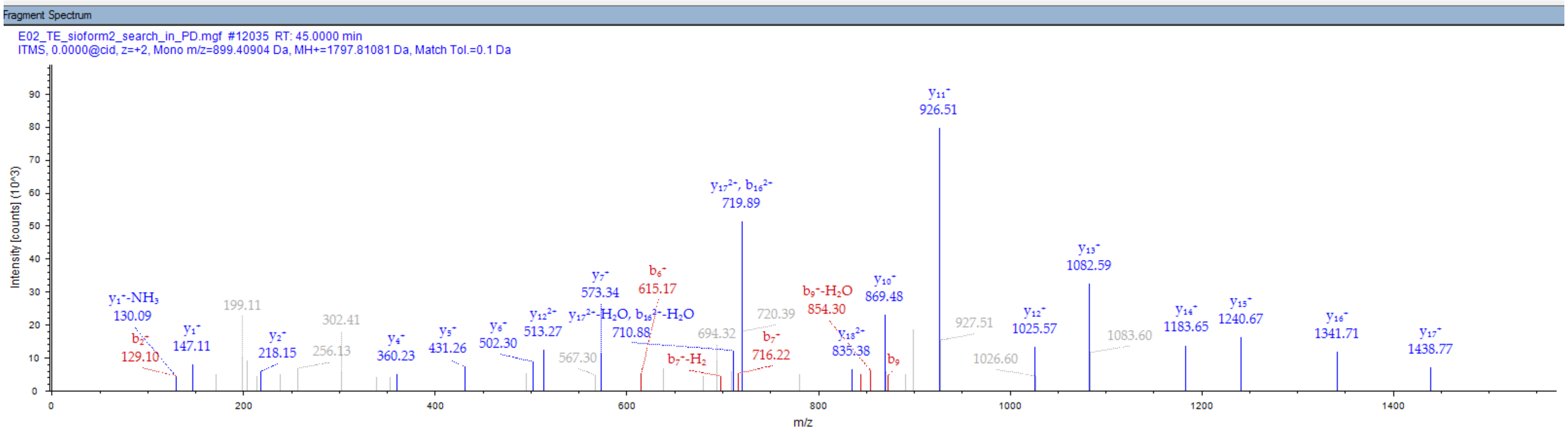

#1	a⁺	a²⁺	b⁺	b²⁺	Seq.	y⁺	y²⁺	#2
1	44.04948	22.52838	72.04439	36.52583	A			20
2	101.07094	51.03911	129.06585	65.03657	G	1726.77171	863.88950	19
3	332.05632	166.53180	360.05124	180.52926	Y-dichlorin...	1669.75025	835.37876	18
4	429.10909	215.05818	457.10400	229.05564	P	1438.76487	719.88607	17
5	530.15677	265.58202	558.15168	279.57948	T	1341.71210	671.35969	16
6	587.17823	294.09275	615.17314	308.09021	G	1240.66443	620.83585	15
7	688.22591	344.61659	716.22082	358.61405	T	1183.64296	592.32512	14
8	745.24737	373.12732	773.24229	387.12478	G	1082.59528	541.80128	13
9	844.31578	422.66153	872.31070	436.65899	V	1025.57382	513.29055	12
10	901.33725	451.17226	929.33216	465.16972	G	926.50541	463.75634	11
11	998.39001	499.69864	1026.38493	513.69610	P	869.48394	435.24561	10
12	1126.44859	563.72793	1154.44350	577.72539	Q	772.43118	386.71923	9
13	1197.48570	599.24649	1225.48062	613.24395	A	644.37260	322.68994	8
14	1268.52282	634.76505	1296.51773	648.76250	A	573.33549	287.17138	7
15	1339.55993	670.28360	1367.55485	684.28106	A	502.29837	251.65282	6
16	1410.59704	705.80216	1438.59196	719.79962	A	431.26126	216.13427	5
17	1481.63416	741.32072	1509.62907	755.31817	A	360.22415	180.61571	4
18	1552.67127	776.83927	1580.66619	790.83673	A	289.18703	145.09715	3
19	1623.70839	812.35783	1651.70330	826.35529	A	218.14992	109.57860	2
20					K	147.11280	74.06004	1

AGYPTGTGVGPQAAAAAAK, Q12-Deamidated (0.98402 Da), Y3-dichlorination (67.92206 Da)

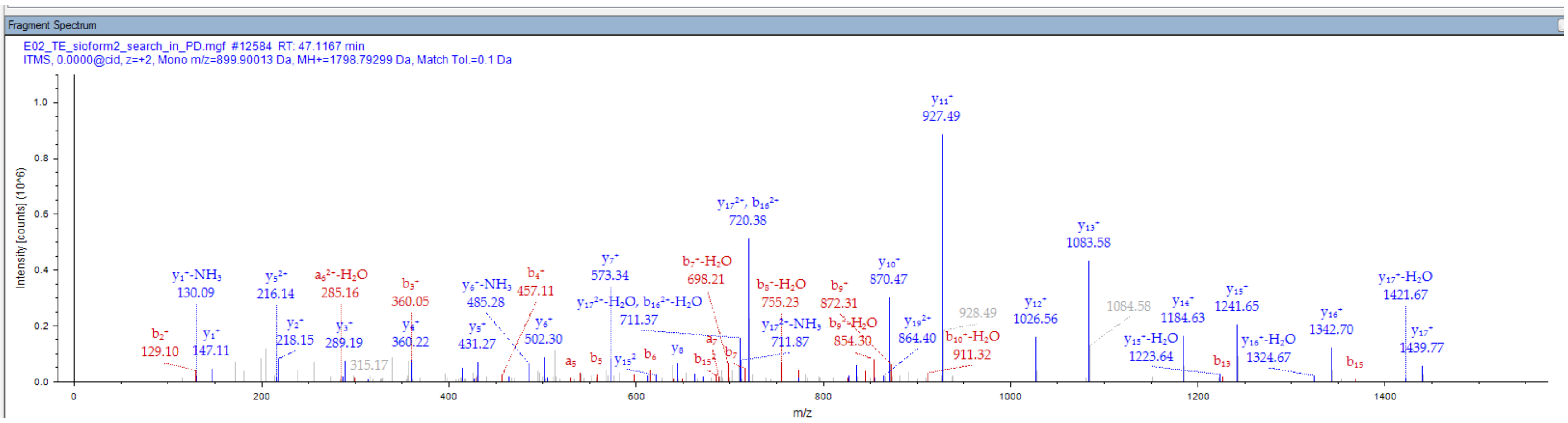

#1	a+	a2+	b+	b2+	Seq.	y+	y2+	#2
1	44.04948	22.52838	72.04439	36.52583	A			20
2	101.07094	51.03911	129.06585	65.03657	G	1727.75573	864.38150	19
3	332.05632	166.53180	360.05124	180.52926	Y-dichlorin...	1670.73427	835.87077	18
4	429.10909	215.05818	457.10400	229.05564	P	1439.74888	720.37808	17
5	530.15677	265.58202	558.15168	279.57948	T	1342.69612	671.85170	16
6	587.17823	294.09275	615.17314	308.09021	G	1241.64844	621.32786	15
7	688.22591	344.61659	716.22082	358.61405	T	1184.62698	592.81713	14
8	745.24737	373.12732	773.24229	387.12478	G	1083.57930	542.29329	13
9	844.31578	422.66153	872.31070	436.65899	V	1026.55784	513.78256	12
10	901.33725	451.17226	929.33216	465.16972	G	927.48942	464.24835	11
11	998.39001	499.69864	1026.38493	513.69610	P	870.46796	435.73762	10
12	1127.43261	564.21994	1155.42752	578.21740	Q-Deamid...	773.41519	387.21124	9
13	1198.46972	599.73850	1226.46463	613.73596	A	644.37260	322.68994	8
14	1269.50683	635.25705	1297.50175	649.25451	A	573.33549	287.17138	7
15	1340.54395	670.77561	1368.53886	684.77307	A	502.29837	251.65282	6
16	1411.58106	706.29417	1439.57598	720.29163	A	431.26126	216.13427	5
17	1482.61817	741.81273	1510.61309	755.81018	A	360.22415	180.61571	4
18	1553.65529	777.33128	1581.65020	791.32874	A	289.18703	145.09715	3
19	1624.69240	812.84984	1652.68732	826.84730	A	218.14992	109.57860	2
20					K	147.11280	74.06004	1

APGVGGAFAGIPGVGPFGGPQPGVPLGYPIK,

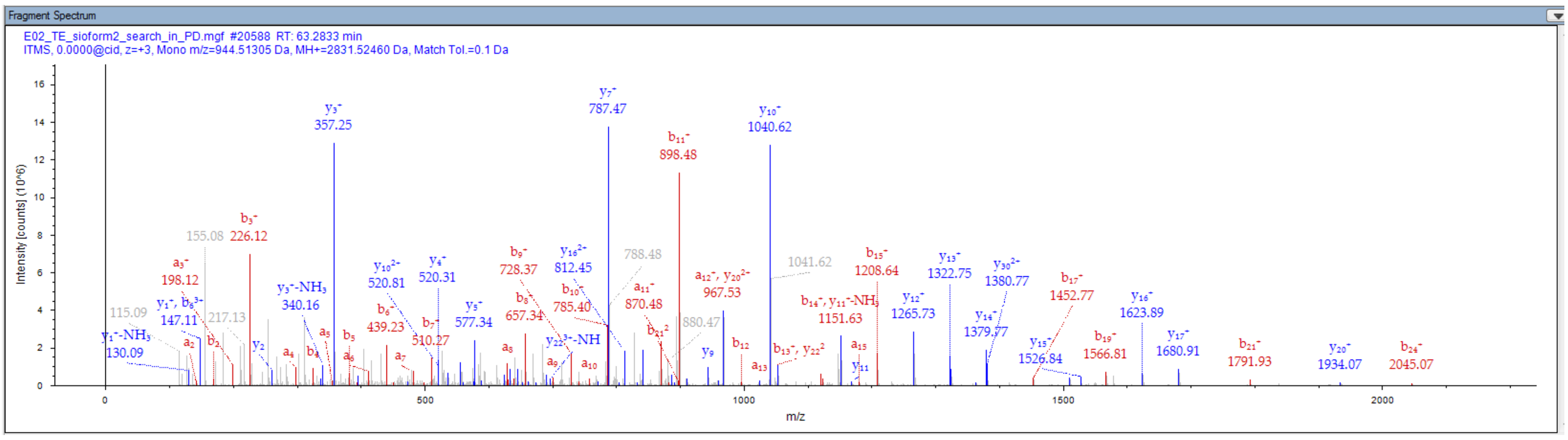

#1	a⁺	a²⁺	a³⁺	b⁺	b²⁺	b³⁺	Seq.	y⁺	y²⁺	y³⁺	#2
1	44.04948	22.52838	15.35468	72.04439	36.52583	24.68631	A				31
2	141.10224	71.05476	47.70560	169.09715	85.05222	57.03724	P	2760.48718	1380.74723	920.83391	30
3	198.12370	99.56549	66.71275	226.11862	113.56295	76.04439	G	2663.43442	1332.22085	888.48299	29
4	297.19212	149.09970	99.73556	325.18703	163.09715	109.06720	V	2606.41295	1303.71012	869.47584	28
5	354.21358	177.61043	118.74271	382.20850	191.60789	128.07435	G	2507.34454	1254.17591	836.45303	27
6	411.23504	206.12116	137.74987	439.22996	220.11862	147.08150	G	2450.32308	1225.66518	817.44588	26
7	482.27216	241.63972	161.42890	510.26707	255.63717	170.76054	A	2393.30161	1197.15445	798.43872	25
8	629.34057	315.17392	210.45171	657.33549	329.17138	219.78335	F	2322.26450	1161.63589	774.75968	24
9	700.37769	350.69248	234.13075	728.37260	364.68994	243.46238	A	2175.19609	1088.10168	725.73688	23
10	757.39915	379.20321	253.13790	785.39406	393.20067	262.46954	G	2104.15897	1052.58312	702.05784	22
11	870.48321	435.74525	290.83259	898.47813	449.74270	300.16423	I	2047.13751	1024.07239	683.05069	21
12	967.53598	484.27163	323.18351	995.53089	498.26908	332.51515	P	1934.05344	967.53036	645.35600	20
13	1024.55744	512.78236	342.19066	1052.55236	526.77982	351.52230	G	1837.00068	919.00398	613.00508	19
14	1123.62585	562.31657	375.21347	1151.62077	576.31402	384.54511	V	1779.97922	890.49325	593.99792	18
15	1180.64732	590.82730	394.22062	1208.64223	604.82475	403.55226	G	1680.91080	840.95904	560.97512	17
16	1277.70008	639.35368	426.57155	1305.69500	653.35114	435.90318	P	1623.88934	812.44831	541.96796	16
17	1424.76850	712.88789	475.59435	1452.76341	726.88534	484.92599	F	1526.83658	763.92193	509.61704	15
18	1481.78996	741.39862	494.60150	1509.78487	755.39608	503.93314	G	1379.76816	690.38772	460.59424	14
19	1538.81142	769.90935	513.60866	1566.80634	783.90681	522.94030	G	1322.74670	661.87699	441.58708	13
20	1635.86419	818.43573	545.95958	1663.85910	832.43319	555.29122	P	1265.72523	633.36626	422.57993	12
21	1763.92276	882.46502	588.64577	1791.91768	896.46248	597.97741	Q	1168.67247	584.83987	390.22901	11
22	1860.97553	930.99140	620.99669	1888.97044	944.98886	630.32833	P	1040.61389	520.81058	347.54282	10
23	1917.99699	959.50213	640.00385	1945.99191	973.49959	649.33549	G	943.56113	472.28420	315.19189	9
24	2017.06541	1009.03634	673.02665	2045.06032	1023.03380	682.35829	V	886.53967	443.77347	296.18474	8
25	2114.11817	1057.56272	705.37757	2142.11308	1071.56018	714.70921	P	787.47125	394.23926	263.16194	7
26	2227.20223	1114.10476	743.07226	2255.19715	1128.10221	752.40390	L	690.41849	345.71288	230.81101	6
27	2284.22370	1142.61549	762.07942	2312.21861	1156.61294	771.41106	G	577.33442	289.17085	193.11633	5
28	2447.28703	1224.14715	816.43386	2475.28194	1238.14461	825.76550	Y	520.31296	260.66012	174.10917	4
29	2544.33979	1272.67353	848.78478	2572.33470	1286.67099	858.11642	P	357.24963	179.12845	119.75473	3
30	2657.42385	1329.21557	886.47947	2685.41877	1343.21302	895.81111	I	260.19687	130.60207	87.40381	2
31							K	147.11280	74.06004	49.70912	1

APGVGGAFAGIPGVGPFGGPQPGVPLGYPIK, Y28-dichlorination (67.92206 Da)

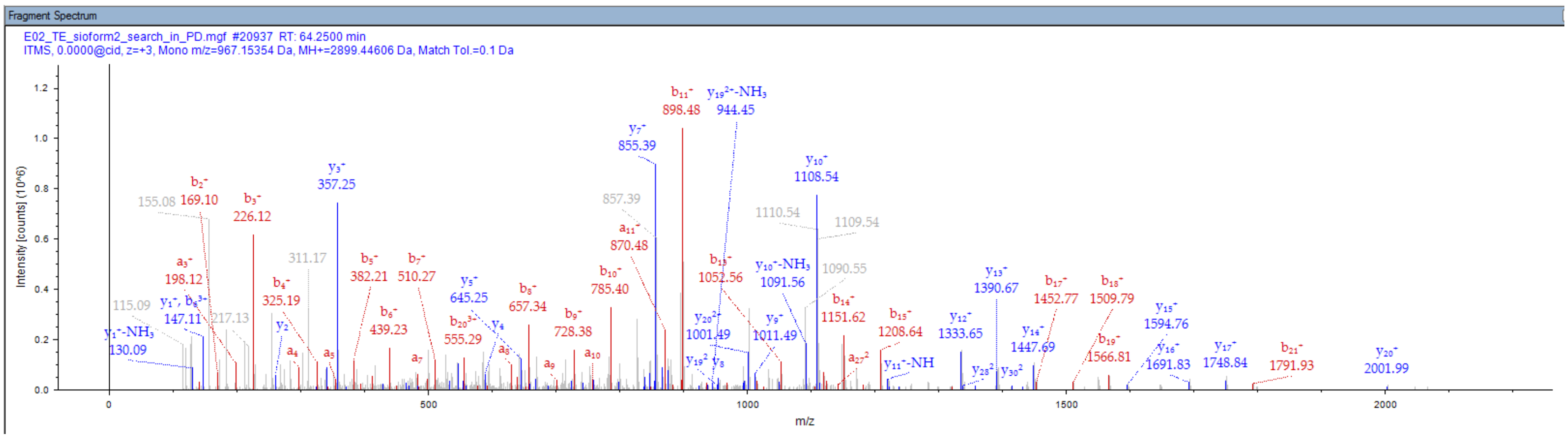

#1	a⁺	a²⁺	a³⁺	b⁺	b²⁺	b³⁺	Seq.	y⁺	y²⁺	y³⁺	#2
1	44.04948	22.52838	15.35468	72.04439	36.52583	24.68631	A				31
2	141.10224	71.05476	47.70560	169.09715	85.05222	57.03724	P	2828.40924	1414.70826	943.47460	30
3	198.12370	99.56549	66.71275	226.11862	113.56295	76.04439	G	2731.35647	1366.18188	911.12368	29
4	297.19212	149.09970	99.73556	325.18703	163.09715	109.06720	V	2674.33501	1337.67114	892.11652	28
5	354.21358	177.61043	118.74271	382.20850	191.60789	128.07435	G	2575.26660	1288.13694	859.09372	27
6	411.23504	206.12116	137.74987	439.22996	220.11862	147.08150	G	2518.24513	1259.62620	840.08656	26
7	482.27216	241.63972	161.42890	510.26707	255.63717	170.76054	A	2461.22367	1231.11547	821.07941	25
8	629.34057	315.17392	210.45171	657.33549	329.17138	219.78335	F	2390.18655	1195.59692	797.40037	24
9	700.37769	350.69248	234.13075	728.37260	364.68994	243.46238	A	2243.11814	1122.06271	748.37756	23
10	757.39915	379.20321	253.13790	785.39406	393.20067	262.46954	G	2172.08103	1086.54415	724.69853	22
11	870.48321	435.74525	290.83259	898.47813	449.74270	300.16423	I	2115.05956	1058.03342	705.69137	21
12	967.53598	484.27163	323.18351	995.53089	498.26908	332.51515	P	2001.97550	1001.49139	667.99668	20
13	1024.55744	512.78236	342.19066	1052.55236	526.77982	351.52230	G	1904.92274	952.96501	635.64576	19
14	1123.62585	562.31657	375.21347	1151.62077	576.31402	384.54511	V	1847.90127	924.45427	616.63861	18
15	1180.64732	590.82730	394.22062	1208.64223	604.82475	403.55226	G	1748.83286	874.92007	583.61580	17
16	1277.70008	639.35368	426.57155	1305.69500	653.35114	435.90318	P	1691.81139	846.40934	564.60865	16
17	1424.76850	712.88789	475.59435	1452.76341	726.88534	484.92599	F	1594.75863	797.88295	532.25773	15
18	1481.78996	741.39862	494.60150	1509.78487	755.39608	503.93314	G	1447.69022	724.34875	483.23492	14
19	1538.81142	769.90935	513.60866	1566.80634	783.90681	522.94030	G	1390.66875	695.83801	464.22777	13
20	1635.86419	818.43573	545.95958	1663.85910	832.43319	555.29122	P	1333.64729	667.32728	445.22061	12
21	1763.92276	882.46502	588.64577	1791.91768	896.46248	597.97741	Q	1236.59453	618.80090	412.86969	11
22	1860.97553	930.99140	620.99669	1888.97044	944.98886	630.32833	P	1108.53595	554.77161	370.18350	10
23	1917.99699	959.50213	640.00385	1945.99191	973.49959	649.33549	G	1011.48318	506.24523	337.83258	9
24	2017.06541	1009.03634	673.02665	2045.06032	1023.03380	682.35829	V	954.46172	477.73450	318.82542	8
25	2114.11817	1057.56272	705.37757	2142.11308	1071.56018	714.70921	P	855.39331	428.20029	285.80262	7
26	2227.20223	1114.10476	743.07226	2255.19715	1128.10221	752.40390	L	758.34054	379.67391	253.45170	6
27	2284.22370	1142.61549	762.07942	2312.21861	1156.61294	771.41106	G	645.25648	323.13188	215.75701	5
28	2515.20908	1258.10818	839.07454	2543.20400	1272.10564	848.40618	Y-dichlorin...	588.23502	294.62115	196.74986	4
29	2612.26184	1306.63456	871.42547	2640.25676	1320.63202	880.75710	P	357.24963	179.12845	119.75473	3
30	2725.34591	1363.17659	909.12015	2753.34082	1377.17405	918.45179	I	260.19687	130.60207	87.40381	2
31							K	147.11280	74.06004	49.70912	1

LPGGYGLPYTTGK

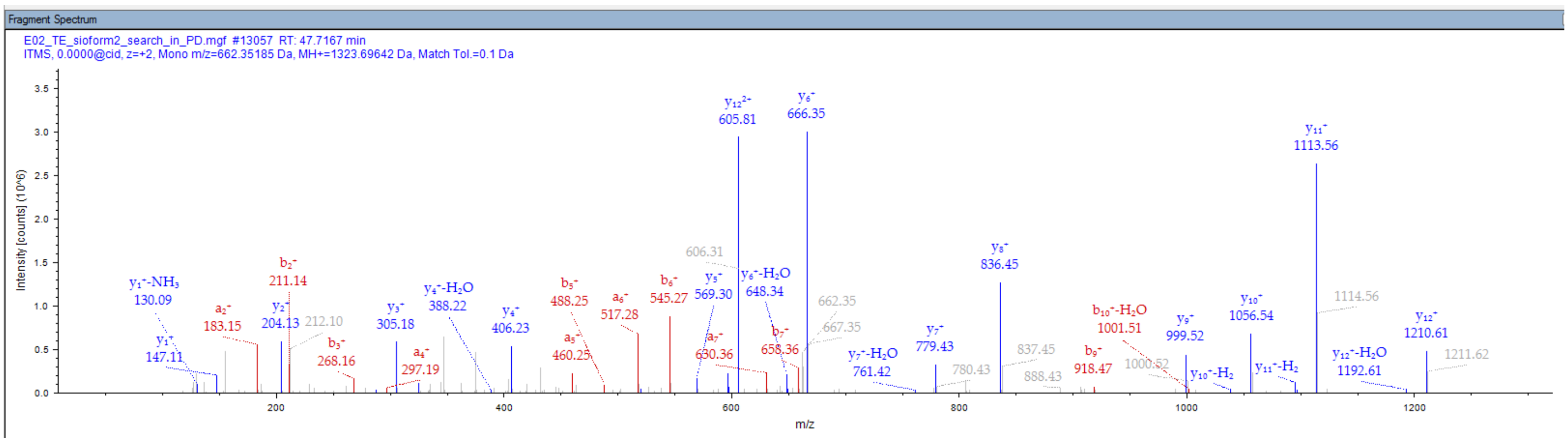

#1	a⁺	a²⁺	b⁺	b²⁺	Seq.	y⁺	y²⁺	#2
1	86.09643	43.55185	114.09134	57.54931	L			13
2	183.14919	92.07823	211.14410	106.07569	P	1210.61026	605.80877	12
3	240.17065	120.58897	268.16557	134.58642	G	1113.55750	557.28239	11
4	297.19212	149.09970	325.18703	163.09715	G	1056.53604	528.77166	10
5	460.25545	230.63136	488.25036	244.62882	Y	999.51457	500.26092	9
6	517.27691	259.14209	545.27182	273.13955	G	836.45124	418.72926	8
7	630.36097	315.68412	658.35589	329.68158	L	779.42978	390.21853	7
8	727.41374	364.21051	755.40865	378.20796	P	666.34572	333.67650	6
9	890.47707	445.74217	918.47198	459.73963	Y	569.29295	285.15011	5
10	991.52474	496.26601	1019.51966	510.26347	T	406.22962	203.61845	4
11	1092.57242	546.78985	1120.56734	560.78731	T	305.18195	153.09461	3
12	1149.59389	575.30058	1177.58880	589.29804	G	204.13427	102.57077	2
13					K	147.11280	74.06004	1

LPGGYGLPYTTGK, Y5-Chlorination (33.96103 Da)

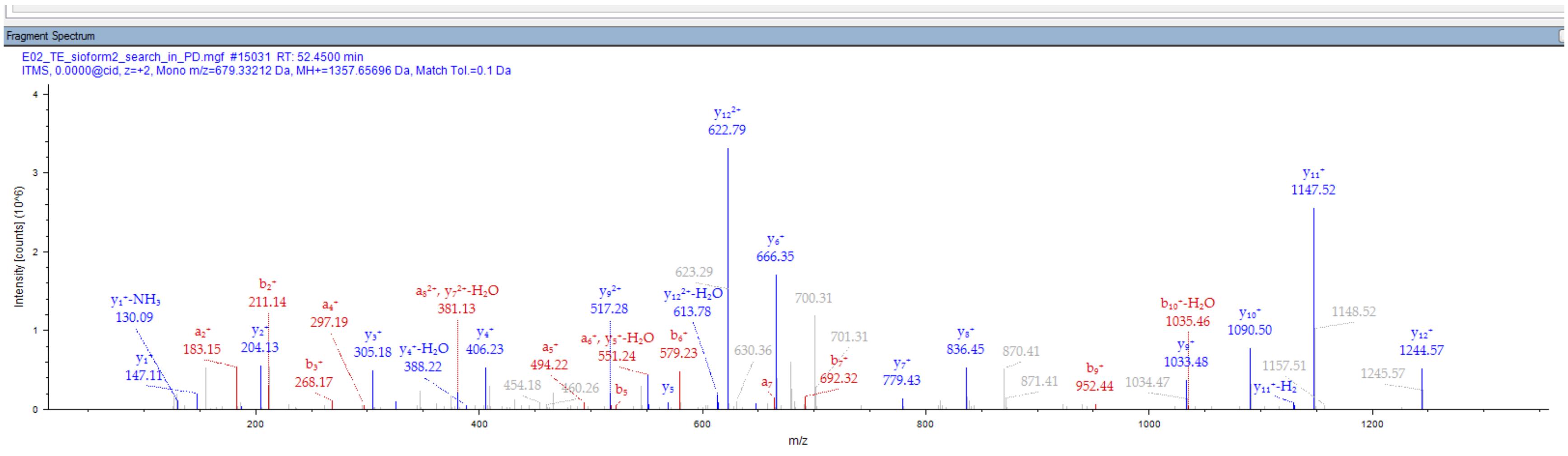

#1	a⁺	a²⁺	b⁺	b²⁺	Seq.	y⁺	y²⁺	#2
1	86.09643	43.55185	114.09134	57.54931	L			13
2	183.14919	92.07823	211.14410	106.07569	P	1244.57129	622.78928	12
3	240.17065	120.58897	268.16557	134.58642	G	1147.51853	574.26290	11
4	297.19212	149.09970	325.18703	163.09715	G	1090.49706	545.75217	10
5	494.21647	247.61188	522.21139	261.60933	Y-Chlorinat...	1033.47560	517.24144	9
6	551.23794	276.12261	579.23285	290.12006	G	836.45124	418.72926	8
7	664.32200	332.66464	692.31692	346.66210	L	779.42978	390.21853	7
8	761.37477	381.19102	789.36968	395.18848	P	666.34572	333.67650	6
9	924.43809	462.72269	952.43301	476.72014	Y	569.29295	285.15011	5
10	1025.48577	513.24652	1053.48069	527.24398	T	406.22962	203.61845	4
11	1126.53345	563.77036	1154.52836	577.76782	T	305.18195	153.09461	3
12	1183.55491	592.28110	1211.54983	606.27855	G	204.13427	102.57077	2
13					K	147.11280	74.06004	1

LPGGYGLPYTTGK, Y9-Chlorination (33.96103 Da)

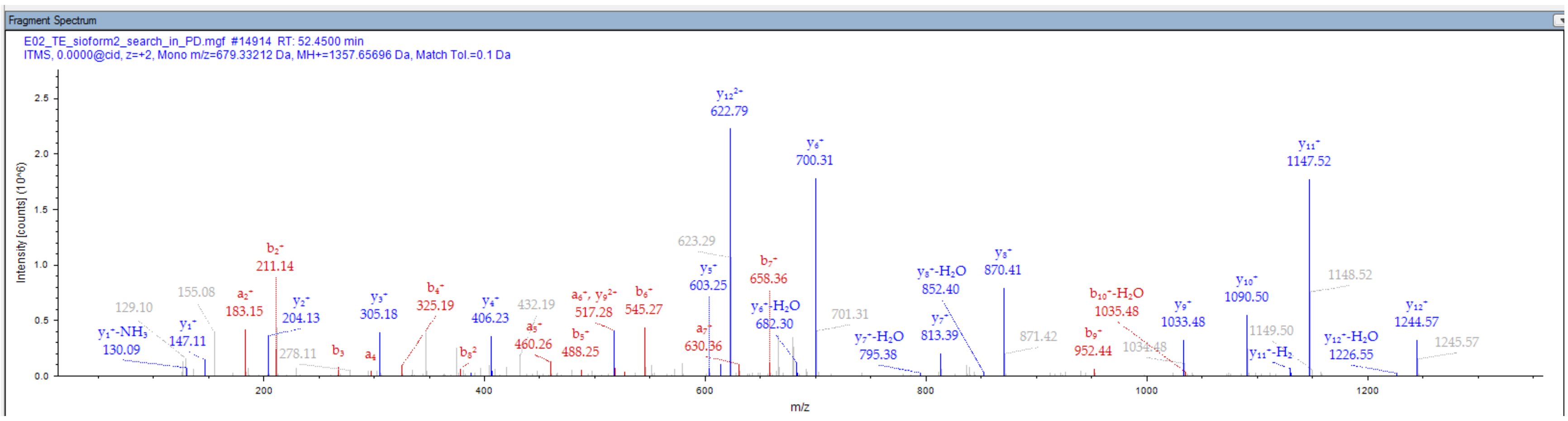

#1	a+	a2+	b+	b2+	Seq.	y+	y2+	#2
1	86.09643	43.55185	114.09134	57.54931	L			13
2	183.14919	92.07823	211.14410	106.07569	P	1244.57129	622.78928	12
3	240.17065	120.58897	268.16557	134.58642	G	1147.51853	574.26290	11
4	297.19212	149.09970	325.18703	163.09715	G	1090.49706	545.75217	10
5	460.25545	230.63136	488.25036	244.62882	Y	1033.47560	517.24144	9
6	517.27691	259.14209	545.27182	273.13955	G	870.41227	435.70977	8
7	630.36097	315.68412	658.35589	329.68158	L	813.39081	407.19904	7
8	727.41374	364.21051	755.40865	378.20796	P	700.30675	350.65701	6
9	924.43809	462.72269	952.43301	476.72014	Y-Chlorinat...	603.25398	302.13063	5
10	1025.48577	513.24652	1053.48069	527.24398	T	406.22962	203.61845	4
11	1126.53345	563.77036	1154.52836	577.76782	T	305.18195	153.09461	3
12	1183.55491	592.28110	1211.54983	606.27855	G	204.13427	102.57077	2
13					K	147.11280	74.06004	1

LPGGYGLPYTTGK, Y5-Chlorination (33.96103 Da), Y9-Chlorination (33.96103 Da)

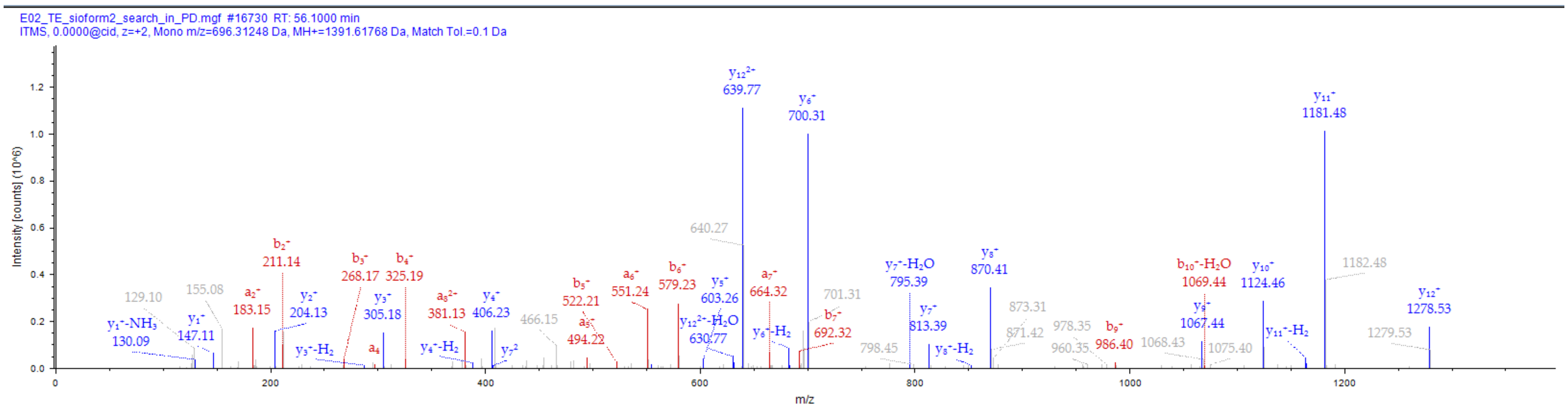

#1	a⁺	a²⁺	b⁺	b²⁺	Seq.	y⁺	y²⁺	#2
1	86.09643	43.55185	114.09134	57.54931	L			13
2	183.14919	92.07823	211.14410	106.07569	P	1278.53232	639.76980	12
3	240.17065	120.58897	268.16557	134.58642	G	1181.47956	591.24342	11
4	297.19212	149.09970	325.18703	163.09715	G	1124.45809	562.73268	10
5	494.21647	247.61188	522.21139	261.60933	Y-Chlorinat...	1067.43663	534.22195	9
6	551.23794	276.12261	579.23285	290.12006	G	870.41227	435.70977	8
7	664.32200	332.66464	692.31692	346.66210	L	813.39081	407.19904	7
8	761.37477	381.19102	789.36968	395.18848	P	700.30675	350.65701	6
9	958.39912	479.70320	986.39404	493.70066	Y-Chlorinat...	603.25398	302.13063	5
10	1059.44680	530.22704	1087.44171	544.22450	T	406.22962	203.61845	4
11	1160.49448	580.75088	1188.48939	594.74833	T	305.18195	153.09461	3
12	1217.51594	609.26161	1245.51086	623.25907	G	204.13427	102.57077	2
13					K	147.11280	74.06004	1

LPGGYGLPYTTGK, Y9-dichlorination (67.92206 Da)

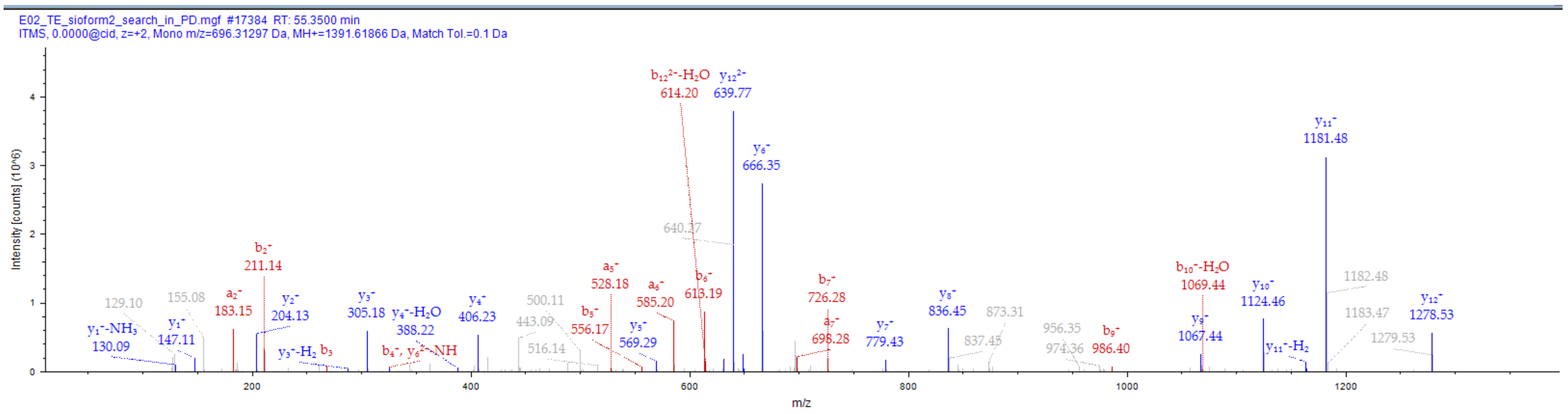

#1	a⁺	a²⁺	b⁺	b²⁺	Seq.	y⁺	y²⁺	#2
1	86.09643	43.55185	114.09134	57.54931	L			13
2	183.14919	92.07823	211.14410	106.07569	P	1278.53232	639.76980	12
3	240.17065	120.58897	268.16557	134.58642	G	1181.47956	591.24342	11
4	297.19212	149.09970	325.18703	163.09715	G	1124.45809	562.73268	10
5	528.17750	264.59239	556.17242	278.58985	Y-dichlorin...	1067.43663	534.22195	9
6	585.19896	293.10312	613.19388	307.10058	G	836.45124	418.72926	8
7	698.28303	349.64515	726.27794	363.64261	L	779.42978	390.21853	7
8	795.33579	398.17153	823.33071	412.16899	P	666.34572	333.67650	6
9	958.39912	479.70320	986.39404	493.70066	Y	569.29295	285.15011	5
10	1059.44680	530.22704	1087.44171	544.22450	T	406.22962	203.61845	4
11	1160.49448	580.75088	1188.48939	594.74833	T	305.18195	153.09461	3
12	1217.51594	609.26161	1245.51086	623.25907	G	204.13427	102.57077	2
13					K	147.11280	74.06004	1

LPGGYGLPYTTGK, Y5-dichlorination (67.92206 Da)

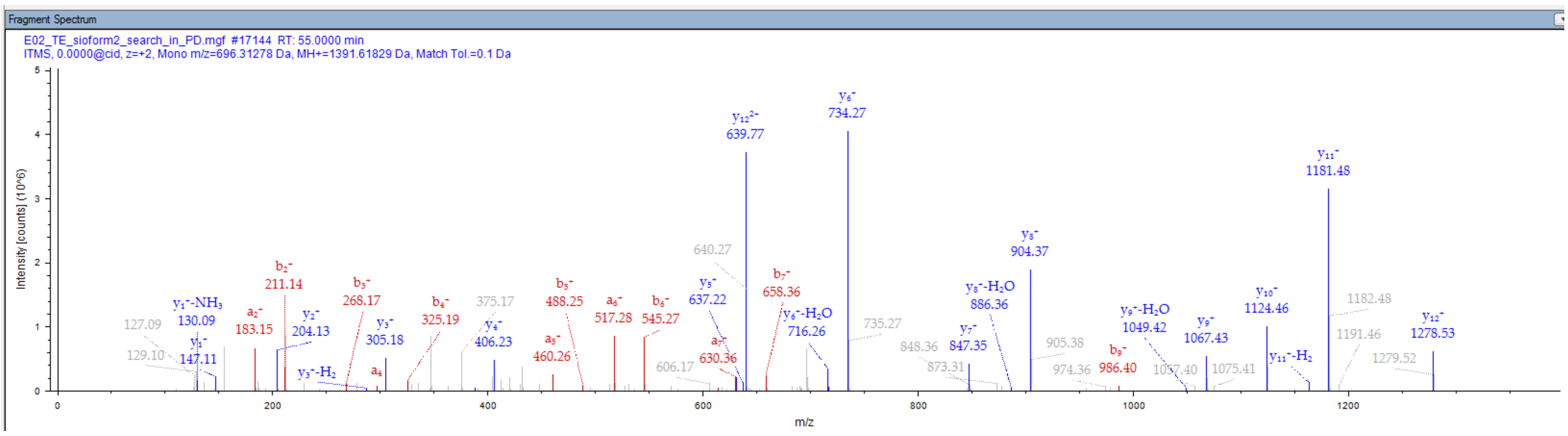

#1	a⁺	a²⁺	b⁺	b²⁺	Seq.	y⁺	y²⁺	#2
1	86.09643	43.55185	114.09134	57.54931	L			13
2	183.14919	92.07823	211.14410	106.07569	P	1278.53232	639.76980	12
3	240.17065	120.58897	268.16557	134.58642	G	1181.47956	591.24342	11
4	297.19212	149.09970	325.18703	163.09715	G	1124.45809	562.73268	10
5	460.25545	230.63136	488.25036	244.62882	Y	1067.43663	534.22195	9
6	517.27691	259.14209	545.27182	273.13955	G	904.37330	452.69029	8
7	630.36097	315.68412	658.35589	329.68158	L	847.35184	424.17956	7
8	727.41374	364.21051	755.40865	378.20796	P	734.26777	367.63752	6
9	958.39912	479.70320	986.39404	493.70066	Y-dichlorin...	637.21501	319.11114	5
10	1059.44680	530.22704	1087.44171	544.22450	T	406.22962	203.61845	4
11	1160.49448	580.75088	1188.48939	594.74833	T	305.18195	153.09461	3
12	1217.51594	609.26161	1245.51086	623.25907	G	204.13427	102.57077	2
13					K	147.11280	74.06004	1

LPGGYGLPYTTGKLPYGYGPGGVAGAA GK

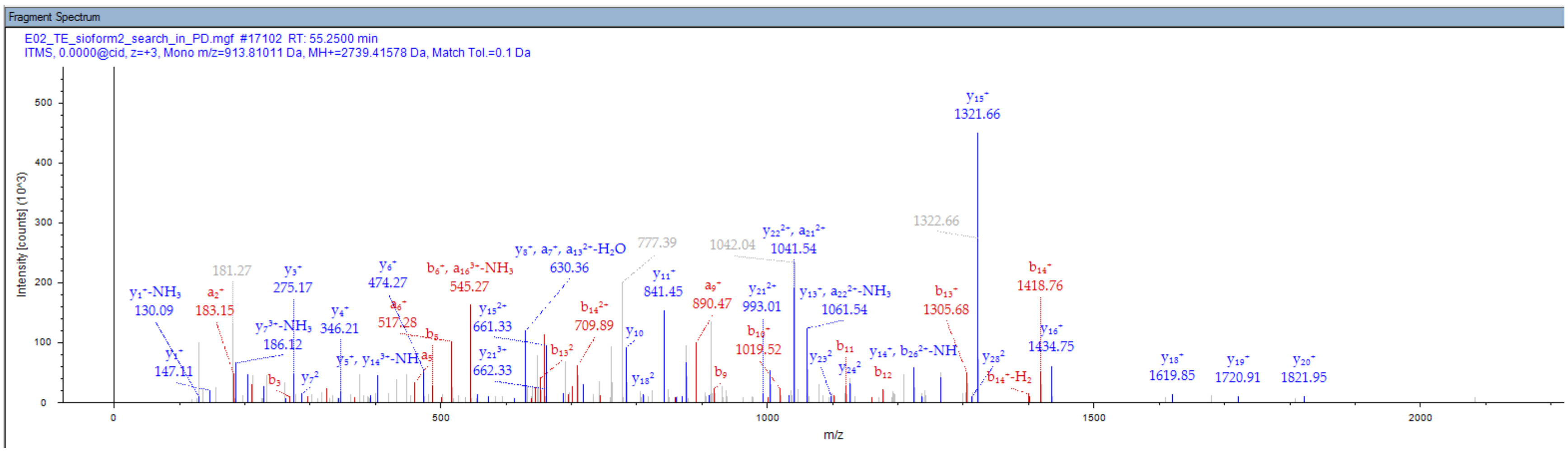

Ion Series | Neutral Losses | Precursor Ions | Internal Fragments

#1	a⁺	a²⁺	a³⁺	b⁺	b²⁺	b³⁺	Seq.	y⁺	y²⁺	y³⁺	#2
1	86.09643	43.55185	29.37033	114.09134	57.54931	38.70196	L				29
2	183.14919	92.07823	61.72125	211.14410	106.07569	71.05289	P	2626.33001	1313.66864	876.11486	28
3	240.17065	120.58897	80.72840	268.16557	134.58642	90.06004	G	2529.27725	1265.14226	843.76393	27
4	297.19212	149.09970	99.73556	325.18703	163.09715	109.06720	G	2472.25579	1236.63153	824.75678	26
5	460.25545	230.63136	154.09000	488.25036	244.62882	163.42164	Y	2415.23432	1208.12080	805.74963	25
6	517.27691	259.14209	173.09715	545.27182	273.13955	182.42879	G	2252.17099	1126.58914	751.39518	24
7	630.36097	315.68412	210.79184	658.35589	329.68158	220.12348	L	2195.14953	1098.07840	732.38803	23
8	727.41374	364.21051	243.14276	755.40865	378.20796	252.47440	P	2082.06547	1041.53637	694.69334	22
9	890.47707	445.74217	297.49721	918.47198	459.73963	306.82884	Y	1985.01270	993.00999	662.34242	21
10	991.52474	496.26601	331.17977	1019.51966	510.26347	340.51140	T	1821.94937	911.47833	607.98798	20
11	1092.57242	546.78985	364.86233	1120.56734	560.78731	374.19396	T	1720.90170	860.95449	574.30542	19
12	1149.59389	575.30058	383.86948	1177.58880	589.29804	393.20112	G	1619.85402	810.43065	540.62286	18
13	1277.68885	639.34806	426.56780	1305.68376	653.34552	435.89944	K	1562.83255	781.91991	521.61570	17
14	1390.77291	695.89009	464.26249	1418.76783	709.88755	473.59413	L	1434.73759	717.87243	478.91738	16
15	1487.82568	744.41648	496.61341	1515.82059	758.41393	505.94505	P	1321.65353	661.33040	441.22269	15
16	1650.88901	825.94814	550.96785	1678.88392	839.94560	560.29949	Y	1224.60076	612.80402	408.87177	14
17	1707.91047	854.45887	569.97501	1735.90538	868.45633	579.30665	G	1061.53743	531.27236	354.51733	13
18	1870.97380	935.99054	624.32945	1898.96871	949.98799	633.66109	Y	1004.51597	502.76162	335.51017	12
19	1927.99526	964.50127	643.33660	1955.99018	978.49873	652.66824	G	841.45264	421.22996	281.15573	11
20	2025.04802	1013.02765	675.68753	2053.04294	1027.02511	685.01916	P	784.43118	392.71923	262.14858	10
21	2082.06949	1041.53838	694.69468	2110.06440	1055.53584	704.02632	G	687.37841	344.19285	229.79766	9
22	2139.09095	1070.04911	713.70184	2167.08587	1084.04657	723.03347	G	630.35695	315.68211	210.79050	8
23	2238.15937	1119.58332	746.72464	2266.15428	1133.58078	756.05628	V	573.33549	287.17138	191.78335	7
24	2309.19648	1155.10188	770.40368	2337.19139	1169.09934	779.73532	A	474.26707	237.63717	158.76054	6
25	2366.21794	1183.61261	789.41083	2394.21286	1197.61007	798.74247	G	403.22996	202.11862	135.08150	5
26	2437.25506	1219.13117	813.08987	2465.24997	1233.12862	822.42151	A	346.20850	173.60789	116.07435	4
27	2508.29217	1254.64972	836.76891	2536.28709	1268.64718	846.10055	A	275.17138	138.08933	92.39531	3
28	2565.31363	1283.16046	855.77606	2593.30855	1297.15791	865.10770	G	204.13427	102.57077	68.71627	2
29							K	147.11280	74.06004	49.70912	1

LPGGYGLPYTTGKLPYGYGPGGVAGAAGK, Y16-dichlorination (67.92206 Da)

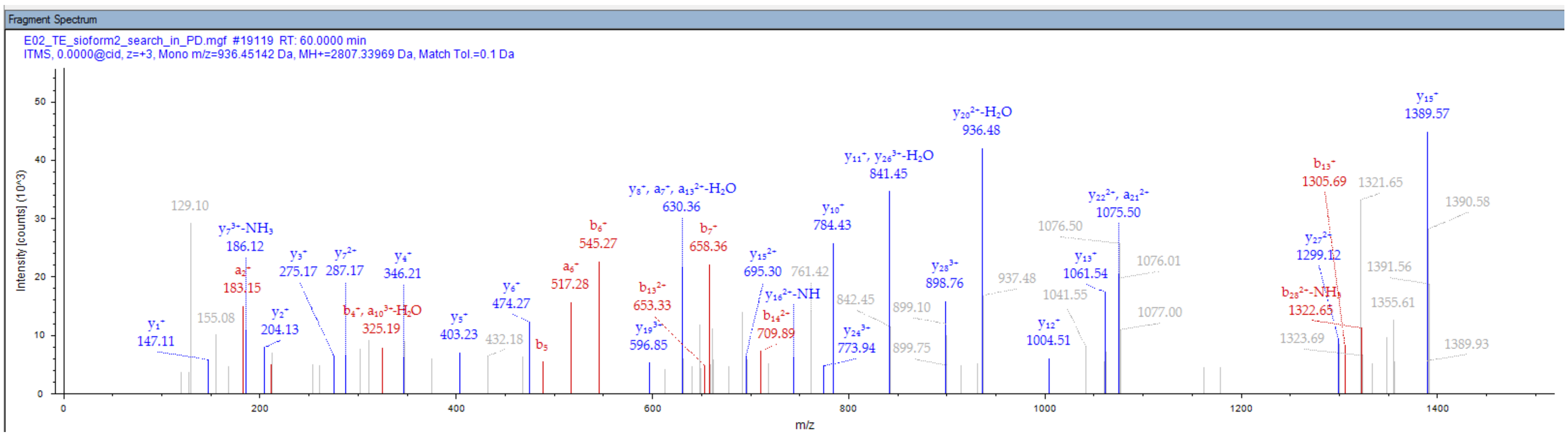

Ion Series | Neutral Losses | Precursor Ions | Internal Fragments

#1	a^+	a^{2+}	a^{3+}	b^+	b^{2+}	b^{3+}	Seq.	y^+	y^{2+}	y^{3+}	#2
1	86.09643	43.55185	29.37033	114.09134	57.54931	38.70196	L				29
2	183.14919	92.07823	61.72125	211.14410	106.07569	71.05289	P	2694.25207	1347.62967	898.75554	28
3	240.17065	120.58897	80.72840	268.16557	134.58642	90.06004	G	2597.19930	1299.10329	866.40462	27
4	297.19212	149.09970	99.73556	325.18703	163.09715	109.06720	G	2540.17784	1270.59256	847.39746	26
5	460.25545	230.63136	154.09000	488.25036	244.62882	163.42164	Y	2483.15638	1242.08183	828.39031	25
6	517.27691	259.14209	173.09715	545.27182	273.13955	182.42879	G	2320.09305	1160.55016	774.03587	24
7	630.36097	315.68412	210.79184	658.35589	329.68158	220.12348	L	2263.07158	1132.03943	755.02871	23
8	727.41374	364.21051	243.14276	755.40865	378.20796	252.47440	P	2149.98752	1075.49740	717.33402	22
9	890.47707	445.74217	297.49721	918.47198	459.73963	306.82884	Y	2052.93476	1026.97102	684.98310	21
10	991.52474	496.26601	331.17977	1019.51966	510.26347	340.51140	T	1889.87143	945.43935	630.62866	20
11	1092.57242	546.78985	364.86233	1120.56734	560.78731	374.19396	T	1788.82375	894.91551	596.94610	19
12	1149.59389	575.30058	383.86948	1177.58880	589.29804	393.20112	G	1687.77607	844.39167	563.26354	18
13	1277.68885	639.34806	426.56780	1305.68376	653.34552	435.89944	K	1630.75461	815.88094	544.25639	17
14	1390.77291	695.89009	464.26249	1418.76783	709.88755	473.59413	L	1502.65965	751.83346	501.55807	16
15	1487.82568	744.41648	496.61341	1515.82059	758.41393	505.94505	P	1389.57558	695.29143	463.86338	15
16	1718.81106	859.90917	573.60854	1746.80597	873.90663	582.94018	Y-dichlorin...	1292.52282	646.76505	431.51246	14
17	1775.83252	888.41990	592.61569	1803.82744	902.41736	601.94733	G	1061.53743	531.27236	354.51733	13
18	1938.89585	969.95156	646.97014	1966.89077	983.94902	656.30177	Y	1004.51597	502.76162	335.51017	12
19	1995.91732	998.46230	665.97729	2023.91223	1012.45975	675.30893	G	841.45264	421.22996	281.15573	11
20	2092.97008	1046.98868	698.32821	2120.96499	1060.98614	707.65985	P	784.43118	392.71923	262.14858	10
21	2149.99154	1075.49941	717.33537	2177.98646	1089.49687	726.66700	G	687.37841	344.19285	229.79766	9
22	2207.01301	1104.01014	736.34252	2235.00792	1118.00760	745.67416	G	630.35695	315.68211	210.79050	8
23	2306.08142	1153.54435	769.36532	2334.07634	1167.54181	778.69696	V	573.33549	287.17138	191.78335	7
24	2377.11853	1189.06291	793.04436	2405.11345	1203.06036	802.37600	A	474.26707	237.63717	158.76054	6
25	2434.14000	1217.57364	812.05152	2462.13491	1231.57109	821.38316	G	403.22996	202.11862	135.08150	5
26	2505.17711	1253.09219	835.73056	2533.17203	1267.08965	845.06219	A	346.20850	173.60789	116.07435	4
27	2576.21423	1288.61075	859.40959	2604.20914	1302.60821	868.74123	A	275.17138	138.08933	92.39531	3
28	2633.23569	1317.12148	878.41675	2661.23060	1331.11894	887.74839	G	204.13427	102.57077	68.71627	2
29							K	147.11280	74.06004	49.70912	1

LPYGYGPGGVAGAAGK

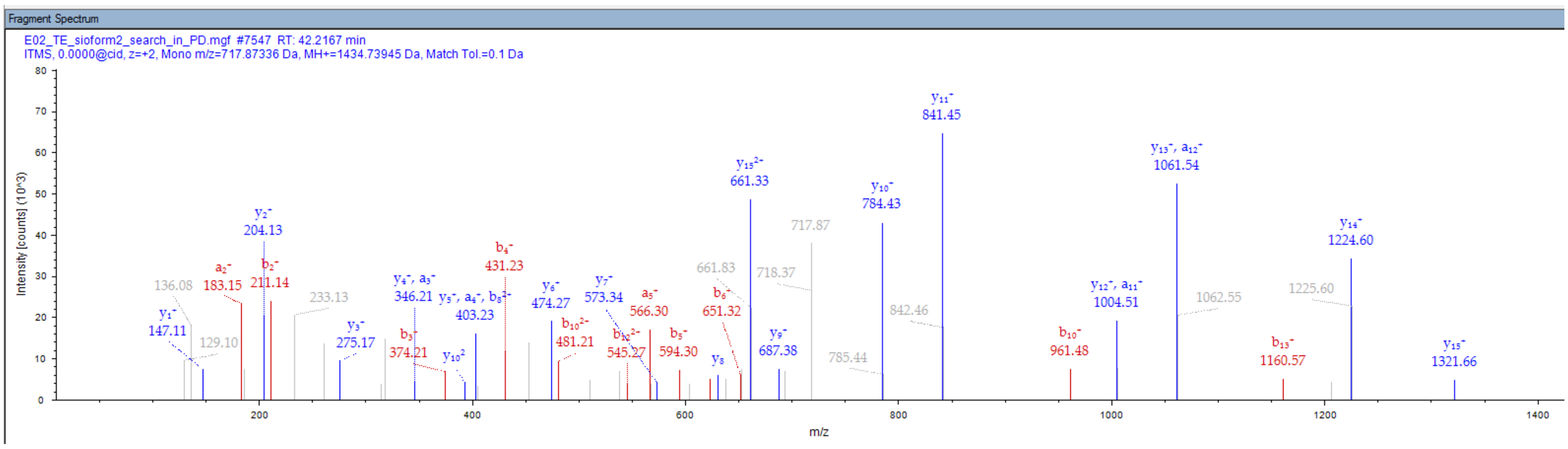

Ion Series | Neutral Losses | Precursor Ions | Internal Fragments

#1	a⁺	a²⁺	b⁺	b²⁺	Seq.	y⁺	y²⁺	#2
1	86.09643	43.55185	114.09134	57.54931	L			16
2	183.14919	92.07823	211.14410	106.07569	P	1321.65353	661.33040	15
3	346.21252	173.60990	374.20743	187.60735	Y	1224.60076	612.80402	14
4	403.23398	202.12063	431.22890	216.11809	G	1061.53743	531.27236	13
5	566.29731	283.65229	594.29223	297.64975	Y	1004.51597	502.76162	12
6	623.31877	312.16303	651.31369	326.16048	G	841.45264	421.22996	11
7	720.37154	360.68941	748.36645	374.68686	P	784.43118	392.71923	10
8	777.39300	389.20014	805.38792	403.19760	G	687.37841	344.19285	9
9	834.41447	417.71087	862.40938	431.70833	G	630.35695	315.68211	8
10	933.48288	467.24508	961.47779	481.24254	V	573.33549	287.17138	7
11	1004.51999	502.76363	1032.51491	516.76109	A	474.26707	237.63717	6
12	1061.54146	531.27437	1089.53637	545.27182	G	403.22996	202.11862	5
13	1132.57857	566.79292	1160.57348	580.79038	A	346.20850	173.60789	4
14	1203.61568	602.31148	1231.61060	616.30894	A	275.17138	138.08933	3
15	1260.63715	630.82221	1288.63206	644.81967	G	204.13427	102.57077	2
16					K	147.11280	74.06004	1

LPYGYGPGGVAGAAGK, Y3-Chlorination (33.96103 Da)

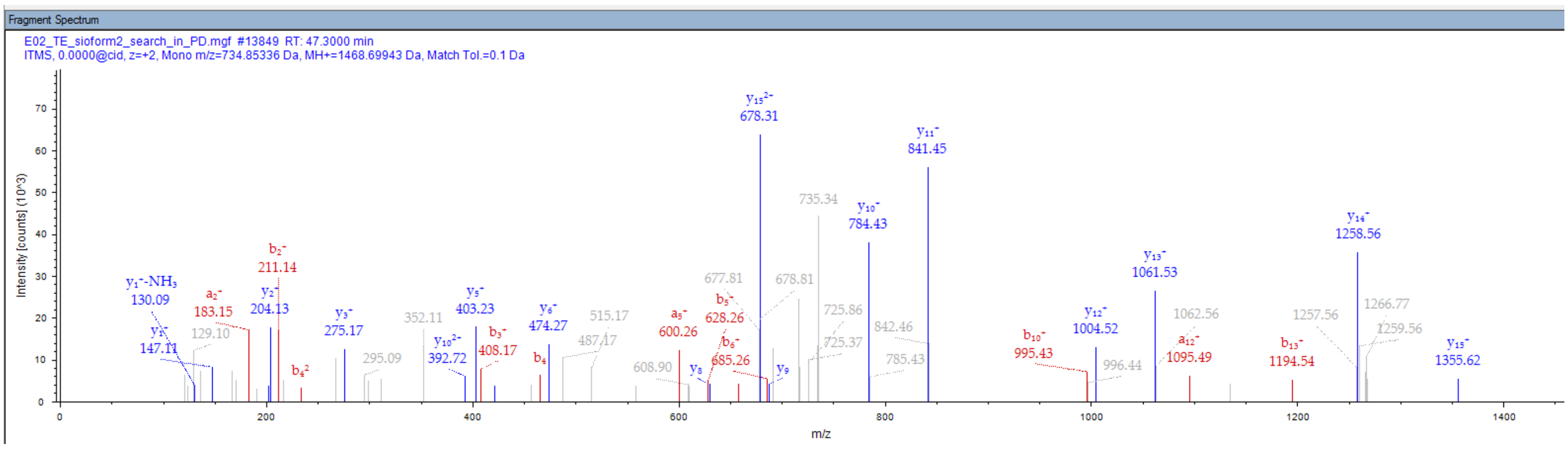

Ion Series	Neutral Losses	Precursor Ions	Internal Fragments

#1	a⁺	a²⁺	b⁺	b²⁺	Seq.	y⁺	y²⁺	#2
1	86.09643	43.55185	114.09134	57.54931	L			16
2	183.14919	92.07823	211.14410	106.07569	P	1355.61455	678.31092	15
3	380.17355	190.59041	408.16846	204.58787	Y-Chlorinat...	1258.56179	629.78453	14
4	437.19501	219.10114	465.18992	233.09860	G	1061.53743	531.27236	13
5	600.25834	300.63281	628.25325	314.63026	Y	1004.51597	502.76162	12
6	657.27980	329.14354	685.27472	343.14100	G	841.45264	421.22996	11
7	754.33257	377.66992	782.32748	391.66738	P	784.43118	392.71923	10
8	811.35403	406.18065	839.34894	420.17811	G	687.37841	344.19285	9
9	868.37549	434.69138	896.37041	448.68884	G	630.35695	315.68211	8
10	967.44391	484.22559	995.43882	498.22305	V	573.33549	287.17138	7
11	1038.48102	519.74415	1066.47594	533.74161	A	474.26707	237.63717	6
12	1095.50248	548.25488	1123.49740	562.25234	G	403.22996	202.11862	5
13	1166.53960	583.77344	1194.53451	597.77089	A	346.20850	173.60789	4
14	1237.57671	619.29199	1265.57163	633.28945	A	275.17138	138.08933	3
15	1294.59818	647.80273	1322.59309	661.80018	G	204.13427	102.57077	2
16					K	147.11280	74.06004	1

LPYGYGPGGVAGAAGK, Y5-Chlorination (33.96103 Da)

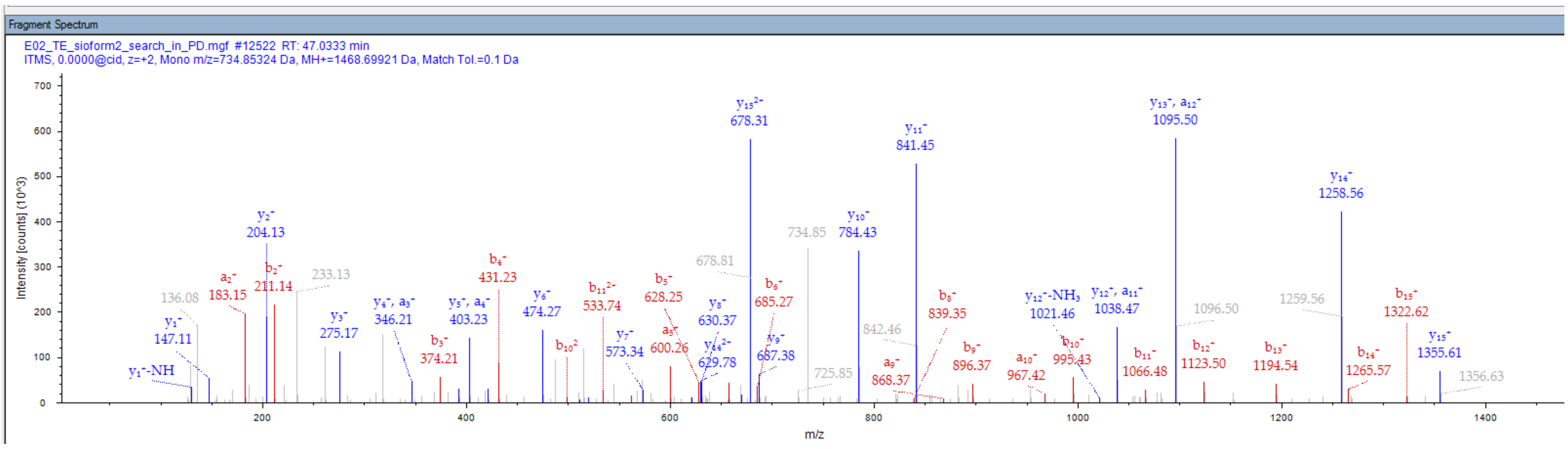

Ion Series | Neutral Losses | Precursor Ions | Internal Fragments

#1	a⁺	a²⁺	b⁺	b²⁺	Seq.	y⁺	y²⁺	#2
1	86.09643	43.55185	114.09134	57.54931	L			16
2	183.14919	92.07823	211.14410	106.07569	P	1355.61455	678.31092	15
3	346.21252	173.60990	374.20743	187.60735	Y	1258.56179	629.78453	14
4	403.23398	202.12063	431.22890	216.11809	G	1095.49846	548.25287	13
5	600.25834	300.63281	628.25325	314.63026	Y-Chlorinat...	1038.47700	519.74214	12
6	657.27980	329.14354	685.27472	343.14100	G	841.45264	421.22996	11
7	754.33257	377.66992	782.32748	391.66738	P	784.43118	392.71923	10
8	811.35403	406.18065	839.34894	420.17811	G	687.37841	344.19285	9
9	868.37549	434.69138	896.37041	448.68884	G	630.35695	315.68211	8
10	967.44391	484.22559	995.43882	498.22305	V	573.33549	287.17138	7
11	1038.48102	519.74415	1066.47594	533.74161	A	474.26707	237.63717	6
12	1095.50248	548.25488	1123.49740	562.25234	G	403.22996	202.11862	5
13	1166.53960	583.77344	1194.53451	597.77089	A	346.20850	173.60789	4
14	1237.57671	619.29199	1265.57163	633.28945	A	275.17138	138.08933	3
15	1294.59818	647.80273	1322.59309	661.80018	G	204.13427	102.57077	2
16					K	147.11280	74.06004	1

LPYGYGPGGVAGAAGK, Y3-dichlorination (67.92206 Da)

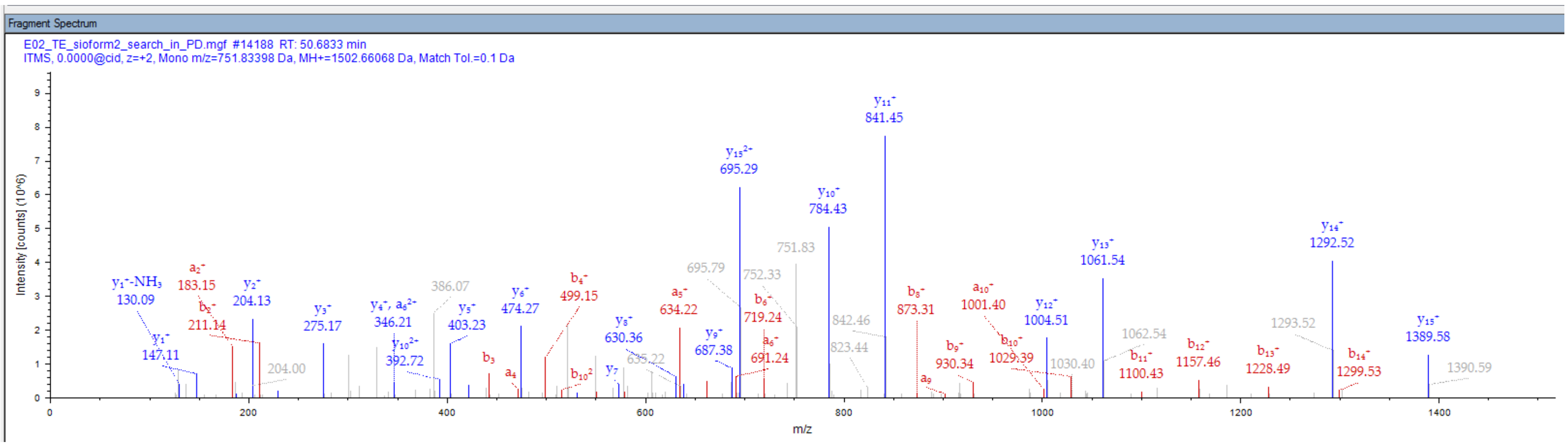

Ion Series | Neutral Losses | Precursor Ions | Internal Fragments

#1	a⁺	a²⁺	b⁺	b²⁺	Seq.	y⁺	y²⁺	#2
1	86.09643	43.55185	114.09134	57.54931	L			16
2	183.14919	92.07823	211.14410	106.07569	P	1389.57558	695.29143	15
3	414.13457	207.57092	442.12949	221.56838	Y-dichlorin...	1292.52282	646.76505	14
4	471.15604	236.08166	499.15095	250.07911	G	1061.53743	531.27236	13
5	634.21937	317.61332	662.21428	331.61078	Y	1004.51597	502.76162	12
6	691.24083	346.12405	719.23574	360.12151	G	841.45264	421.22996	11
7	788.29359	394.65043	816.28851	408.64789	P	784.43118	392.71923	10
8	845.31506	423.16117	873.30997	437.15862	G	687.37841	344.19285	9
9	902.33652	451.67190	930.33143	465.66936	G	630.35695	315.68211	8
10	1001.40493	501.20611	1029.39985	515.20356	V	573.33549	287.17138	7
11	1072.44205	536.72466	1100.43696	550.72212	A	474.26707	237.63717	6
12	1129.46351	565.23539	1157.45843	579.23285	G	403.22996	202.11862	5
13	1200.50063	600.75395	1228.49554	614.75141	A	346.20850	173.60789	4
14	1271.53774	636.27251	1299.53265	650.26997	A	275.17138	138.08933	3
15	1328.55920	664.78324	1356.55412	678.78070	G	204.13427	102.57077	2
16					K	147.11280	74.06004	1

LPYGYGPGGVAGAAGK, Y3-Chlorination (33.96103 Da), Y5-Chlorination (33.96103 Da)

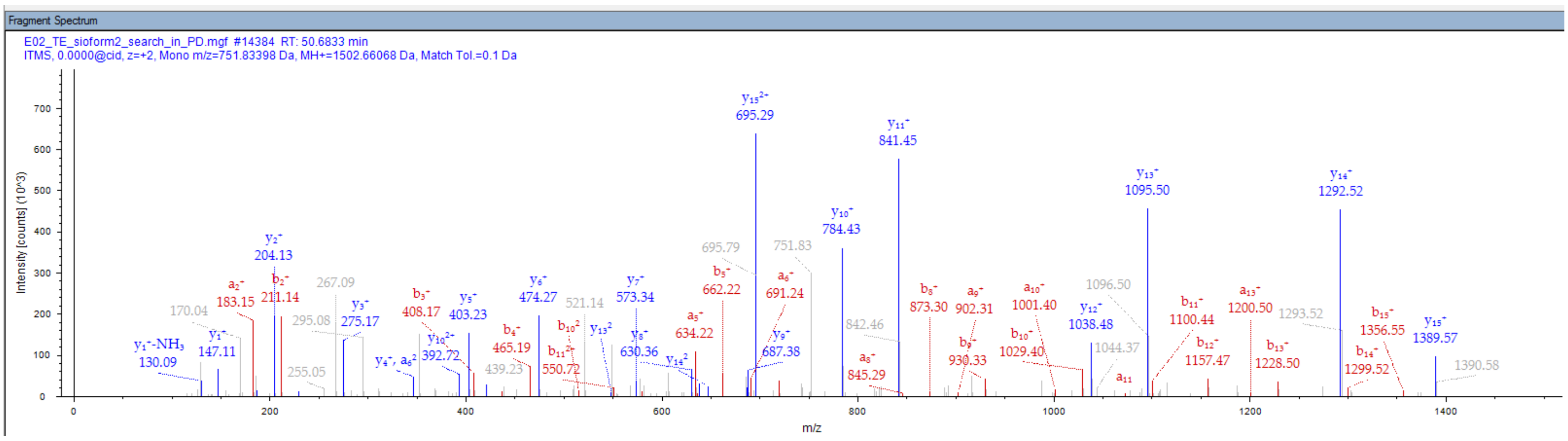

Ion Series | Neutral Losses | Precursor Ions | Internal Fragments

#1	a⁺	a²⁺	b⁺	b²⁺	Seq.	y⁺	y²⁺	#2
1	86.09643	43.55185	114.09134	57.54931	L			16
2	183.14919	92.07823	211.14410	106.07569	P	1389.57558	695.29143	15
3	380.17355	190.59041	408.16846	204.58787	Y-Chlorinat...	1292.52282	646.76505	14
4	437.19501	219.10114	465.18992	233.09860	G	1095.49846	548.25287	13
5	634.21937	317.61332	662.21428	331.61078	Y-Chlorinat...	1038.47700	519.74214	12
6	691.24083	346.12405	719.23574	360.12151	G	841.45264	421.22996	11
7	788.29359	394.65044	816.28851	408.64789	P	784.43118	392.71923	10
8	845.31506	423.16117	873.30997	437.15862	G	687.37841	344.19285	9
9	902.33652	451.67190	930.33144	465.66936	G	630.35695	315.68211	8
10	1001.40494	501.20611	1029.39985	515.20356	V	573.33549	287.17138	7
11	1072.44205	536.72466	1100.43696	550.72212	A	474.26707	237.63717	6
12	1129.46351	565.23539	1157.45843	579.23285	G	403.22996	202.11862	5
13	1200.50063	600.75395	1228.49554	614.75141	A	346.20850	173.60789	4
14	1271.53774	636.27251	1299.53265	650.26997	A	275.17138	138.08933	3
15	1328.55920	664.78324	1356.55412	678.78070	G	204.13427	102.57077	2
16					K	147.11280	74.06004	1

VPGVGLPGVYPGGVLPGAR

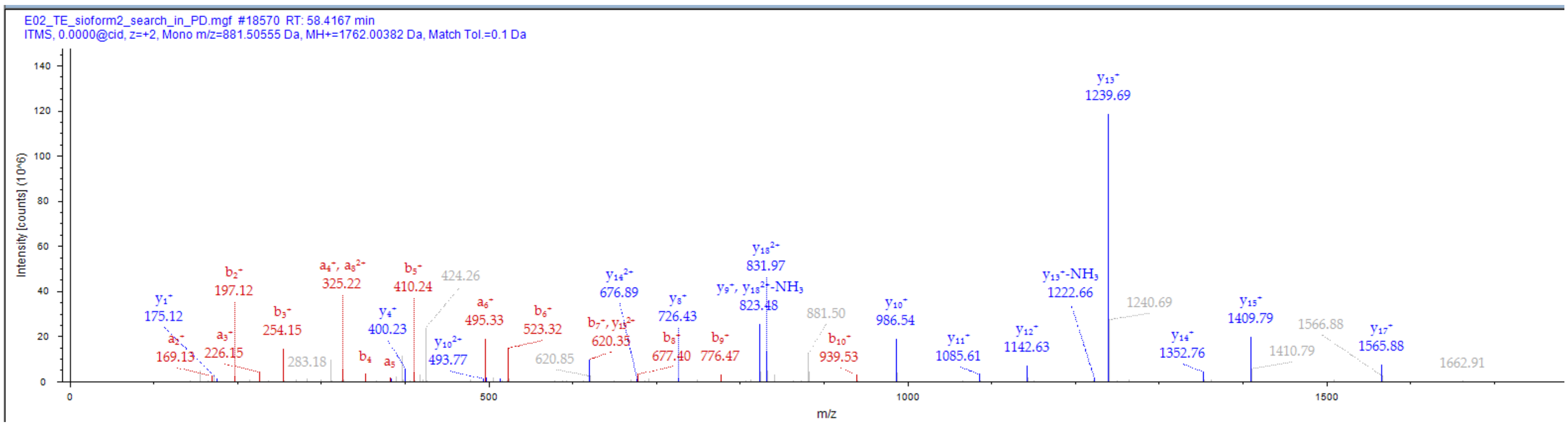

Ion Series | Neutral Losses | Precursor Ions | Internal Fragments

#1	a⁺	a²⁺	b⁺	b²⁺	Seq.	y⁺	y²⁺	#2
1	72.08078	36.54403	100.07569	50.54148	V			19
2	169.13354	85.07041	197.12845	99.06787	P	1662.93260	831.96994	18
3	226.15500	113.58114	254.14992	127.57860	G	1565.87984	783.44356	17
4	325.22342	163.11535	353.21833	177.11280	V	1508.85837	754.93283	16
5	382.24488	191.62608	410.23980	205.62354	G	1409.78996	705.39862	15
6	495.32894	248.16811	523.32386	262.16557	L	1352.76850	676.88789	14
7	592.38171	296.69449	620.37662	310.69195	P	1239.68443	620.34585	13
8	649.40317	325.20522	677.39809	339.20268	G	1142.63167	571.81947	12
9	748.47159	374.73943	776.46650	388.73689	V	1085.61020	543.30874	11
10	911.53491	456.27110	939.52983	470.26855	Y	986.54179	493.77453	10
11	1008.58768	504.79748	1036.58259	518.79493	P	823.47846	412.24287	9
12	1065.60914	533.30821	1093.60406	547.30567	G	726.42570	363.71649	8
13	1122.63061	561.81894	1150.62552	575.81640	G	669.40423	335.20576	7
14	1221.69902	611.35315	1249.69393	625.35061	V	612.38277	306.69502	6
15	1334.78308	667.89518	1362.77800	681.89264	L	513.31436	257.16082	5
16	1431.83585	716.42156	1459.83076	730.41902	P	400.23029	200.61879	4
17	1488.85731	744.93229	1516.85223	758.92975	G	303.17753	152.09240	3
18	1559.89442	780.45085	1587.88934	794.44831	A	246.15607	123.58167	2
19					R	175.11895	88.06311	1

VPGVGLPGVYPGGVLPGAR, Y10-Chlorination (33.96103 Da)

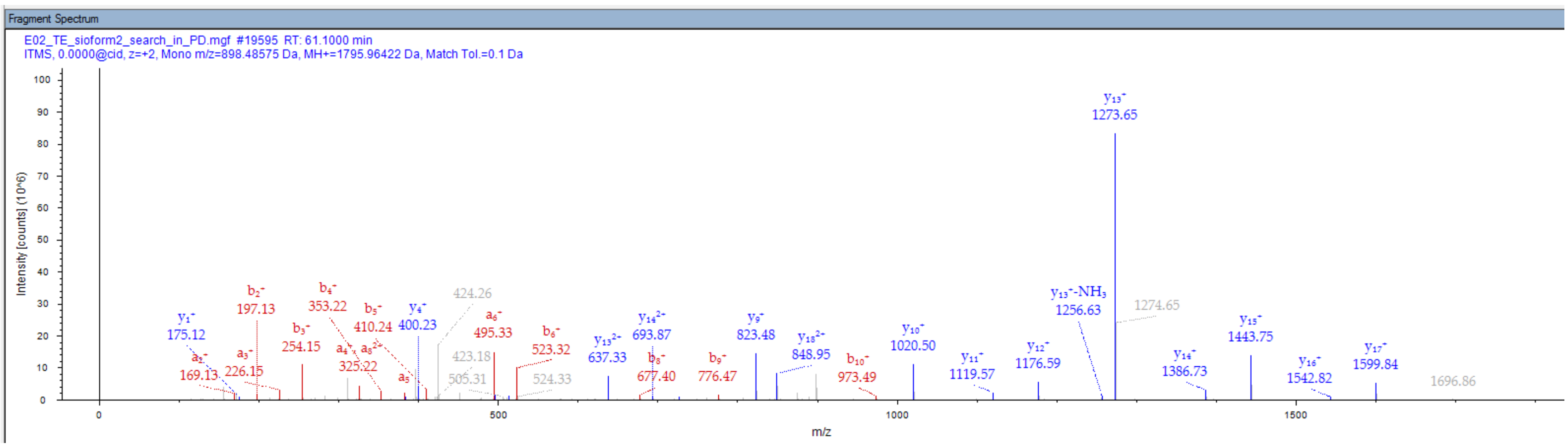

Ion Series | Neutral Losses | Precursor Ions | Internal Fragments

#1	a⁺	a²⁺	b⁺	b²⁺	Seq.	y⁺	y²⁺	#2
1	72.08078	36.54403	100.07569	50.54148	V			19
2	169.13354	85.07041	197.12845	99.06787	P	1696.89363	848.95045	18
3	226.15500	113.58114	254.14992	127.57860	G	1599.84087	800.42407	17
4	325.22342	163.11535	353.21833	177.11280	V	1542.81940	771.91334	16
5	382.24488	191.62608	410.23980	205.62354	G	1443.75099	722.37913	15
6	495.32894	248.16811	523.32386	262.16557	L	1386.72952	693.86840	14
7	592.38171	296.69449	620.37662	310.69195	P	1273.64546	637.32637	13
8	649.40317	325.20522	677.39809	339.20268	G	1176.59270	588.79999	12
9	748.47159	374.73943	776.46650	388.73689	V	1119.57123	560.28925	11
10	945.49594	473.25161	973.49086	487.24907	Y-Chlorinat...	1020.50282	510.75505	10
11	1042.54871	521.77799	1070.54362	535.77545	P	823.47846	412.24287	9
12	1099.57017	550.28872	1127.56508	564.28618	G	726.42570	363.71649	8
13	1156.59163	578.79946	1184.58655	592.79691	G	669.40423	335.20576	7
14	1255.66005	628.33366	1283.65496	642.33112	V	612.38277	306.69502	6
15	1368.74411	684.87569	1396.73903	698.87315	L	513.31436	257.16082	5
16	1465.79688	733.40208	1493.79179	747.39953	P	400.23029	200.61879	4
17	1522.81834	761.91281	1550.81325	775.91027	G	303.17753	152.09240	3
18	1593.85545	797.43136	1621.85037	811.42882	A	246.15607	123.58167	2
19					R	175.11895	88.06311	1

VPGVGLPGVYPGGVLPGAR, Y10-dichlorination (67.92206 Da)

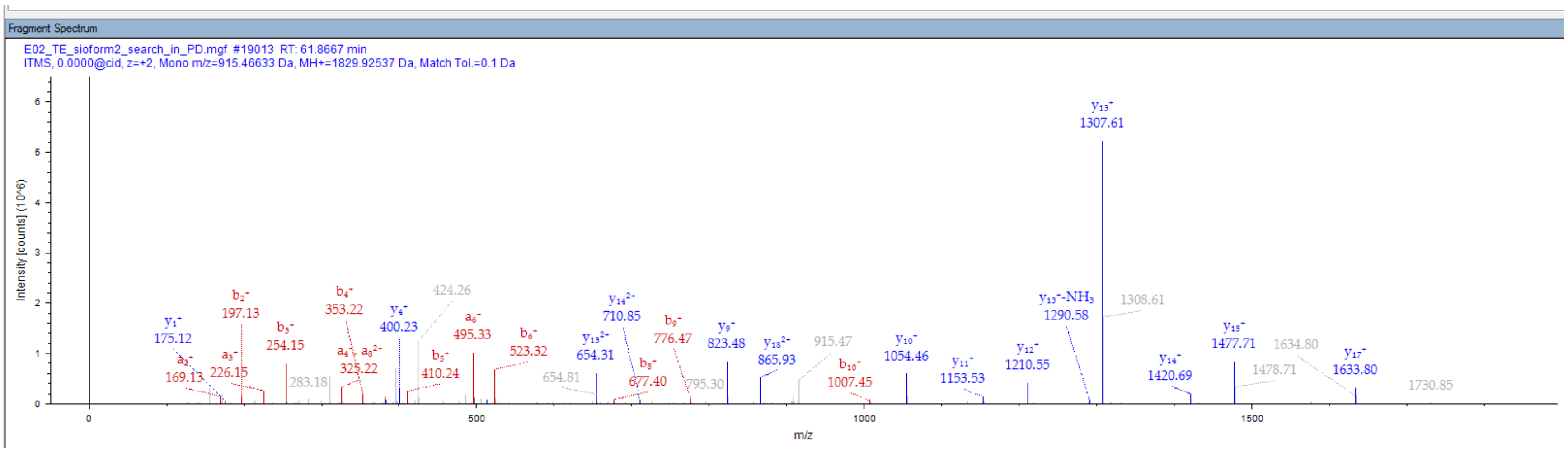

#1	a⁺	a²⁺	b⁺	b²⁺	Seq.	y⁺	y²⁺	#2
1	72.08078	36.54403	100.07569	50.54148	V			19
2	169.13354	85.07041	197.12845	99.06787	P	1730.85466	865.93097	18
3	226.15500	113.58114	254.14992	127.57860	G	1633.80189	817.40458	17
4	325.22342	163.11535	353.21833	177.11280	V	1576.78043	788.89385	16
5	382.24488	191.62608	410.23980	205.62354	G	1477.71201	739.35965	15
6	495.32894	248.16811	523.32386	262.16557	L	1420.69055	710.84891	14
7	592.38171	296.69449	620.37662	310.69195	P	1307.60649	654.30688	13
8	649.40317	325.20522	677.39809	339.20268	G	1210.55372	605.78050	12
9	748.47159	374.73943	776.46650	388.73689	V	1153.53226	577.26977	11
10	979.45697	490.23212	1007.45188	504.22958	Y-dichlorin...	1054.46385	527.73556	10
11	1076.50973	538.75851	1104.50465	552.75596	P	823.47846	412.24287	9
12	1133.53120	567.26924	1161.52611	581.26669	G	726.42570	363.71649	8
13	1190.55266	595.77997	1218.54758	609.77743	G	669.40423	335.20576	7
14	1289.62107	645.31418	1317.61599	659.31163	V	612.38277	306.69502	6
15	1402.70514	701.85621	1430.70005	715.85366	L	513.31436	257.16082	5
16	1499.75790	750.38259	1527.75282	764.38005	P	400.23029	200.61879	4
17	1556.77937	778.89332	1584.77428	792.89078	G	303.17753	152.09240	3
18	1627.81648	814.41188	1655.81139	828.40934	A	246.15607	123.58167	2
19					R	175.11895	88.06311	1

YGAAGLGGVLGGAGQFPLGGVAARPGFGLS PIFPGGACLGKACGR, Q15-Deamidated (0.98402 Da), C43-Oxidation (15.99492 Da)

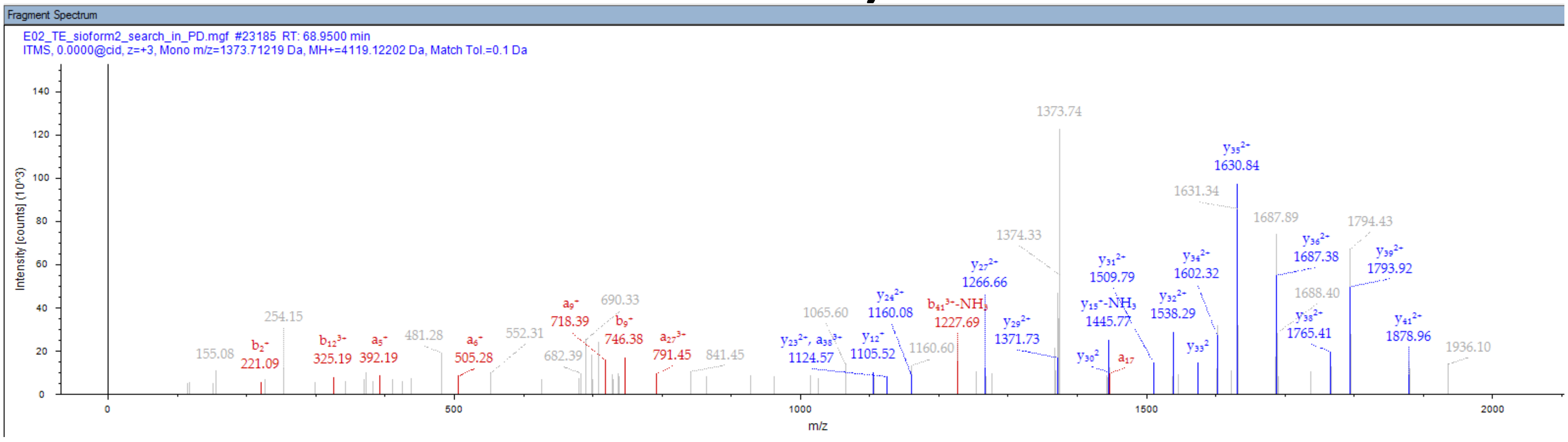

Ion Series | Neutral Losses | Precursor Ions | Internal Fragments

#1	a^+	a^{2+}	a^{3+}	b^+	b^{2+}	b^{3+}	Seq.	y^+	y^{2+}	y^{3+}	#2
1	136.07569	68.54148	46.03008	164.07061	82.53894	55.36172	Y				45
2	193.09715	97.05222	65.03724	221.09207	111.04967	74.36887	G	3956.04219	1978.52473	1319.35225	44
3	264.13427	132.57077	88.71627	292.12918	146.56823	98.04791	A	3899.02073	1950.01400	1300.34509	43
4	335.17138	168.08933	112.39531	363.16630	182.08679	121.72695	A	3827.98361	1914.49545	1276.66606	42
5	392.19285	196.60006	131.40247	420.18776	210.59752	140.73410	G	3756.94650	1878.97689	1252.98702	41
6	505.27691	253.14209	169.09715	533.27182	267.13955	178.42879	L	3699.92504	1850.46616	1233.97986	40
7	562.29837	281.65282	188.10431	590.29329	295.65028	197.43595	G	3586.84097	1793.92412	1196.28518	39
8	619.31984	310.16356	207.11146	647.31475	324.16101	216.44310	G	3529.81951	1765.41339	1177.27802	38
9	718.38825	359.69776	240.13427	746.38317	373.69522	249.46591	V	3472.79805	1736.90266	1158.27087	37
10	831.47231	416.23980	277.82896	859.46723	430.23725	287.16059	L	3373.72963	1687.36845	1125.24806	36
11	888.49378	444.75053	296.83611	916.48869	458.74798	306.16775	G	3260.64557	1630.82642	1087.55337	35
12	945.51524	473.26126	315.84327	973.51016	487.25872	325.17490	G	3203.62410	1602.31569	1068.54622	34
13	1016.55236	508.77982	339.52230	1044.54727	522.77727	348.85394	A	3146.60264	1573.80496	1049.53906	33
14	1073.57382	537.29055	358.52946	1101.56873	551.28801	367.86110	G	3075.56553	1538.28640	1025.86003	32
15	1202.61641	601.81184	401.54366	1230.61133	615.80930	410.87529	Q-Deamid...	3018.54406	1509.77567	1006.85287	31
16	1349.68483	675.34605	450.56646	1377.67974	689.34351	459.89810	F	2889.50147	1445.25437	963.83867	30
17	1446.73759	723.87243	482.91738	1474.73251	737.86989	492.24902	P	2742.43306	1371.72017	914.81587	29
18	1559.82165	780.41447	520.61207	1587.81657	794.41192	529.94371	L	2645.38029	1323.19378	882.46495	28
19	1616.84312	808.92520	539.61922	1644.83803	822.92265	548.95086	G	2532.29623	1266.65175	844.77026	27
20	1673.86458	837.43593	558.62638	1701.85950	851.43339	567.95802	G	2475.27476	1238.14102	825.76311	26
21	1772.93300	886.97014	591.64918	1800.92791	900.96759	600.98082	V	2418.25330	1209.63029	806.75595	25
22	1843.97011	922.48869	615.32822	1871.96502	936.48615	624.65986	A	2319.18489	1160.09608	773.73315	24
23	1915.00722	958.00725	639.00726	1943.00214	972.00471	648.33890	A	2248.14777	1124.57752	750.05411	23
24	2071.10833	1036.05781	691.04096	2099.10325	1050.05526	700.37260	R	2177.11066	1089.05897	726.37507	22
25	2168.16110	1084.58419	723.39188	2196.15601	1098.58164	732.72352	P	2021.00955	1011.00841	674.34137	21
26	2225.18256	1113.09492	742.39904	2253.17748	1127.09238	751.73068	G	1923.95678	962.48203	641.99045	20
27	2372.25098	1186.62913	791.42184	2400.24589	1200.62658	800.75348	F	1866.93532	933.97130	622.98329	19
28	2429.27244	1215.13986	810.42900	2457.26735	1229.13732	819.76064	G	1719.86691	860.43709	573.96049	18
29	2542.35650	1271.68189	848.12369	2570.35142	1285.67935	857.45532	L	1662.84544	831.92636	554.95333	17
30	2629.38853	1315.19790	877.13436	2657.38345	1329.19536	886.46600	S	1549.76138	775.38433	517.25864	16
31	2726.44130	1363.72429	909.48528	2754.43621	1377.72174	918.81692	P	1462.72935	731.86831	488.24797	15
32	2839.52536	1420.26632	947.17997	2867.52027	1434.26378	956.51161	I	1365.67659	683.34193	455.89705	14
33	2986.59377	1493.80052	996.20278	3014.58869	1507.79798	1005.53441	F	1252.59252	626.79990	418.20236	13
34	3083.64654	1542.32691	1028.55370	3111.64145	1556.32436	1037.88533	P	1105.52411	553.26569	369.17955	12
35	3140.66800	1570.83764	1047.56085	3168.66292	1584.83510	1056.89249	G	1008.47135	504.73931	336.82863	11
36	3197.68946	1599.34837	1066.56801	3225.68438	1613.34583	1075.89964	G	951.44988	476.22858	317.82148	10
37	3268.72658	1634.86693	1090.24704	3296.72149	1648.86438	1099.57868	A	894.42842	447.71785	298.81432	9
38	3371.73576	1686.37152	1124.58344	3399.73068	1700.36898	1133.91508	C	823.39130	412.19929	275.13529	8
39	3484.81983	1742.91355	1162.27813	3512.81474	1756.91101	1171.60976	L	720.38212	360.69470	240.79889	7
40	3541.84129	1771.42428	1181.28528	3569.83620	1785.42174	1190.61692	G	607.29806	304.15267	203.10420	6
41	3669.93625	1835.47176	1223.98360	3697.93117	1849.46922	1233.31524	K	550.27659	275.64193	184.09705	5
42	3740.97337	1870.99032	1247.66264	3768.96828	1884.98778	1256.99428	A	422.18163	211.59445	141.39873	4
43	3859.97747	1930.49237	1287.33067	3887.97238	1944.48983	1296.66231	C-Oxidation	351.14452	176.07590	117.71969	3
44	3916.99893	1959.00310	1306.33783	3944.99384	1973.00056	1315.66947	G	232.14042	116.57385	78.05166	2
45							R	175.11895	88.06311	59.04450	1

YGAAGLGGVLGGAGQFPLGGVAARPGFGLSPIFPGGACLGKACGR, Q15-Deamidated (0.98402 Da), C38-Oxidation (15.99492 Da)

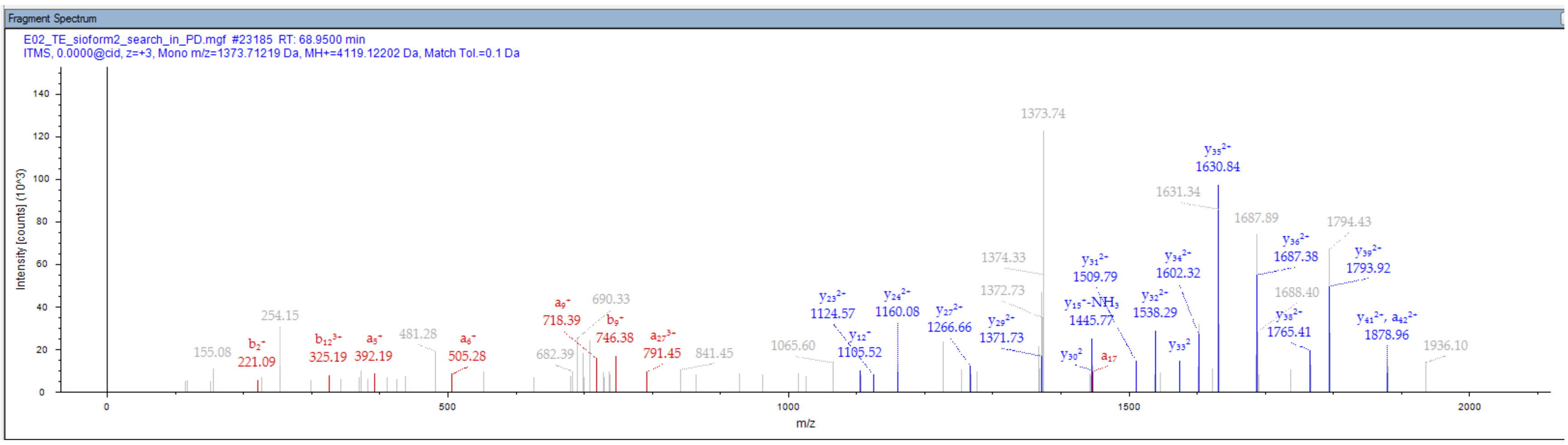

Ion Series | Neutral Losses | Precursor Ions | Internal Fragments

#1	a⁺	a²⁺	a³⁺	b⁺	b²⁺	b³⁺	Seq.	y⁺	y²⁺	y³⁺	#2
1	136.07569	68.54148	46.03008	164.07061	82.53894	55.36172	Y				45
2	193.09715	97.05222	65.03724	221.09207	111.04967	74.36887	G	3956.04219	1978.52473	1319.35225	44
3	264.13427	132.57077	88.71627	292.12918	146.56823	98.04791	A	3899.02073	1950.01400	1300.34509	43
4	335.17138	168.08933	112.39531	363.16630	182.08679	121.72695	A	3827.98361	1914.49545	1276.66606	42
5	392.19285	196.60006	131.40247	420.18776	210.59752	140.73410	G	3756.94650	1878.97689	1252.98702	41
6	505.27691	253.14209	169.09715	533.27182	267.13955	178.42879	L	3699.92504	1850.46616	1233.97986	40
7	562.29837	281.65282	188.10431	590.29329	295.65028	197.43595	G	3586.84097	1793.92412	1196.28518	39
8	619.31984	310.16356	207.11146	647.31475	324.16101	216.44310	G	3529.81951	1765.41339	1177.27802	38
9	718.38825	359.69776	240.13427	746.38317	373.69522	249.46591	V	3472.79805	1736.90266	1158.27087	37
10	831.47231	416.23980	277.82896	859.46723	430.23725	287.16059	L	3373.72963	1687.36845	1125.24806	36
11	888.49378	444.75053	296.83611	916.48869	458.74798	306.16775	G	3260.64557	1630.82642	1087.55337	35
12	945.51524	473.26126	315.84327	973.51016	487.25872	325.17490	G	3203.62410	1602.31569	1068.54622	34
13	1016.55236	508.77982	339.52230	1044.54727	522.77727	348.85394	A	3146.60264	1573.80496	1049.53906	33
14	1073.57382	537.29055	358.52946	1101.56873	551.28801	367.86110	G	3075.56553	1538.28640	1025.86003	32
15	1202.61641	601.81184	401.54366	1230.61133	615.80930	410.87529	Q-Deamid...	3018.54406	1509.77567	1006.85287	31
16	1349.68483	675.34605	450.56646	1377.67974	689.34351	459.89810	F	2889.50147	1445.25437	963.83867	30
17	1446.73759	723.87243	482.91738	1474.73251	737.86989	492.24902	P	2742.43306	1371.72017	914.81587	29
18	1559.82165	780.41447	520.61207	1587.81657	794.41192	529.94371	L	2645.38029	1323.19378	882.46495	28
19	1616.84312	808.92520	539.61922	1644.83803	822.92265	548.95086	G	2532.29623	1266.65175	844.77026	27
20	1673.86458	837.43593	558.62638	1701.85950	851.43339	567.95802	G	2475.27476	1238.14102	825.76311	26
21	1772.93300	886.97014	591.64918	1800.92791	900.96759	600.98082	V	2418.25330	1209.63029	806.75595	25
22	1843.97011	922.48869	615.32822	1871.96502	936.48615	624.65986	A	2319.18489	1160.09608	773.73315	24
23	1915.00722	958.00725	639.00726	1943.00214	972.00471	648.33890	A	2248.14777	1124.57752	750.05411	23
24	2071.10833	1036.05781	691.04096	2099.10325	1050.05526	700.37260	R	2177.11066	1089.05897	726.37507	22
25	2168.16110	1084.58419	723.39188	2196.15601	1098.58164	732.72352	P	2021.00955	1011.00841	674.34137	21
26	2225.18256	1113.09492	742.39904	2253.17748	1127.09238	751.73068	G	1923.95678	962.48203	641.99045	20
27	2372.25098	1186.62913	791.42184	2400.24589	1200.62658	800.75348	F	1866.93532	933.97130	622.98329	19
28	2429.27244	1215.13986	810.42900	2457.26735	1229.13732	819.76064	G	1719.86691	860.43709	573.96049	18
29	2542.35650	1271.68189	848.12369	2570.35142	1285.67935	857.45532	L	1662.84544	831.92636	554.95333	17
30	2629.38853	1315.19790	877.13436	2657.38345	1329.19536	886.46600	S	1549.76138	775.38433	517.25864	16
31	2726.44130	1363.72429	909.48528	2754.43621	1377.72174	918.81692	P	1462.72935	731.86831	488.24797	15
32	2839.52536	1420.26632	947.17997	2867.52027	1434.26378	956.51161	I	1365.67659	683.34193	455.89705	14
33	2986.59377	1493.80052	996.20278	3014.58869	1507.79798	1005.53441	F	1252.59252	626.79990	418.20236	13
34	3083.64654	1542.32691	1028.55370	3111.64145	1556.32436	1037.88533	P	1105.52411	553.26569	369.17955	12
35	3140.66800	1570.83764	1047.56085	3168.66292	1584.83510	1056.89249	G	1008.47135	504.73931	336.82863	11
36	3197.68946	1599.34837	1066.56801	3225.68438	1613.34583	1075.89964	G	951.44988	476.22858	317.82148	10
37	3268.72658	1634.86693	1090.24704	3296.72149	1648.86438	1099.57868	A	894.42842	447.71785	298.81432	9
38	3387.73068	1694.36898	1129.91508	3415.72559	1708.36643	1139.24672	C-Oxidation	823.39130	412.19929	275.13529	8
39	3500.81474	1750.91101	1167.60976	3528.80966	1764.90847	1176.94140	L	704.38720	352.69724	235.46725	7
40	3557.83621	1779.42174	1186.61692	3585.83112	1793.41920	1195.94856	G	591.30314	296.15521	197.77256	6
41	3685.93117	1843.46922	1229.31524	3713.92608	1857.46668	1238.64688	K	534.28168	267.64448	178.76541	5
42	3756.96828	1878.98778	1252.99428	3784.96320	1892.98524	1262.32592	A	406.18671	203.59700	136.06709	4
43	3859.97747	1930.49237	1287.33067	3887.97238	1944.48983	1296.66231	C	335.14960	168.07844	112.38805	3
44	3916.99893	1959.00310	1306.33783	3944.99384	1973.00056	1315.66947	G	232.14042	116.57385	78.05166	2
45							R	175.11895	88.06311	59.04450	1

YGAAGLGGVLGGAGQFPLGGVAARPGFGLSPIFPGGACLGKACGR, C43-Oxidation (15.99492 Da), Y1-Chlorination (33.96103 Da)

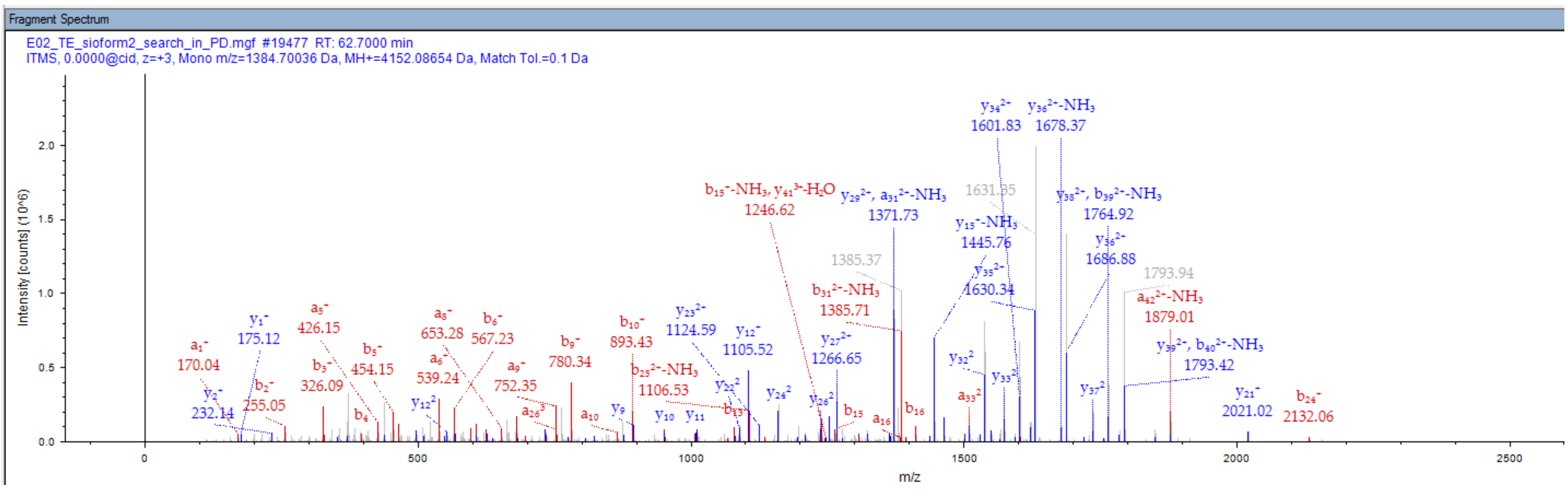

Ion Series	Neutral Losses	Precursor Ions	Internal Fragments

#1	a⁺	a²⁺	a³⁺	b⁺	b²⁺	b³⁺	Seq.	y⁺	y²⁺	y³⁺	#2
1	170.03672	85.52200	57.35042	198.03163	99.51945	66.68206	Y-Chlorinat...				45
2	227.05818	114.03273	76.35758	255.05310	128.03019	85.68922	G	3955.05818	1978.03273	1319.02424	44
3	298.09530	149.55129	100.03662	326.09021	163.54874	109.36825	A	3898.03671	1949.52199	1300.01709	43
4	369.13241	185.06984	123.71565	397.12732	199.06730	133.04729	A	3826.99960	1914.00344	1276.33805	42
5	426.15387	213.58058	142.72281	454.14879	227.57803	152.05445	G	3755.96248	1878.48488	1252.65901	41
6	539.23794	270.12261	180.41750	567.23285	284.12006	189.74914	L	3698.94102	1849.97415	1233.65186	40
7	596.25940	298.63334	199.42465	624.25432	312.63080	208.75629	G	3585.85696	1793.43212	1195.95717	39
8	653.28086	327.14407	218.43181	681.27578	341.14153	227.76344	G	3528.83549	1764.92138	1176.95002	38
9	752.34928	376.67828	251.45461	780.34419	390.67573	260.78625	V	3471.81403	1736.41065	1157.94286	37
10	865.43334	433.22031	289.14930	893.42826	447.21777	298.48094	L	3372.74562	1686.87645	1124.92006	36
11	922.45481	461.73104	308.15645	950.44972	475.72850	317.48809	G	3259.66155	1630.33441	1087.22537	35
12	979.47627	490.24177	327.16361	1007.47118	504.23923	336.49525	G	3202.64009	1601.82368	1068.21821	34
13	1050.51338	525.76033	350.84265	1078.50830	539.75779	360.17428	A	3145.61862	1573.31295	1049.21106	33
14	1107.53485	554.27106	369.84980	1135.52976	568.26852	379.18144	G	3074.58151	1537.79439	1025.53202	32
15	1235.59342	618.30035	412.53599	1263.58834	632.29781	421.86763	Q	3017.56005	1509.28366	1006.52487	31
16	1382.66184	691.83456	461.55880	1410.65675	705.83201	470.89044	F	2889.50147	1445.25437	963.83867	30
17	1479.71460	740.36094	493.90972	1507.70952	754.35840	503.24136	P	2742.43306	1371.72017	914.81587	29
18	1592.79867	796.90297	531.60441	1620.79358	810.90043	540.93604	L	2645.38029	1323.19378	882.46495	28
19	1649.82013	825.41370	550.61156	1677.81504	839.41116	559.94320	G	2532.29623	1266.65175	844.77026	27
20	1706.84159	853.92444	569.61872	1734.83651	867.92189	578.95035	G	2475.27476	1238.14102	825.76311	26
21	1805.91001	903.45864	602.64152	1833.90492	917.45610	611.97316	V	2418.25330	1209.63029	806.75595	25
22	1876.94712	938.97720	626.32056	1904.94204	952.97466	635.65220	A	2319.18489	1160.09608	773.73315	24
23	1947.98424	974.49576	649.99960	1975.97915	988.49321	659.33123	A	2248.14777	1124.57752	750.05411	23
24	2104.08535	1052.54631	702.03330	2132.08026	1066.54377	711.36494	R	2177.11066	1089.05897	726.37507	22
25	2201.13811	1101.07269	734.38422	2229.13302	1115.07015	743.71586	P	2021.00955	1011.00841	674.34137	21
26	2258.15957	1129.58343	753.39138	2286.15449	1143.58088	762.72301	G	1923.95678	962.48203	641.99045	20
27	2405.22799	1203.11763	802.41418	2433.22290	1217.11509	811.74582	F	1866.93532	933.97130	622.98329	19
28	2462.24945	1231.62836	821.42133	2490.24437	1245.62582	830.75297	G	1719.86691	860.43709	573.96049	18
29	2575.33352	1288.17040	859.11602	2603.32843	1302.16785	868.44766	L	1662.84544	831.92636	554.95333	17
30	2662.36554	1331.68641	888.12670	2690.36046	1345.68387	897.45834	S	1549.76138	775.38433	517.25864	16
31	2759.41831	1380.21279	920.47762	2787.41322	1394.21025	929.80926	P	1462.72935	731.86831	488.24797	15
32	2872.50237	1436.75482	958.17231	2900.49729	1450.75228	967.50395	I	1365.67659	683.34193	455.89705	14
33	3019.57079	1510.28903	1007.19511	3047.56570	1524.28649	1016.52675	F	1252.59252	626.79990	418.20236	13
34	3116.62355	1558.81541	1039.54603	3144.61846	1572.81287	1048.87767	P	1105.52411	553.26569	369.17955	12
35	3173.64501	1587.32614	1058.55319	3201.63993	1601.32360	1067.88483	G	1008.47135	504.73931	336.82863	11
36	3230.66648	1615.83688	1077.56034	3258.66139	1629.83433	1086.89198	G	951.44988	476.22858	317.82148	10
37	3301.70359	1651.35543	1101.23938	3329.69850	1665.35289	1110.57102	A	894.42842	447.71785	298.81432	9
38	3404.71277	1702.86003	1135.57578	3432.70769	1716.85748	1144.90741	C	823.39130	412.19929	275.13529	8
39	3517.79684	1759.40206	1173.27046	3545.79175	1773.39951	1182.60210	L	720.38212	360.69470	240.79889	7
40	3574.81830	1787.91279	1192.27762	3602.81322	1801.91025	1201.60926	G	607.29806	304.15267	203.10420	6
41	3702.91326	1851.96027	1234.97594	3730.90818	1865.95773	1244.30758	K	550.27659	275.64193	184.09705	5
42	3773.95038	1887.47883	1258.65498	3801.94529	1901.47628	1267.98662	A	422.18163	211.59445	141.39873	4
43	3892.95448	1946.98088	1298.32301	3920.94939	1960.97833	1307.65465	C-Oxidation	351.14452	176.07590	117.71969	3
44	3949.97594	1975.49161	1317.33017	3977.97086	1989.48907	1326.66180	G	232.14042	116.57385	78.05166	2
45							R	175.11895	88.06311	59.04450	1

YGAAGLGGVLGGAGQFPLGGVAARPGFGLSPIFPGGACLGKACGR, C38-Oxidation (15.99492 Da), Y1-Chlorination (33.96103 Da)

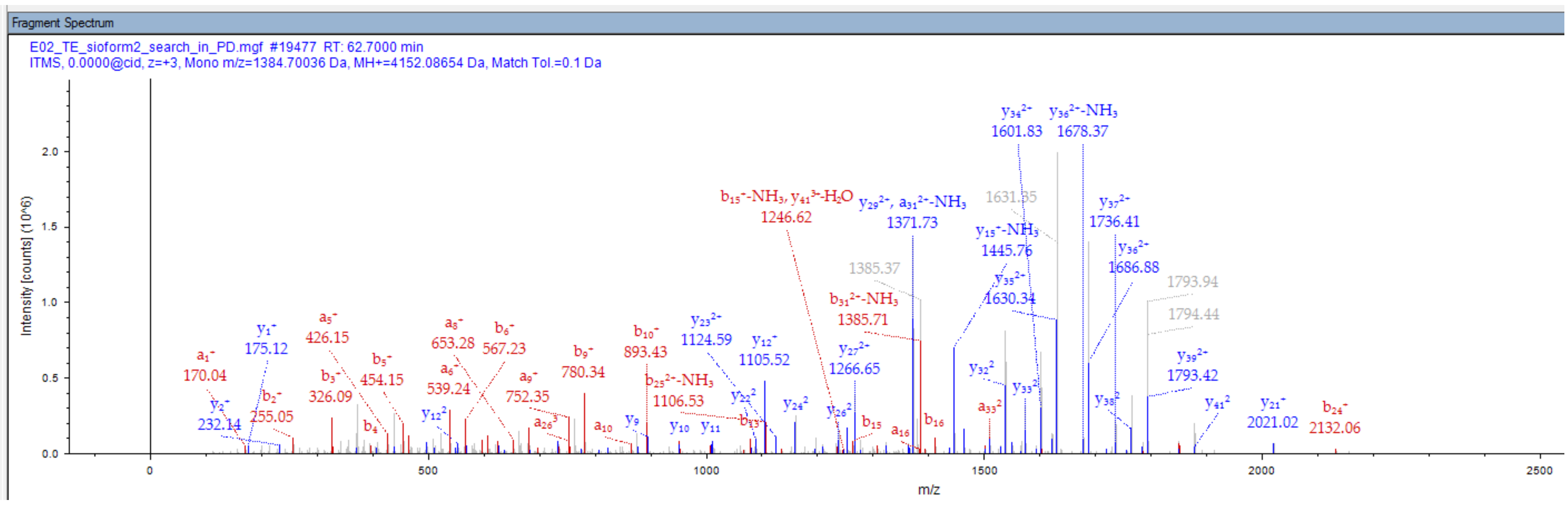

Ion Series | Neutral Losses | Precursor Ions | Internal Fragments

#1	a⁺	a²⁺	a³⁺	b⁺	b²⁺	b³⁺	Seq.	y⁺	y²⁺	y³⁺	#2
1	170.03672	85.52200	57.35042	198.03163	99.51945	66.68206	Y-Chlorinat...				45
2	227.05818	114.03273	76.35758	255.05310	128.03019	85.68922	G	3955.05818	1978.03273	1319.02424	44
3	298.09530	149.55129	100.03662	326.09021	163.54874	109.36825	A	3898.03671	1949.52199	1300.01709	43
4	369.13241	185.06984	123.71565	397.12732	199.06730	133.04729	A	3826.99960	1914.00344	1276.33805	42
5	426.15387	213.58058	142.72281	454.14879	227.57803	152.05445	G	3755.96248	1878.48488	1252.65901	41
6	539.23794	270.12261	180.41750	567.23285	284.12006	189.74914	L	3698.94102	1849.97415	1233.65186	40
7	596.25940	298.63334	199.42465	624.25432	312.63080	208.75629	G	3585.85696	1793.43212	1195.95717	39
8	653.28086	327.14407	218.43181	681.27578	341.14153	227.76344	G	3528.83549	1764.92138	1176.95002	38
9	752.34928	376.67828	251.45461	780.34419	390.67573	260.78625	V	3471.81403	1736.41065	1157.94286	37
10	865.43334	433.22031	289.14930	893.42826	447.21777	298.48094	L	3372.74562	1686.87645	1124.92006	36
11	922.45481	461.73104	308.15645	950.44972	475.72850	317.48809	G	3259.66155	1630.33441	1087.22537	35
12	979.47627	490.24177	327.16361	1007.47118	504.23923	336.49525	G	3202.64009	1601.82368	1068.21821	34
13	1050.51338	525.76033	350.84265	1078.50830	539.75779	360.17428	A	3145.61862	1573.31295	1049.21106	33
14	1107.53485	554.27106	369.84980	1135.52976	568.26852	379.18144	G	3074.58151	1537.79439	1025.53202	32
15	1235.59342	618.30035	412.53599	1263.58834	632.29781	421.86763	Q	3017.56005	1509.28366	1006.52487	31
16	1382.66184	691.83456	461.55880	1410.65675	705.83201	470.89044	F	2889.50147	1445.25437	963.83867	30
17	1479.71460	740.36094	493.90972	1507.70952	754.35840	503.24136	P	2742.43306	1371.72017	914.81587	29
18	1592.79867	796.90297	531.60441	1620.79358	810.90043	540.93604	L	2645.38029	1323.19378	882.46495	28
19	1649.82013	825.41370	550.61156	1677.81504	839.41116	559.94320	G	2532.29623	1266.65175	844.77026	27
20	1706.84159	853.92444	569.61872	1734.83651	867.92189	578.95035	G	2475.27476	1238.14102	825.76311	26
21	1805.91001	903.45864	602.64152	1833.90492	917.45610	611.97316	V	2418.25330	1209.63029	806.75595	25
22	1876.94712	938.97720	626.32056	1904.94204	952.97466	635.65220	A	2319.18489	1160.09608	773.73315	24
23	1947.98424	974.49576	649.99960	1975.97915	988.49321	659.33123	A	2248.14777	1124.57752	750.05411	23
24	2104.08535	1052.54631	702.03330	2132.08026	1066.54377	711.36494	R	2177.11066	1089.05897	726.37507	22
25	2201.13811	1101.07269	734.38422	2229.13302	1115.07015	743.71586	P	2021.00955	1011.00841	674.34137	21
26	2258.15957	1129.58343	753.39138	2286.15449	1143.58088	762.72301	G	1923.95678	962.48203	641.99045	20
27	2405.22799	1203.11763	802.41418	2433.22290	1217.11509	811.74582	F	1866.93532	933.97130	622.98329	19
28	2462.24945	1231.62836	821.42133	2490.24437	1245.62582	830.75297	G	1719.86691	860.43709	573.96049	18
29	2575.33352	1288.17040	859.11602	2603.32843	1302.16785	868.44766	L	1662.84544	831.92636	554.95333	17
30	2662.36554	1331.68641	888.12670	2690.36046	1345.68387	897.45834	S	1549.76138	775.38433	517.25864	16
31	2759.41831	1380.21279	920.47762	2787.41322	1394.21025	929.80926	P	1462.72935	731.86831	488.24797	15
32	2872.50237	1436.75482	958.17231	2900.49729	1450.75228	967.50395	I	1365.67659	683.34193	455.89705	14
33	3019.57079	1510.28903	1007.19511	3047.56570	1524.28649	1016.52675	F	1252.59252	626.79990	418.20236	13
34	3116.62355	1558.81541	1039.54603	3144.61846	1572.81287	1048.87767	P	1105.52411	553.26569	369.17955	12
35	3173.64501	1587.32614	1058.55319	3201.63993	1601.32360	1067.88483	G	1008.47135	504.73931	336.82863	11
36	3230.66648	1615.83688	1077.56034	3258.66139	1629.83433	1086.89198	G	951.44988	476.22858	317.82148	10
37	3301.70359	1651.35543	1101.23938	3329.69850	1665.35289	1110.57102	A	894.42842	447.71785	298.81432	9
38	3420.70769	1710.85748	1140.90741	3448.70260	1724.85494	1150.23905	C-Oxidation	823.39130	412.19929	275.13529	8
39	3533.79175	1767.39951	1178.60210	3561.78667	1781.39697	1187.93374	L	704.38720	352.69724	235.46725	7
40	3590.81322	1795.91025	1197.60926	3618.80813	1809.90770	1206.94089	G	591.30314	296.15521	197.77256	6
41	3718.90818	1859.95773	1240.30758	3746.90309	1873.95519	1249.63922	K	534.28168	267.64448	178.76541	5
42	3789.94529	1895.47629	1263.98662	3817.94021	1909.47374	1273.31825	A	406.18671	203.59700	136.06709	4
43	3892.95448	1946.98088	1298.32301	3920.94939	1960.97833	1307.65465	C	335.14960	168.07844	112.38805	3
44	3949.97594	1975.49161	1317.33017	3977.97086	1989.48907	1326.66180	G	232.14042	116.57385	78.05166	2
45							R	175.11895	88.06311	59.04450	1

YGAAGLGGVLGGAGQFPLGGVAARPGFGLSPIFPGGACLGKACGRK, C43-Oxidation (15.99492 Da), Y1-Chlorination (33.96103 Da)

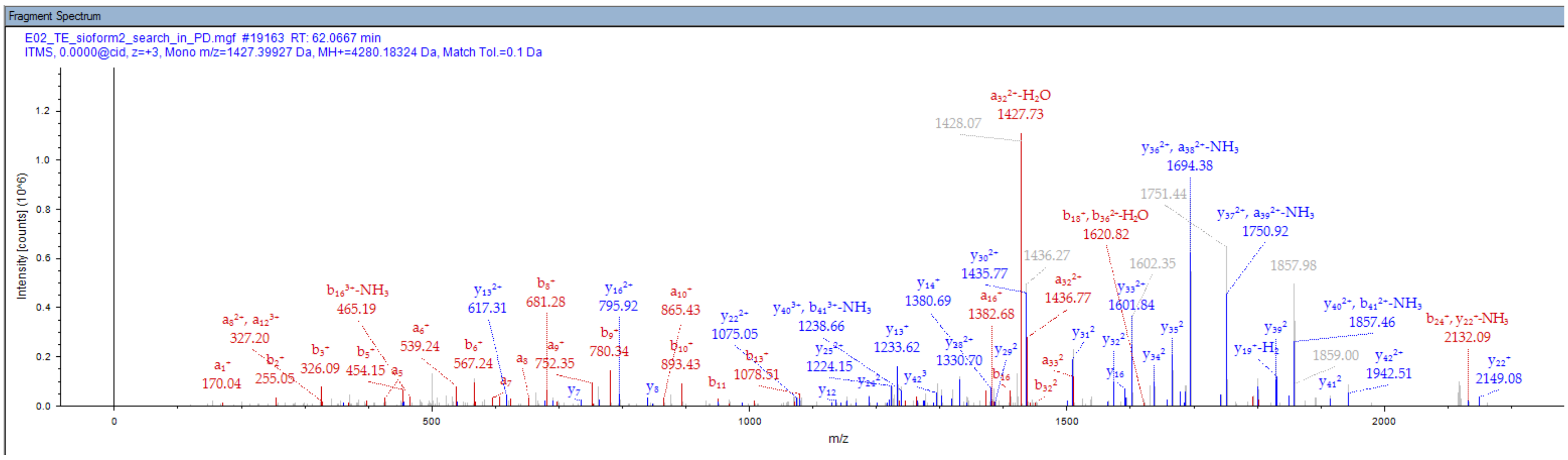

Ion Series | Neutral Losses | Precursor Ions | Internal Fragments

#1	a⁺	a²⁺	a³⁺	b⁺	b²⁺	b³⁺	Seq.	y⁺	y²⁺	y³⁺	#2
1	170.03672	85.52200	57.35042	198.03163	99.51945	66.68206	Y-Chlorinat...				46
2	227.05818	114.03273	76.35758	255.05310	128.03019	85.68922	G	4083.15314	2042.08021	1361.72256	45
3	298.09530	149.55129	100.03662	326.09021	163.54874	109.36825	A	4026.13167	2013.56948	1342.71541	44
4	369.13241	185.06984	123.71565	397.12732	199.06730	133.04729	A	3955.09456	1978.05092	1319.03637	43
5	426.15387	213.58058	142.72281	454.14879	227.57803	152.05445	G	3884.05745	1942.53236	1295.35733	42
6	539.23794	270.12261	180.41750	567.23285	284.12006	189.74914	L	3827.03598	1914.02163	1276.35018	41
7	596.25940	298.63334	199.42465	624.25432	312.63080	208.75629	G	3713.95192	1857.47960	1238.65549	40
8	653.28086	327.14407	218.43181	681.27578	341.14153	227.76344	G	3656.93046	1828.96887	1219.64834	39
9	752.34928	376.67828	251.45461	780.34419	390.67573	260.78625	V	3599.90899	1800.45813	1200.64118	38
10	865.43334	433.22031	289.14930	893.42826	447.21777	298.48094	L	3500.84058	1750.92393	1167.61838	37
11	922.45481	461.73104	308.15645	950.44972	475.72850	317.48809	G	3387.75651	1694.38190	1129.92369	36
12	979.47627	490.24177	327.16361	1007.47118	504.23923	336.49525	G	3330.73505	1665.87116	1110.91653	35
13	1050.51338	525.76033	350.84265	1078.50830	539.75779	360.17428	A	3273.71359	1637.36043	1091.90938	34
14	1107.53485	554.27106	369.84980	1135.52976	568.26852	379.18144	G	3202.67647	1601.84188	1068.23034	33
15	1235.59342	618.30035	412.53599	1263.58834	632.29781	421.86763	Q	3145.65501	1573.33114	1049.22319	32
16	1382.66184	691.83456	461.55880	1410.65675	705.83201	470.89044	F	3017.59643	1509.30185	1006.53700	31
17	1479.71460	740.36094	493.90972	1507.70952	754.35840	503.24136	P	2870.52802	1435.76765	957.51419	30
18	1592.79867	796.90297	531.60441	1620.79358	810.90043	540.93604	L	2773.47525	1387.24127	925.16327	29
19	1649.82013	825.41370	550.61156	1677.81504	839.41116	559.94320	G	2660.39119	1330.69923	887.46858	28
20	1706.84159	853.92444	569.61872	1734.83651	867.92189	578.95035	G	2603.36973	1302.18850	868.46143	27
21	1805.91001	903.45864	602.64152	1833.90492	917.45610	611.97316	V	2546.34826	1273.67777	849.45427	26
22	1876.94712	938.97720	626.32056	1904.94204	952.97466	635.65220	A	2447.27985	1224.14356	816.43147	25
23	1947.98424	974.49576	649.99960	1975.97915	988.49321	659.33123	A	2376.24274	1188.62501	792.75243	24
24	2104.08535	1052.54631	702.03330	2132.08026	1066.54377	711.36494	R	2305.20562	1153.10645	769.07339	23
25	2201.13811	1101.07269	734.38422	2229.13302	1115.07015	743.71586	P	2149.10451	1075.05589	717.03969	22
26	2258.15957	1129.58343	753.39138	2286.15449	1143.58088	762.72301	G	2052.05175	1026.52951	684.68877	21
27	2405.22799	1203.11763	802.41418	2433.22290	1217.11509	811.74582	F	1995.03028	998.01878	665.68161	20
28	2462.24945	1231.62836	821.42133	2490.24437	1245.62582	830.75297	G	1847.96187	924.48457	616.65881	19
29	2575.33352	1288.17040	859.11602	2603.32843	1302.16785	868.44766	L	1790.94041	895.97384	597.65165	18
30	2662.36554	1331.68641	888.12670	2690.36046	1345.68387	897.45834	S	1677.85634	839.43181	559.95697	17
31	2759.41831	1380.21279	920.47762	2787.41322	1394.21025	929.80926	P	1590.82431	795.91580	530.94629	16
32	2872.50237	1436.75482	958.17231	2900.49729	1450.75228	967.50395	I	1493.77155	747.38941	498.59537	15
33	3019.57079	1510.28903	1007.19511	3047.56570	1524.28649	1016.52675	F	1380.68749	690.84738	460.90068	14
34	3116.62355	1558.81541	1039.54603	3144.61846	1572.81287	1048.87767	P	1233.61907	617.31317	411.87788	13
35	3173.64501	1587.32614	1058.55319	3201.63993	1601.32360	1067.88483	G	1136.56631	568.78679	379.52695	12
36	3230.66648	1615.83688	1077.56034	3258.66139	1629.83433	1086.89198	G	1079.54484	540.27606	360.51980	11
37	3301.70359	1651.35543	1101.23938	3329.69850	1665.35289	1110.57102	A	1022.52338	511.76533	341.51264	10
38	3404.71277	1702.86003	1135.57578	3432.70769	1716.85748	1144.90741	C	951.48627	476.24677	317.83361	9
39	3517.79684	1759.40206	1173.27046	3545.79175	1773.39951	1182.60210	L	848.47708	424.74218	283.49721	8
40	3574.81830	1787.91279	1192.27762	3602.81322	1801.91025	1201.60926	G	735.39302	368.20015	245.80252	7
41	3702.91326	1851.96027	1234.97594	3730.90818	1865.95773	1244.30758	K	678.37156	339.68942	226.79537	6
42	3773.95038	1887.47883	1258.65498	3801.94529	1901.47628	1267.98662	A	550.27659	275.64193	184.09705	5
43	3892.95448	1946.98088	1298.32301	3920.94939	1960.97833	1307.65465	C-Oxidation	479.23948	240.12338	160.41801	4
44	3949.97594	1975.49161	1317.33017	3977.97086	1989.48907	1326.66180	G	360.23538	180.62133	120.74998	3
45	4106.07705	2053.54216	1369.36387	4134.07197	2067.53962	1378.69551	R	303.21392	152.11060	101.74282	2
46							K	147.11280	74.06004	49.70912	1

YGAAGLGGVLGGAGQFPLGGVAARPGFGLSPIFPGGACLGKACGRK, C38-Oxidation (15.99492 Da), Y1-Chlorination (33.96103 Da)

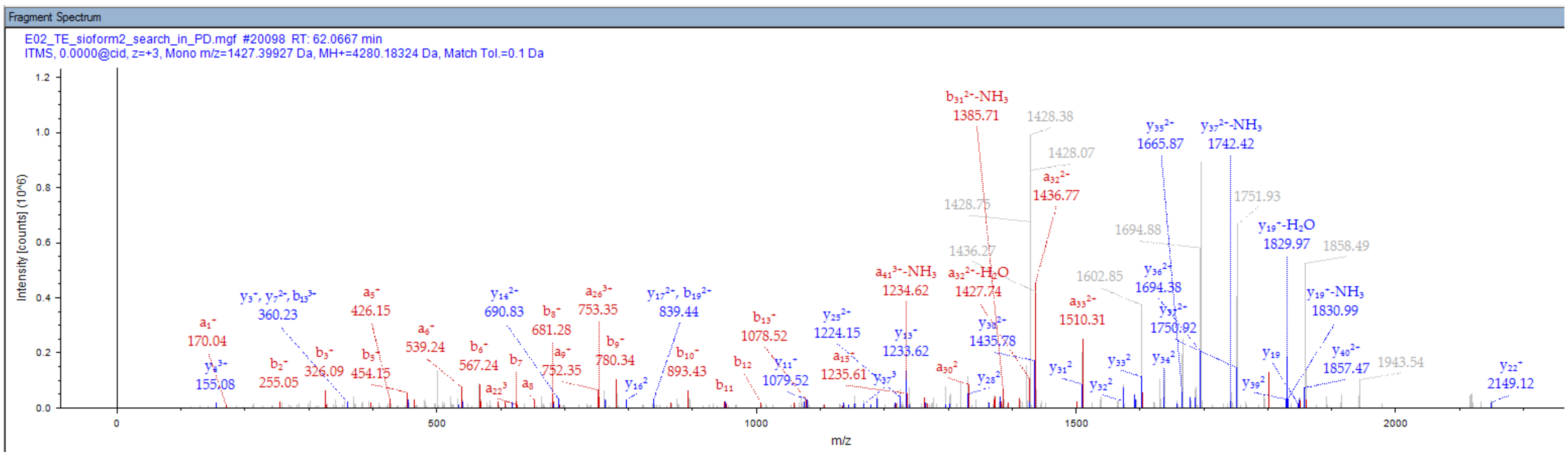

Ion Series	Neutral Losses	Precursor Ions	Internal Fragments

#1	a⁺	a²⁺	a³⁺	b⁺	b²⁺	b³⁺	Seq.	y⁺	y²⁺	y³⁺	#2
1	170.03672	85.52200	57.35042	198.03163	99.51945	66.68206	Y-Chlorinat...				46
2	227.05818	114.03273	76.35758	255.05310	128.03019	85.68922	G	4083.15314	2042.08021	1361.72256	45
3	298.09530	149.55129	100.03662	326.09021	163.54874	109.36825	A	4026.13167	2013.56948	1342.71541	44
4	369.13241	185.06984	123.71565	397.12732	199.06730	133.04729	A	3955.09456	1978.05092	1319.03637	43
5	426.15387	213.58058	142.72281	454.14879	227.57803	152.05445	G	3884.05745	1942.53236	1295.35733	42
6	539.23794	270.12261	180.41750	567.23285	284.12006	189.74914	L	3827.03598	1914.02163	1276.35018	41
7	596.25940	298.63334	199.42465	624.25432	312.63080	208.75629	G	3713.95192	1857.47960	1238.65549	40
8	653.28086	327.14407	218.43181	681.27578	341.14153	227.76344	G	3656.93046	1828.96887	1219.64834	39
9	752.34928	376.67828	251.45461	780.34419	390.67573	260.78625	V	3599.90899	1800.45813	1200.64118	38
10	865.43334	433.22031	289.14930	893.42826	447.21777	298.48094	L	3500.84058	1750.92393	1167.61838	37
11	922.45481	461.73104	308.15645	950.44972	475.72850	317.48809	G	3387.75651	1694.38190	1129.92369	36
12	979.47627	490.24177	327.16361	1007.47118	504.23923	336.49525	G	3330.73505	1665.87116	1110.91653	35
13	1050.51338	525.76033	350.84265	1078.50830	539.75779	360.17428	A	3273.71359	1637.36043	1091.90938	34
14	1107.53485	554.27106	369.84980	1135.52976	568.26852	379.18144	G	3202.67647	1601.84188	1068.23034	33
15	1235.59342	618.30035	412.53599	1263.58834	632.29781	421.86763	Q	3145.65501	1573.33114	1049.22319	32
16	1382.66184	691.83456	461.55880	1410.65675	705.83201	470.89044	F	3017.59643	1509.30185	1006.53700	31
17	1479.71460	740.36094	493.90972	1507.70952	754.35840	503.24136	P	2870.52802	1435.76765	957.51419	30
18	1592.79867	796.90297	531.60441	1620.79358	810.90043	540.93604	L	2773.47525	1387.24127	925.16327	29
19	1649.82013	825.41370	550.61156	1677.81504	839.41116	559.94320	G	2660.39119	1330.69923	887.46858	28
20	1706.84159	853.92444	569.61872	1734.83651	867.92189	578.95035	G	2603.36973	1302.18850	868.46143	27
21	1805.91001	903.45864	602.64152	1833.90492	917.45610	611.97316	V	2546.34826	1273.67777	849.45427	26
22	1876.94712	938.97720	626.32056	1904.94204	952.97466	635.65220	A	2447.27985	1224.14356	816.43147	25
23	1947.98424	974.49576	649.99960	1975.97915	988.49321	659.33123	A	2376.24274	1188.62501	792.75243	24
24	2104.08535	1052.54631	702.03330	2132.08026	1066.54377	711.36494	R	2305.20562	1153.10645	769.07339	23
25	2201.13811	1101.07269	734.38422	2229.13302	1115.07015	743.71586	P	2149.10451	1075.05589	717.03969	22
26	2258.15957	1129.58343	753.39138	2286.15449	1143.58088	762.72301	G	2052.05175	1026.52951	684.68877	21
27	2405.22799	1203.11763	802.41418	2433.22290	1217.11509	811.74582	F	1995.03028	998.01878	665.68161	20
28	2462.24945	1231.62836	821.42133	2490.24437	1245.62582	830.75297	G	1847.96187	924.48457	616.65881	19
29	2575.33352	1288.17040	859.11602	2603.32843	1302.16785	868.44766	L	1790.94041	895.97384	597.65165	18
30	2662.36554	1331.68641	888.12670	2690.36046	1345.68387	897.45834	S	1677.85634	839.43181	559.95697	17
31	2759.41831	1380.21279	920.47762	2787.41322	1394.21025	929.80926	P	1590.82431	795.91580	530.94629	16
32	2872.50237	1436.75482	958.17231	2900.49729	1450.75228	967.50395	I	1493.77155	747.38941	498.59537	15
33	3019.57079	1510.28903	1007.19511	3047.56570	1524.28649	1016.52675	F	1380.68749	690.84738	460.90068	14
34	3116.62355	1558.81541	1039.54603	3144.61846	1572.81287	1048.87767	P	1233.61907	617.31317	411.87788	13
35	3173.64501	1587.32614	1058.55319	3201.63993	1601.32360	1067.88483	G	1136.56631	568.78679	379.52695	12
36	3230.66648	1615.83688	1077.56034	3258.66139	1629.83433	1086.89198	G	1079.54484	540.27606	360.51980	11
37	3301.70359	1651.35543	1101.23938	3329.69850	1665.35289	1110.57102	A	1022.52338	511.76533	341.51264	10
38	3420.70769	1710.85748	1140.90741	3448.70260	1724.85494	1150.23905	C-Oxidation	951.48627	476.24677	317.83361	9
39	3533.79175	1767.39951	1178.60210	3561.78667	1781.39697	1187.93374	L	832.48217	416.74472	278.16557	8
40	3590.81322	1795.91025	1197.60926	3618.80813	1809.90770	1206.94089	G	719.39810	360.20269	240.47089	7
41	3718.90818	1859.95773	1240.30758	3746.90309	1873.95519	1249.63922	K	662.37664	331.69196	221.46373	6
42	3789.94529	1895.47629	1263.98662	3817.94021	1909.47374	1273.31825	A	534.28168	267.64448	178.76541	5
43	3892.95448	1946.98088	1298.32301	3920.94939	1960.97833	1307.65465	C	463.24456	232.12592	155.08637	4
44	3949.97594	1975.49161	1317.33017	3977.97086	1989.48907	1326.66180	G	360.23538	180.62133	120.74998	3
45	4106.07705	2053.54216	1369.36387	4134.07197	2067.53962	1378.69551	R	303.21392	152.11060	101.74282	2
46							K	147.11280	74.06004	49.70912	1

YGAAVPGVLGGLGALGGVGIPGGVVGA GPAAAAAAK

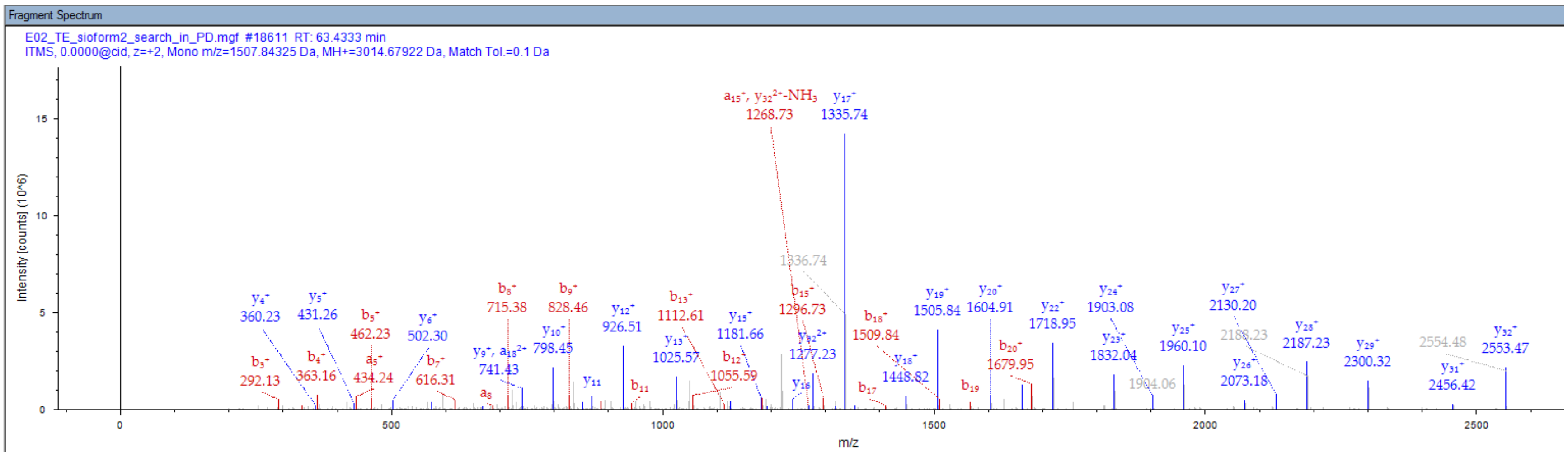

Ion Series | Neutral Losses | Precursor Ions | Internal Fragments

#1	a⁺	a²⁺	b⁺	b²⁺	Seq.	y⁺	y²⁺	#2
1	136.07569	68.54148	164.07061	82.53894	Y			37
2	193.09715	97.05222	221.09207	111.04967	G	2851.61524	1426.31126	36
3	264.13427	132.57077	292.12918	146.56823	A	2794.59377	1397.80052	35
4	335.17138	168.08933	363.16630	182.08679	A	2723.55666	1362.28197	34
5	434.23980	217.62354	462.23471	231.62099	V	2652.51955	1326.76341	33
6	531.29256	266.14992	559.28747	280.14738	P	2553.45113	1277.22920	32
7	588.31402	294.66065	616.30894	308.65811	G	2456.39837	1228.70282	31
8	687.38244	344.19486	715.37735	358.19231	V	2399.37690	1200.19209	30
9	800.46650	400.73689	828.46142	414.73435	L	2300.30849	1150.65788	29
10	857.48796	429.24762	885.48288	443.24508	G	2187.22443	1094.11585	28
11	914.50943	457.75835	942.50434	471.75581	G	2130.20296	1065.60512	27
12	1027.59349	514.30038	1055.58841	528.29784	L	2073.18150	1037.09439	26
13	1084.61496	542.81112	1112.60987	556.80857	G	1960.09743	980.55236	25
14	1155.65207	578.32967	1183.64698	592.32713	A	1903.07597	952.04162	24
15	1268.73613	634.87171	1296.73105	648.86916	L	1832.03886	916.52307	23
16	1325.75760	663.38244	1353.75251	677.37989	G	1718.95479	859.98103	22
17	1382.77906	691.89317	1410.77398	705.89063	G	1661.93333	831.47030	21
18	1481.84747	741.42738	1509.84239	755.42483	V	1604.91187	802.95957	20
19	1538.86894	769.93811	1566.86385	783.93556	G	1505.84345	753.42536	19
20	1651.95300	826.48014	1679.94792	840.47760	I	1448.82199	724.91463	18
21	1749.00577	875.00652	1777.00068	889.00398	P	1335.73792	668.37260	17
22	1806.02723	903.51725	1834.02214	917.51471	G	1238.68516	619.84622	16
23	1863.04869	932.02799	1891.04361	946.02544	G	1181.66370	591.33549	15
24	1962.11711	981.56219	1990.11202	995.55965	V	1124.64223	562.82475	14
25	2061.18552	1031.09640	2089.18044	1045.09386	V	1025.57382	513.29055	13
26	2118.20699	1059.60713	2146.20190	1073.60459	G	926.50541	463.75634	12
27	2189.24410	1095.12569	2217.23901	1109.12315	A	869.48394	435.24561	11
28	2246.26556	1123.63642	2274.26048	1137.63388	G	798.44683	399.72705	10
29	2343.31833	1172.16280	2371.31324	1186.16026	P	741.42536	371.21632	9
30	2414.35544	1207.68136	2442.35035	1221.67882	A	644.37260	322.68994	8
31	2485.39255	1243.19992	2513.38747	1257.19737	A	573.33549	287.17138	7
32	2556.42967	1278.71847	2584.42458	1292.71593	A	502.29837	251.65282	6
33	2627.46678	1314.23703	2655.46170	1328.23449	A	431.26126	216.13427	5
34	2698.50390	1349.75559	2726.49881	1363.75304	A	360.22415	180.61571	4
35	2769.54101	1385.27414	2797.53592	1399.27160	A	289.18703	145.09715	3
36	2840.57812	1420.79270	2868.57304	1434.79016	A	218.14992	109.57860	2
37					K	147.11280	74.06004	1

YGAAVPGVLGGLGALGGVGIPGGVVGAGPAAAAAAK, Y1-Chlorination (33.96103 Da)

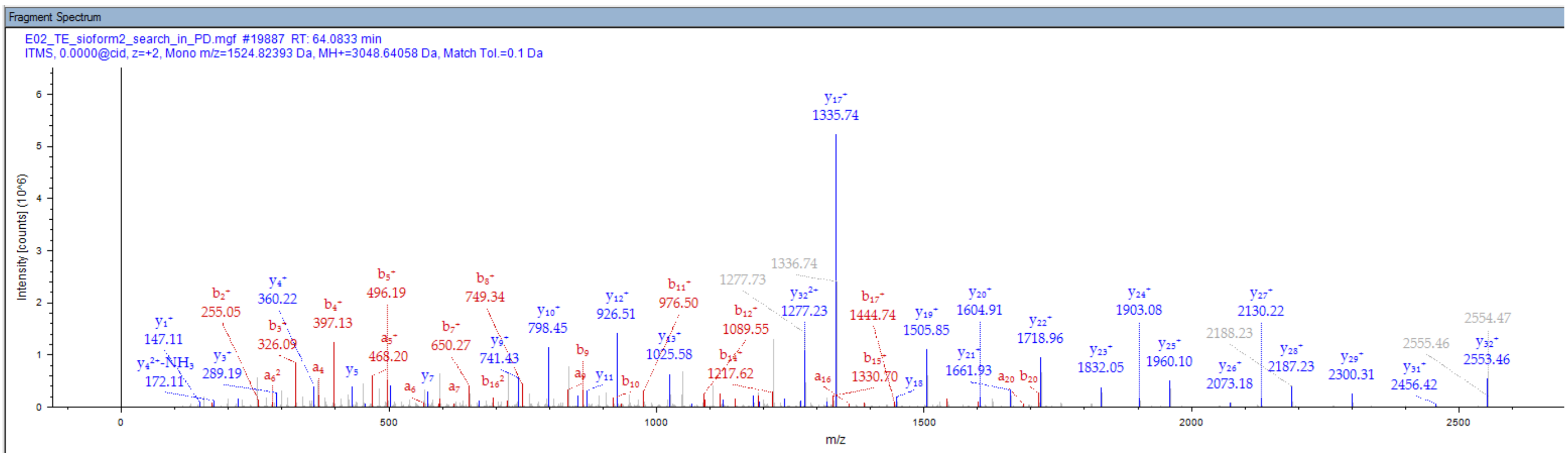

Ion Series | Neutral Losses | Precursor Ions | Internal Fragments

#1	a+	a2+	b+	b2+	Seq.	y+	y2+	#2
1	170.03672	85.52200	198.03163	99.51945	Y-Chlorinat...			37
2	227.05818	114.03273	255.05310	128.03019	G	2851.61524	1426.31126	36
3	298.09530	149.55129	326.09021	163.54874	A	2794.59377	1397.80052	35
4	369.13241	185.06984	397.12732	199.06730	A	2723.55666	1362.28197	34
5	468.20082	234.60405	496.19574	248.60151	V	2652.51955	1326.76341	33
6	565.25359	283.13043	593.24850	297.12789	P	2553.45113	1277.22920	32
7	622.27505	311.64116	650.26997	325.63862	G	2456.39837	1228.70282	31
8	721.34346	361.17537	749.33838	375.17283	V	2399.37690	1200.19209	30
9	834.42753	417.71740	862.42244	431.71486	L	2300.30849	1150.65788	29
10	891.44899	446.22813	919.44391	460.22559	G	2187.22443	1094.11585	28
11	948.47046	474.73887	976.46537	488.73632	G	2130.20296	1065.60512	27
12	1061.55452	531.28090	1089.54943	545.27836	L	2073.18150	1037.09439	26
13	1118.57598	559.79163	1146.57090	573.78909	G	1960.09743	980.55236	25
14	1189.61310	595.31019	1217.60801	609.30764	A	1903.07597	952.04162	24
15	1302.69716	651.85222	1330.69208	665.84968	L	1832.03886	916.52307	23
16	1359.71863	680.36295	1387.71354	694.36041	G	1718.95479	859.98103	22
17	1416.74009	708.87368	1444.73500	722.87114	G	1661.93333	831.47030	21
18	1515.80850	758.40789	1543.80342	772.40535	V	1604.91187	802.95957	20
19	1572.82997	786.91862	1600.82488	800.91608	G	1505.84345	753.42536	19
20	1685.91403	843.46065	1713.90895	857.45811	I	1448.82199	724.91463	18
21	1782.96679	891.98704	1810.96171	905.98449	P	1335.73792	668.37260	17
22	1839.98826	920.49777	1867.98317	934.49522	G	1238.68516	619.84622	16
23	1897.00972	949.00850	1925.00464	963.00596	G	1181.66370	591.33549	15
24	1996.07814	998.54271	2024.07305	1012.54016	V	1124.64223	562.82475	14
25	2095.14655	1048.07691	2123.14146	1062.07437	V	1025.57382	513.29055	13
26	2152.16801	1076.58764	2180.16293	1090.58510	G	926.50541	463.75634	12
27	2223.20513	1112.10620	2251.20004	1126.10366	A	869.48394	435.24561	11
28	2280.22659	1140.61693	2308.22151	1154.61439	G	798.44683	399.72705	10
29	2377.27935	1189.14332	2405.27427	1203.14077	P	741.42536	371.21632	9
30	2448.31647	1224.66187	2476.31138	1238.65933	A	644.37260	322.68994	8
31	2519.35358	1260.18043	2547.34850	1274.17789	A	573.33549	287.17138	7
32	2590.39070	1295.69899	2618.38561	1309.69644	A	502.29837	251.65282	6
33	2661.42781	1331.21754	2689.42272	1345.21500	A	431.26126	216.13427	5
34	2732.46492	1366.73610	2760.45984	1380.73356	A	360.22415	180.61571	4
35	2803.50204	1402.25466	2831.49695	1416.25211	A	289.18703	145.09715	3
36	2874.53915	1437.77321	2902.53407	1451.77067	A	218.14992	109.57860	2
37					K	147.11280	74.06004	1

YGAAVPGVLGGLGALGGVGIPGGVVGAGPAAAAAAK, Y1-dichlorination (67.92206 Da)

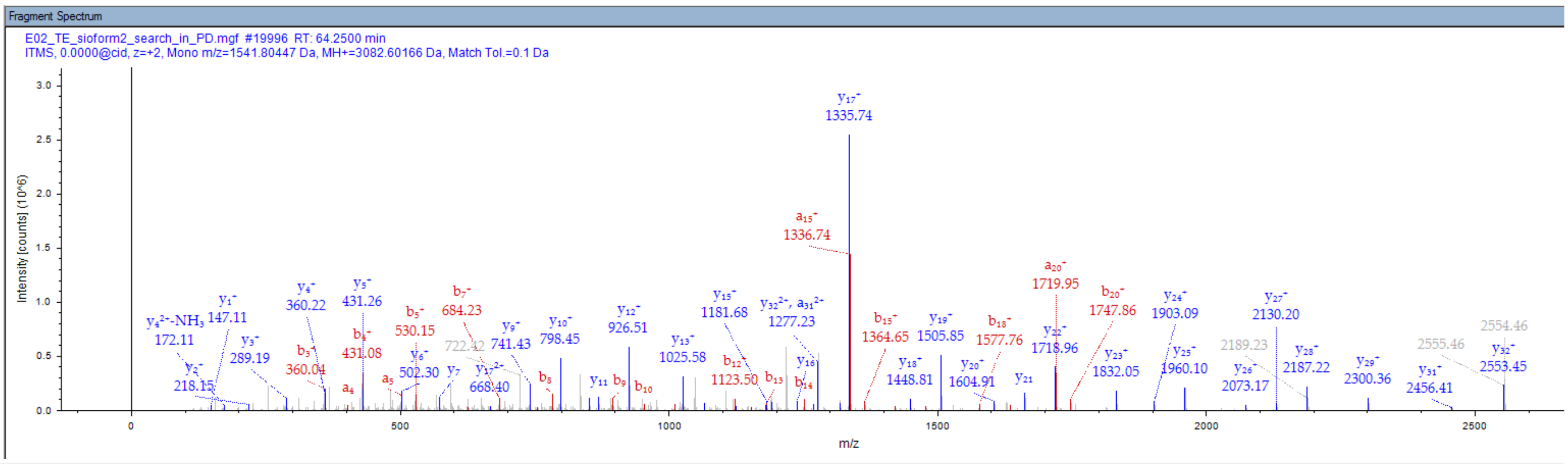

Ion Series | Neutral Losses | Precursor Ions | Internal Fragments

#1	a⁺	a²⁺	b⁺	b²⁺	Seq.	y⁺	y²⁺	#2
1	203.99775	102.50251	231.99266	116.49997	Y-dichlorin...			37
2	261.01921	131.01324	289.01412	145.01070	G	2851.61524	1426.31126	36
3	332.05632	166.53180	360.05124	180.52926	A	2794.59377	1397.80052	35
4	403.09344	202.05036	431.08835	216.04781	A	2723.55666	1362.28197	34
5	502.16185	251.58456	530.15677	265.58202	V	2652.51955	1326.76341	33
6	599.21461	300.11095	627.20953	314.10840	P	2553.45113	1277.22920	32
7	656.23608	328.62168	684.23099	342.61913	G	2456.39837	1228.70282	31
8	755.30449	378.15588	783.29941	392.15334	V	2399.37690	1200.19209	30
9	868.38856	434.69792	896.38347	448.69537	L	2300.30849	1150.65788	29
10	925.41002	463.20865	953.40493	477.20611	G	2187.22443	1094.11585	28
11	982.43148	491.71938	1010.42640	505.71684	G	2130.20296	1065.60512	27
12	1095.51555	548.26141	1123.51046	562.25887	L	2073.18150	1037.09439	26
13	1152.53701	576.77214	1180.53193	590.76960	G	1960.09743	980.55236	25
14	1223.57412	612.29070	1251.56904	626.28816	A	1903.07597	952.04162	24
15	1336.65819	668.83273	1364.65310	682.83019	L	1832.03886	916.52307	23
16	1393.67965	697.34346	1421.67457	711.34092	G	1718.95479	859.98103	22
17	1450.70112	725.85420	1478.69603	739.85165	G	1661.93333	831.47030	21
18	1549.76953	775.38840	1577.76444	789.38586	V	1604.91187	802.95957	20
19	1606.79099	803.89914	1634.78591	817.89659	G	1505.84345	753.42536	19
20	1719.87506	860.44117	1747.86997	874.43862	I	1448.82199	724.91463	18
21	1816.92782	908.96755	1844.92274	922.96501	P	1335.73792	668.37260	17
22	1873.94928	937.47828	1901.94420	951.47574	G	1238.68516	619.84622	16
23	1930.97075	965.98901	1958.96566	979.98647	G	1181.66370	591.33549	15
24	2030.03916	1015.52322	2058.03408	1029.52068	V	1124.64223	562.82475	14
25	2129.10758	1065.05743	2157.10249	1079.05488	V	1025.57382	513.29055	13
26	2186.12904	1093.56816	2214.12395	1107.56562	G	926.50541	463.75634	12
27	2257.16615	1129.08672	2285.16107	1143.08417	A	869.48394	435.24561	11
28	2314.18762	1157.59745	2342.18253	1171.59490	G	798.44683	399.72705	10
29	2411.24038	1206.12383	2439.23530	1220.12129	P	741.42536	371.21632	9
30	2482.27750	1241.64239	2510.27241	1255.63984	A	644.37260	322.68994	8
31	2553.31461	1277.16094	2581.30952	1291.15840	A	573.33549	287.17138	7
32	2624.35172	1312.67950	2652.34664	1326.67696	A	502.29837	251.65282	6
33	2695.38884	1348.19806	2723.38375	1362.19551	A	431.26126	216.13427	5
34	2766.42595	1383.71661	2794.42086	1397.71407	A	360.22415	180.61571	4
35	2837.46306	1419.23517	2865.45798	1433.23263	A	289.18703	145.09715	3
36	2908.50018	1454.75373	2936.49509	1468.75118	A	218.14992	109.57860	2
37					K	147.11280	74.06004	1

YGVGTPAAAAK

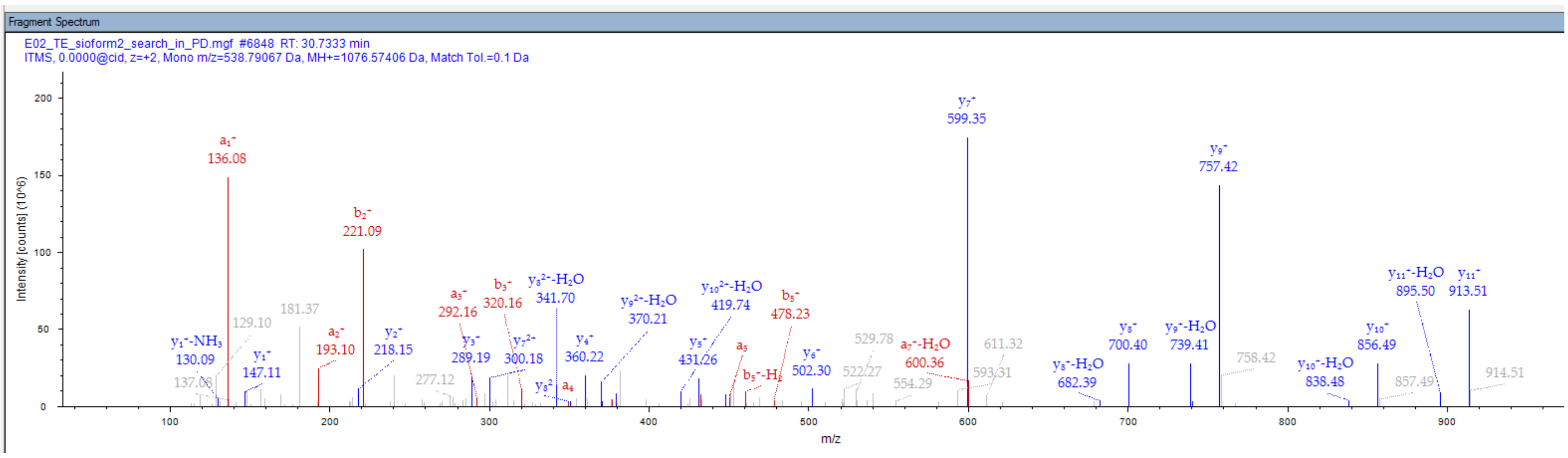

Ion Series | Neutral Losses | Precursor Ions | Internal Fragments

#1	a⁺	a²⁺	b⁺	b²⁺	Seq.	y⁺	y²⁺	#2
1	136.07569	68.54148	164.07061	82.53894	Y			12
2	193.09715	97.05222	221.09207	111.04967	G	913.51016	457.25872	11
3	292.16557	146.58642	320.16048	160.58388	V	856.48869	428.74798	10
4	349.18703	175.09715	377.18195	189.09461	G	757.42028	379.21378	9
5	450.23471	225.62099	478.22962	239.61845	T	700.39882	350.70305	8
6	547.28747	274.14738	575.28239	288.14483	P	599.35114	300.17921	7
7	618.32459	309.66593	646.31950	323.66339	A	502.29837	251.65282	6
8	689.36170	345.18449	717.35662	359.18195	A	431.26126	216.13427	5
9	760.39882	380.70305	788.39373	394.70050	A	360.22415	180.61571	4
10	831.43593	416.22160	859.43084	430.21906	A	289.18703	145.09715	3
11	902.47304	451.74016	930.46796	465.73762	A	218.14992	109.57860	2
12					K	147.11280	74.06004	1

YGVGTPAAAAK, Y1-Chlorination (33.96103 Da)

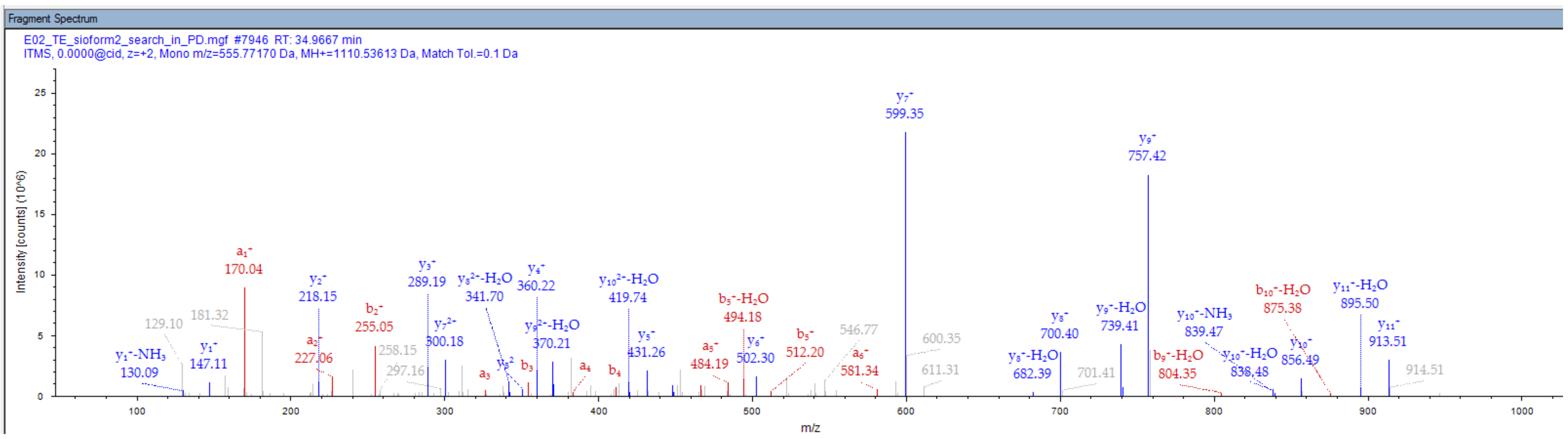

Ion Series	Neutral Losses	Precursor Ions	Internal Fragments

#1	a⁺	a²⁺	b⁺	b²⁺	Seq.	y⁺	y²⁺	#2
1	170.03672	85.52200	198.03163	99.51945	Y-Chlorinat...			12
2	227.05818	114.03273	255.05310	128.03019	G	913.51016	457.25872	11
3	326.12660	163.56694	354.12151	177.56439	V	856.48869	428.74798	10
4	383.14806	192.07767	411.14297	206.07513	G	757.42028	379.21378	9
5	484.19574	242.60151	512.19065	256.59896	T	700.39882	350.70305	8
6	581.24850	291.12789	609.24342	305.12535	P	599.35114	300.17921	7
7	652.28562	326.64645	680.28053	340.64390	A	502.29837	251.65282	6
8	723.32273	362.16500	751.31764	376.16246	A	431.26126	216.13427	5
9	794.35984	397.68356	822.35476	411.68102	A	360.22415	180.61571	4
10	865.39696	433.20212	893.39187	447.19957	A	289.18703	145.09715	3
11	936.43407	468.72067	964.42899	482.71813	A	218.14992	109.57860	2
12					K	147.11280	74.06004	1

YGVGTPAAAAK, Y1-dichlorination (67.92206 Da)

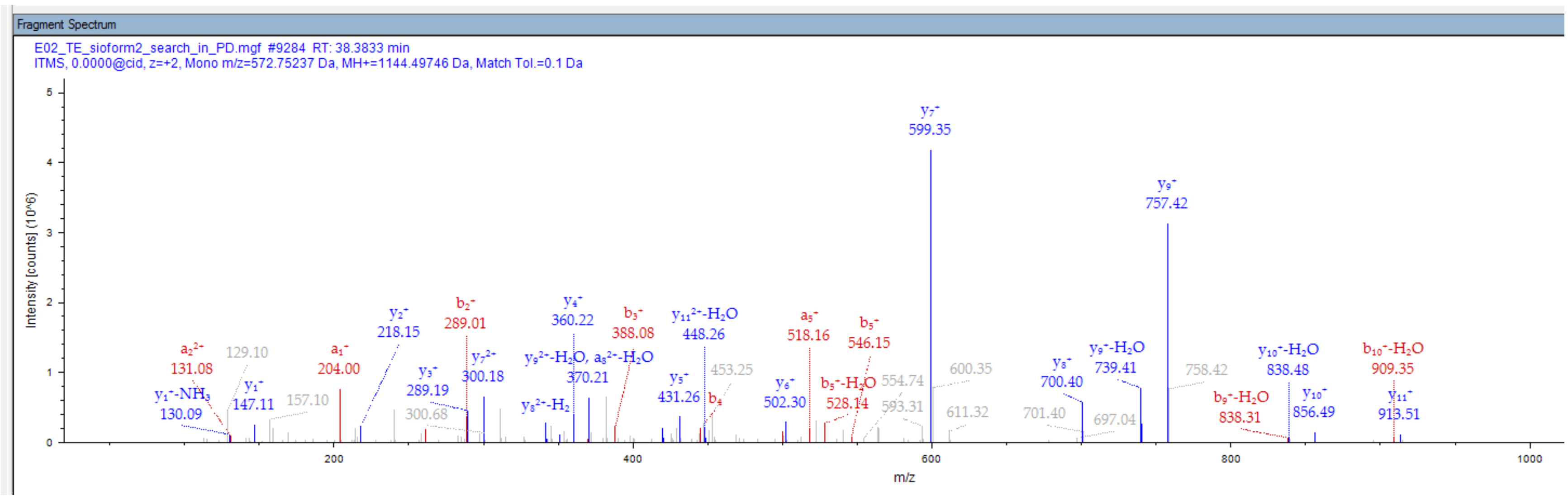

Ion Series | Neutral Losses | Precursor Ions | Internal Fragments

#1	a⁺	a²⁺	b⁺	b²⁺	Seq.	y⁺	y²⁺	#2
1	203.99775	102.50251	231.99266	116.49997	Y-dichlorin...			12
2	261.01921	131.01324	289.01412	145.01070	G	913.51016	457.25872	11
3	360.08762	180.54745	388.08254	194.54491	V	856.48869	428.74798	10
4	417.10909	209.05818	445.10400	223.05564	G	757.42028	379.21378	9
5	518.15677	259.58202	546.15168	273.57948	T	700.39882	350.70305	8
6	615.20953	308.10840	643.20444	322.10586	P	599.35114	300.17921	7
7	686.24664	343.62696	714.24156	357.62442	A	502.29837	251.65282	6
8	757.28376	379.14552	785.27867	393.14297	A	431.26126	216.13427	5
9	828.32087	414.66407	856.31578	428.66153	A	360.22415	180.61571	4
10	899.35798	450.18263	927.35290	464.18009	A	289.18703	145.09715	3
11	970.39510	485.70119	998.39001	499.69864	A	218.14992	109.57860	2
12					K	147.11280	74.06004	1

Tropoelastin Isoform 6

Treated with HOCl

VPGVGLPGVYPGGVLPGAR

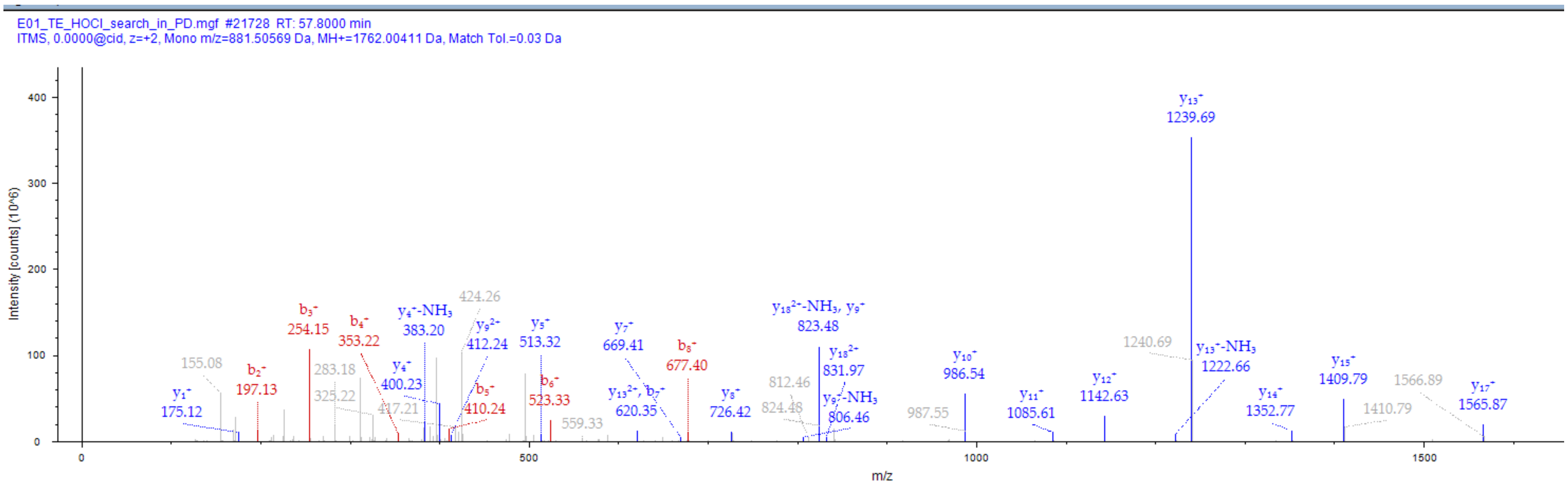

#1	Immonium	b^+	b^{2+}	Seq.	y^+	y^{2+}	#2
1	72.08078	100.07569	50.54148	V			19
2	70.06513	197.12845	99.06787	P	1662.93260	831.96994	18
3	30.03383	254.14992	127.57860	G	1565.87984	783.44356	17
4	72.08078	353.21833	177.11280	V	1508.85837	754.93283	16
5	30.03383	410.23980	205.62354	G	1409.78996	705.39862	15
6	86.09643	523.32386	262.16557	L	1352.76850	676.88789	14
7	70.06513	620.37662	310.69195	P	1239.68443	620.34585	13
8	30.03383	677.39809	339.20268	G	1142.63167	571.81947	12
9	72.08078	776.46650	388.73689	V	1085.61020	543.30874	11
10	136.07569	939.52983	470.26855	Y	986.54179	493.77453	10
11	70.06513	1036.58259	518.79493	P	823.47846	412.24287	9
12	30.03383	1093.60406	547.30567	G	726.42570	363.71649	8
13	30.03383	1150.62552	575.81640	G	669.40423	335.20576	7
14	72.08078	1249.69393	625.35061	V	612.38277	306.69502	6
15	86.09643	1362.77800	681.89264	L	513.31436	257.16082	5
16	70.06513	1459.83076	730.41902	P	400.23029	200.61879	4
17	30.03383	1516.85223	758.92975	G	303.17753	152.09240	3
18	44.04948	1587.88934	794.44831	A	246.15607	123.58167	2
19	129.11347			R	175.11895	88.06311	1

VPGVGLPGVYPGGVLPGAR, Y10-Chlorination (33.96103 Da)

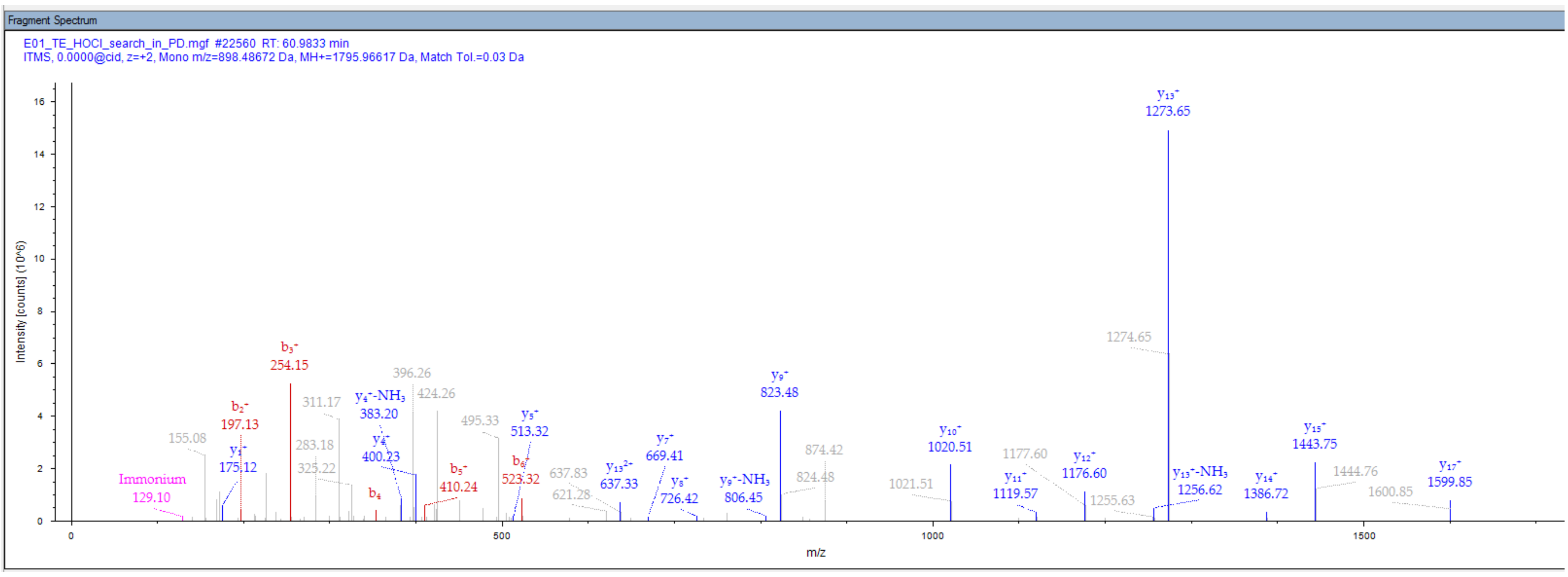

#1	Immonium	b+	b2+	Seq.	y+	y2+	#2
1	72.08078	100.07569	50.54148	V			19
2	70.06513	197.12845	99.06787	P	1696.89363	848.95045	18
3	30.03383	254.14992	127.57860	G	1599.84087	800.42407	17
4	72.08078	353.21833	177.11280	V	1542.81940	771.91334	16
5	30.03383	410.23980	205.62354	G	1443.75099	722.37913	15
6	86.09643	523.32386	262.16557	L	1386.72952	693.86840	14
7	70.06513	620.37662	310.69195	P	1273.64546	637.32637	13
8	30.03383	677.39809	339.20268	G	1176.59270	588.79999	12
9	72.08078	776.46650	388.73689	V	1119.57123	560.28925	11
10	170.03672	973.49086	487.24907	Y-Chlorinat...	1020.50282	510.75505	10
11	70.06513	1070.54362	535.77545	P	823.47846	412.24287	9
12	30.03383	1127.56508	564.28618	G	726.42570	363.71649	8
13	30.03383	1184.58655	592.79691	G	669.40423	335.20576	7
14	72.08078	1283.65496	642.33112	V	612.38277	306.69502	6
15	86.09643	1396.73903	698.87315	L	513.31436	257.16082	5
16	70.06513	1493.79179	747.39953	P	400.23029	200.61879	4
17	30.03383	1550.81325	775.91027	G	303.17753	152.09240	3
18	44.04948	1621.85037	811.42882	A	246.15607	123.58167	2
19	129.11347			R	175.11895	88.06311	1

VPGVGLPGVYPGGVLPGAR, Y10-dichlorination (67.92206 Da)

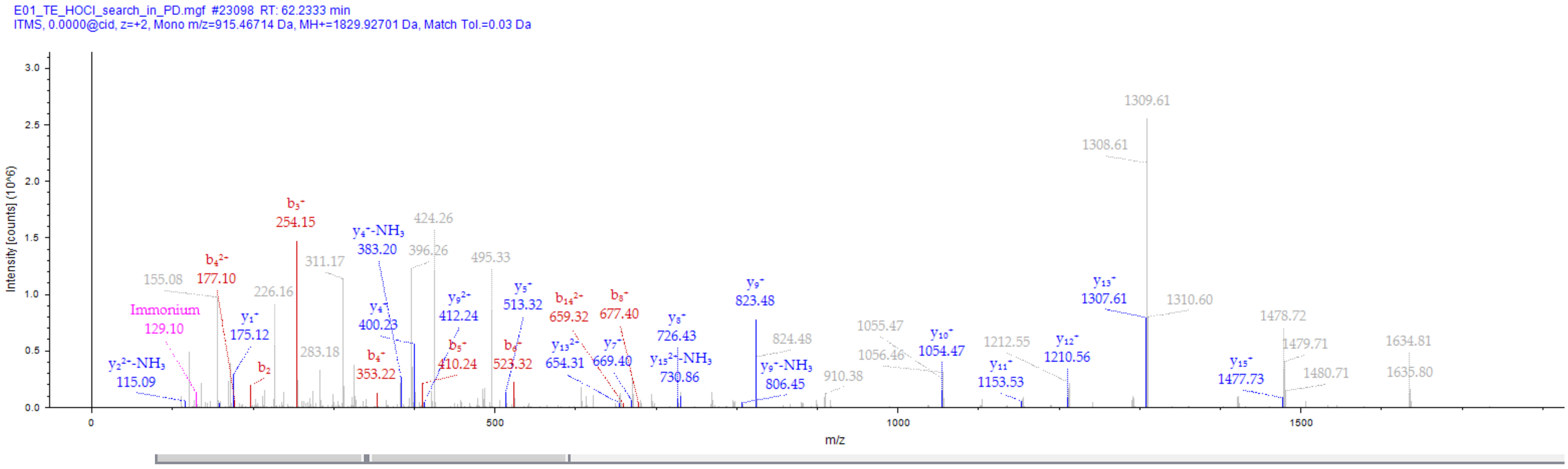

#1	Immonium	b⁺	b²⁺	Seq.	y⁺	y²⁺	#2
1	72.08078	100.07569	50.54148	V			19
2	70.06513	197.12845	99.06787	P	1730.85466	865.93097	18
3	30.03383	254.14992	127.57860	G	1633.80189	817.40458	17
4	72.08078	353.21833	177.11280	V	1576.78043	788.89385	16
5	30.03383	410.23980	205.62354	G	1477.71201	739.35965	15
6	86.09643	523.32386	262.16557	L	1420.69055	710.84891	14
7	70.06513	620.37662	310.69195	P	1307.60649	654.30688	13
8	30.03383	677.39809	339.20268	G	1210.55372	605.78050	12
9	72.08078	776.46650	388.73689	V	1153.53226	577.26977	11
10	203.99775	1007.45188	504.22958	Y-dichlorin...	1054.46385	527.73556	10
11	70.06513	1104.50465	552.75596	P	823.47846	412.24287	9
12	30.03383	1161.52611	581.26669	G	726.42570	363.71649	8
13	30.03383	1218.54758	609.77743	G	669.40423	335.20576	7
14	72.08078	1317.61599	659.31163	V	612.38277	306.69502	6
15	86.09643	1430.70005	715.85366	L	513.31436	257.16082	5
16	70.06513	1527.75282	764.38005	P	400.23029	200.61879	4
17	30.03383	1584.77428	792.89078	G	303.17753	152.09240	3
18	44.04948	1655.81139	828.40934	A	246.15607	123.58167	2
19	129.11347			R	175.11895	88.06311	1

APKLPGGYGLPYTTGKLPYGYGPGGVAG AAGK

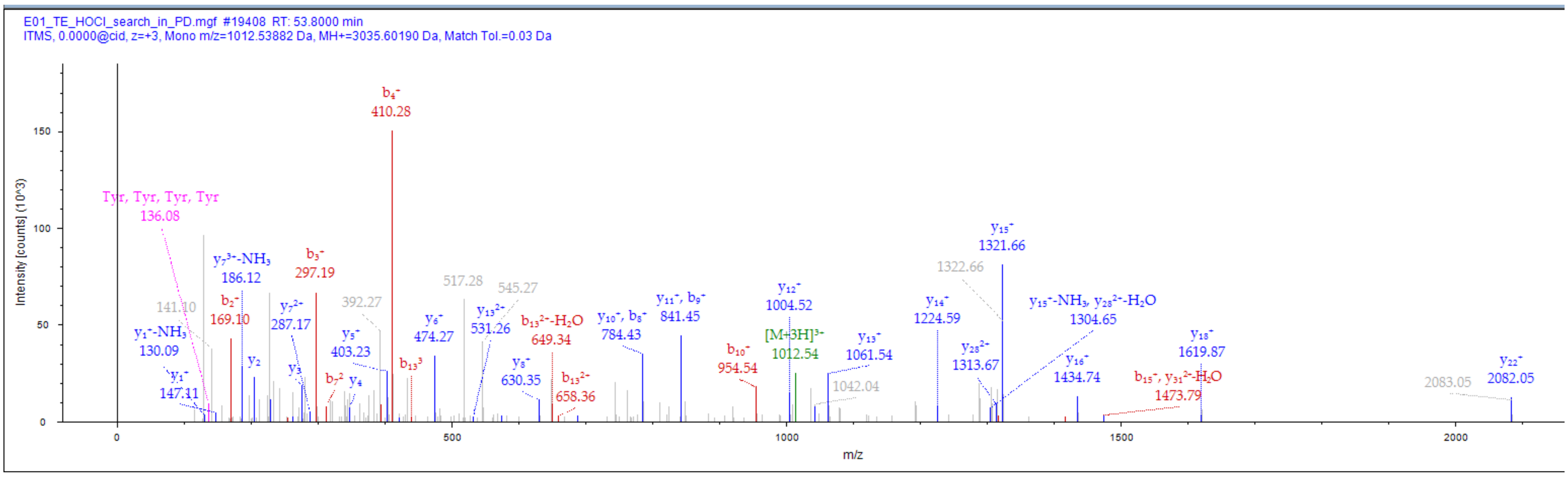

#1	Immonium	b⁺	b²⁺	b³⁺	Seq.	y⁺	y²⁺	y³⁺	#2
1	44.04948	72.04439	36.52583	24.68631	A				32
2	70.06513	169.09715	85.05222	57.03724	P	2964.56180	1482.78454	988.85879	31
3	101.10732	297.19212	149.09970	99.73556	K	2867.50904	1434.25816	956.50786	30
4	86.09643	410.27618	205.64173	137.43024	L	2739.41408	1370.21068	913.80954	29
5	70.06513	507.32894	254.16811	169.78117	P	2626.33001	1313.66864	876.11486	28
6	30.03383	564.35041	282.67884	188.78832	G	2529.27725	1265.14226	843.76393	27
7	30.03383	621.37187	311.18957	207.79548	G	2472.25579	1236.63153	824.75678	26
8	136.07569	784.43520	392.72124	262.14992	Y	2415.23432	1208.12080	805.74963	25
9	30.03383	841.45666	421.23197	281.15707	G	2252.17099	1126.58914	751.39518	24
10	86.09643	954.54073	477.77400	318.85176	L	2195.14953	1098.07840	732.38803	23
11	70.06513	1051.59349	526.30038	351.20268	P	2082.06547	1041.53637	694.69334	22
12	136.07569	1214.65682	607.83205	405.55712	Y	1985.01270	993.00999	662.34242	21
13	74.06004	1315.70450	658.35589	439.23968	T	1821.94937	911.47833	607.98798	20
14	74.06004	1416.75218	708.87973	472.92224	T	1720.90170	860.95449	574.30542	19
15	30.03383	1473.77364	737.39046	491.92940	G	1619.85402	810.43065	540.62286	18
16	101.10732	1601.86860	801.43794	534.62772	K	1562.83255	781.91991	521.61570	17
17	86.09643	1714.95267	857.97997	572.32241	L	1434.73759	717.87243	478.91738	16
18	70.06513	1812.00543	906.50635	604.67333	P	1321.65353	661.33040	441.22269	15
19	136.07569	1975.06876	988.03802	659.02777	Y	1224.60076	612.80402	408.87177	14
20	30.03383	2032.09022	1016.54875	678.03493	G	1061.53743	531.27236	354.51733	13
21	136.07569	2195.15355	1098.08041	732.38937	Y	1004.51597	502.76162	335.51017	12
22	30.03383	2252.17502	1126.59115	751.39652	G	841.45264	421.22996	281.15573	11
23	70.06513	2349.22778	1175.11753	783.74744	P	784.43118	392.71923	262.14858	10
24	30.03383	2406.24924	1203.62826	802.75460	G	687.37841	344.19285	229.79766	9
25	30.03383	2463.27071	1232.13899	821.76175	G	630.35695	315.68211	210.79050	8
26	72.08078	2562.33912	1281.67320	854.78456	V	573.33549	287.17138	191.78335	7
27	44.04948	2633.37623	1317.19176	878.46360	A	474.26707	237.63717	158.76054	6
28	30.03383	2690.39770	1345.70249	897.47075	G	403.22996	202.11862	135.08150	5
29	44.04948	2761.43481	1381.22104	921.14979	A	346.20850	173.60789	116.07435	4
30	44.04948	2832.47193	1416.73960	944.82883	A	275.17138	138.08933	92.39531	3
31	30.03383	2889.49339	1445.25033	963.83598	G	204.13427	102.57077	68.71627	2
32	101.10732				K	147.11280	74.06004	49.70912	1

APKLPGGYGLPYTTGKLPYGYGPGGVAGAAGK, Y8-Chlorination (33.96103 Da)

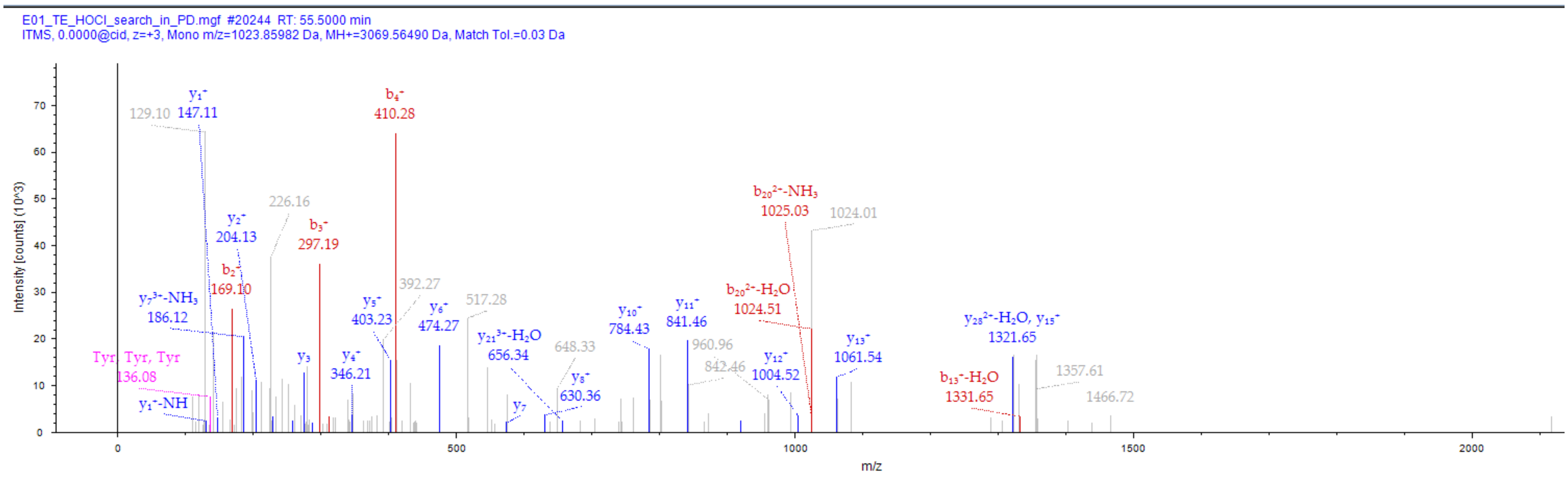

#1	Immonium	b⁺	b²⁺	b³⁺	Seq.	y⁺	y²⁺	y³⁺	#2
1	44.04948	72.04439	36.52583	24.68631	A				32
2	70.06513	169.09715	85.05222	57.03724	P	2998.52283	1499.76505	1000.17913	31
3	101.10732	297.19212	149.09970	99.73556	K	2901.47007	1451.23867	967.82821	30
4	86.09643	410.27618	205.64173	137.43024	L	2773.37510	1387.19119	925.12989	29
5	70.06513	507.32894	254.16811	169.78117	P	2660.29104	1330.64916	887.43520	28
6	30.03383	564.35041	282.67884	188.78832	G	2563.23828	1282.12278	855.08428	27
7	30.03383	621.37187	311.18957	207.79548	G	2506.21681	1253.61205	836.07712	26
8	170.03672	818.39623	409.70175	273.47026	Y-Chlorinat...	2449.19535	1225.10131	817.06997	25
9	30.03383	875.41769	438.21248	292.47742	G	2252.17099	1126.58914	751.39518	24
10	86.09643	988.50176	494.75452	330.17210	L	2195.14953	1098.07840	732.38803	23
11	70.06513	1085.55452	543.28090	362.52302	P	2082.06547	1041.53637	694.69334	22
12	136.07569	1248.61785	624.81256	416.87747	Y	1985.01270	993.00999	662.34242	21
13	74.06004	1349.66553	675.33640	450.56003	T	1821.94937	911.47833	607.98798	20
14	74.06004	1450.71321	725.86024	484.24259	T	1720.90170	860.95449	574.30542	19
15	30.03383	1507.73467	754.37097	503.24974	G	1619.85402	810.43065	540.62286	18
16	101.10732	1635.82963	818.41845	545.94806	K	1562.83255	781.91991	521.61570	17
17	86.09643	1748.91370	874.96049	583.64275	L	1434.73759	717.87243	478.91738	16
18	70.06513	1845.96646	923.48687	615.99367	P	1321.65353	661.33040	441.22269	15
19	136.07569	2009.02979	1005.01853	670.34811	Y	1224.60076	612.80402	408.87177	14
20	30.03383	2066.05125	1033.52926	689.35527	G	1061.53743	531.27236	354.51733	13
21	136.07569	2229.11458	1115.06093	743.70971	Y	1004.51597	502.76162	335.51017	12
22	30.03383	2286.13604	1143.57166	762.71687	G	841.45264	421.22996	281.15573	11
23	70.06513	2383.18881	1192.09804	795.06779	P	784.43118	392.71923	262.14858	10
24	30.03383	2440.21027	1220.60877	814.07494	G	687.37841	344.19285	229.79766	9
25	30.03383	2497.23174	1249.11951	833.08210	G	630.35695	315.68211	210.79050	8
26	72.08078	2596.30015	1298.65371	866.10490	V	573.33549	287.17138	191.78335	7
27	44.04948	2667.33726	1334.17227	889.78394	A	474.26707	237.63717	158.76054	6
28	30.03383	2724.35873	1362.68300	908.79109	G	403.22996	202.11862	135.08150	5
29	44.04948	2795.39584	1398.20156	932.47013	A	346.20850	173.60789	116.07435	4
30	44.04948	2866.43295	1433.72012	956.14917	A	275.17138	138.08933	92.39531	3
31	30.03383	2923.45442	1462.23085	975.15632	G	204.13427	102.57077	68.71627	2
32	101.10732				K	147.11280	74.06004	49.70912	1

APKLPGGYGLPYTTGKLPYGYGPGGVAGAAGK, Y12-Chlorination (33.96103 Da)

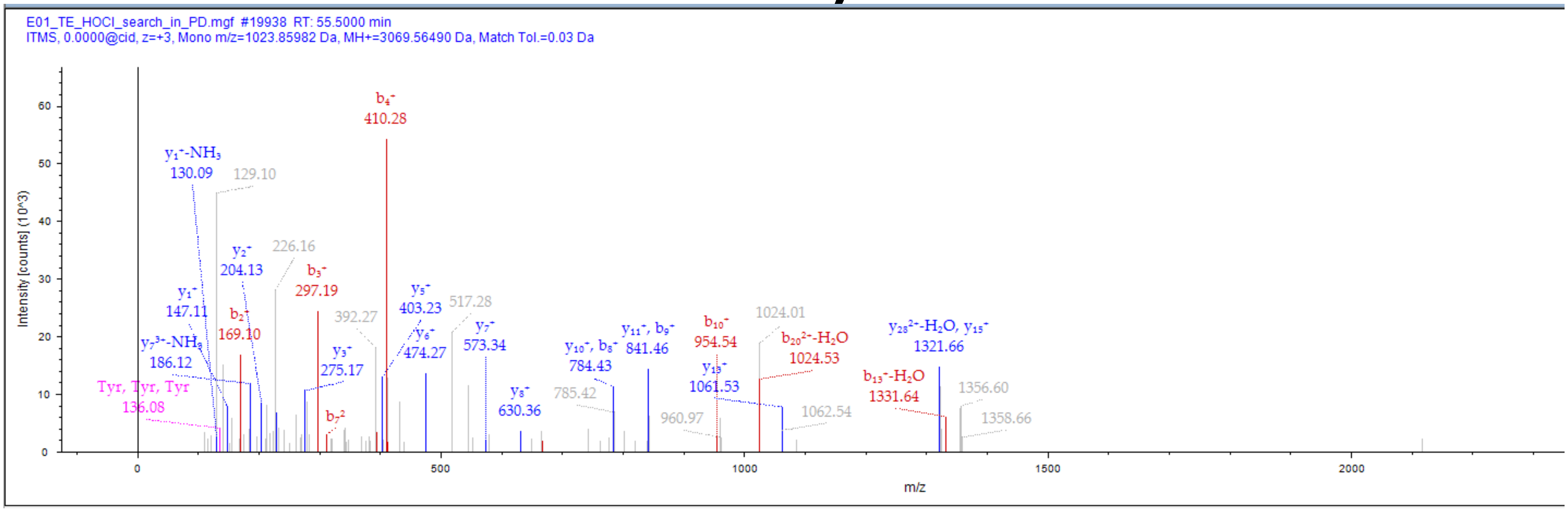

#1	Immonium	b⁺	b²⁺	b³⁺	Seq.	y⁺	y²⁺	y³⁺	#2
1	44.04948	72.04439	36.52583	24.68631	A				32
2	70.06513	169.09715	85.05222	57.03724	P	2998.52283	1499.76505	1000.17913	31
3	101.10732	297.19212	149.09970	99.73556	K	2901.47007	1451.23867	967.82821	30
4	86.09643	410.27618	205.64173	137.43024	L	2773.37510	1387.19119	925.12989	29
5	70.06513	507.32894	254.16811	169.78117	P	2660.29104	1330.64916	887.43520	28
6	30.03383	564.35041	282.67884	188.78832	G	2563.23828	1282.12278	855.08428	27
7	30.03383	621.37187	311.18957	207.79548	G	2506.21681	1253.61205	836.07712	26
8	136.07569	784.43520	392.72124	262.14992	Y	2449.19535	1225.10131	817.06997	25
9	30.03383	841.45666	421.23197	281.15707	G	2286.13202	1143.56965	762.71552	24
10	86.09643	954.54073	477.77400	318.85176	L	2229.11056	1115.05892	743.70837	23
11	70.06513	1051.59349	526.30038	351.20268	P	2116.02649	1058.51689	706.01368	22
12	170.03672	1248.61785	624.81256	416.87747	Y-Chlorinat...	2018.97373	1009.99050	673.66276	21
13	74.06004	1349.66553	675.33640	450.56003	T	1821.94937	911.47833	607.98798	20
14	74.06004	1450.71321	725.86024	484.24259	T	1720.90170	860.95449	574.30542	19
15	30.03383	1507.73467	754.37097	503.24974	G	1619.85402	810.43065	540.62286	18
16	101.10732	1635.82963	818.41845	545.94806	K	1562.83255	781.91991	521.61570	17
17	86.09643	1748.91370	874.96049	583.64275	L	1434.73759	717.87243	478.91738	16
18	70.06513	1845.96646	923.48687	615.99367	P	1321.65353	661.33040	441.22269	15
19	136.07569	2009.02979	1005.01853	670.34811	Y	1224.60076	612.80402	408.87177	14
20	30.03383	2066.05125	1033.52926	689.35527	G	1061.53743	531.27236	354.51733	13
21	136.07569	2229.11458	1115.06093	743.70971	Y	1004.51597	502.76162	335.51017	12
22	30.03383	2286.13604	1143.57166	762.71687	G	841.45264	421.22996	281.15573	11
23	70.06513	2383.18881	1192.09804	795.06779	P	784.43118	392.71923	262.14858	10
24	30.03383	2440.21027	1220.60877	814.07494	G	687.37841	344.19285	229.79766	9
25	30.03383	2497.23174	1249.11951	833.08210	G	630.35695	315.68211	210.79050	8
26	72.08078	2596.30015	1298.65371	866.10490	V	573.33549	287.17138	191.78335	7
27	44.04948	2667.33726	1334.17227	889.78394	A	474.26707	237.63717	158.76054	6
28	30.03383	2724.35873	1362.68300	908.79109	G	403.22996	202.11862	135.08150	5
29	44.04948	2795.39584	1398.20156	932.47013	A	346.20850	173.60789	116.07435	4
30	44.04948	2866.43295	1433.72012	956.14917	A	275.17138	138.08933	92.39531	3
31	30.03383	2923.45442	1462.23085	975.15632	G	204.13427	102.57077	68.71627	2
32	101.10732				K	147.11280	74.06004	49.70912	1

APKLPGGYGLPYTTGKLPYGYGPGGVAGAAGK, Y19-Chlorination (33.96103 Da)

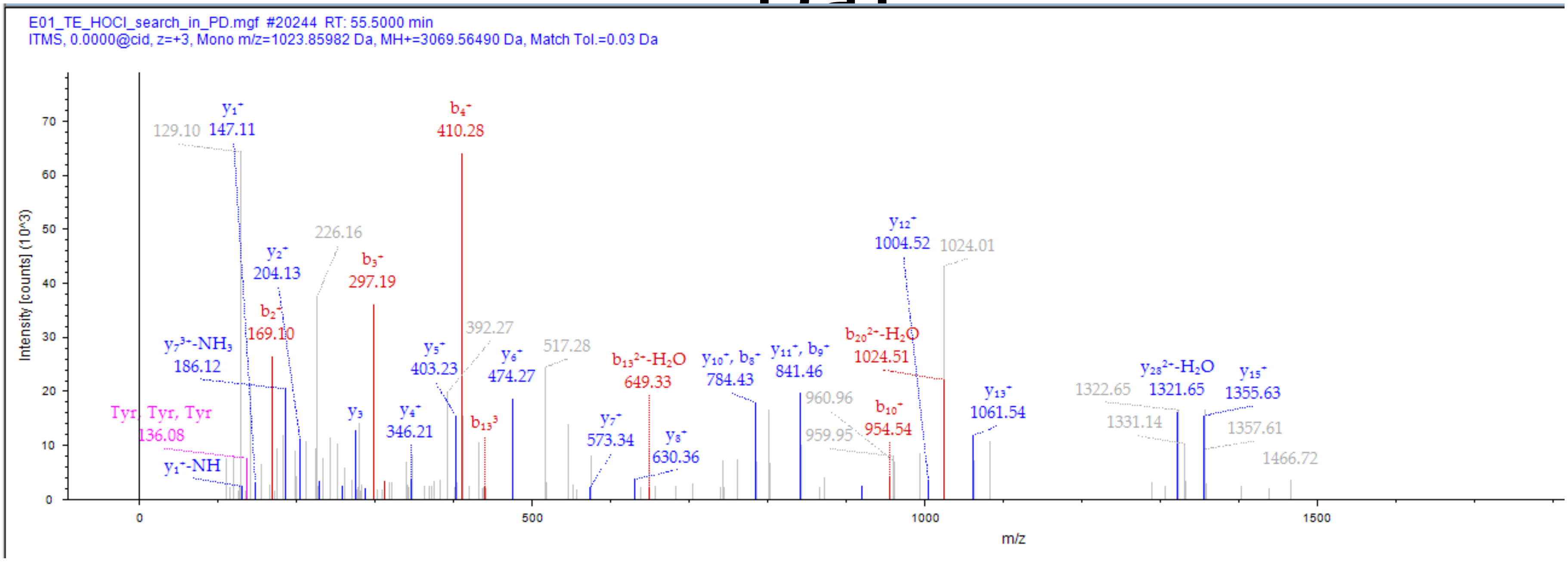

#1	Immonium	b⁺	b²⁺	b³⁺	Seq.	y⁺	y²⁺	y³⁺	#2
1	44.04948	72.04439	36.52583	24.68631	A				32
2	70.06513	169.09715	85.05222	57.03724	P	2998.52283	1499.76505	1000.17913	31
3	101.10732	297.19212	149.09970	99.73556	K	2901.47007	1451.23867	967.82821	30
4	86.09643	410.27618	205.64173	137.43024	L	2773.37510	1387.19119	925.12989	29
5	70.06513	507.32894	254.16811	169.78117	P	2660.29104	1330.64916	887.43520	28
6	30.03383	564.35041	282.67884	188.78832	G	2563.23828	1282.12278	855.08428	27
7	30.03383	621.37187	311.18957	207.79548	G	2506.21681	1253.61205	836.07712	26
8	136.07569	784.43520	392.72124	262.14992	Y	2449.19535	1225.10131	817.06997	25
9	30.03383	841.45666	421.23197	281.15707	G	2286.13202	1143.56965	762.71552	24
10	86.09643	954.54073	477.77400	318.85176	L	2229.11056	1115.05892	743.70837	23
11	70.06513	1051.59349	526.30038	351.20268	P	2116.02649	1058.51689	706.01368	22
12	136.07569	1214.65682	607.83205	405.55712	Y	2018.97373	1009.99050	673.66276	21
13	74.06004	1315.70450	658.35589	439.23968	T	1855.91040	928.45884	619.30832	20
14	74.06004	1416.75218	708.87973	472.92224	T	1754.86272	877.93500	585.62576	19
15	30.03383	1473.77364	737.39046	491.92940	G	1653.81504	827.41116	551.94320	18
16	101.10732	1601.86860	801.43794	534.62772	K	1596.79358	798.90043	532.93604	17
17	86.09643	1714.95267	857.97997	572.32241	L	1468.69862	734.85295	490.23772	16
18	70.06513	1812.00543	906.50635	604.67333	P	1355.61455	678.31092	452.54304	15
19	170.03672	2009.02979	1005.01853	670.34811	Y-Chlorinat...	1258.56179	629.78453	420.19211	14
20	30.03383	2066.05125	1033.52926	689.35527	G	1061.53743	531.27236	354.51733	13
21	136.07569	2229.11458	1115.06093	743.70971	Y	1004.51597	502.76162	335.51017	12
22	30.03383	2286.13604	1143.57166	762.71687	G	841.45264	421.22996	281.15573	11
23	70.06513	2383.18881	1192.09804	795.06779	P	784.43118	392.71923	262.14858	10
24	30.03383	2440.21027	1220.60877	814.07494	G	687.37841	344.19285	229.79766	9
25	30.03383	2497.23174	1249.11951	833.08210	G	630.35695	315.68211	210.79050	8
26	72.08078	2596.30015	1298.65371	866.10490	V	573.33549	287.17138	191.78335	7
27	44.04948	2667.33726	1334.17227	889.78394	A	474.26707	237.63717	158.76054	6
28	30.03383	2724.35873	1362.68300	908.79109	G	403.22996	202.11862	135.08150	5
29	44.04948	2795.39584	1398.20156	932.47013	A	346.20850	173.60789	116.07435	4
30	44.04948	2866.43295	1433.72012	956.14917	A	275.17138	138.08933	92.39531	3
31	30.03383	2923.45442	1462.23085	975.15632	G	204.13427	102.57077	68.71627	2
32	101.10732				K	147.11280	74.06004	49.70912	1

APKLPGGYGLPYTTGKLPYGYGPGGVAGAAGK, Y8-Chlorination (33.96103 Da)

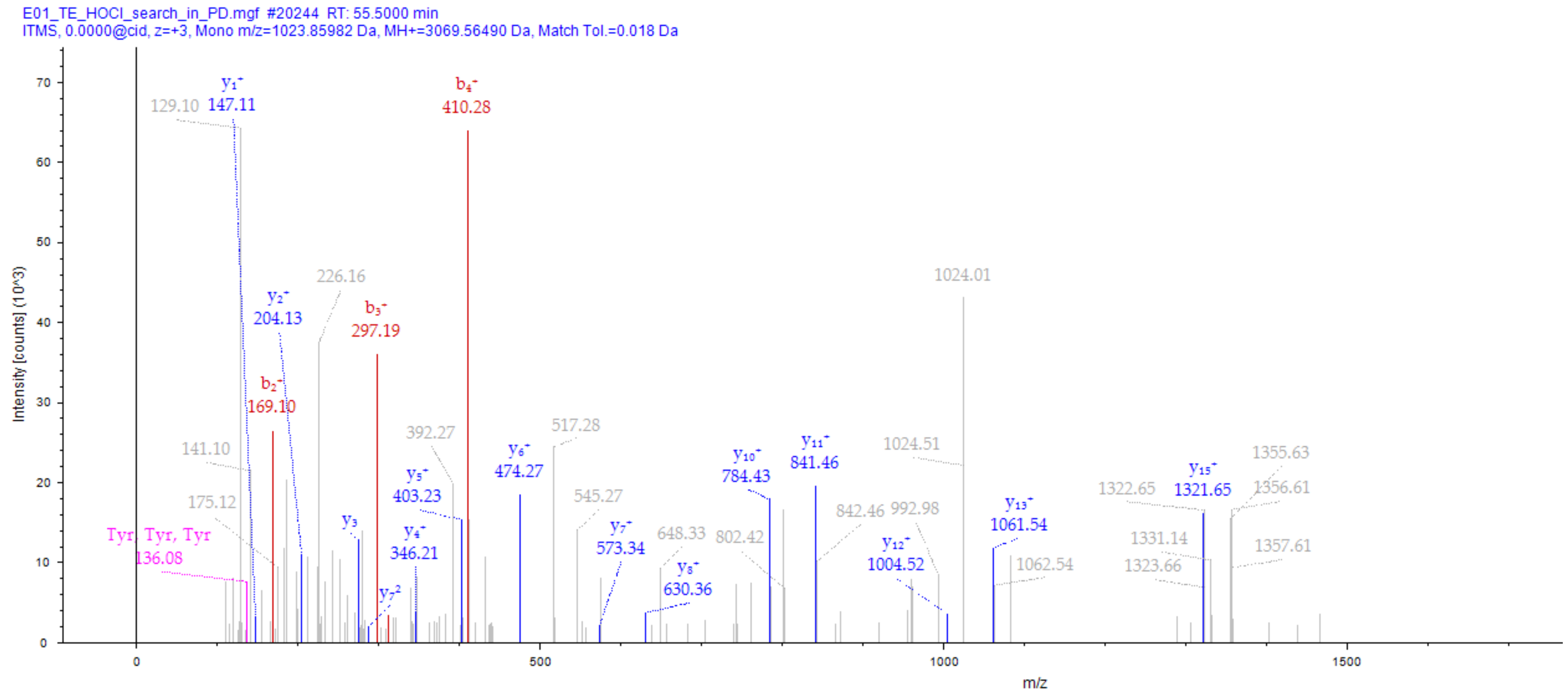

#1	Immonium	b⁺	b²⁺	b³⁺	Seq.	y⁺	y²⁺	y³⁺	#2
1	44.04948	72.04439	36.52583	24.68631	A				32
2	70.06513	169.09715	85.05222	57.03724	P	2998.52283	1499.76505	1000.17913	31
3	101.10732	297.19212	149.09970	99.73556	K	2901.47007	1451.23867	967.82821	30
4	86.09643	410.27618	205.64173	137.43024	L	2773.37510	1387.19119	925.12989	29
5	70.06513	507.32894	254.16811	169.78117	P	2660.29104	1330.64916	887.43520	28
6	30.03383	564.35041	282.67884	188.78832	G	2563.23828	1282.12278	855.08428	27
7	30.03383	621.37187	311.18957	207.79548	G	2506.21681	1253.61205	836.07712	26
8	170.03672	818.39623	409.70175	273.47026	Y-Chlorinat...	2449.19535	1225.10131	817.06997	25
9	30.03383	875.41769	438.21248	292.47742	G	2252.17099	1126.58914	751.39518	24
10	86.09643	988.50176	494.75452	330.17210	L	2195.14953	1098.07840	732.38803	23
11	70.06513	1085.55452	543.28090	362.52302	P	2082.06547	1041.53637	694.69334	22
12	136.07569	1248.61785	624.81256	416.87747	Y	1985.01270	993.00999	662.34242	21
13	74.06004	1349.66553	675.33640	450.56003	T	1821.94937	911.47833	607.98798	20
14	74.06004	1450.71321	725.86024	484.24259	T	1720.90170	860.95449	574.30542	19
15	30.03383	1507.73467	754.37097	503.24974	G	1619.85402	810.43065	540.62286	18
16	101.10732	1635.82963	818.41845	545.94806	K	1562.83255	781.91991	521.61570	17
17	86.09643	1748.91370	874.96049	583.64275	L	1434.73759	717.87243	478.91738	16
18	70.06513	1845.96646	923.48687	615.99367	P	1321.65353	661.33040	441.22269	15
19	136.07569	2009.02979	1005.01853	670.34811	Y	1224.60076	612.80402	408.87177	14
20	30.03383	2066.05125	1033.52926	689.35527	G	1061.53743	531.27236	354.51733	13
21	136.07569	2229.11458	1115.06093	743.70971	Y	1004.51597	502.76162	335.51017	12
22	30.03383	2286.13604	1143.57166	762.71687	G	841.45264	421.22996	281.15573	11
23	70.06513	2383.18881	1192.09804	795.06779	P	784.43118	392.71923	262.14858	10
24	30.03383	2440.21027	1220.60877	814.07494	G	687.37841	344.19285	229.79766	9
25	30.03383	2497.23174	1249.11951	833.08210	G	630.35695	315.68211	210.79050	8
26	72.08078	2596.30015	1298.65371	866.10490	V	573.33549	287.17138	191.78335	7
27	44.04948	2667.33726	1334.17227	889.78394	A	474.26707	237.63717	158.76054	6
28	30.03383	2724.35873	1362.68300	908.79109	G	403.22996	202.11862	135.08150	5
29	44.04948	2795.39584	1398.20156	932.47013	A	346.20850	173.60789	116.07435	4
30	44.04948	2866.43295	1433.72012	956.14917	A	275.17138	138.08933	92.39531	3
31	30.03383	2923.45442	1462.23085	975.15632	G	204.13427	102.57077	68.71627	2
32	101.10732				K	147.11280	74.06004	49.70912	1

LPGGYGLPYTTGK

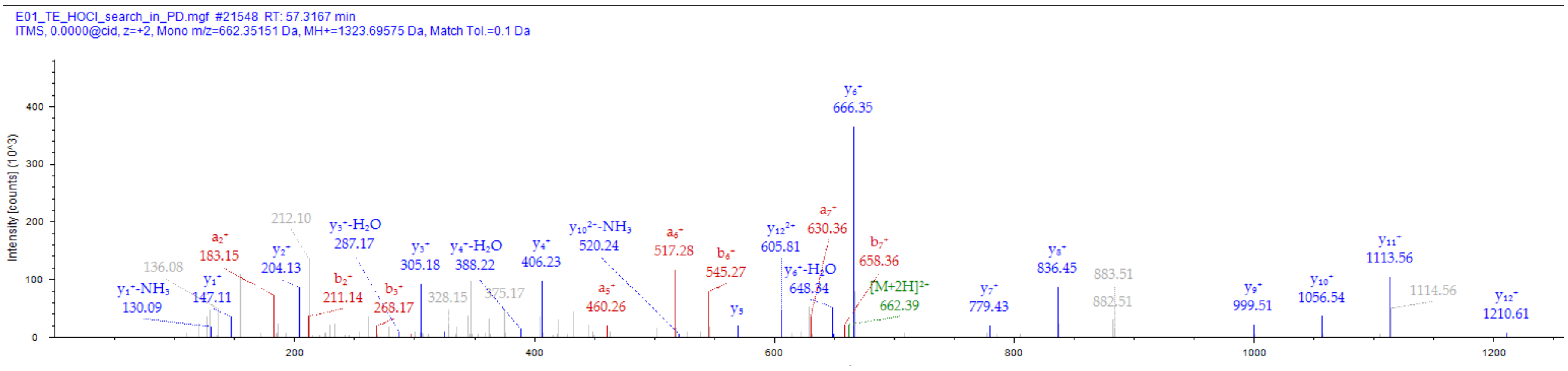

Ion Series | Neutral Losses | Precursor Ions

#1	a+	a2+	b+	b2+	Seq.	y+	y2+	#2
1	86.09643	43.55185	114.09134	57.54931	L			13
2	183.14919	92.07823	211.14410	106.07569	P	1210.61026	605.80877	12
3	240.17065	120.58897	268.16557	134.58642	G	1113.55750	557.28239	11
4	297.19212	149.09970	325.18703	163.09715	G	1056.53604	528.77166	10
5	460.25545	230.63136	488.25036	244.62882	Y	999.51457	500.26092	9
6	517.27691	259.14209	545.27182	273.13955	G	836.45124	418.72926	8
7	630.36097	315.68412	658.35589	329.68158	L	779.42978	390.21853	7
8	727.41374	364.21051	755.40865	378.20796	P	666.34572	333.67650	6
9	890.47707	445.74217	918.47198	459.73963	Y	569.29295	285.15011	5
10	991.52474	496.26601	1019.51966	510.26347	T	406.22962	203.61845	4
11	1092.57242	546.78985	1120.56734	560.78731	T	305.18195	153.09461	3
12	1149.59389	575.30058	1177.58880	589.29804	G	204.13427	102.57077	2
13					K	147.11280	74.06004	1

LPGGYGLPYTTGK
Y5-Chlorination (33.96103 Da) and Y9-Chlorination (33.96103 Da)

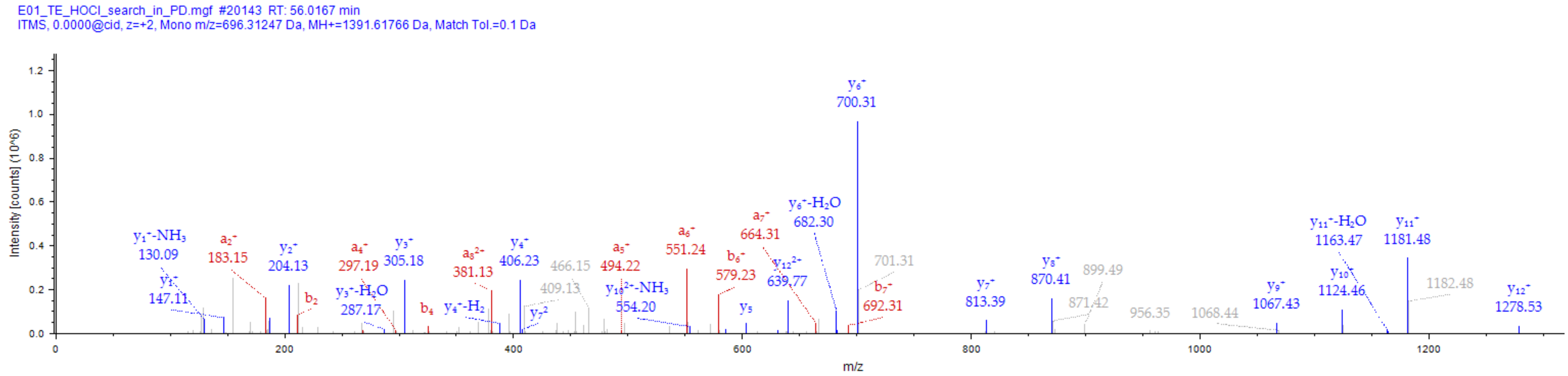

#1	a^+	a^{2+}	b^+	b^{2+}	Seq.	y^+	y^{2+}	#2
1	86.09643	43.55185	114.09134	57.54931	L			13
2	183.14919	92.07823	211.14410	106.07569	P	1278.53232	639.76980	12
3	240.17065	120.58897	268.16557	134.58642	G	1181.47956	591.24342	11
4	297.19212	149.09970	325.18703	163.09715	G	1124.45809	562.73268	10
5	494.21647	247.61188	522.21139	261.60933	Y-Chlorinat...	1067.43663	534.22195	9
6	551.23794	276.12261	579.23285	290.12006	G	870.41227	435.70977	8
7	664.32200	332.66464	692.31692	346.66210	L	813.39081	407.19904	7
8	761.37477	381.19102	789.36968	395.18848	P	700.30675	350.65701	6
9	958.39912	479.70320	986.39404	493.70066	Y-Chlorinat...	603.25398	302.13063	5
10	1059.44680	530.22704	1087.44171	544.22450	T	406.22962	203.61845	4
11	1160.49448	580.75088	1188.48939	594.74833	T	305.18195	153.09461	3
12	1217.51594	609.26161	1245.51086	623.25907	G	204.13427	102.57077	2
13					K	147.11280	74.06004	1

LPGGYGLPYTTGK

Y5-Chlorination (33.96103 Da)

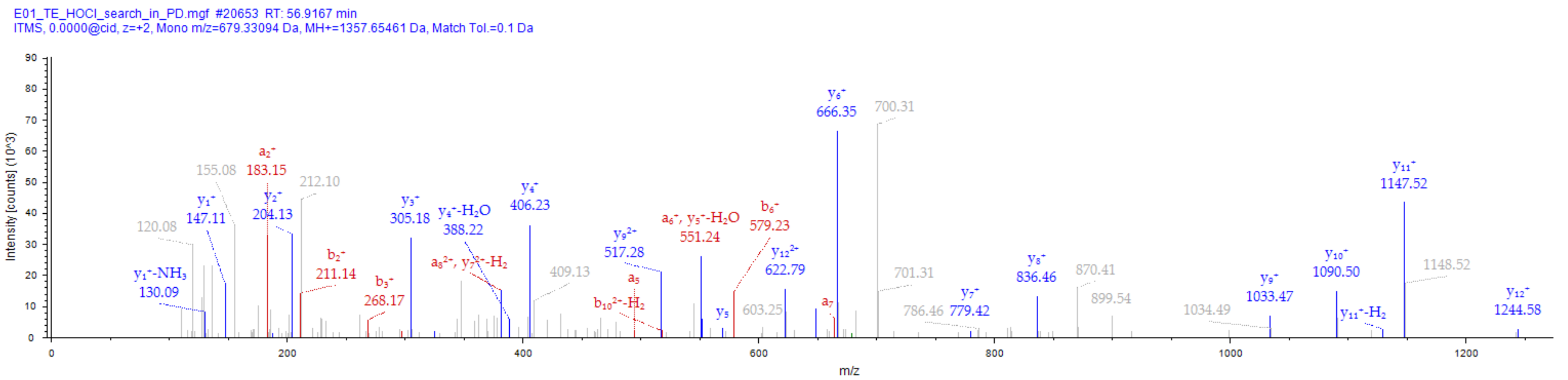

#1	a+	a2+	b+	b2+	Seq.	y+	y2+	#2
1	86.09643	43.55185	114.09134	57.54931	L			13
2	183.14919	92.07823	211.14410	106.07569	P	1244.57129	622.78928	12
3	240.17065	120.58897	268.16557	134.58642	G	1147.51853	574.26290	11
4	297.19212	149.09970	325.18703	163.09715	G	1090.49706	545.75217	10
5	494.21647	247.61188	522.21139	261.60933	Y-Chlorinat...	1033.47560	517.24144	9
6	551.23794	276.12261	579.23285	290.12006	G	836.45124	418.72926	8
7	664.32200	332.66464	692.31692	346.66210	L	779.42978	390.21853	7
8	761.37477	381.19102	789.36968	395.18848	P	666.34572	333.67650	6
9	924.43809	462.72269	952.43301	476.72014	Y	569.29295	285.15011	5
10	1025.48577	513.24652	1053.48069	527.24398	T	406.22962	203.61845	4
11	1126.53345	563.77036	1154.52836	577.76782	T	305.18195	153.09461	3
12	1183.55491	592.28110	1211.54983	606.27855	G	204.13427	102.57077	2
13					K	147.11280	74.06004	1

LPGGYGLPYTTGK

Y9-Chlorination (33.96103 Da)

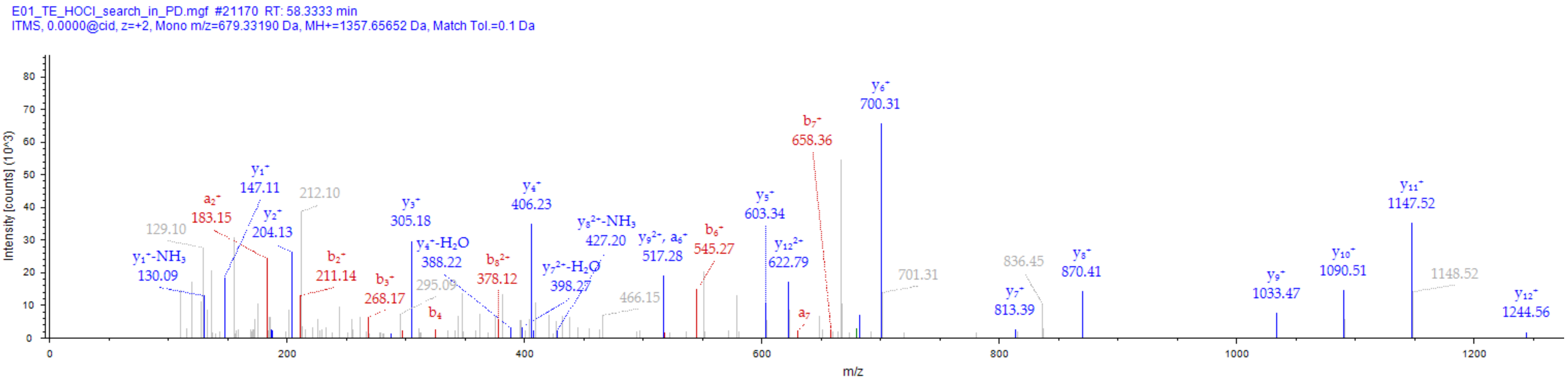

#1	a+	a2+	b+	b2+	Seq.	y+	y2+	#2
1	86.09643	43.55185	114.09134	57.54931	L			13
2	183.14919	92.07823	211.14410	106.07569	P	1244.57129	622.78928	12
3	240.17065	120.58897	268.16557	134.58642	G	1147.51853	574.26290	11
4	297.19212	149.09970	325.18703	163.09715	G	1090.49706	545.75217	10
5	460.25545	230.63136	488.25036	244.62882	Y	1033.47560	517.24144	9
6	517.27691	259.14209	545.27182	273.13955	G	870.41227	435.70977	8
7	630.36097	315.68412	658.35589	329.68158	L	813.39081	407.19904	7
8	727.41374	364.21051	755.40865	378.20796	P	700.30675	350.65701	6
9	924.43809	462.72269	952.43301	476.72014	Y-Chlorinat...	603.25398	302.13063	5
10	1025.48577	513.24652	1053.48069	527.24398	T	406.22962	203.61845	4
11	1126.53345	563.77036	1154.52836	577.76782	T	305.18195	153.09461	3
12	1183.55491	592.28110	1211.54983	606.27855	G	204.13427	102.57077	2
13					K	147.11280	74.06004	1

LPGGYGLPYTTGK

Y9-dichlorination (67.92206 Da)

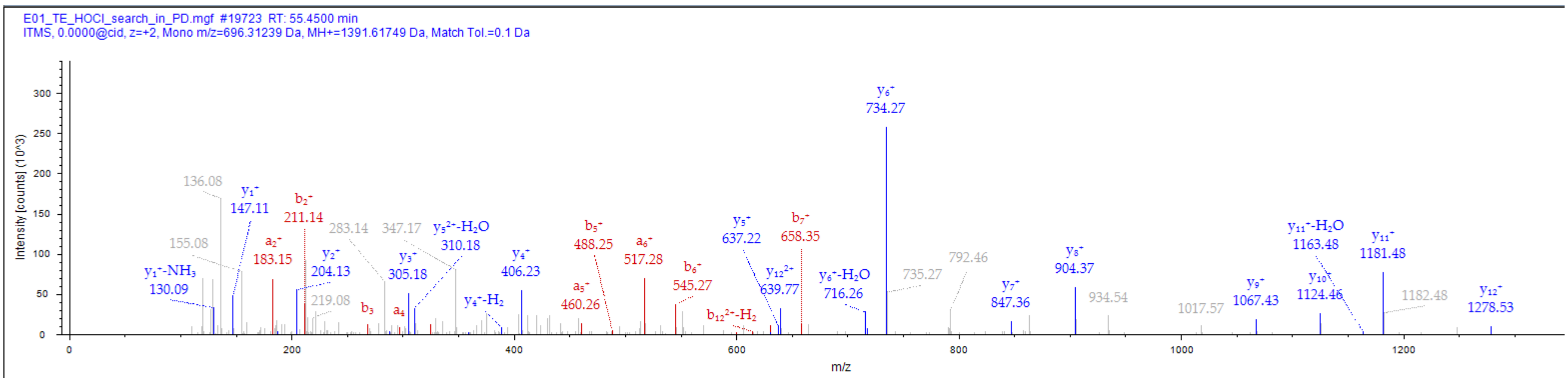

#1	a⁺	a²⁺	b⁺	b²⁺	Seq.	y⁺	y²⁺	#2
1	86.09643	43.55185	114.09134	57.54931	L			13
2	183.14919	92.07823	211.14410	106.07569	P	1278.53232	639.76980	12
3	240.17065	120.58897	268.16557	134.58642	G	1181.47956	591.24342	11
4	297.19212	149.09970	325.18703	163.09715	G	1124.45809	562.73268	10
5	460.25545	230.63136	488.25036	244.62882	Y	1067.43663	534.22195	9
6	517.27691	259.14209	545.27182	273.13955	G	904.37330	452.69029	8
7	630.36097	315.68412	658.35589	329.68158	L	847.35184	424.17956	7
8	727.41374	364.21051	755.40865	378.20796	P	734.26777	367.63752	6
9	958.39912	479.70320	986.39404	493.70066	Y-dichlorin...	637.21501	319.11114	5
10	1059.44680	530.22704	1087.44171	544.22450	T	406.22962	203.61845	4
11	1160.49448	580.75088	1188.48939	594.74833	T	305.18195	153.09461	3
12	1217.51594	609.26161	1245.51086	623.25907	G	204.13427	102.57077	2
13					K	147.11280	74.06004	1

LPYGYGPGGVAGAAGK

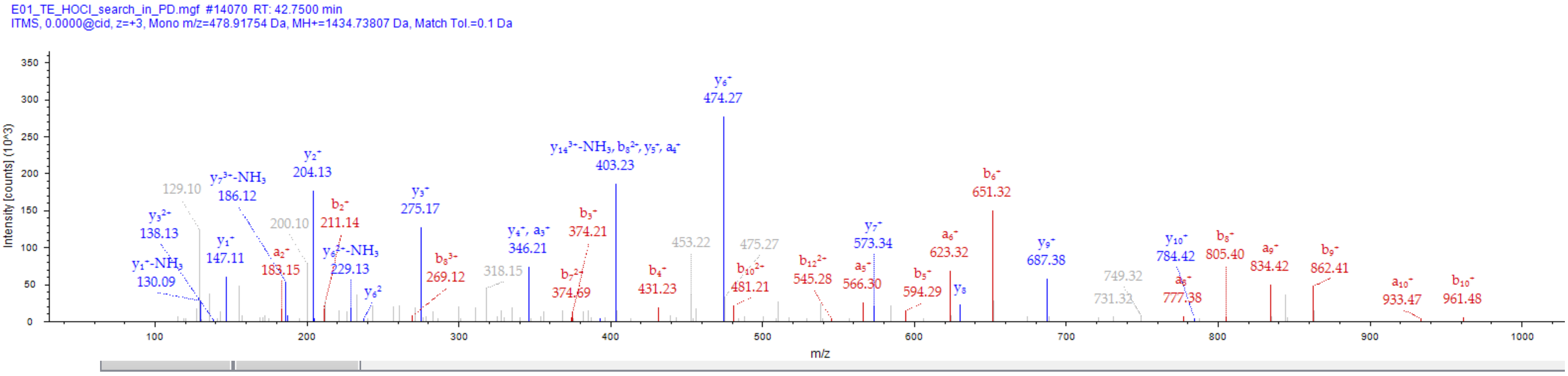

#1	a^+	a^{2+}	a^{3+}	b^+	b^{2+}	b^{3+}	Seq.	y^+	y^{2+}	y^{3+}	#2
1	86.09643	43.55185	29.37033	114.09134	57.54931	38.70196	L				16
2	183.14919	92.07823	61.72125	211.14410	106.07569	71.05289	P	1321.65353	661.33040	441.22269	15
3	346.21252	173.60990	116.07569	374.20743	187.60735	125.40733	Y	1224.60076	612.80402	408.87177	14
4	403.23398	202.12063	135.08285	431.22890	216.11809	144.41448	G	1061.53743	531.27236	354.51733	13
5	566.29731	283.65229	189.43729	594.29223	297.64975	198.76893	Y	1004.51597	502.76162	335.51017	12
6	623.31877	312.16303	208.44444	651.31369	326.16048	217.77608	G	841.45264	421.22996	281.15573	11
7	720.37154	360.68941	240.79536	748.36645	374.68686	250.12700	P	784.43118	392.71923	262.14858	10
8	777.39300	389.20014	259.80252	805.38792	403.19760	269.13416	G	687.37841	344.19285	229.79766	9
9	834.41447	417.71087	278.80967	862.40938	431.70833	288.14131	G	630.35695	315.68211	210.79050	8
10	933.48288	467.24508	311.83248	961.47779	481.24254	321.16412	V	573.33549	287.17138	191.78335	7
11	1004.51999	502.76363	335.51152	1032.51491	516.76109	344.84315	A	474.26707	237.63717	158.76054	6
12	1061.54146	531.27437	354.51867	1089.53637	545.27182	363.85031	G	403.22996	202.11862	135.08150	5
13	1132.57857	566.79292	378.19771	1160.57348	580.79038	387.52935	A	346.20850	173.60789	116.07435	4
14	1203.61568	602.31148	401.87675	1231.61060	616.30894	411.20838	A	275.17138	138.08933	92.39531	3
15	1260.63715	630.82221	420.88390	1288.63206	644.81967	430.21554	G	204.13427	102.57077	68.71627	2
16							K	147.11280	74.06004	49.70912	1

LPYGYGPGGVAGAAGK
Y3-Chlorination (33.96103 Da) and Y5-Chlorination (33.96103 Da)

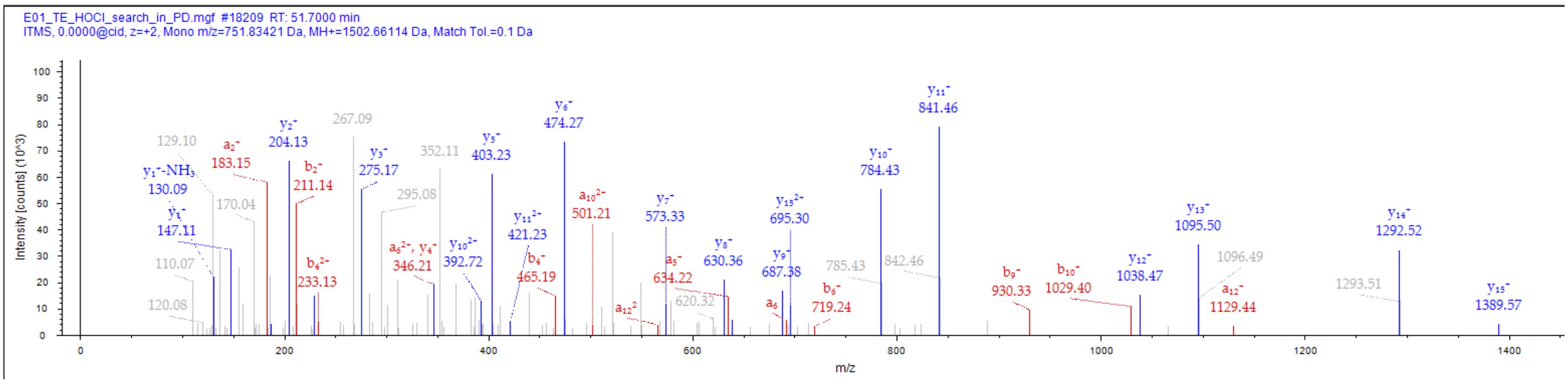

#1	a⁺	a²⁺	b⁺	b²⁺	Seq.	y⁺	y²⁺	#2
1	86.09643	43.55185	114.09134	57.54931	L			16
2	183.14919	92.07823	211.14410	106.07569	P	1389.57558	695.29143	15
3	380.17355	190.59041	408.16846	204.58787	Y-Chlorinat...	1292.52282	646.76505	14
4	437.19501	219.10114	465.18992	233.09860	G	1095.49846	548.25287	13
5	634.21937	317.61332	662.21428	331.61078	Y-Chlorinat...	1038.47700	519.74214	12
6	691.24083	346.12405	719.23574	360.12151	G	841.45264	421.22996	11
7	788.29359	394.65044	816.28851	408.64789	P	784.43118	392.71923	10
8	845.31506	423.16117	873.30997	437.15862	G	687.37841	344.19285	9
9	902.33652	451.67190	930.33144	465.66936	G	630.35695	315.68211	8
10	1001.40494	501.20611	1029.39985	515.20356	V	573.33549	287.17138	7
11	1072.44205	536.72466	1100.43696	550.72212	A	474.26707	237.63717	6
12	1129.46351	565.23539	1157.45843	579.23285	G	403.22996	202.11862	5
13	1200.50063	600.75395	1228.49554	614.75141	A	346.20850	173.60789	4
14	1271.53774	636.27251	1299.53265	650.26997	A	275.17138	138.08933	3
15	1328.55920	664.78324	1356.55412	678.78070	G	204.13427	102.57077	2
16					K	147.11280	74.06004	1

LPYGYGPGGVAGAAGK
Y3-Chlorination (33.96103 Da)

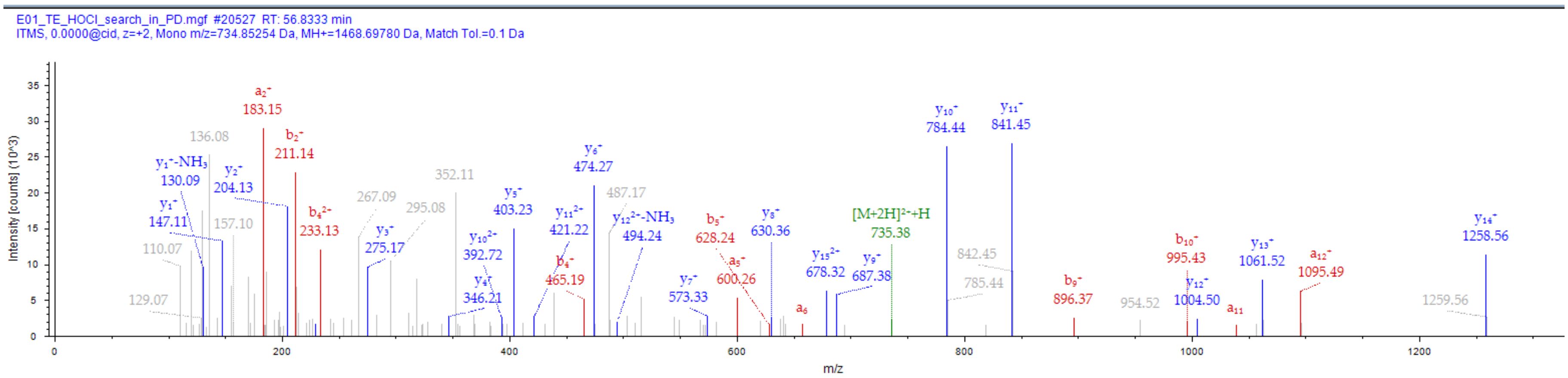

#1	a⁺	a²⁺	b⁺	b²⁺	Seq.	y⁺	y²⁺	#2
1	86.09643	43.55185	114.09134	57.54931	L			16
2	183.14919	92.07823	211.14410	106.07569	P	1355.61455	678.31092	15
3	380.17355	190.59041	408.16846	204.58787	Y-Chlorinat...	1258.56179	629.78453	14
4	437.19501	219.10114	465.18992	233.09860	G	1061.53743	531.27236	13
5	600.25834	300.63281	628.25325	314.63026	Y	1004.51597	502.76162	12
6	657.27980	329.14354	685.27472	343.14100	G	841.45264	421.22996	11
7	754.33257	377.66992	782.32748	391.66738	P	784.43118	392.71923	10
8	811.35403	406.18065	839.34894	420.17811	G	687.37841	344.19285	9
9	868.37549	434.69138	896.37041	448.68884	G	630.35695	315.68211	8
10	967.44391	484.22559	995.43882	498.22305	V	573.33549	287.17138	7
11	1038.48102	519.74415	1066.47594	533.74161	A	474.26707	237.63717	6
12	1095.50248	548.25488	1123.49740	562.25234	G	403.22996	202.11862	5
13	1166.53960	583.77344	1194.53451	597.77089	A	346.20850	173.60789	4
14	1237.57671	619.29199	1265.57163	633.28945	A	275.17138	138.08933	3
15	1294.59818	647.80273	1322.59309	661.80018	G	204.13427	102.57077	2
16					K	147.11280	74.06004	1

LPYGYGPGGVAGAAGK
Y3-dichlorination (67.92206 Da)

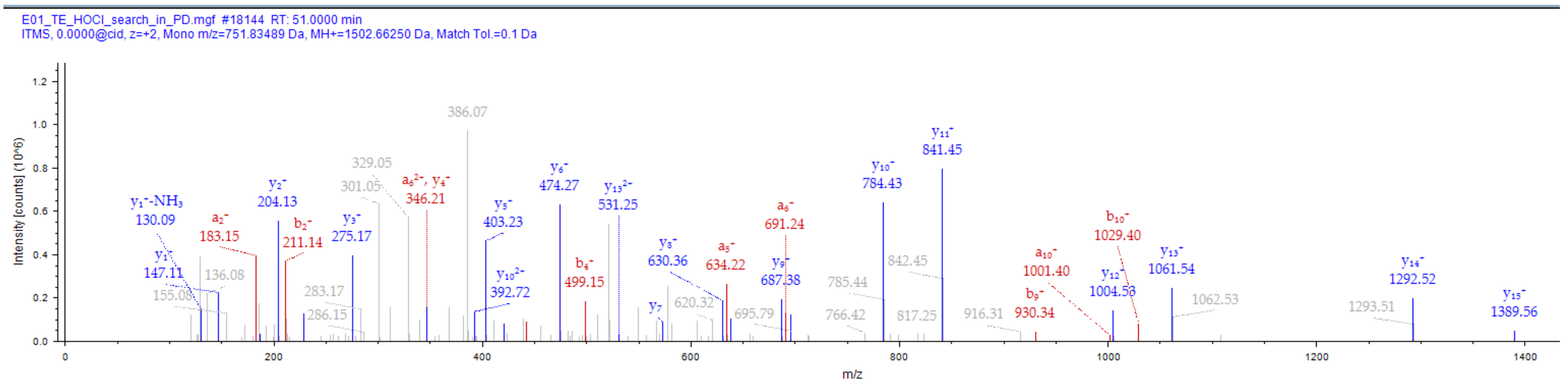

#1	a+	a2+	b+	b2+	Seq.	y+	y2+	#2
1	86.09643	43.55185	114.09134	57.54931	L			16
2	183.14919	92.07823	211.14410	106.07569	P	1389.57558	695.29143	15
3	414.13457	207.57092	442.12949	221.56838	Y-dichlorin...	1292.52282	646.76505	14
4	471.15604	236.08166	499.15095	250.07911	G	1061.53743	531.27236	13
5	634.21937	317.61332	662.21428	331.61078	Y	1004.51597	502.76162	12
6	691.24083	346.12405	719.23574	360.12151	G	841.45264	421.22996	11
7	788.29359	394.65043	816.28851	408.64789	P	784.43118	392.71923	10
8	845.31506	423.16117	873.30997	437.15862	G	687.37841	344.19285	9
9	902.33652	451.67190	930.33143	465.66936	G	630.35695	315.68211	8
10	1001.40493	501.20611	1029.39985	515.20356	V	573.33549	287.17138	7
11	1072.44205	536.72466	1100.43696	550.72212	A	474.26707	237.63717	6
12	1129.46351	565.23539	1157.45843	579.23285	G	403.22996	202.11862	5
13	1200.50063	600.75395	1228.49554	614.75141	A	346.20850	173.60789	4
14	1271.53774	636.27251	1299.53265	650.26997	A	275.17138	138.08933	3
15	1328.55920	664.78324	1356.55412	678.78070	G	204.13427	102.57077	2
16					K	147.11280	74.06004	1

LPYGYGPGGVAGAAGK
Y5-Chlorination (33.96103 Da)

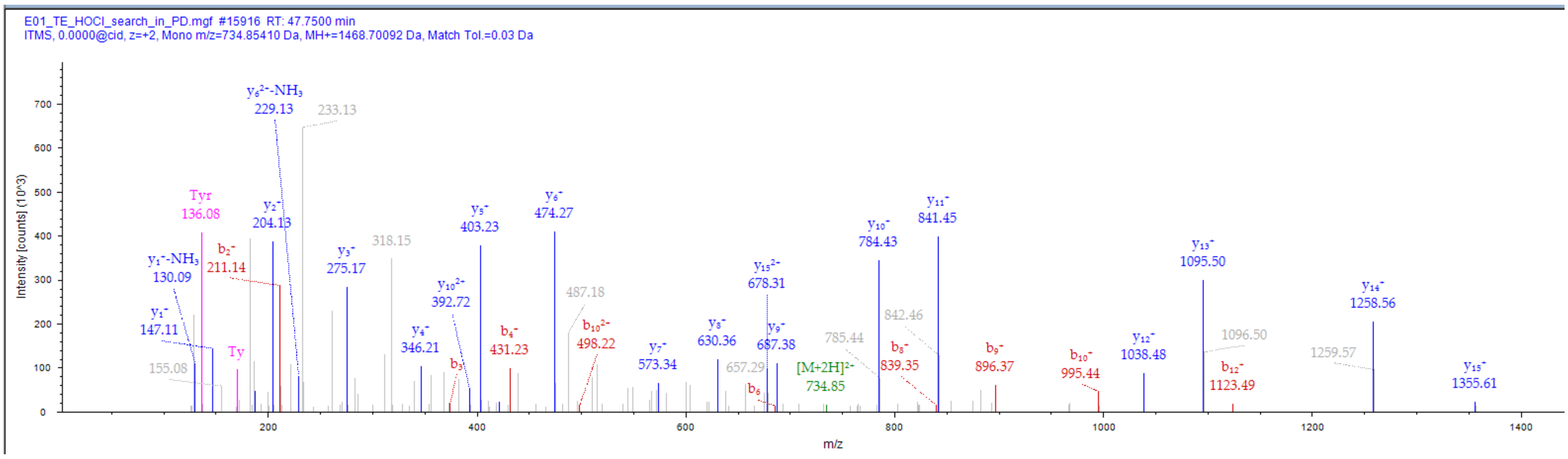

#1	Immonium	b^+	b^{2+}	Seq.	y^+	y^{2+}	#2
1	86.09643	114.09134	57.54931	L			16
2	70.06513	211.14410	106.07569	P	1355.61455	678.31092	15
3	136.07569	374.20743	187.60735	Y	1258.56179	629.78453	14
4	30.03383	431.22890	216.11809	G	1095.49846	548.25287	13
5	170.03672	628.25325	314.63026	Y-Chlorinat...	1038.47700	519.74214	12
6	30.03383	685.27472	343.14100	G	841.45264	421.22996	11
7	70.06513	782.32748	391.66738	P	784.43118	392.71923	10
8	30.03383	839.34894	420.17811	G	687.37841	344.19285	9
9	30.03383	896.37041	448.68884	G	630.35695	315.68211	8
10	72.08078	995.43882	498.22305	V	573.33549	287.17138	7
11	44.04948	1066.47594	533.74161	A	474.26707	237.63717	6
12	30.03383	1123.49740	562.25234	G	403.22996	202.11862	5
13	44.04948	1194.53451	597.77089	A	346.20850	173.60789	4
14	44.04948	1265.57163	633.28945	A	275.17138	138.08933	3
15	30.03383	1322.59309	661.80018	G	204.13427	102.57077	2
16	101.10732			K	147.11280	74.06004	1

LPYGYGPGGVAGAAGK

Y5-dichlorination (67.92206 Da)

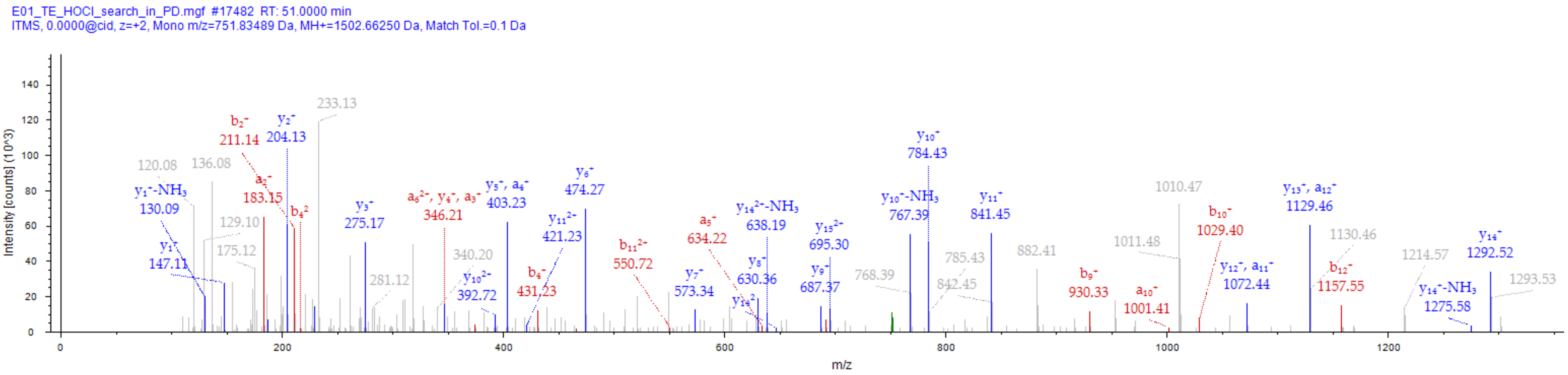

#1	a^+	a^{2+}	b^+	b^{2+}	Seq.	y^+	y^{2+}	#2
1	86.09643	43.55185	114.09134	57.54931	L			16
2	183.14919	92.07823	211.14410	106.07569	P	1389.57558	695.29143	15
3	346.21252	173.60990	374.20743	187.60735	Y	1292.52282	646.76505	14
4	403.23398	202.12063	431.22890	216.11809	G	1129.45949	565.23338	13
5	634.21937	317.61332	662.21428	331.61078	Y-dichlorin...	1072.43803	536.72265	12
6	691.24083	346.12405	719.23574	360.12151	G	841.45264	421.22996	11
7	788.29359	394.65043	816.28851	408.64789	P	784.43118	392.71923	10
8	845.31506	423.16117	873.30997	437.15862	G	687.37841	344.19285	9
9	902.33652	451.67190	930.33143	465.66936	G	630.35695	315.68211	8
10	1001.40493	501.20611	1029.39985	515.20356	V	573.33549	287.17138	7
11	1072.44205	536.72466	1100.43696	550.72212	A	474.26707	237.63717	6
12	1129.46351	565.23539	1157.45843	579.23285	G	403.22996	202.11862	5
13	1200.50063	600.75395	1228.49554	614.75141	A	346.20850	173.60789	4
14	1271.53774	636.27251	1299.53265	650.26997	A	275.17138	138.08933	3
15	1328.55920	664.78324	1356.55412	678.78070	G	204.13427	102.57077	2
16					K	147.11280	74.06004	1

LPYGYGPGGVAGAAGK

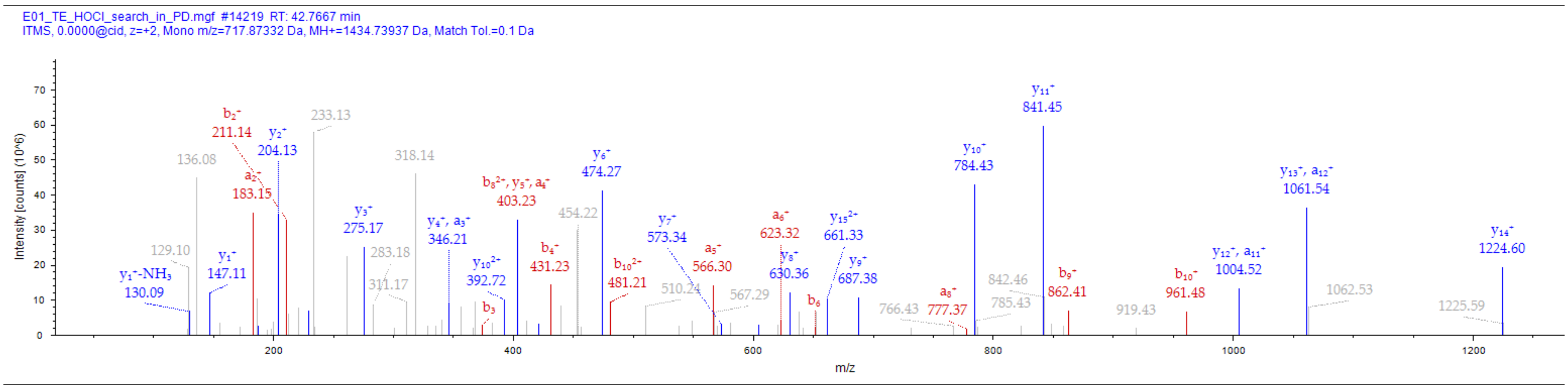

#1	a⁺	a²⁺	b⁺	b²⁺	Seq.	y⁺	y²⁺	#2
1	86.09643	43.55185	114.09134	57.54931	L			16
2	183.14919	92.07823	211.14410	106.07569	P	1321.65353	661.33040	15
3	346.21252	173.60990	374.20743	187.60735	Y	1224.60076	612.80402	14
4	403.23398	202.12063	431.22890	216.11809	G	1061.53743	531.27236	13
5	566.29731	283.65229	594.29223	297.64975	Y	1004.51597	502.76162	12
6	623.31877	312.16303	651.31369	326.16048	G	841.45264	421.22996	11
7	720.37154	360.68941	748.36645	374.68686	P	784.43118	392.71923	10
8	777.39300	389.20014	805.38792	403.19760	G	687.37841	344.19285	9
9	834.41447	417.71087	862.40938	431.70833	G	630.35695	315.68211	8
10	933.48288	467.24508	961.47779	481.24254	V	573.33549	287.17138	7
11	1004.51999	502.76363	1032.51491	516.76109	A	474.26707	237.63717	6
12	1061.54146	531.27437	1089.53637	545.27182	G	403.22996	202.11862	5
13	1132.57857	566.79292	1160.57348	580.79038	A	346.20850	173.60789	4
14	1203.61568	602.31148	1231.61060	616.30894	A	275.17138	138.08933	3
15	1260.63715	630.82221	1288.63206	644.81967	G	204.13427	102.57077	2
16					K	147.11280	74.06004	1

AGYPTGTGVGPQAAAAAAK

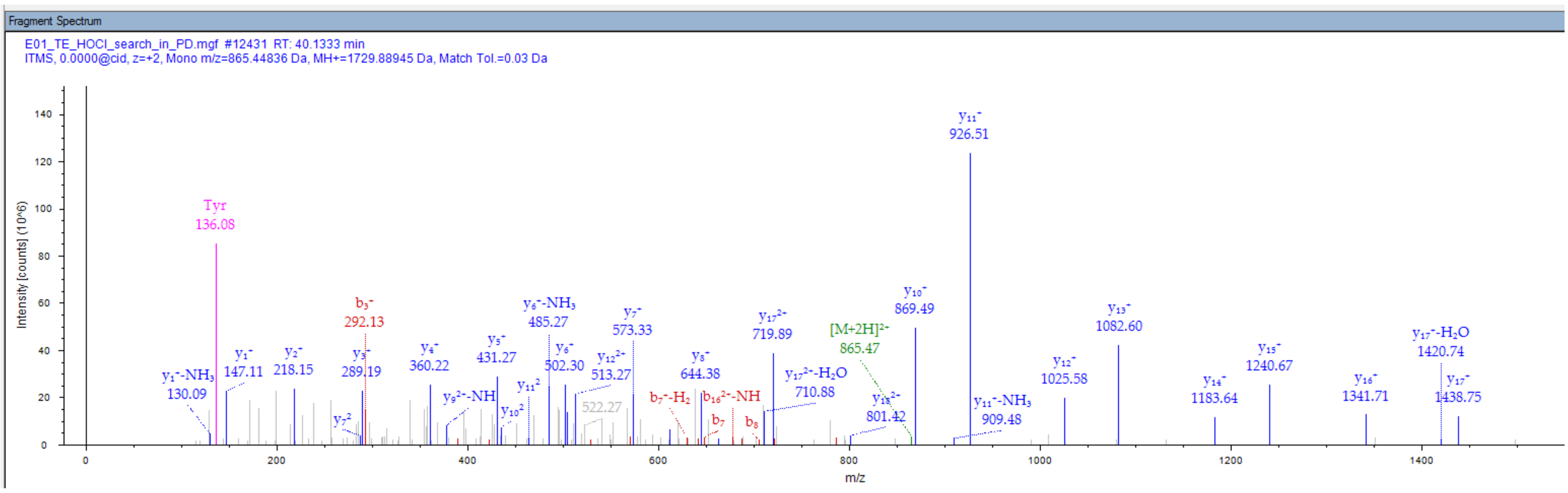

#1	Immonium	b^+	b^{2+}	Seq.	y^+	y^{2+}	#2
1	44.04948	72.04439	36.52583	A			20
2	30.03383	129.06585	65.03657	G	1658.84966	829.92847	19
3	136.07569	292.12918	146.56823	Y	1601.82820	801.41774	18
4	70.06513	389.18195	195.09461	P	1438.76487	719.88607	17
5	74.06004	490.22962	245.61845	T	1341.71210	671.35969	16
6	30.03383	547.25109	274.12918	G	1240.66443	620.83585	15
7	74.06004	648.29877	324.65302	T	1183.64296	592.32512	14
8	30.03383	705.32023	353.16375	G	1082.59528	541.80128	13
9	72.08078	804.38864	402.69796	V	1025.57382	513.29055	12
10	30.03383	861.41011	431.20869	G	926.50541	463.75634	11
11	70.06513	958.46287	479.73507	P	869.48394	435.24561	10
12	101.07094	1086.52145	543.76436	Q	772.43118	386.71923	9
13	44.04948	1157.55856	579.28292	A	644.37260	322.68994	8
14	44.04948	1228.59568	614.80148	A	573.33549	287.17138	7
15	44.04948	1299.63279	650.32003	A	502.29837	251.65282	6
16	44.04948	1370.66990	685.83859	A	431.26126	216.13427	5
17	44.04948	1441.70702	721.35715	A	360.22415	180.61571	4
18	44.04948	1512.74413	756.87570	A	289.18703	145.09715	3
19	44.04948	1583.78125	792.39426	A	218.14992	109.57860	2
20	101.10732			K	147.11280	74.06004	1

AGYPTGTGVGPQAAAAAAK, Y3-Chlorination (33.96103 Da)

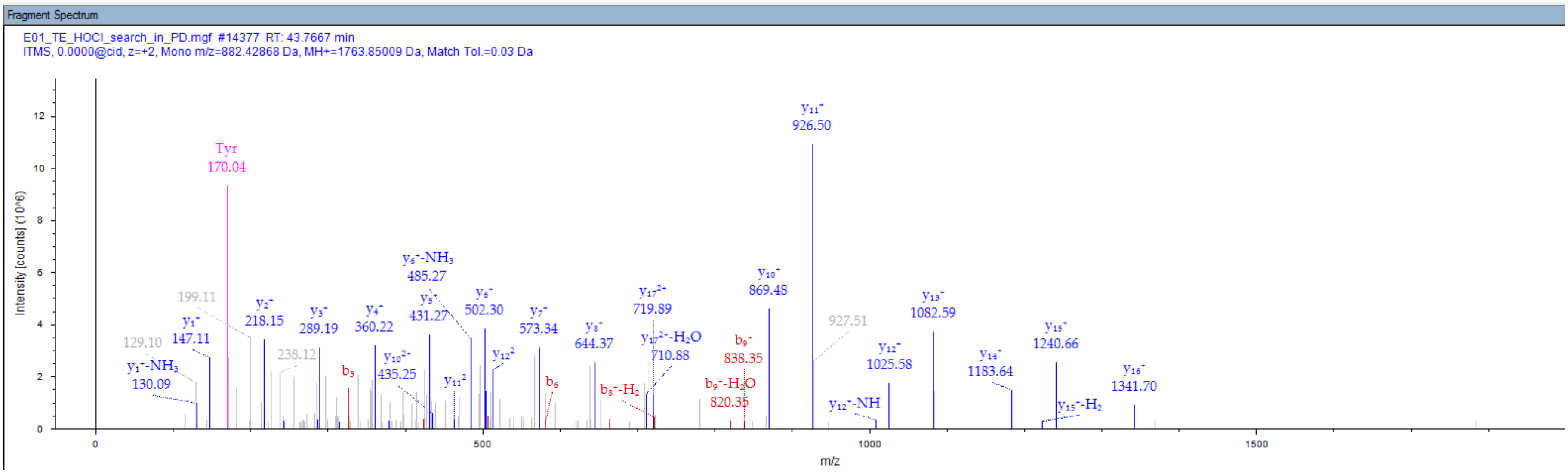

#1	Immonium	b⁺	b²⁺	Seq.	y⁺	y²⁺	#2
1	44.04948	72.04439	36.52583	A			20
2	30.03383	129.06585	65.03657	G	1692.81069	846.90898	19
3	170.03672	326.09021	163.54874	Y-Chlorinat...	1635.78922	818.39825	18
4	70.06513	423.14297	212.07513	P	1438.76487	719.88607	17
5	74.06004	524.19065	262.59896	T	1341.71210	671.35969	16
6	30.03383	581.21212	291.10970	G	1240.66443	620.83585	15
7	74.06004	682.25979	341.63354	T	1183.64296	592.32512	14
8	30.03383	739.28126	370.14427	G	1082.59528	541.80128	13
9	72.08078	838.34967	419.67847	V	1025.57382	513.29055	12
10	30.03383	895.37114	448.18921	G	926.50541	463.75634	11
11	70.06513	992.42390	496.71559	P	869.48394	435.24561	10
12	101.07094	1120.48248	560.74488	Q	772.43118	386.71923	9
13	44.04948	1191.51959	596.26343	A	644.37260	322.68994	8
14	44.04948	1262.55670	631.78199	A	573.33549	287.17138	7
15	44.04948	1333.59382	667.30055	A	502.29837	251.65282	6
16	44.04948	1404.63093	702.81910	A	431.26126	216.13427	5
17	44.04948	1475.66805	738.33766	A	360.22415	180.61571	4
18	44.04948	1546.70516	773.85622	A	289.18703	145.09715	3
19	44.04948	1617.74227	809.37478	A	218.14992	109.57860	2
20	101.10732			K	147.11280	74.06004	1

AGYPTGTGVGPQAAAAAAAK, Y3-dichlorination (67.92206 Da)

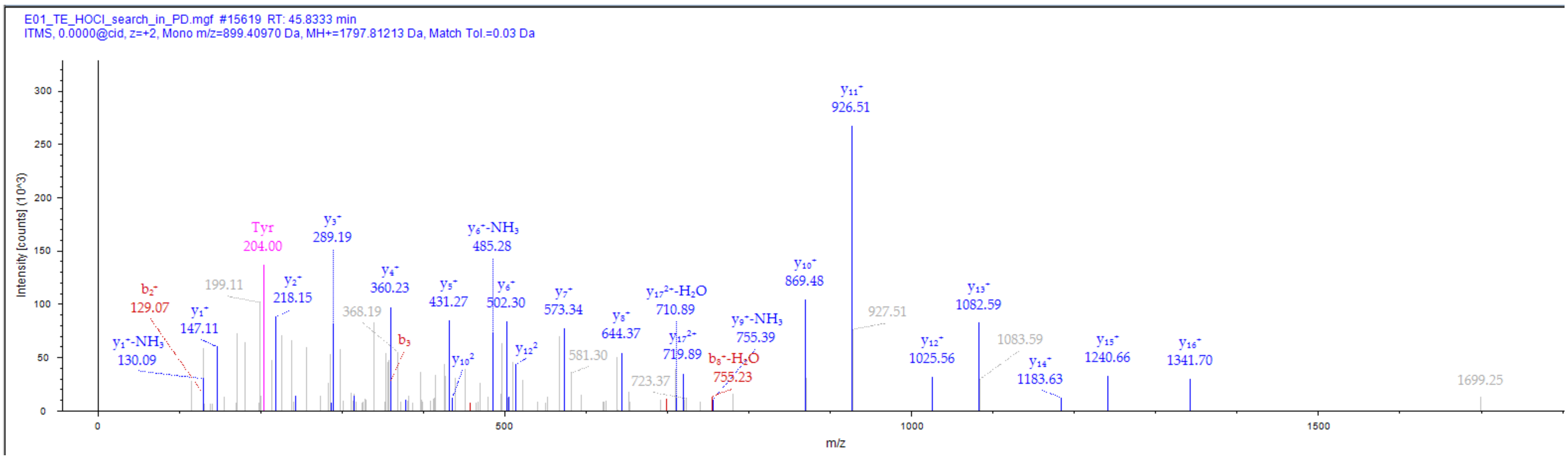

#1	Immonium	b+	b2+	Seq.	y+	y2+	#2
1	44.04948	72.04439	36.52583	A			20
2	30.03383	129.06585	65.03657	G	1726.77171	863.88950	19
3	203.99775	360.05124	180.52926	Y-dichlorin..	1669.75025	835.37876	18
4	70.06513	457.10400	229.05564	P	1438.76487	719.88607	17
5	74.06004	558.15168	279.57948	T	1341.71210	671.35969	16
6	30.03383	615.17314	308.09021	G	1240.66443	620.83585	15
7	74.06004	716.22082	358.61405	T	1183.64296	592.32512	14
8	30.03383	773.24229	387.12478	G	1082.59528	541.80128	13
9	72.08078	872.31070	436.65899	V	1025.57382	513.29055	12
10	30.03383	929.33216	465.16972	G	926.50541	463.75634	11
11	70.06513	1026.38493	513.69610	P	869.48394	435.24561	10
12	101.07094	1154.44350	577.72539	Q	772.43118	386.71923	9
13	44.04948	1225.48062	613.24395	A	644.37260	322.68994	8
14	44.04948	1296.51773	648.76250	A	573.33549	287.17138	7
15	44.04948	1367.55485	684.28106	A	502.29837	251.65282	6
16	44.04948	1438.59196	719.79962	A	431.26126	216.13427	5
17	44.04948	1509.62907	755.31817	A	360.22415	180.61571	4
18	44.04948	1580.66619	790.83673	A	289.18703	145.09715	3
19	44.04948	1651.70330	826.35529	A	218.14992	109.57860	2
20	101.10732			K	147.11280	74.06004	1

YGAAVPGVLGGLGALGGVGIPGGVVGA GPAAAAAAK

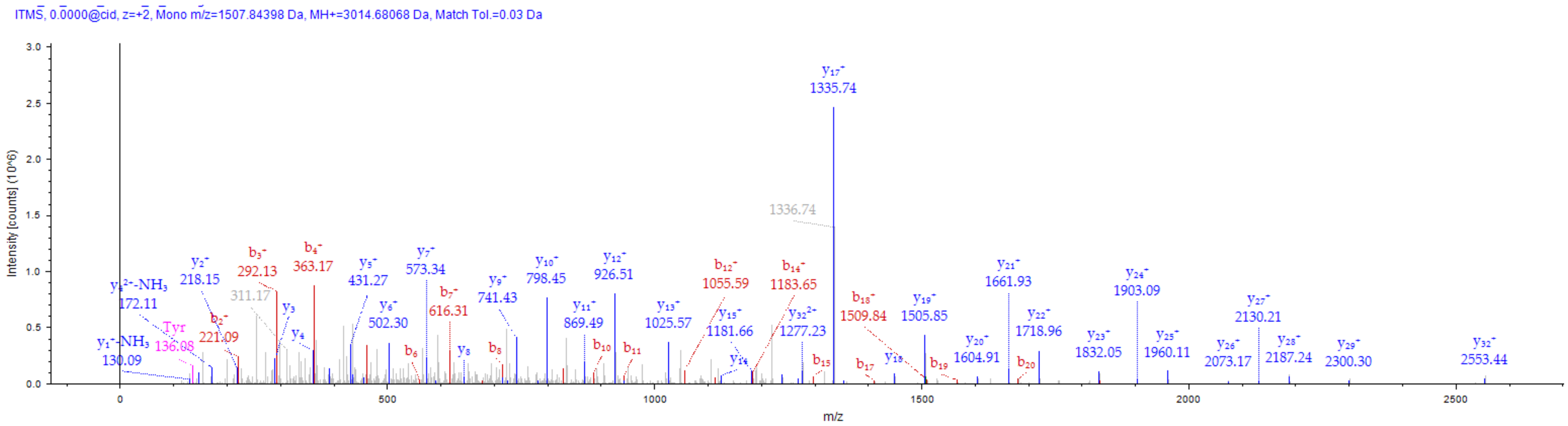

#1	Immonium	b⁺	b²⁺	Seq.	y⁺	y²⁺	#2
1	136.07569	164.07061	82.53894	Y			37
2	30.03383	221.09207	111.04967	G	2851.61524	1426.31126	36
3	44.04948	292.12918	146.56823	A	2794.59377	1397.80052	35
4	44.04948	363.16630	182.08679	A	2723.55666	1362.28197	34
5	72.08078	462.23471	231.62099	V	2652.51955	1326.76341	33
6	70.06513	559.28747	280.14738	P	2553.45113	1277.22920	32
7	30.03383	616.30894	308.65811	G	2456.39837	1228.70282	31
8	72.08078	715.37735	358.19231	V	2399.37690	1200.19209	30
9	86.09643	828.46142	414.73435	L	2300.30849	1150.65788	29
10	30.03383	885.48288	443.24508	G	2187.22443	1094.11585	28
11	30.03383	942.50434	471.75581	G	2130.20296	1065.60512	27
12	86.09643	1055.58841	528.29784	L	2073.18150	1037.09439	26
13	30.03383	1112.60987	556.80857	G	1960.09743	980.55236	25
14	44.04948	1183.64698	592.32713	A	1903.07597	952.04162	24
15	86.09643	1296.73105	648.86916	L	1832.03886	916.52307	23
16	30.03383	1353.75251	677.37989	G	1718.95479	859.98103	22
17	30.03383	1410.77398	705.89063	G	1661.93333	831.47030	21
18	72.08078	1509.84239	755.42483	V	1604.91187	802.95957	20
19	30.03383	1566.86385	783.93556	G	1505.84345	753.42536	19
20	86.09643	1679.94792	840.47760	I	1448.82199	724.91463	18
21	70.06513	1777.00068	889.00398	P	1335.73792	668.37260	17
22	30.03383	1834.02214	917.51471	G	1238.68516	619.84622	16
23	30.03383	1891.04361	946.02544	G	1181.66370	591.33549	15
24	72.08078	1990.11202	995.55965	V	1124.64223	562.82475	14
25	72.08078	2089.18044	1045.09386	V	1025.57382	513.29055	13
26	30.03383	2146.20190	1073.60459	G	926.50541	463.75634	12
27	44.04948	2217.23901	1109.12315	A	869.48394	435.24561	11
28	30.03383	2274.26048	1137.63388	G	798.44683	399.72705	10
29	70.06513	2371.31324	1186.16026	P	741.42536	371.21632	9
30	44.04948	2442.35035	1221.67882	A	644.37260	322.68994	8
31	44.04948	2513.38747	1257.19737	A	573.33549	287.17138	7
32	44.04948	2584.42458	1292.71593	A	502.29837	251.65282	6
33	44.04948	2655.46170	1328.23449	A	431.26126	216.13427	5
34	44.04948	2726.49881	1363.75304	A	360.22415	180.61571	4
35	44.04948	2797.53592	1399.27160	A	289.18703	145.09715	3
36	44.04948	2868.57304	1434.79016	A	218.14992	109.57860	2
37	101.10732			K	147.11280	74.06004	1

YGAAVPGVLGGLGALGGVGIPGGVVGAGPAAAAAAAK, Y1-Chlorination (33.96103 Da)

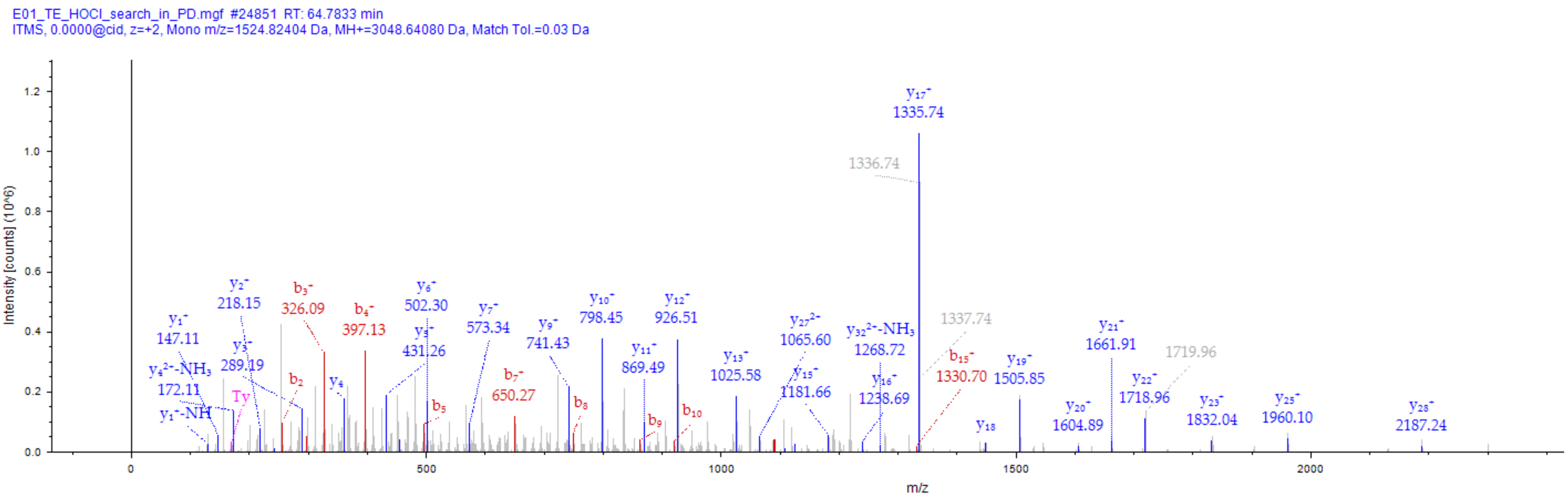

#1	Immonium	b⁺	b²⁺	Seq.	y⁺	y²⁺	#2
1	170.03672	198.03163	99.51945	Y-Chlorinat...			37
2	30.03383	255.05310	128.03019	G	2851.61524	1426.31126	36
3	44.04948	326.09021	163.54874	A	2794.59377	1397.80052	35
4	44.04948	397.12732	199.06730	A	2723.55666	1362.28197	34
5	72.08078	496.19574	248.60151	V	2652.51955	1326.76341	33
6	70.06513	593.24850	297.12789	P	2553.45113	1277.22920	32
7	30.03383	650.26997	325.63862	G	2456.39837	1228.70282	31
8	72.08078	749.33838	375.17283	V	2399.37690	1200.19209	30
9	86.09643	862.42244	431.71486	L	2300.30849	1150.65788	29
10	30.03383	919.44391	460.22559	G	2187.22443	1094.11585	28
11	30.03383	976.46537	488.73632	G	2130.20296	1065.60512	27
12	86.09643	1089.54943	545.27836	L	2073.18150	1037.09439	26
13	30.03383	1146.57090	573.78909	G	1960.09743	980.55236	25
14	44.04948	1217.60801	609.30764	A	1903.07597	952.04162	24
15	86.09643	1330.69208	665.84968	L	1832.03886	916.52307	23
16	30.03383	1387.71354	694.36041	G	1718.95479	859.98103	22
17	30.03383	1444.73500	722.87114	G	1661.93333	831.47030	21
18	72.08078	1543.80342	772.40535	V	1604.91187	802.95957	20
19	30.03383	1600.82488	800.91608	G	1505.84345	753.42536	19
20	86.09643	1713.90895	857.45811	I	1448.82199	724.91463	18
21	70.06513	1810.96171	905.98449	P	1335.73792	668.37260	17
22	30.03383	1867.98317	934.49522	G	1238.68516	619.84622	16
23	30.03383	1925.00464	963.00596	G	1181.66370	591.33549	15
24	72.08078	2024.07305	1012.54016	V	1124.64223	562.82475	14
25	72.08078	2123.14146	1062.07437	V	1025.57382	513.29055	13
26	30.03383	2180.16293	1090.58510	G	926.50541	463.75634	12
27	44.04948	2251.20004	1126.10366	A	869.48394	435.24561	11
28	30.03383	2308.22151	1154.61439	G	798.44683	399.72705	10
29	70.06513	2405.27427	1203.14077	P	741.42536	371.21632	9
30	44.04948	2476.31138	1238.65933	A	644.37260	322.68994	8
31	44.04948	2547.34850	1274.17789	A	573.33549	287.17138	7
32	44.04948	2618.38561	1309.69644	A	502.29837	251.65282	6
33	44.04948	2689.42272	1345.21500	A	431.26126	216.13427	5
34	44.04948	2760.45984	1380.73356	A	360.22415	180.61571	4
35	44.04948	2831.49695	1416.25211	A	289.18703	145.09715	3
36	44.04948	2902.53407	1451.77067	A	218.14992	109.57860	2
37	101.10732			K	147.11280	74.06004	1

YGLVPGVGVAPGVGVAPGVGVAPGVGLAPGVGVAPGVGVAPGVGVAPGIGPGGVAAAAK, Y1-Chlorination (33.96103 Da)

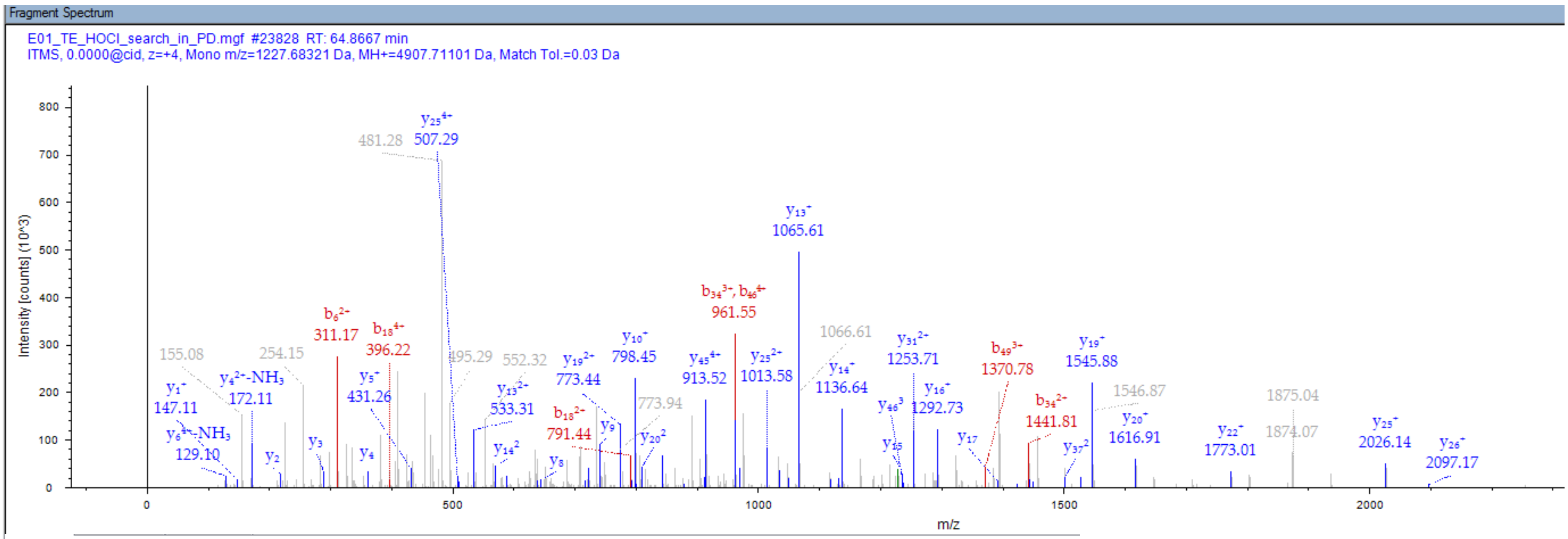

#1	Immonium	b+	b2+	b3+	b4+	Seq.	y+	y2+	y3+	y4+	#2
1	170.03672	198.03163	99.51945	66.68206	50.26337	Y-Chlorinat...					59
2	30.03383	255.05310	128.03019	85.68922	64.51873	G	4710.68214	2355.84471	1570.89890	1178.42599	58
3	86.09643	368.13716	184.57222	123.38390	92.78975	L	4653.66068	2327.33398	1551.89174	1164.17063	57
4	72.08078	467.20557	234.10643	156.40671	117.55685	V	4540.57661	2270.79194	1514.19706	1135.89961	56
5	70.06513	564.25834	282.63281	188.75763	141.82004	P	4441.50820	2221.25774	1481.17425	1111.13251	55
6	30.03383	621.27980	311.14354	207.76479	156.07541	G	4344.45543	2172.73136	1448.82333	1086.86932	54
7	72.08078	720.34822	360.67775	240.78759	180.84251	V	4287.43397	2144.22062	1429.81617	1072.61395	53
8	30.03383	777.36968	389.18848	259.79474	195.09788	G	4188.36556	2094.68642	1396.79337	1047.84685	52
9	72.08078	876.43809	438.72269	292.81755	219.86498	V	4131.34409	2066.17568	1377.78622	1033.59148	51
10	44.04948	947.47521	474.24124	316.49659	237.62426	A	4032.27568	2016.64148	1344.76341	1008.82438	50
11	70.06513	1044.52797	522.76762	348.84751	261.88745	P	3961.23857	1981.12292	1321.08437	991.06510	49
12	30.03383	1101.54943	551.27836	367.85466	276.14282	G	3864.18580	1932.59654	1288.73345	966.80191	48
13	72.08078	1200.61785	600.81256	400.87747	300.90992	V	3807.16434	1904.08581	1269.72630	952.54654	47
14	30.03383	1257.63931	629.32329	419.88462	315.16529	G	3708.09592	1854.55160	1236.70349	927.77944	46
15	72.08078	1356.70773	678.85750	452.90743	339.93239	V	3651.07446	1826.04087	1217.69634	913.52407	45
16	44.04948	1427.74484	714.37606	476.58646	357.69167	A	3552.00605	1776.50666	1184.67353	888.75697	44
17	70.06513	1524.79760	762.90244	508.93739	381.95486	P	3480.96893	1740.98810	1160.99450	870.99769	43
18	30.03383	1581.81907	791.41317	527.94454	396.21022	G	3383.91617	1692.46172	1128.64357	846.73450	42
19	72.08078	1680.88748	840.94738	560.96734	420.97733	V	3326.89471	1663.95099	1109.63642	832.47913	41
20	30.03383	1737.90895	869.45811	579.97450	435.23269	G	3227.82629	1614.41678	1076.61361	807.71203	40
21	72.08078	1836.97736	918.99232	612.99730	459.99980	V	3170.80483	1585.90605	1057.60646	793.45666	39
22	44.04948	1908.01447	954.51087	636.67634	477.75908	A	3071.73641	1536.37185	1024.58366	768.68956	38
23	70.06513	2005.06724	1003.03726	669.02726	502.02227	P	3000.69930	1500.85329	1000.90462	750.93028	37
24	30.03383	2062.08870	1031.54799	688.03442	516.27763	G	2903.64654	1452.32691	968.55370	726.66709	36
25	72.08078	2161.15711	1081.08220	721.05722	541.04474	V	2846.62507	1423.81617	949.54654	712.41173	35
26	30.03383	2218.17858	1109.59293	740.06438	555.30010	G	2747.55666	1374.28197	916.52374	687.64462	34
27	86.09643	2331.26264	1166.13496	777.75906	583.57112	L	2690.53520	1345.77124	897.51658	673.38926	33
28	44.04948	2402.29976	1201.65352	801.43810	601.33040	A	2577.45113	1289.22920	859.82189	645.11824	32
29	70.06513	2499.35252	1250.17990	833.78902	625.59359	P	2506.41402	1253.71065	836.14286	627.35896	31
30	30.03383	2556.37398	1278.69063	852.79618	639.84895	G	2409.36125	1205.18427	803.79194	603.09577	30
31	72.08078	2655.44240	1328.22484	885.81898	664.61606	V	2352.33979	1176.67353	784.78478	588.84040	29
32	30.03383	2712.46386	1356.73557	904.82614	678.87142	G	2253.27138	1127.13933	751.76198	564.07330	28
33	72.08078	2811.53227	1406.26978	937.84894	703.63853	V	2196.24991	1098.62859	732.75482	549.81794	27
34	44.04948	2882.56939	1441.78833	961.52798	721.39780	A	2097.18150	1049.09439	699.73202	525.05083	26
35	70.06513	2979.62215	1490.31471	993.87890	745.66100	P	2026.14438	1013.57583	676.05298	507.29155	25
36	30.03383	3036.64362	1518.82545	1012.88606	759.91636	G	1929.09162	965.04945	643.70206	483.02836	24
37	72.08078	3135.71203	1568.35965	1045.90886	784.68346	V	1872.07016	936.53872	624.69490	468.77300	23
38	30.03383	3192.73349	1596.87038	1064.91602	798.93883	G	1773.00174	887.00451	591.67210	444.00589	22
39	72.08078	3291.80191	1646.40459	1097.93882	823.70593	V	1715.98028	858.49378	572.66494	429.75053	21
40	44.04948	3362.83902	1681.92315	1121.61786	841.46521	A	1616.91187	808.95957	539.64214	404.98342	20
41	70.06513	3459.89178	1730.44953	1153.96878	865.72840	P	1545.87475	773.44101	515.96310	387.22415	19
42	30.03383	3516.91325	1758.96026	1172.97593	879.98377	G	1448.82199	724.91463	483.61218	362.96095	18
43	72.08078	3615.98166	1808.49447	1205.99874	904.75087	V	1391.80052	696.40390	464.60503	348.70559	17
44	30.03383	3673.00313	1837.00520	1225.00589	919.00624	G	1292.73211	646.86969	431.58222	323.93849	16
45	72.08078	3772.07154	1886.53941	1258.02870	943.77334	V	1235.71065	618.35896	412.57507	309.68312	15
46	44.04948	3843.10865	1922.05797	1281.70774	961.53262	A	1136.64223	568.82475	379.55226	284.91602	14
47	70.06513	3940.16142	1970.58435	1314.05866	985.79581	P	1065.60512	533.30620	355.87322	267.15674	13
48	30.03383	3997.18288	1999.09508	1333.06581	1000.05118	G	968.55236	484.77982	323.52230	242.89355	12
49	86.09643	4110.26694	2055.63711	1370.76050	1028.32219	I	911.53089	456.26908	304.51515	228.63818	11
50	30.03383	4167.28841	2084.14784	1389.76765	1042.57756	G	798.44683	399.72705	266.82046	200.36716	10
50	30.03383	4167.28841	2084.14784	1389.76765	1042.57756	G	798.44683	399.72705	266.82046	200.36716	10
51	70.06513	4264.34117	2132.67422	1422.11858	1066.84075	P	741.42536	371.21632	247.81331	186.11180	9
52	30.03383	4321.36264	2161.18496	1441.12573	1081.09612	G	644.37260	322.68994	215.46238	161.84861	8
53	30.03383	4378.38410	2189.69569	1460.13288	1095.35148	G	587.35114	294.17921	196.45523	147.59324	7
54	72.08078	4477.45251	2239.22990	1493.15569	1120.11859	V	530.32967	265.66847	177.44808	133.33788	6
55	44.04948	4548.48963	2274.74845	1516.83473	1137.87786	A	431.26126	216.13427	144.42527	108.57077	5
56	44.04948	4619.52674	2310.26701	1540.51376	1155.63714	A	360.22415	180.61571	120.74623	90.81149	4
57	44.04948	4690.56385	2345.78557	1564.19280	1173.39642	A	289.18703	145.09715	97.06720	73.05222	3
58	44.04948	4761.60097	2381.30412	1587.87184	1191.15570	A	218.14992	109.57860	73.38816	55.29294	2
59	101.10732					K	147.11280	74.06004	49.70912	37.53366	1

YGVAARPGFGLSPIFPGGACLGKACGR, C20-Oxidation (15.99492 Da), Y1-Chlorination (33.96103 Da)

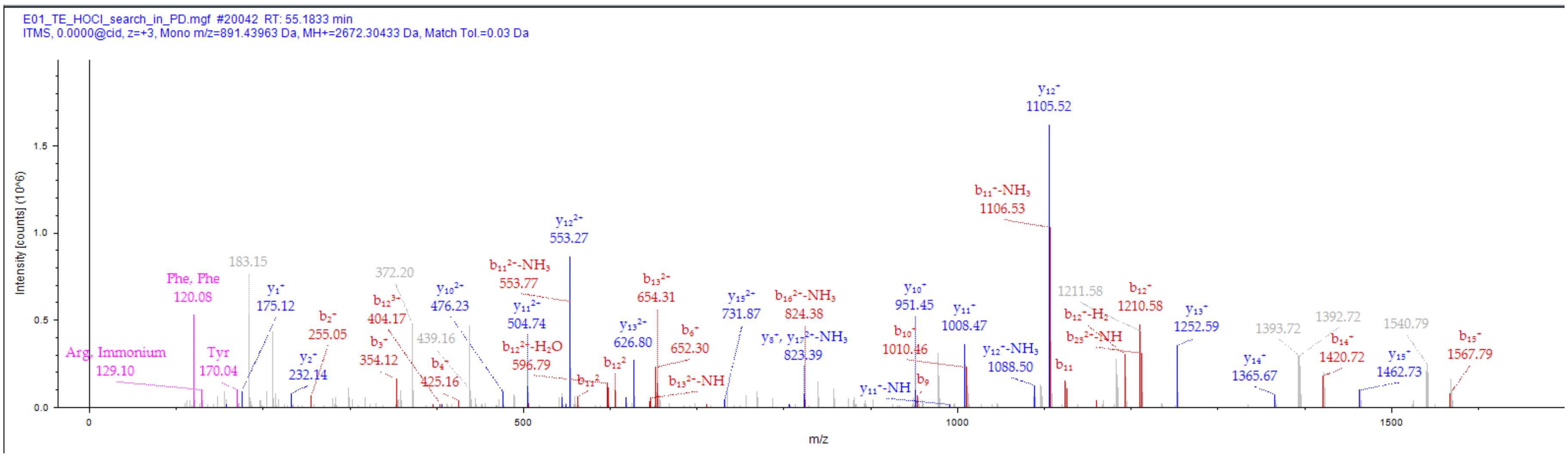

#1	Immonium	b⁺	b²⁺	b³⁺	Seq.	y⁺	y²⁺	y³⁺	#2
1	170.03672	198.03163	99.51945	66.68206	Y-Chlorinat...				27
2	30.03383	255.05310	128.03019	85.68922	G	2475.27476	1238.14102	825.76311	26
3	72.08078	354.12151	177.56439	118.71202	V	2418.25330	1209.63029	806.75595	25
4	44.04948	425.15862	213.08295	142.39106	A	2319.18489	1160.09608	773.73315	24
5	44.04948	496.19574	248.60151	166.07010	A	2248.14777	1124.57752	750.05411	23
6	129.11347	652.29685	326.65206	218.10380	R	2177.11066	1089.05897	726.37507	22
7	70.06513	749.34961	375.17844	250.45472	P	2021.00955	1011.00841	674.34137	21
8	30.03383	806.37108	403.68918	269.46188	G	1923.95678	962.48203	641.99045	20
9	120.08078	953.43949	477.22338	318.48468	F	1866.93532	933.97130	622.98329	19
10	30.03383	1010.46095	505.73412	337.49184	G	1719.86691	860.43709	573.96049	18
11	86.09643	1123.54502	562.27615	375.18652	L	1662.84544	831.92636	554.95333	17
12	60.04439	1210.57705	605.79216	404.19720	S	1549.76138	775.38433	517.25864	16
13	70.06513	1307.62981	654.31854	436.54812	P	1462.72935	731.86831	488.24797	15
14	86.09643	1420.71387	710.86058	474.24281	I	1365.67659	683.34193	455.89705	14
15	120.08078	1567.78229	784.39478	523.26561	F	1252.59252	626.79990	418.20236	13
16	70.06513	1664.83505	832.92116	555.61654	P	1105.52411	553.26569	369.17955	12
17	30.03383	1721.85652	861.43190	574.62369	G	1008.47135	504.73931	336.82863	11
18	30.03383	1778.87798	889.94263	593.63084	G	951.44988	476.22858	317.82148	10
19	44.04948	1849.91509	925.46118	617.30988	A	894.42842	447.71785	298.81432	9
20	92.01646	1968.91919	984.96323	656.97792	C-Oxidation	823.39130	412.19929	275.13529	8
21	86.09643	2082.00326	1041.50527	694.67260	L	704.38720	352.69724	235.46725	7
22	30.03383	2139.02472	1070.01600	713.67976	G	591.30314	296.15521	197.77256	6
23	101.10732	2267.11968	1134.06348	756.37808	K	534.28168	267.64448	178.76541	5
24	44.04948	2338.15680	1169.58204	780.05712	A	406.18671	203.59700	136.06709	4
25	76.02155	2441.16598	1221.08663	814.39351	C	335.14960	168.07844	112.38805	3
26	30.03383	2498.18744	1249.59736	833.40067	G	232.14042	116.57385	78.05166	2
27	129.11347				R	175.11895	88.06311	59.04450	1

YGVAARPGFGLSPIFPGGACLGKACGRK, C20-Oxidation (15.99492 Da), C25-Trioxidation (47.98474 Da)

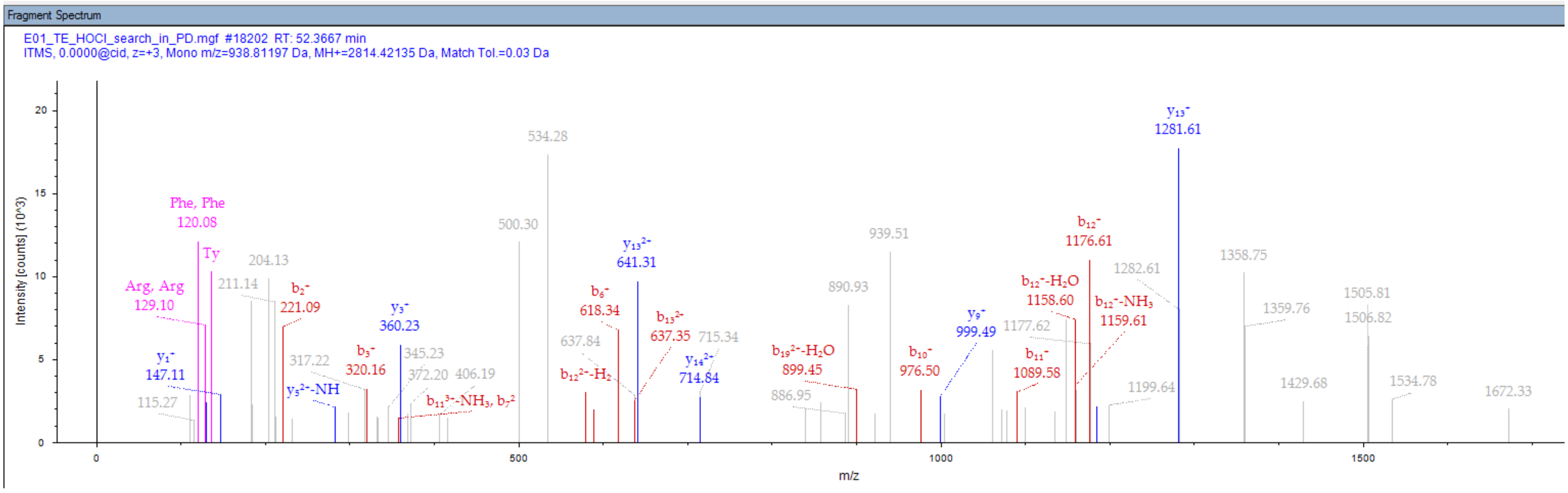

#1	Immonium	b+	b2+	b3+	Seq.	y+	y2+	y3+	#2
1	136.07569	164.07061	82.53894	55.36172	Y				28
2	30.03383	221.09207	111.04967	74.36887	G	2651.35447	1326.18087	884.45634	27
3	72.08078	320.16048	160.58388	107.39168	V	2594.33301	1297.67014	865.44919	26
4	44.04948	391.19760	196.10244	131.07072	A	2495.26459	1248.13594	832.42638	25
5	44.04948	462.23471	231.62099	154.74975	A	2424.22748	1212.61738	808.74734	24
6	129.11347	618.33582	309.67155	206.78346	R	2353.19037	1177.09882	785.06831	23
7	70.06513	715.38858	358.19793	239.13438	P	2197.08926	1099.04827	733.03460	22
8	30.03383	772.41005	386.70866	258.14153	G	2100.03649	1050.52188	700.68368	21
9	120.08078	919.47846	460.24287	307.16434	F	2043.01503	1022.01115	681.67653	20
10	30.03383	976.49993	488.75360	326.17149	G	1895.94661	948.47695	632.65372	19
11	86.09643	1089.58399	545.29563	363.86618	L	1838.92515	919.96621	613.64657	18
12	60.04439	1176.61602	588.81165	392.87686	S	1725.84109	863.42418	575.95188	17
13	70.06513	1273.66878	637.33803	425.22778	P	1638.80906	819.90817	546.94120	16
14	86.09643	1386.75285	693.88006	462.92247	I	1541.75629	771.38179	514.59028	15
15	120.08078	1533.82126	767.41427	511.94527	F	1428.67223	714.83975	476.89559	14
16	70.06513	1630.87402	815.94065	544.29619	P	1281.60382	641.30555	427.87279	13
17	30.03383	1687.89549	844.45138	563.30335	G	1184.55105	592.77916	395.52187	12
18	30.03383	1744.91695	872.96211	582.31050	G	1127.52959	564.26843	376.51471	11
19	44.04948	1815.95407	908.48067	605.98954	A	1070.50812	535.75770	357.50756	10
20	92.01646	1934.95816	967.98272	645.65757	C-Oxidation	999.47101	500.23914	333.82852	9
21	86.09643	2048.04223	1024.52475	683.35226	L	880.46691	440.73709	294.16049	8
22	30.03383	2105.06369	1053.03548	702.35942	G	767.38285	384.19506	256.46580	7
23	101.10732	2233.15866	1117.08297	745.05774	K	710.36138	355.68433	237.45865	6
24	44.04948	2304.19577	1152.60152	768.73677	A	582.26642	291.63685	194.76032	5
25	124.00629	2455.18970	1228.09849	819.06808	C-Trioxidat..	511.22931	256.11829	171.08129	4
26	30.03383	2512.21116	1256.60922	838.07524	G	360.23538	180.62133	120.74998	3
27	129.11347	2668.31227	1334.65977	890.10894	R	303.21392	152.11060	101.74282	2
28	101.10732				K	147.11280	74.06004	49.70912	1

YGVAARPGFGLSPIFPGGACLGKACGRK, C20-Oxidation (15.99492 Da)

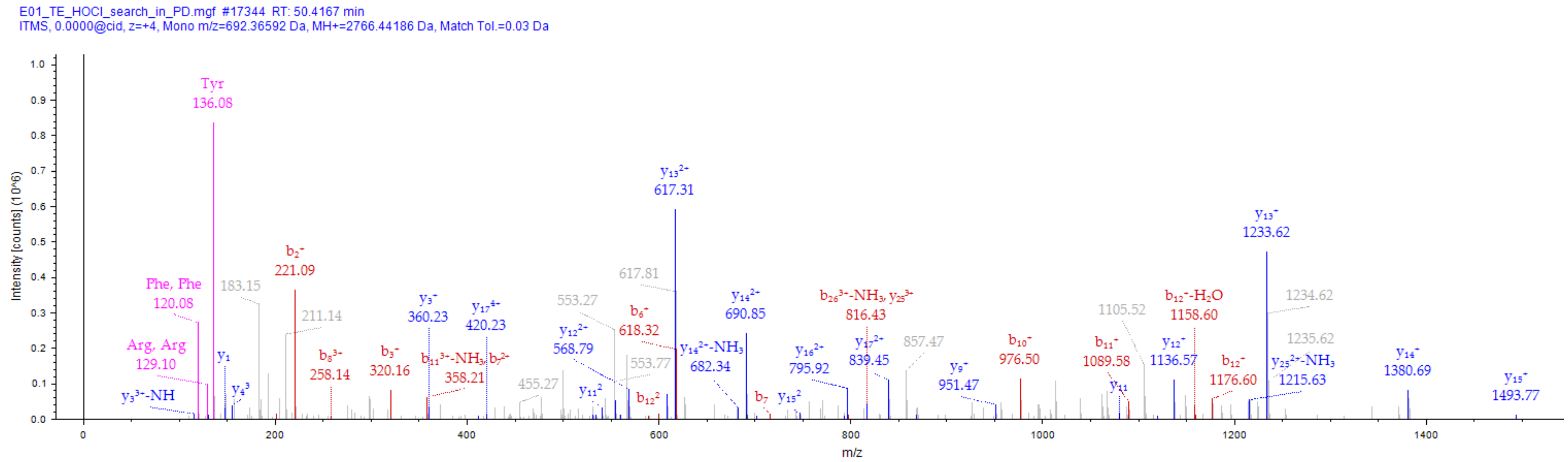

#1	Immonium	b^+	b^{2+}	b^{3+}	b^{4+}	Seq.	y^+	y^{2+}	y^{3+}	y^{4+}	#2
1	136.07569	164.07061	82.53894	55.36172	41.77311	Y					28
2	30.03383	221.09207	111.04967	74.36887	56.02847	G	2603.36973	1302.18850	868.46143	651.59789	27
3	72.08078	320.16048	160.58388	107.39168	80.79558	V	2546.34826	1273.67777	849.45427	637.34252	26
4	44.04948	391.19760	196.10244	131.07072	98.55486	A	2447.27985	1224.14356	816.43147	612.57542	25
5	44.04948	462.23471	231.62099	154.74975	116.31414	A	2376.24274	1188.62501	792.75243	594.81614	24
6	129.11347	618.33582	309.67155	206.78346	155.33941	R	2305.20562	1153.10645	769.07339	577.05686	23
7	70.06513	715.38858	358.19793	239.13438	179.60260	P	2149.10451	1075.05589	717.03969	538.03159	22
8	30.03383	772.41005	386.70866	258.14153	193.85797	G	2052.05175	1026.52951	684.68877	513.76839	21
9	120.08078	919.47846	460.24287	307.16434	230.62507	F	1995.03028	998.01878	665.68161	499.51303	20
10	30.03383	976.49993	488.75360	326.17149	244.88044	G	1847.96187	924.48457	616.65881	462.74592	19
11	86.09643	1089.58399	545.29563	363.86618	273.15146	L	1790.94041	895.97384	597.65165	448.49056	18
12	60.04439	1176.61602	588.81165	392.87686	294.90946	S	1677.85634	839.43181	559.95697	420.21954	17
13	70.06513	1273.66878	637.33803	425.22778	319.17265	P	1590.82431	795.91580	530.94629	398.46154	16
14	86.09643	1386.75285	693.88006	462.92247	347.44367	I	1493.77155	747.38941	498.59537	374.19834	15
15	120.08078	1533.82126	767.41427	511.94527	384.21077	F	1380.68749	690.84738	460.90068	345.92733	14
16	70.06513	1630.87402	815.94065	544.29619	408.47396	P	1233.61907	617.31317	411.87788	309.16023	13
17	30.03383	1687.89549	844.45138	563.30335	422.72933	G	1136.56631	568.78679	379.52695	284.89703	12
18	30.03383	1744.91695	872.96211	582.31050	436.98470	G	1079.54484	540.27606	360.51980	270.64167	11
19	44.04948	1815.95407	908.48067	605.98954	454.74397	A	1022.52338	511.76533	341.51264	256.38630	10
20	92.01646	1934.95816	967.98272	645.65757	484.49500	C-Oxidation	951.48627	476.24677	317.83361	238.62702	9
21	86.09643	2048.04223	1024.52475	683.35226	512.76601	L	832.48217	416.74472	278.16557	208.87600	8
22	30.03383	2105.06369	1053.03548	702.35942	527.02138	G	719.39810	360.20269	240.47089	180.60498	7
23	101.10732	2233.15866	1117.08297	745.05774	559.04512	K	662.37664	331.69196	221.46373	166.34962	6
24	44.04948	2304.19577	1152.60152	768.73677	576.80440	A	534.28168	267.64448	178.76541	134.32588	5
25	76.02155	2407.20495	1204.10611	803.07317	602.55670	C	463.24456	232.12592	155.08637	116.56660	4
26	30.03383	2464.22642	1232.61685	822.08032	616.81206	G	360.23538	180.62133	120.74998	90.81430	3
27	129.11347	2620.32753	1310.66740	874.11403	655.83734	R	303.21392	152.11060	101.74282	76.55894	2
28	101.10732					K	147.11280	74.06004	49.70912	37.53366	1

YGVAARPGFGLSPIFPGGACLGKACGRK, C25-Dioxidation (31.98983Da)

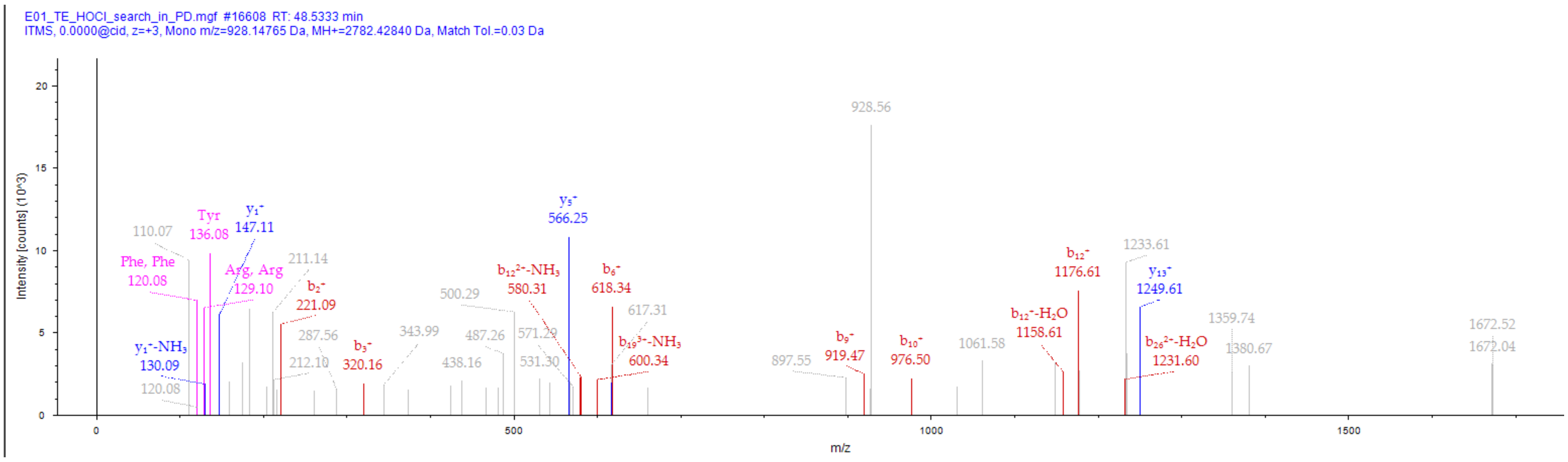

#1	Immonium	b+	b2+	b3+	Seq.	y+	y2+	y3+	#2
1	136.07569	164.07061	82.53894	55.36172	Y				28
2	30.03383	221.09207	111.04967	74.36887	G	2619.36464	1310.18596	873.79306	27
3	72.08078	320.16048	160.58388	107.39168	V	2562.34318	1281.67523	854.78591	26
4	44.04948	391.19760	196.10244	131.07072	A	2463.27476	1232.14102	821.76311	25
5	44.04948	462.23471	231.62099	154.74975	A	2392.23765	1196.62246	798.08407	24
6	129.11347	618.33582	309.67155	206.78346	R	2321.20054	1161.10391	774.40503	23
7	70.06513	715.38858	358.19793	239.13438	P	2165.09943	1083.05335	722.37133	22
8	30.03383	772.41005	386.70866	258.14153	G	2068.04666	1034.52697	690.02040	21
9	120.08078	919.47846	460.24287	307.16434	F	2011.02520	1006.01624	671.01325	20
10	30.03383	976.49993	488.75360	326.17149	G	1863.95678	932.48203	621.99045	19
11	86.09643	1089.58399	545.29563	363.86618	L	1806.93532	903.97130	602.98329	18
12	60.04439	1176.61602	588.81165	392.87686	S	1693.85126	847.42927	565.28860	17
13	70.06513	1273.66878	637.33803	425.22778	P	1606.81923	803.91325	536.27793	16
14	86.09643	1386.75285	693.88006	462.92247	I	1509.76646	755.38687	503.92701	15
15	120.08078	1533.82126	767.41427	511.94527	F	1396.68240	698.84484	466.23232	14
16	70.06513	1630.87402	815.94065	544.29619	P	1249.61399	625.31063	417.20951	13
17	30.03383	1687.89549	844.45138	563.30335	G	1152.56122	576.78425	384.85859	12
18	30.03383	1744.91695	872.96211	582.31050	G	1095.53976	548.27352	365.85144	11
19	44.04948	1815.95407	908.48067	605.98954	A	1038.51829	519.76279	346.84428	10
20	76.02155	1918.96325	959.98526	640.32593	C	967.48118	484.24423	323.16524	9
21	86.09643	2032.04731	1016.52730	678.02062	L	864.47200	432.73964	288.82885	8
22	30.03383	2089.06878	1045.03803	697.02778	G	751.38793	376.19760	251.13416	7
23	101.10732	2217.16374	1109.08551	739.72610	K	694.36647	347.68687	232.12701	6
24	44.04948	2288.20085	1144.60407	763.40514	A	566.27151	283.63939	189.42869	5
25	108.01138	2423.19987	1212.10357	808.40481	C-Dioxidati...	495.23439	248.12083	165.74965	4
26	30.03383	2480.22133	1240.61430	827.41196	G	360.23538	180.62133	120.74998	3
27	129.11347	2636.32244	1318.66486	879.44567	R	303.21392	152.11060	101.74282	2
28	101.10732				K	147.11280	74.06004	49.70912	1

YGVAARPGFGLSPIFPGGACLGKACGR, C20-Dioxidation (31.98983 Da), C25-Dioxidation (31.98983 Da)

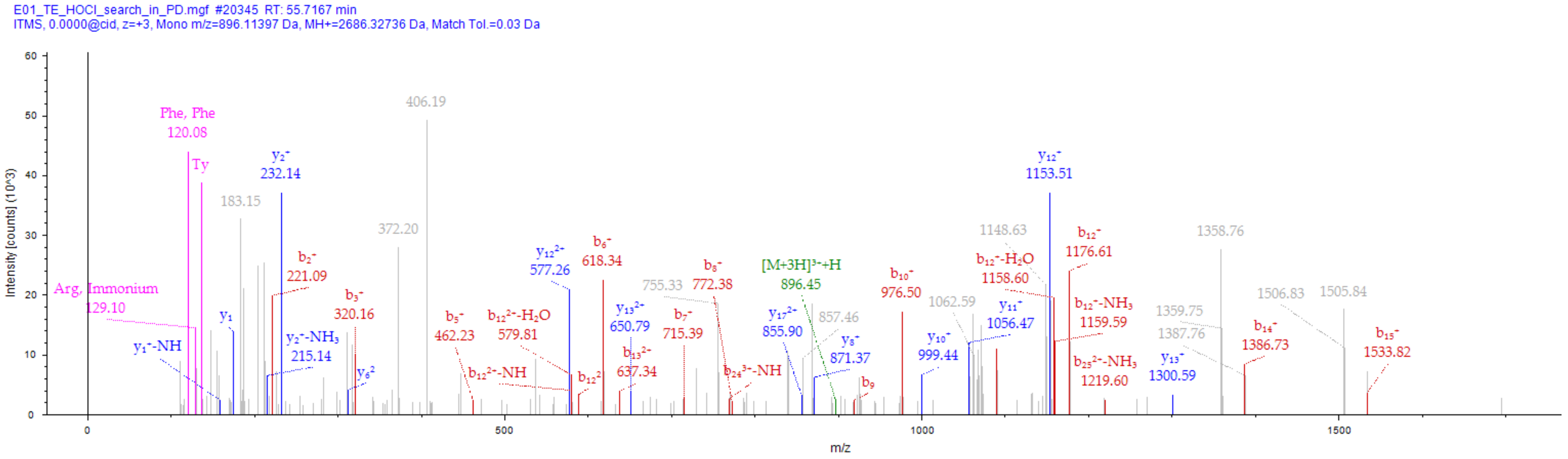

#1	Immonium	b⁺	b²⁺	b³⁺	Seq.	y⁺	y²⁺	y³⁺	#2
1	136.07569	164.07061	82.53894	55.36172	Y				27
2	30.03383	221.09207	111.04967	74.36887	G	2523.25951	1262.13339	841.75802	26
3	72.08078	320.16048	160.58388	107.39168	V	2466.23804	1233.62266	822.75087	25
4	44.04948	391.19760	196.10244	131.07072	A	2367.16963	1184.08845	789.72806	24
5	44.04948	462.23471	231.62099	154.74975	A	2296.13252	1148.56990	766.04902	23
6	129.11347	618.33582	309.67155	206.78346	R	2225.09540	1113.05134	742.36999	22
7	70.06513	715.38858	358.19793	239.13438	P	2068.99429	1035.00078	690.33628	21
8	30.03383	772.41005	386.70866	258.14153	G	1971.94153	986.47440	657.98536	20
9	120.08078	919.47846	460.24287	307.16434	F	1914.92006	957.96367	638.97821	19
10	30.03383	976.49993	488.75360	326.17149	G	1767.85165	884.42946	589.95540	18
11	86.09643	1089.58399	545.29563	363.86618	L	1710.83019	855.91873	570.94825	17
12	60.04439	1176.61602	588.81165	392.87686	S	1597.74612	799.37670	533.25356	16
13	70.06513	1273.66878	637.33803	425.22778	P	1510.71409	755.86069	504.24288	15
14	86.09643	1386.75285	693.88006	462.92247	I	1413.66133	707.33430	471.89196	14
15	120.08078	1533.82126	767.41427	511.94527	F	1300.57727	650.79227	434.19727	13
16	70.06513	1630.87402	815.94065	544.29619	P	1153.50885	577.25806	385.17447	12
17	30.03383	1687.89549	844.45138	563.30335	G	1056.45609	528.73168	352.82355	11
18	30.03383	1744.91695	872.96211	582.31050	G	999.43462	500.22095	333.81639	10
19	44.04948	1815.95407	908.48067	605.98954	A	942.41316	471.71022	314.80924	9
20	108.01138	1950.95308	975.98018	650.98921	C-Dioxidati...	871.37605	436.19166	291.13020	8
21	86.09643	2064.03714	1032.52221	688.68390	L	736.37703	368.69216	246.13053	7
22	30.03383	2121.05861	1061.03294	707.69105	G	623.29297	312.15012	208.43584	6
23	101.10732	2249.15357	1125.08042	750.38937	K	566.27151	283.63939	189.42869	5
24	44.04948	2320.19068	1160.59898	774.06841	A	438.17654	219.59191	146.73037	4
25	108.01138	2455.18970	1228.09849	819.06808	C-Dioxidati...	367.13943	184.07335	123.05133	3
26	30.03383	2512.21116	1256.60922	838.07524	G	232.14042	116.57385	78.05166	2
27	129.11347				R	175.11895	88.06311	59.04450	1

YGVAARPGFGLSPIFPGGACLGKACGR, C25-Dioxidation (31.98983 Da)

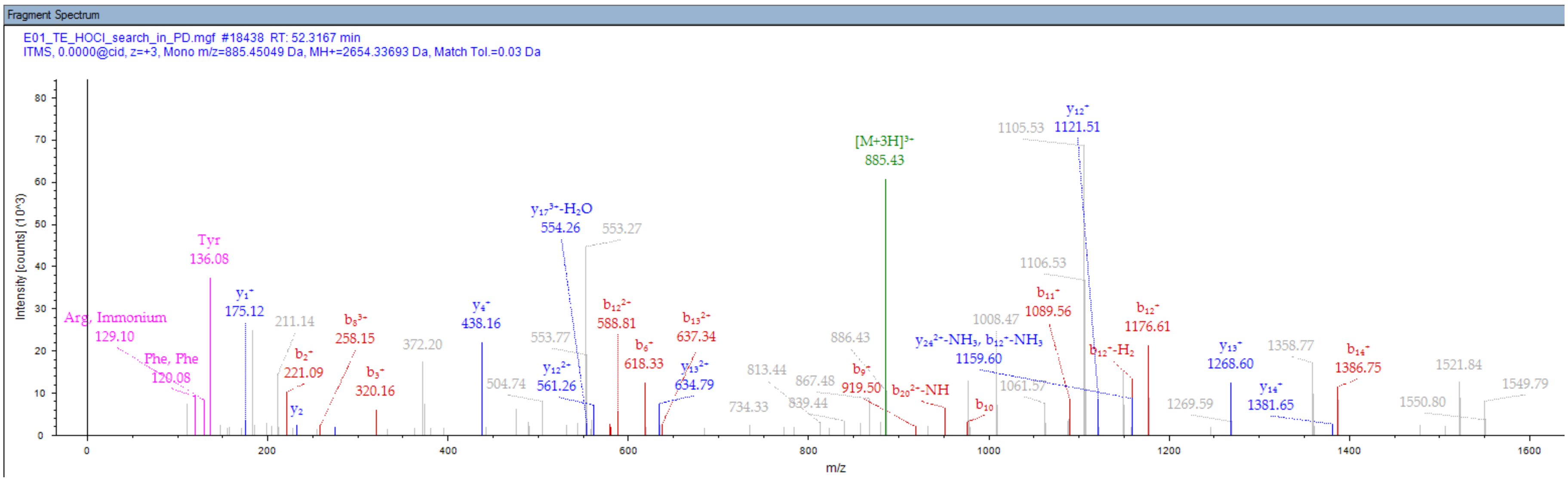

#1	Immonium	b⁺	b²⁺	b³⁺	Seq.	y⁺	y²⁺	y³⁺	#2
1	136.07569	164.07061	82.53894	55.36172	Y				27
2	30.03383	221.09207	111.04967	74.36887	G	2491.26968	1246.13848	831.09474	26
3	72.08078	320.16048	160.58388	107.39168	V	2434.24821	1217.62775	812.08759	25
4	44.04948	391.19760	196.10244	131.07072	A	2335.17980	1168.09354	779.06478	24
5	44.04948	462.23471	231.62099	154.74975	A	2264.14269	1132.57498	755.38575	23
6	129.11347	618.33582	309.67155	206.78346	R	2193.10557	1097.05642	731.70671	22
7	70.06513	715.38858	358.19793	239.13438	P	2037.00446	1019.00587	679.67301	21
8	30.03383	772.41005	386.70866	258.14153	G	1939.95170	970.47949	647.32208	20
9	120.08078	919.47846	460.24287	307.16434	F	1882.93023	941.96876	628.31493	19
10	30.03383	976.49993	488.75360	326.17149	G	1735.86182	868.43455	579.29212	18
11	86.09643	1089.58399	545.29563	363.86618	L	1678.84036	839.92382	560.28497	17
12	60.04439	1176.61602	588.81165	392.87686	S	1565.75629	783.38178	522.59028	16
13	70.06513	1273.66878	637.33803	425.22778	P	1478.72426	739.86577	493.57961	15
14	86.09643	1386.75285	693.88006	462.92247	I	1381.67150	691.33939	461.22868	14
15	120.08078	1533.82126	767.41427	511.94527	F	1268.58744	634.79736	423.53400	13
16	70.06513	1630.87402	815.94065	544.29619	P	1121.51902	561.26315	374.51119	12
17	30.03383	1687.89549	844.45138	563.30335	G	1024.46626	512.73677	342.16027	11
18	30.03383	1744.91695	872.96211	582.31050	G	967.44480	484.22604	323.15312	10
19	44.04948	1815.95407	908.48067	605.98954	A	910.42333	455.71530	304.14596	9
20	76.02155	1918.96325	959.98526	640.32593	C	839.38622	420.19675	280.46692	8
21	86.09643	2032.04731	1016.52730	678.02062	L	736.37703	368.69216	246.13053	7
22	30.03383	2089.06878	1045.03803	697.02778	G	623.29297	312.15012	208.43584	6
23	101.10732	2217.16374	1109.08551	739.72610	K	566.27151	283.63939	189.42869	5
24	44.04948	2288.20085	1144.60407	763.40514	A	438.17654	219.59191	146.73037	4
25	108.01138	2423.19987	1212.10357	808.40481	C-Dioxidati...	367.13943	184.07335	123.05133	3
26	30.03383	2480.22133	1240.61430	827.41196	G	232.14042	116.57385	78.05166	2
27	129.11347				R	175.11895	88.06311	59.04450	1

YGVAARPGFGLSPIFPGGACLGK, C20-Trioxidation (47.98474 Da)

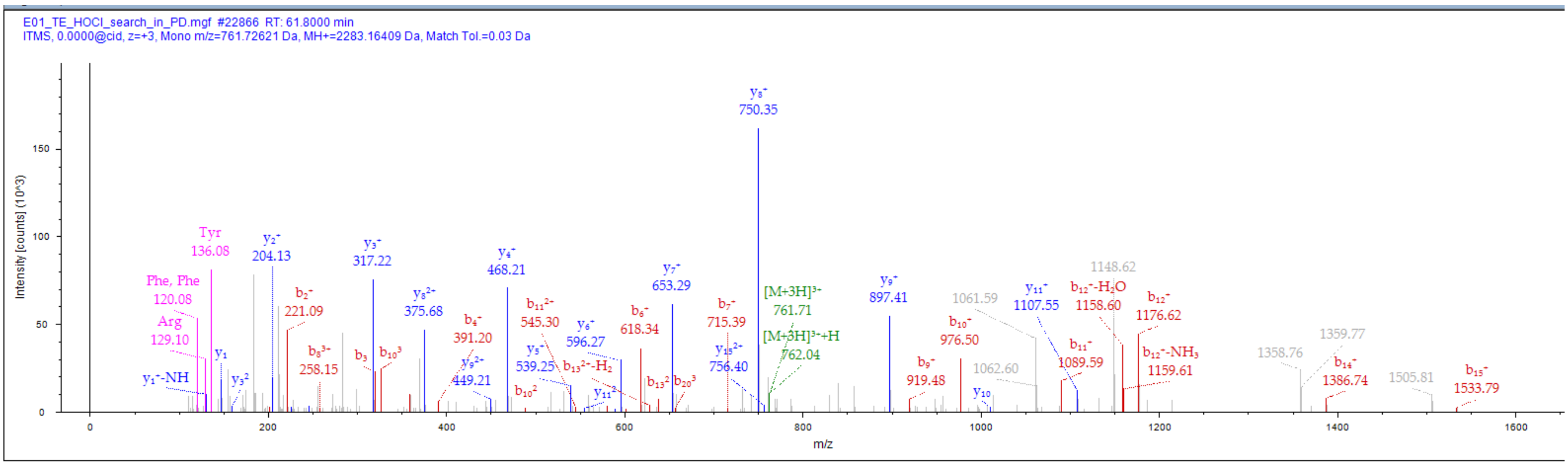

#1	Immonium	b⁺	b²⁺	b³⁺	Seq.	y⁺	y²⁺	y³⁺	#2
1	136.07569	164.07061	82.53894	55.36172	Y				23
2	30.03383	221.09207	111.04967	74.36887	G	2120.09572	1060.55150	707.37009	22
3	72.08078	320.16048	160.58388	107.39168	V	2063.07426	1032.04077	688.36294	21
4	44.04948	391.19760	196.10244	131.07072	A	1964.00584	982.50656	655.34013	20
5	44.04948	462.23471	231.62099	154.74975	A	1892.96873	946.98800	631.66109	19
6	129.11347	618.33582	309.67155	206.78346	R	1821.93162	911.46945	607.98206	18
7	70.06513	715.38858	358.19793	239.13438	P	1665.83050	833.41889	555.94835	17
8	30.03383	772.41005	386.70866	258.14153	G	1568.77774	784.89251	523.59743	16
9	120.08078	919.47846	460.24287	307.16434	F	1511.75628	756.38178	504.59028	15
10	30.03383	976.49993	488.75360	326.17149	G	1364.68786	682.84757	455.56747	14
11	86.09643	1089.58399	545.29563	363.86618	L	1307.66640	654.33684	436.56032	13
12	60.04439	1176.61602	588.81165	392.87686	S	1194.58234	597.79481	398.86563	12
13	70.06513	1273.66878	637.33803	425.22778	P	1107.55031	554.27879	369.85495	11
14	86.09643	1386.75285	693.88006	462.92247	I	1010.49754	505.75241	337.50403	10
15	120.08078	1533.82126	767.41427	511.94527	F	897.41348	449.21038	299.80934	9
16	70.06513	1630.87402	815.94065	544.29619	P	750.34507	375.67617	250.78654	8
17	30.03383	1687.89549	844.45138	563.30335	G	653.29230	327.14979	218.43562	7
18	30.03383	1744.91695	872.96211	582.31050	G	596.27084	298.63906	199.42846	6
19	44.04948	1815.95407	908.48067	605.98954	A	539.24937	270.12833	180.42131	5
20	124.00629	1966.94799	983.97764	656.32085	C-Trioxidat..	468.21226	234.60977	156.74227	4
21	86.09643	2080.03206	1040.51967	694.01554	L	317.21833	159.11280	106.41096	3
22	30.03383	2137.05352	1069.03040	713.02269	G	204.13427	102.57077	68.71627	2
23	101.10732				K	147.11280	74.06004	49.70912	1

YGVAARPGFGLSPIFPGGACLGK, C20-Dioxidation (31.98983 Da)

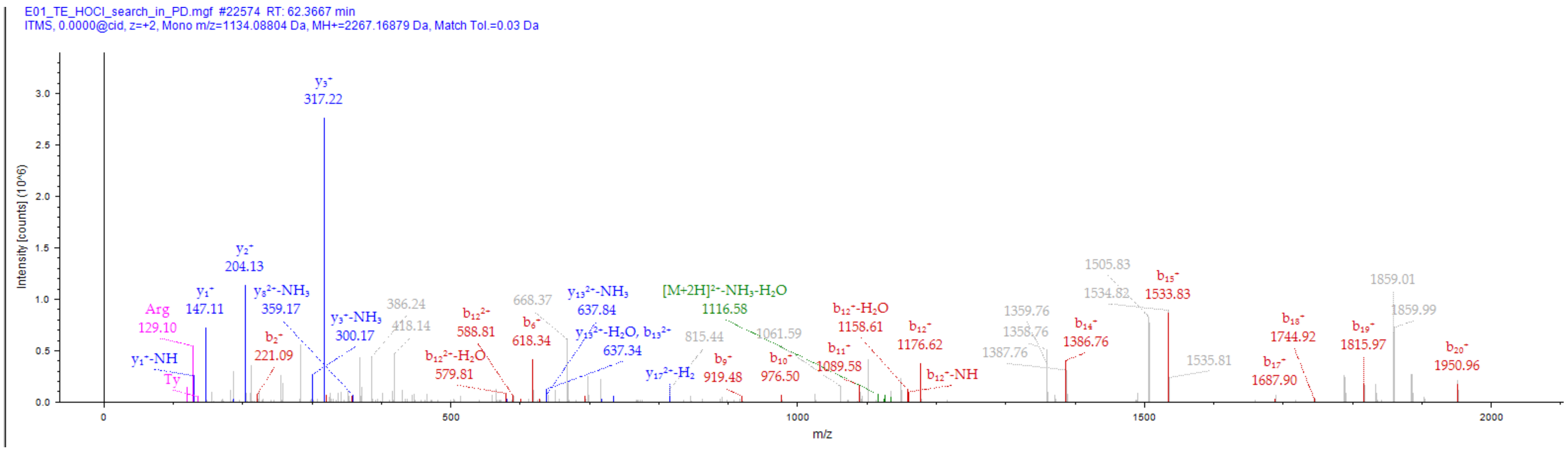

#1	Immonium	b⁺	b²⁺	Seq.	y⁺	y²⁺	#2
1	136.07569	164.07061	82.53894	Y			23
2	30.03383	221.09207	111.04967	G	2104.10081	1052.55404	22
3	72.08078	320.16048	160.58388	V	2047.07934	1024.04331	21
4	44.04948	391.19760	196.10244	A	1948.01093	974.50910	20
5	44.04948	462.23471	231.62099	A	1876.97381	938.99055	19
6	129.11347	618.33582	309.67155	R	1805.93670	903.47199	18
7	70.06513	715.38858	358.19793	P	1649.83559	825.42143	17
8	30.03383	772.41005	386.70866	G	1552.78283	776.89505	16
9	120.08078	919.47846	460.24287	F	1495.76136	748.38432	15
10	30.03383	976.49993	488.75360	G	1348.69295	674.85011	14
11	86.09643	1089.58399	545.29563	L	1291.67148	646.33938	13
12	60.04439	1176.61602	588.81165	S	1178.58742	589.79735	12
13	70.06513	1273.66878	637.33803	P	1091.55539	546.28133	11
14	86.09643	1386.75285	693.88006	I	994.50263	497.75495	10
15	120.08078	1533.82126	767.41427	F	881.41856	441.21292	9
16	70.06513	1630.87402	815.94065	P	734.35015	367.67871	8
17	30.03383	1687.89549	844.45138	G	637.29739	319.15233	7
18	30.03383	1744.91695	872.96211	G	580.27592	290.64160	6
19	44.04948	1815.95407	908.48067	A	523.25446	262.13087	5
20	108.01138	1950.95308	975.98018	C-Dioxidati...	452.21735	226.61231	4
21	86.09643	2064.03714	1032.52221	L	317.21833	159.11280	3
22	30.03383	2121.05861	1061.03294	G	204.13427	102.57077	2
23	101.10732			K	147.11280	74.06004	1

YGVAARPGFGLSPIFPGGACLGKACGR, C25-Oxidation (15.99492 Da), C20-Trioxidation (47.98474 Da)

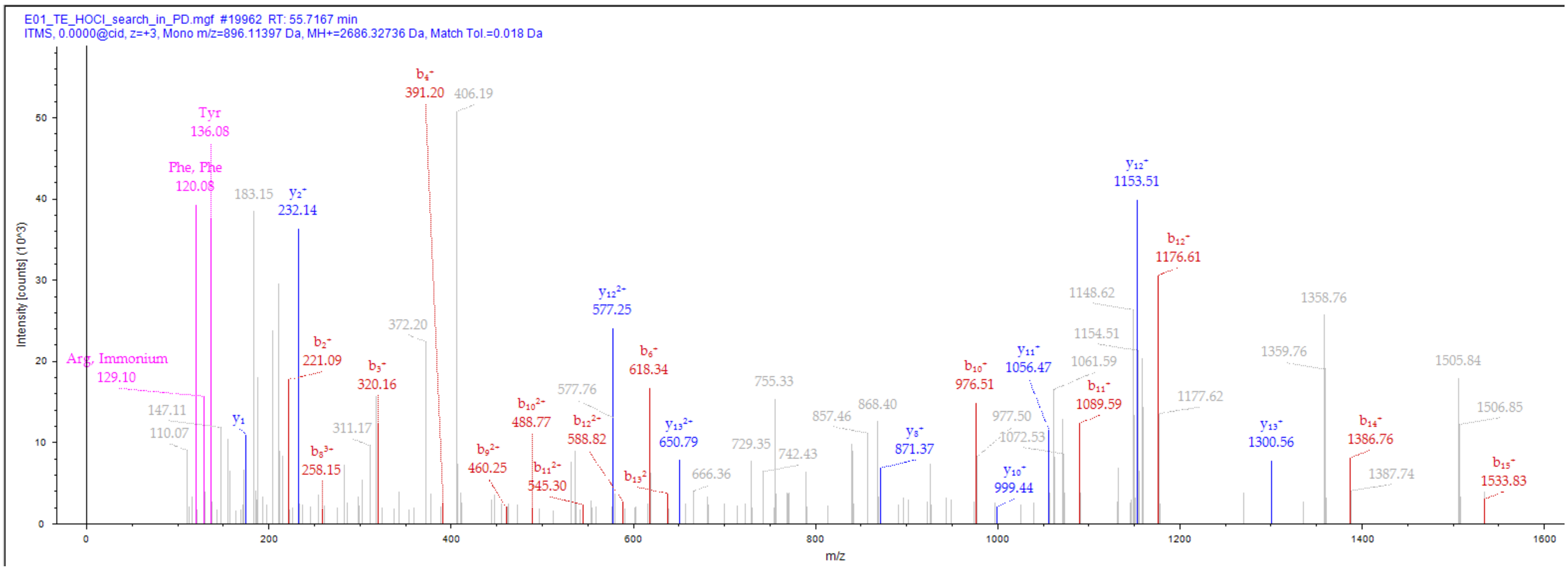

#1	Immonium	b⁺	b²⁺	b³⁺	Seq.	y⁺	y²⁺	y³⁺	#2
1	136.07569	164.07061	82.53894	55.36172	Y				27
2	30.03383	221.09207	111.04967	74.36887	G	2523.25951	1262.13339	841.75802	26
3	72.08078	320.16048	160.58388	107.39168	V	2466.23804	1233.62266	822.75087	25
4	44.04948	391.19760	196.10244	131.07072	A	2367.16963	1184.08845	789.72806	24
5	44.04948	462.23471	231.62099	154.74975	A	2296.13252	1148.56990	766.04902	23
6	129.11347	618.33582	309.67155	206.78346	R	2225.09540	1113.05134	742.36999	22
7	70.06513	715.38858	358.19793	239.13438	P	2068.99429	1035.00078	690.33628	21
8	30.03383	772.41005	386.70866	258.14153	G	1971.94153	986.47440	657.98536	20
9	120.08078	919.47846	460.24287	307.16434	F	1914.92006	957.96367	638.97821	19
10	30.03383	976.49993	488.75360	326.17149	G	1767.85165	884.42946	589.95540	18
11	86.09643	1089.58399	545.29563	363.86618	L	1710.83019	855.91873	570.94825	17
12	60.04439	1176.61602	588.81165	392.87686	S	1597.74612	799.37670	533.25356	16
13	70.06513	1273.66878	637.33803	425.22778	P	1510.71409	755.86069	504.24288	15
14	86.09643	1386.75285	693.88006	462.92247	I	1413.66133	707.33430	471.89196	14
15	120.08078	1533.82126	767.41427	511.94527	F	1300.57727	650.79227	434.19727	13
16	70.06513	1630.87402	815.94065	544.29619	P	1153.50885	577.25806	385.17447	12
17	30.03383	1687.89549	844.45138	563.30335	G	1056.45609	528.73168	352.82355	11
18	30.03383	1744.91695	872.96211	582.31050	G	999.43463	500.22095	333.81639	10
19	44.04948	1815.95407	908.48067	605.98954	A	942.41316	471.71022	314.80924	9
20	124.00629	1966.94799	983.97764	656.32085	C-Trioxidat..	871.37605	436.19166	291.13020	8
21	86.09643	2080.03206	1040.51967	694.01554	L	720.38212	360.69470	240.79889	7
22	30.03383	2137.05352	1069.03040	713.02269	G	607.29806	304.15267	203.10420	6
23	101.10732	2265.14848	1133.07788	755.72101	K	550.27659	275.64193	184.09705	5
24	44.04948	2336.18560	1168.59644	779.40005	A	422.18163	211.59445	141.39873	4
25	92.01646	2455.18970	1228.09849	819.06808	C-Oxidation	351.14452	176.07590	117.71969	3
26	30.03383	2512.21116	1256.60922	838.07524	G	232.14042	116.57385	78.05166	2
27	129.11347				R	175.11895	88.06311	59.04450	1

Tropoelastin Isoform 6

Treated with MPO

VPGVGLPGVYPGGVLPGAR, Y10-Chlorination (33.96103 Da)

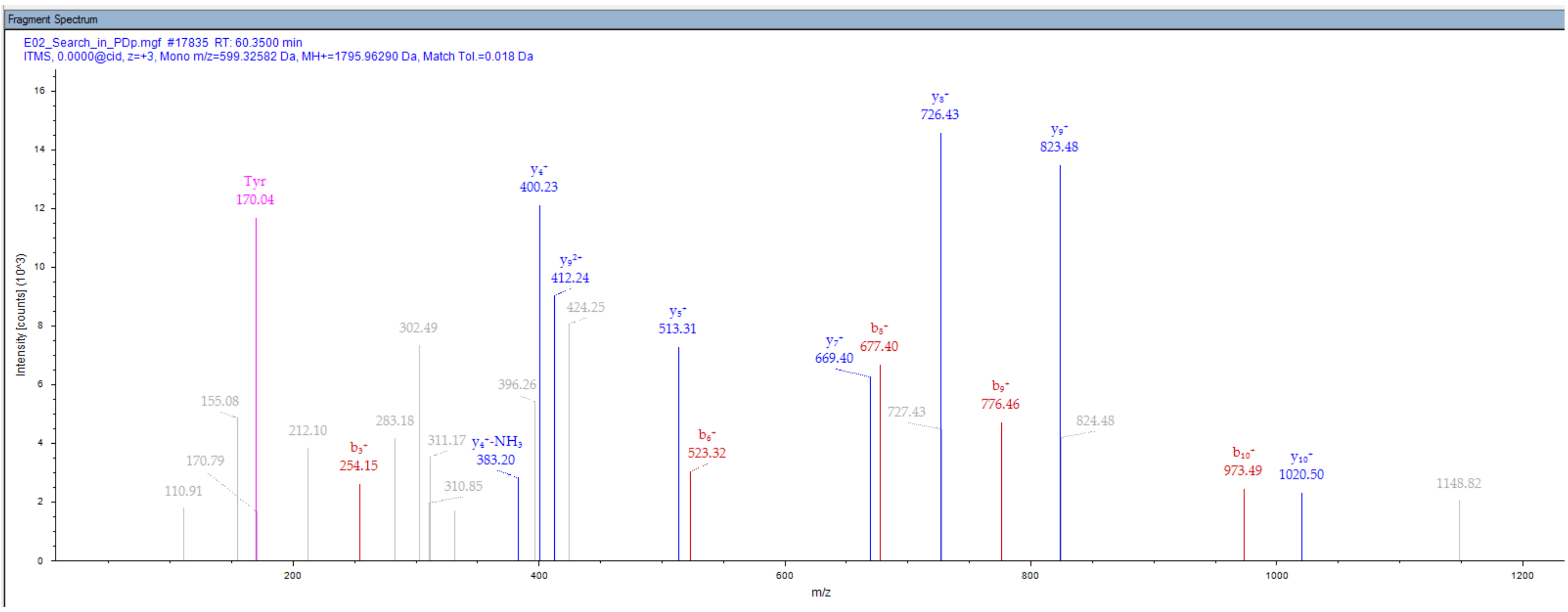

Ion Series	Neutral Losses	Precursor Ions

#1	Immonium	b+	b2+	b3+	Seq.	y+	y2+	y3+	#2
1	72.08078	100.07569	50.54148	34.03008	V				19
2	70.06513	197.12845	99.06787	66.38100	P	1696.89363	848.95045	566.30273	18
3	30.03383	254.14992	127.57860	85.38816	G	1599.84087	800.42407	533.95181	17
4	72.08078	353.21833	177.11280	118.41096	V	1542.81940	771.91334	514.94465	16
5	30.03383	410.23980	205.62354	137.41812	G	1443.75099	722.37913	481.92185	15
6	86.09643	523.32386	262.16557	175.11280	L	1386.72952	693.86840	462.91469	14
7	70.06513	620.37662	310.69195	207.46373	P	1273.64546	637.32637	425.22000	13
8	30.03383	677.39809	339.20268	226.47088	G	1176.59270	588.79999	392.86908	12
9	72.08078	776.46650	388.73689	259.49368	V	1119.57123	560.28925	373.86193	11
10	170.03672	973.49086	487.24907	325.16847	Y-Chlorinat...	1020.50282	510.75505	340.83912	10
11	70.06513	1070.54362	535.77545	357.51939	P	823.47846	412.24287	275.16434	9
12	30.03383	1127.56508	564.28618	376.52655	G	726.42570	363.71649	242.81342	8
13	30.03383	1184.58655	592.79691	395.53370	G	669.40423	335.20576	223.80626	7
14	72.08078	1283.65496	642.33112	428.55651	V	612.38277	306.69502	204.79911	6
15	86.09643	1396.73903	698.87315	466.25119	L	513.31436	257.16082	171.77630	5
16	70.06513	1493.79179	747.39953	498.60211	P	400.23029	200.61879	134.08162	4
17	30.03383	1550.81325	775.91027	517.60927	G	303.17753	152.09240	101.73069	3
18	44.04948	1621.85037	811.42882	541.28831	A	246.15607	123.58167	82.72354	2
19	129.11347				R	175.11895	88.06311	59.04450	1

VPGVGLPGVYPGGVLPGAR

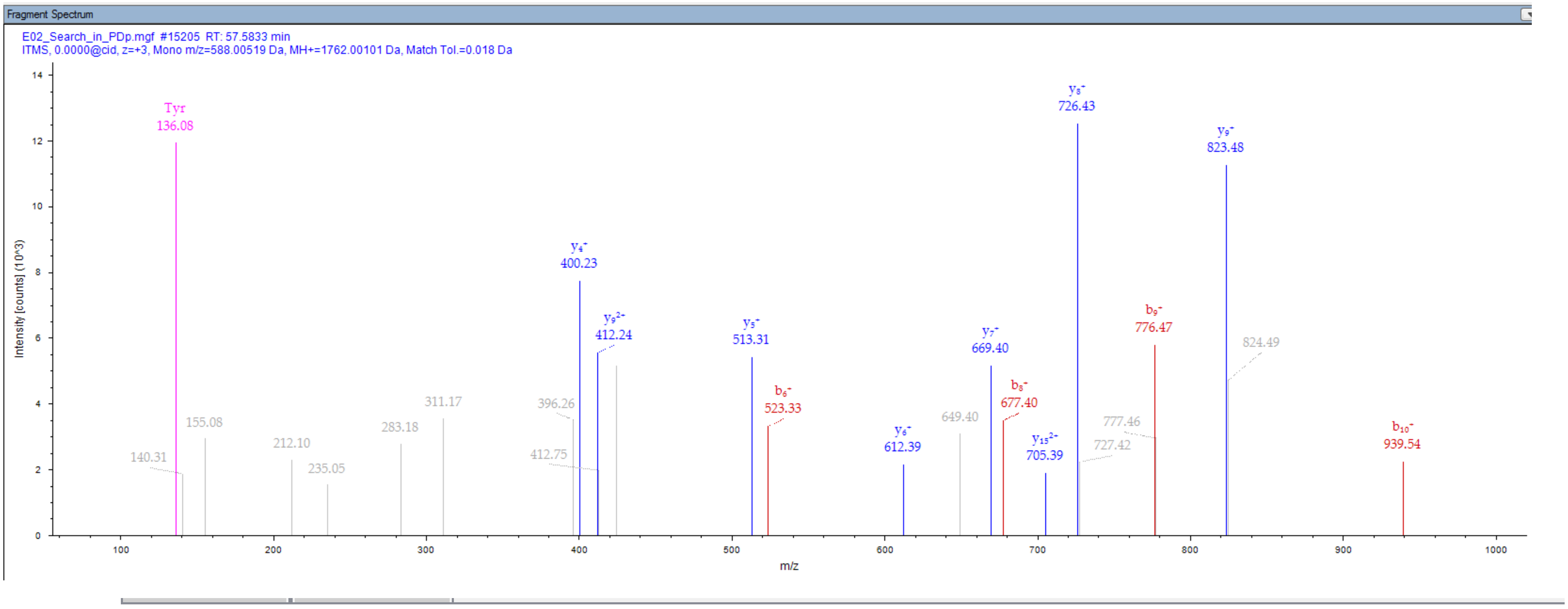

#1	Immonium	b⁺	b²⁺	b³⁺	Seq.	y⁺	y²⁺	y³⁺	#2
1	72.08078	100.07569	50.54148	34.03008	V				19
2	70.06513	197.12845	99.06787	66.38100	P	1662.93260	831.96994	554.98238	18
3	30.03383	254.14992	127.57860	85.38816	G	1565.87984	783.44356	522.63146	17
4	72.08078	353.21833	177.11280	118.41096	V	1508.85837	754.93283	503.62431	16
5	30.03383	410.23980	205.62354	137.41812	G	1409.78996	705.39862	470.60150	15
6	86.09643	523.32386	262.16557	175.11280	L	1352.76850	676.88789	451.59435	14
7	70.06513	620.37662	310.69195	207.46373	P	1239.68443	620.34585	413.89966	13
8	30.03383	677.39809	339.20268	226.47088	G	1142.63167	571.81947	381.54874	12
9	72.08078	776.46650	388.73689	259.49368	V	1085.61020	543.30874	362.54159	11
10	136.07569	939.52983	470.26855	313.84813	Y	986.54179	493.77453	329.51878	10
11	70.06513	1036.58259	518.79493	346.19905	P	823.47846	412.24287	275.16434	9
12	30.03383	1093.60406	547.30567	365.20620	G	726.42570	363.71649	242.81342	8
13	30.03383	1150.62552	575.81640	384.21336	G	669.40423	335.20576	223.80626	7
14	72.08078	1249.69393	625.35061	417.23616	V	612.38277	306.69502	204.79911	6
15	86.09643	1362.77800	681.89264	454.93085	L	513.31436	257.16082	171.77630	5
16	70.06513	1459.83076	730.41902	487.28177	P	400.23029	200.61879	134.08162	4
17	30.03383	1516.85223	758.92975	506.28893	G	303.17753	152.09240	101.73069	3
18	44.04948	1587.88934	794.44831	529.96796	A	246.15607	123.58167	82.72354	2
19	129.11347				R	175.11895	88.06311	59.04450	1

APGVGGAFAGIPGVGPFGGPQPGVPLGYPIK, Y28-Chlorination (33.96103 Da)

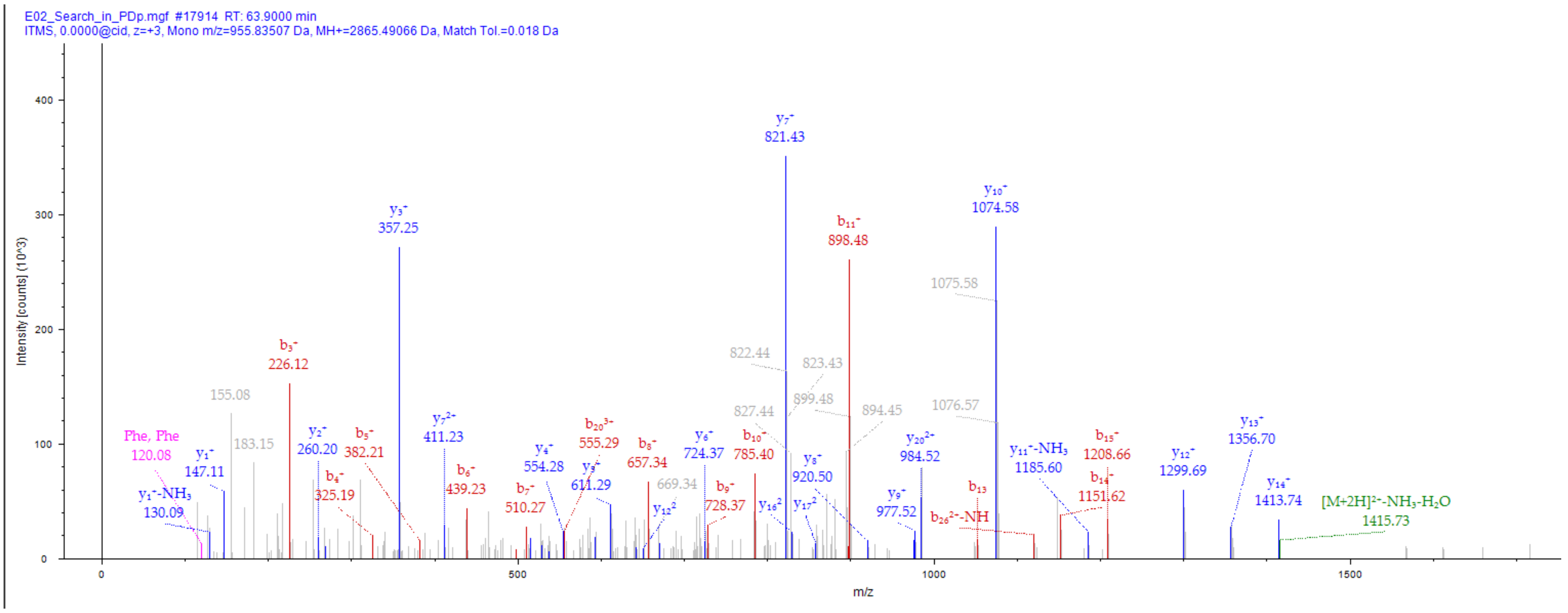

#1	Immonium	b⁺	b²⁺	b³⁺	Seq.	y⁺	y²⁺	y³⁺	#2
1	44.04948	72.04439	36.52583	24.68631	A				31
2	70.06513	169.09715	85.05222	57.03724	P	2794.44821	1397.72774	932.15425	30
3	30.03383	226.11862	113.56295	76.04439	G	2697.39545	1349.20136	899.80333	29
4	72.08078	325.18703	163.09715	109.06720	V	2640.37398	1320.69063	880.79618	28
5	30.03383	382.20850	191.60789	128.07435	G	2541.30557	1271.15642	847.77337	27
6	30.03383	439.22996	220.11862	147.08150	G	2484.28411	1242.64569	828.76622	26
7	44.04948	510.26707	255.63717	170.76054	A	2427.26264	1214.13496	809.75906	25
8	120.08078	657.33549	329.17138	219.78335	F	2356.22553	1178.61640	786.08003	24
9	44.04948	728.37260	364.68994	243.46238	A	2209.15711	1105.08220	737.05722	23
10	30.03383	785.39406	393.20067	262.46954	G	2138.12000	1069.56364	713.37818	22
11	86.09643	898.47813	449.74270	300.16423	I	2081.09854	1041.05291	694.37103	21
12	70.06513	995.53089	498.26908	332.51515	P	1968.01447	984.51087	656.67634	20
13	30.03383	1052.55236	526.77982	351.52230	G	1870.96171	935.98449	624.32542	19
14	72.08078	1151.62077	576.31402	384.54511	V	1813.94025	907.47376	605.31827	18
15	30.03383	1208.64223	604.82475	403.55226	G	1714.87183	857.93955	572.29546	17
16	70.06513	1305.69500	653.35114	435.90318	P	1657.85037	829.42882	553.28831	16
17	120.08078	1452.76341	726.88534	484.92599	F	1560.79760	780.90244	520.93739	15
18	30.03383	1509.78487	755.39608	503.93314	G	1413.72919	707.36823	471.91458	14
19	30.03383	1566.80634	783.90681	522.94030	G	1356.70773	678.85750	452.90743	13
20	70.06513	1663.85910	832.43319	555.29122	P	1299.68626	650.34677	433.90027	12
21	101.07094	1791.91768	896.46248	597.97741	Q	1202.63350	601.82039	401.54935	11
22	70.06513	1888.97044	944.98886	630.32833	P	1074.57492	537.79110	358.86316	10
23	30.03383	1945.99191	973.49959	649.33549	G	977.52216	489.26472	326.51224	9
24	72.08078	2045.06032	1023.03380	682.35829	V	920.50069	460.75399	307.50508	8
25	70.06513	2142.11308	1071.56018	714.70921	P	821.43228	411.21978	274.48228	7
26	86.09643	2255.19715	1128.10221	752.40390	L	724.37952	362.69340	242.13136	6
27	30.03383	2312.21861	1156.61294	771.41106	G	611.29545	306.15136	204.43667	5
28	170.03672	2509.24297	1255.12512	837.08584	Y-Chlorinat...	554.27399	277.64063	185.42951	4
29	70.06513	2606.29573	1303.65150	869.43676	P	357.24963	179.12845	119.75473	3
30	86.09643	2719.37980	1360.19354	907.13145	I	260.19687	130.60207	87.40381	2
31	101.10732				K	147.11280	74.06004	49.70912	1

APGVGGAFAGIPGVGPFGGPQPGVPLGYPIK, Y28-diChlorination (67.92206 Da)

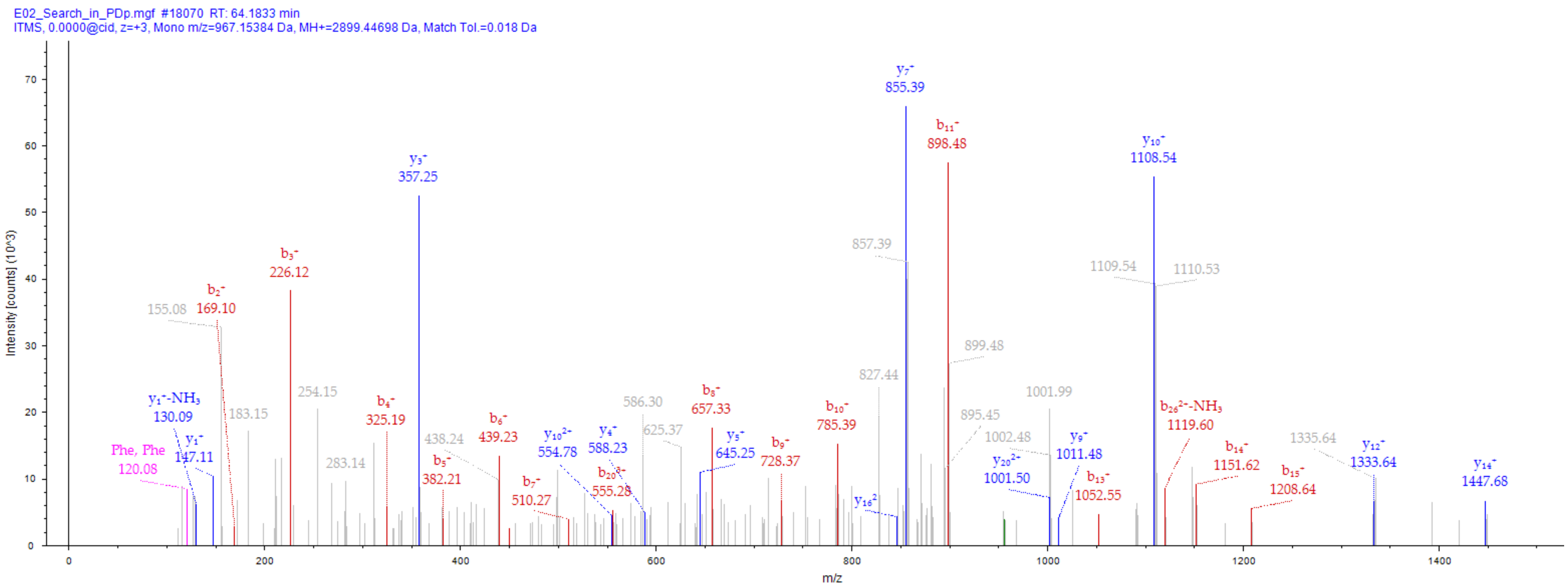

#1	Immonium	b⁺	b²⁺	b³⁺	Seq.	y⁺	y²⁺	y³⁺	#2
1	44.04948	72.04439	36.52583	24.68631	A				31
2	70.06513	169.09715	85.05222	57.03724	P	2828.40924	1414.70826	943.47460	30
3	30.03383	226.11862	113.56295	76.04439	G	2731.35647	1366.18188	911.12368	29
4	72.08078	325.18703	163.09715	109.06720	V	2674.33501	1337.67114	892.11652	28
5	30.03383	382.20850	191.60789	128.07435	G	2575.26660	1288.13694	859.09372	27
6	30.03383	439.22996	220.11862	147.08150	G	2518.24513	1259.62620	840.08656	26
7	44.04948	510.26707	255.63717	170.76054	A	2461.22367	1231.11547	821.07941	25
8	120.08078	657.33549	329.17138	219.78335	F	2390.18655	1195.59692	797.40037	24
9	44.04948	728.37260	364.68994	243.46238	A	2243.11814	1122.06271	748.37756	23
10	30.03383	785.39406	393.20067	262.46954	G	2172.08103	1086.54415	724.69853	22
11	86.09643	898.47813	449.74270	300.16423	I	2115.05956	1058.03342	705.69137	21
12	70.06513	995.53089	498.26908	332.51515	P	2001.97550	1001.49139	667.99668	20
13	30.03383	1052.55236	526.77982	351.52230	G	1904.92274	952.96501	635.64576	19
14	72.08078	1151.62077	576.31402	384.54511	V	1847.90127	924.45427	616.63861	18
15	30.03383	1208.64223	604.82475	403.55226	G	1748.83286	874.92007	583.61580	17
16	70.06513	1305.69500	653.35114	435.90318	P	1691.81139	846.40934	564.60865	16
17	120.08078	1452.76341	726.88534	484.92599	F	1594.75863	797.88295	532.25773	15
18	30.03383	1509.78487	755.39608	503.93314	G	1447.69022	724.34875	483.23492	14
19	30.03383	1566.80634	783.90681	522.94030	G	1390.66875	695.83801	464.22777	13
20	70.06513	1663.85910	832.43319	555.29122	P	1333.64729	667.32728	445.22061	12
21	101.07094	1791.91768	896.46248	597.97741	Q	1236.59453	618.80090	412.86969	11
22	70.06513	1888.97044	944.98886	630.32833	P	1108.53595	554.77161	370.18350	10
23	30.03383	1945.99191	973.49959	649.33549	G	1011.48318	506.24523	337.83258	9
24	72.08078	2045.06032	1023.03380	682.35829	V	954.46172	477.73450	318.82542	8
25	70.06513	2142.11308	1071.56018	714.70921	P	855.39331	428.20029	285.80262	7
26	86.09643	2255.19715	1128.10221	752.40390	L	758.34054	379.67391	253.45170	6
27	30.03383	2312.21861	1156.61294	771.41106	G	645.25648	323.13188	215.75701	5
28	203.99775	2543.20400	1272.10564	848.40618	Y-dichlorin...	588.23502	294.62115	196.74986	4
29	70.06513	2640.25676	1320.63202	880.75710	P	357.24963	179.12845	119.75473	3
30	86.09643	2753.34082	1377.17405	918.45179	I	260.19687	130.60207	87.40381	2
31	101.10732				K	147.11280	74.06004	49.70912	1

APGVGGAFAGIPGVGPFGGPQPGVPLGYPI K

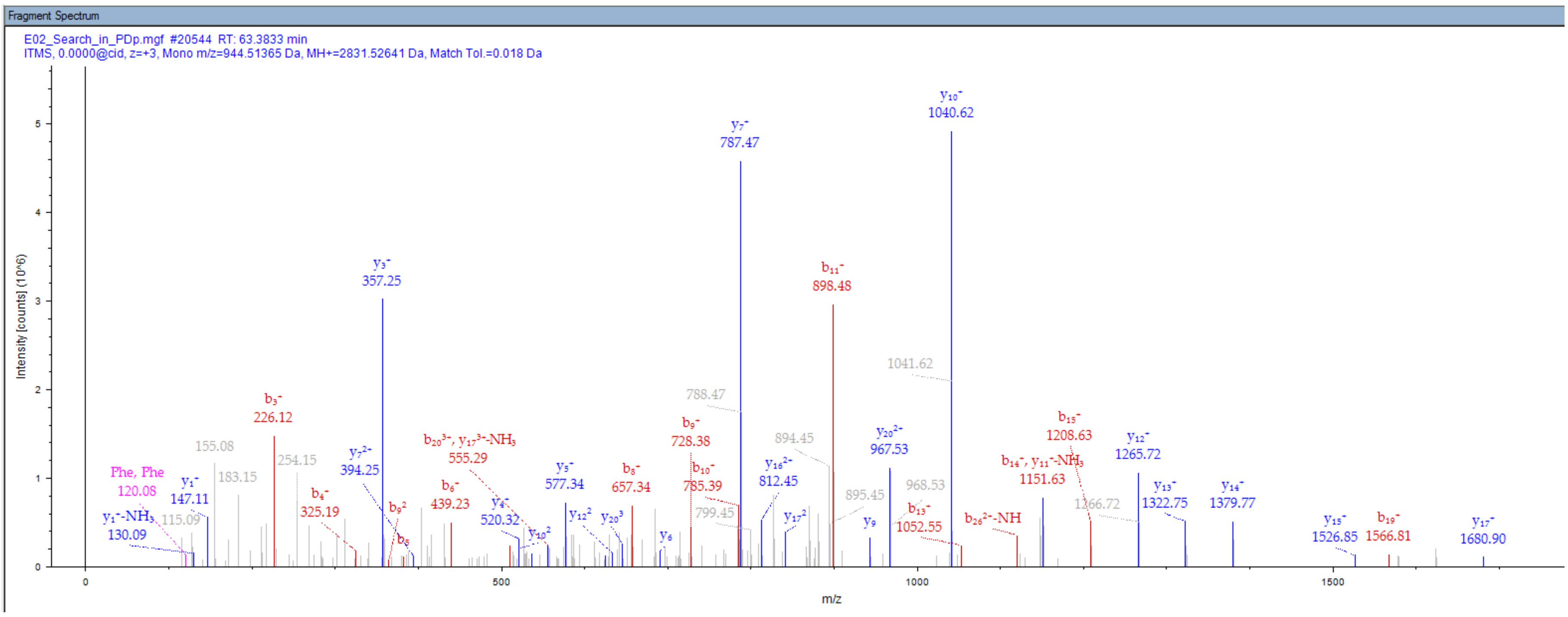

#1	Immonium	b⁺	b²⁺	b³⁺	Seq.	y⁺	y²⁺	y³⁺	#2
1	44.04948	72.04439	36.52583	24.68631	A				31
2	70.06513	169.09715	85.05222	57.03724	P	2760.48718	1380.74723	920.83391	30
3	30.03383	226.11862	113.56295	76.04439	G	2663.43442	1332.22085	888.48299	29
4	72.08078	325.18703	163.09715	109.06720	V	2606.41295	1303.71012	869.47584	28
5	30.03383	382.20850	191.60789	128.07435	G	2507.34454	1254.17591	836.45303	27
6	30.03383	439.22996	220.11862	147.08150	G	2450.32308	1225.66518	817.44588	26
7	44.04948	510.26707	255.63717	170.76054	A	2393.30161	1197.15445	798.43872	25
8	120.08078	657.33549	329.17138	219.78335	F	2322.26450	1161.63589	774.75968	24
9	44.04948	728.37260	364.68994	243.46238	A	2175.19609	1088.10168	725.73688	23
10	30.03383	785.39406	393.20067	262.46954	G	2104.15897	1052.58312	702.05784	22
11	86.09643	898.47813	449.74270	300.16423	I	2047.13751	1024.07239	683.05069	21
12	70.06513	995.53089	498.26908	332.51515	P	1934.05344	967.53036	645.35600	20
13	30.03383	1052.55236	526.77982	351.52230	G	1837.00068	919.00398	613.00508	19
14	72.08078	1151.62077	576.31402	384.54511	V	1779.97922	890.49325	593.99792	18
15	30.03383	1208.64223	604.82475	403.55226	G	1680.91080	840.95904	560.97512	17
16	70.06513	1305.69500	653.35114	435.90318	P	1623.88934	812.44831	541.96796	16
17	120.08078	1452.76341	726.88534	484.92599	F	1526.83658	763.92193	509.61704	15
18	30.03383	1509.78487	755.39608	503.93314	G	1379.76816	690.38772	460.59424	14
19	30.03383	1566.80634	783.90681	522.94030	G	1322.74670	661.87699	441.58708	13
20	70.06513	1663.85910	832.43319	555.29122	P	1265.72523	633.36626	422.57993	12
21	101.07094	1791.91768	896.46248	597.97741	Q	1168.67247	584.83987	390.22901	11
22	70.06513	1888.97044	944.98886	630.32833	P	1040.61389	520.81058	347.54282	10
23	30.03383	1945.99191	973.49959	649.33549	G	943.56113	472.28420	315.19189	9
24	72.08078	2045.06032	1023.03380	682.35829	V	886.53967	443.77347	296.18474	8
25	70.06513	2142.11308	1071.56018	714.70921	P	787.47125	394.23926	263.16194	7
26	86.09643	2255.19715	1128.10221	752.40390	L	690.41849	345.71288	230.81101	6
27	30.03383	2312.21861	1156.61294	771.41106	G	577.33442	289.17085	193.11633	5
28	136.07569	2475.28194	1238.14461	825.76550	Y	520.31296	260.66012	174.10917	4
29	70.06513	2572.33470	1286.67099	858.11642	P	357.24963	179.12845	119.75473	3
30	86.09643	2685.41877	1343.21302	895.81111	I	260.19687	130.60207	87.40381	2
31	101.10732				K	147.11280	74.06004	49.70912	1

LPGGYGLPYTTGK

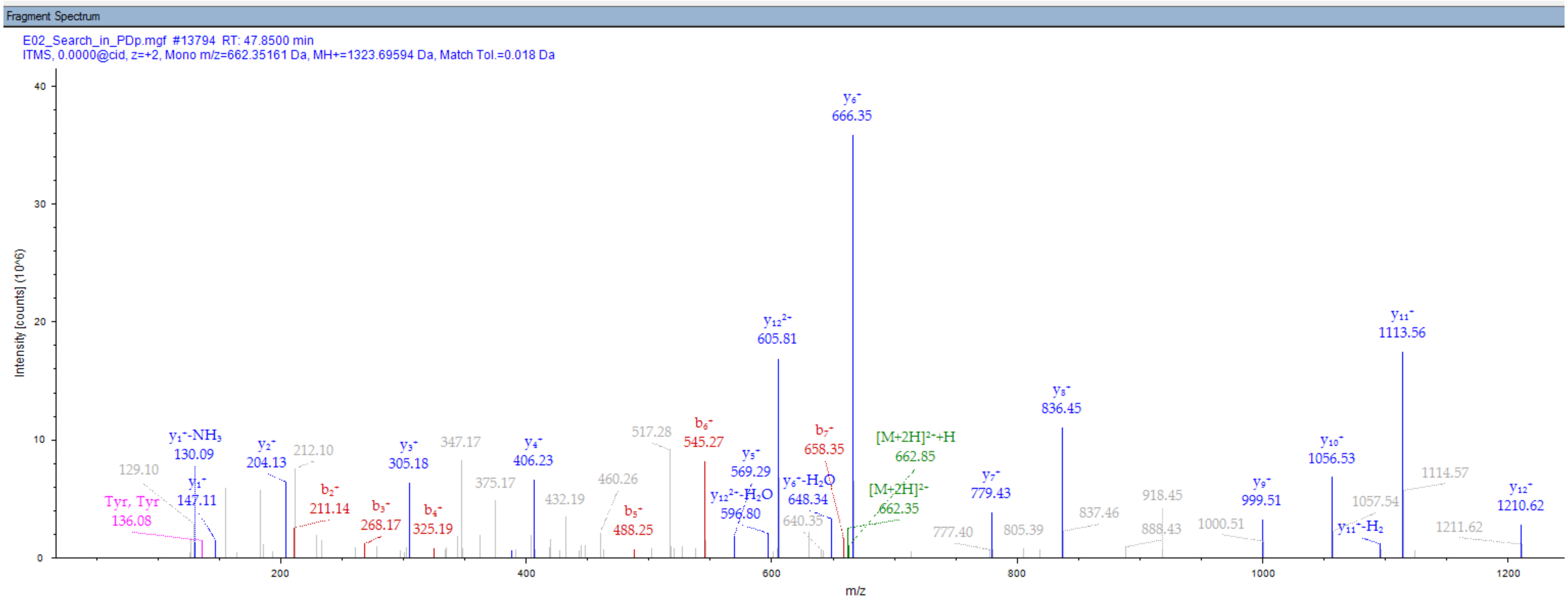

#1	Immonium	b+	b2+	Seq.	y+	y2+	#2
1	86.09643	114.09134	57.54931	L			13
2	70.06513	211.14410	106.07569	P	1210.61026	605.80877	12
3	30.03383	268.16557	134.58642	G	1113.55750	557.28239	11
4	30.03383	325.18703	163.09715	G	1056.53604	528.77166	10
5	136.07569	488.25036	244.62882	Y	999.51457	500.26092	9
6	30.03383	545.27182	273.13955	G	836.45124	418.72926	8
7	86.09643	658.35589	329.68158	L	779.42978	390.21853	7
8	70.06513	755.40865	378.20796	P	666.34572	333.67650	6
9	136.07569	918.47198	459.73963	Y	569.29295	285.15011	5
10	74.06004	1019.51966	510.26347	T	406.22962	203.61845	4
11	74.06004	1120.56734	560.78731	T	305.18195	153.09461	3
12	30.03383	1177.58880	589.29804	G	204.13427	102.57077	2
13	101.10732			K	147.11280	74.06004	1

LPGGYGLPYTTGK,
Y5-Chlorination (33.96103 Da)

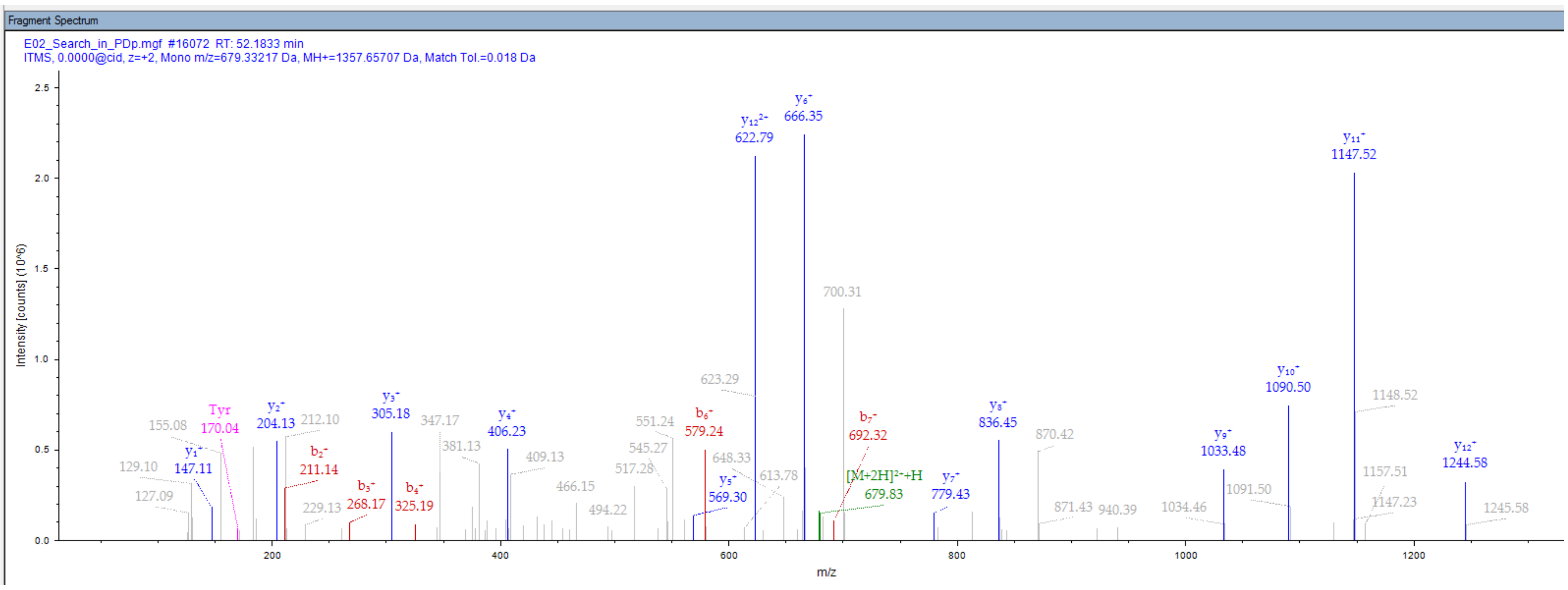

#1	Immonium	b+	b2+	Seq.	y+	y2+	#2
1	86.09643	114.09134	57.54931	L			13
2	70.06513	211.14410	106.07569	P	1244.57129	622.78928	12
3	30.03383	268.16557	134.58642	G	1147.51853	574.26290	11
4	30.03383	325.18703	163.09715	G	1090.49706	545.75217	10
5	170.03672	522.21139	261.60933	Y-Chlorinat...	1033.47560	517.24144	9
6	30.03383	579.23285	290.12006	G	836.45124	418.72926	8
7	86.09643	692.31692	346.66210	L	779.42978	390.21853	7
8	70.06513	789.36968	395.18848	P	666.34572	333.67650	6
9	136.07569	952.43301	476.72014	Y	569.29295	285.15011	5
10	74.06004	1053.48069	527.24398	T	406.22962	203.61845	4
11	74.06004	1154.52836	577.76782	T	305.18195	153.09461	3
12	30.03383	1211.54983	606.27855	G	204.13427	102.57077	2
13	101.10732			K	147.11280	74.06004	1

LPGGYGLPYTTGK,
Y5-dichlorination (67.92206 Da)

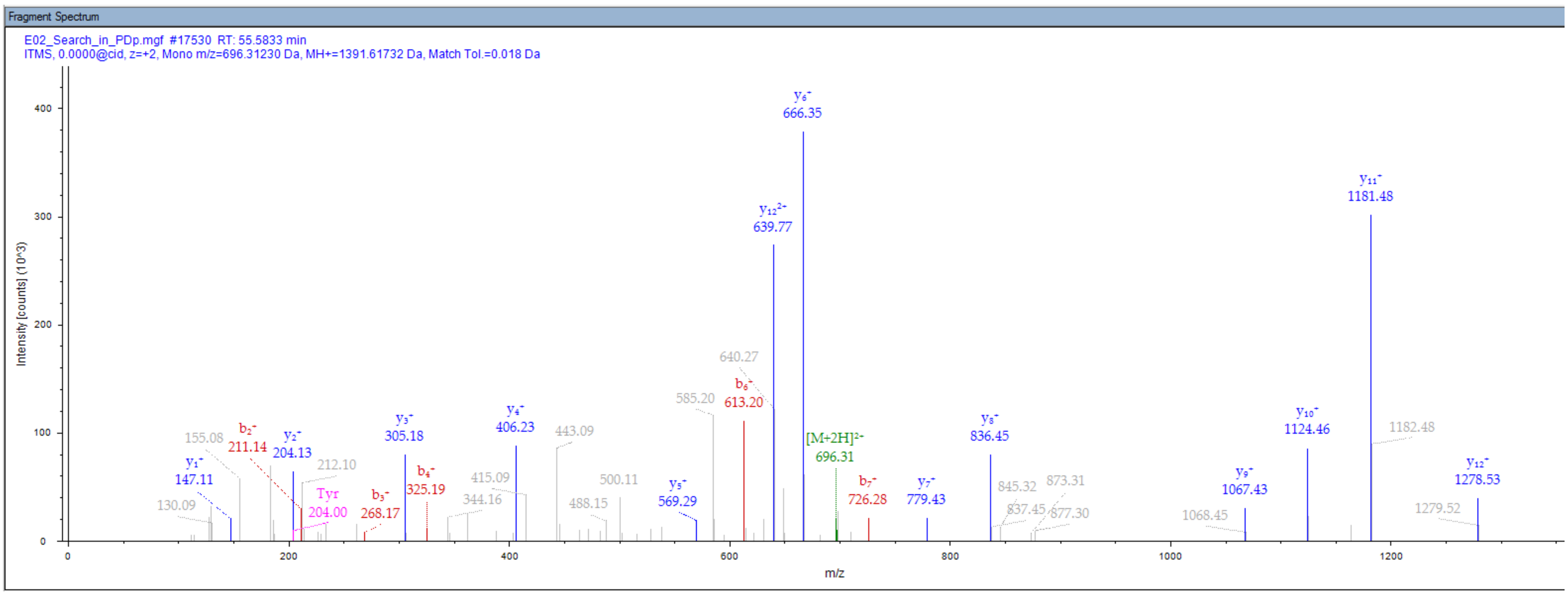

#1	Immonium	b+	b2+	Seq.	y+	y2+	#2
1	86.09643	114.09134	57.54931	L			13
2	70.06513	211.14410	106.07569	P	1278.53232	639.76980	12
3	30.03383	268.16557	134.58642	G	1181.47956	591.24342	11
4	30.03383	325.18703	163.09715	G	1124.45809	562.73268	10
5	203.99775	556.17242	278.58985	Y-dichlorin...	1067.43663	534.22195	9
6	30.03383	613.19388	307.10058	G	836.45124	418.72926	8
7	86.09643	726.27794	363.64261	L	779.42978	390.21853	7
8	70.06513	823.33071	412.16899	P	666.34572	333.67650	6
9	136.07569	986.39404	493.70066	Y	569.29295	285.15011	5
10	74.06004	1087.44171	544.22450	T	406.22962	203.61845	4
11	74.06004	1188.48939	594.74833	T	305.18195	153.09461	3
12	30.03383	1245.51086	623.25907	G	204.13427	102.57077	2
13	101.10732			K	147.11280	74.06004	1

LPGGYGLPYTTGK,
Y9-Chlorination (33.96103 Da)

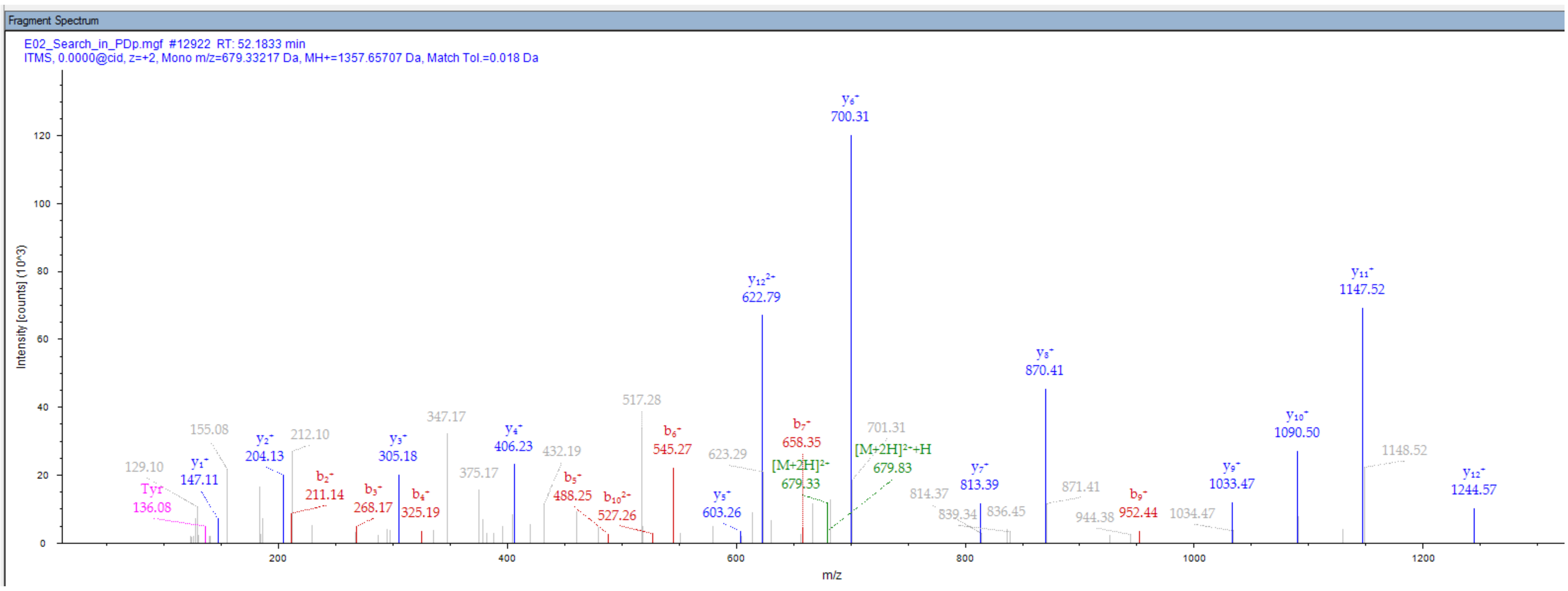

#1	Immonium	b⁺	b²⁺	Seq.	y⁺	y²⁺	#2
1	86.09643	114.09134	57.54931	L			13
2	70.06513	211.14410	106.07569	P	1244.57129	622.78928	12
3	30.03383	268.16557	134.58642	G	1147.51853	574.26290	11
4	30.03383	325.18703	163.09715	G	1090.49706	545.75217	10
5	136.07569	488.25036	244.62882	Y	1033.47560	517.24144	9
6	30.03383	545.27182	273.13955	G	870.41227	435.70977	8
7	86.09643	658.35589	329.68158	L	813.39081	407.19904	7
8	70.06513	755.40865	378.20796	P	700.30675	350.65701	6
9	170.03672	952.43301	476.72014	Y-Chlorinat...	603.25398	302.13063	5
10	74.06004	1053.48069	527.24398	T	406.22962	203.61845	4
11	74.06004	1154.52836	577.76782	T	305.18195	153.09461	3
12	30.03383	1211.54983	606.27855	G	204.13427	102.57077	2
13	101.10732			K	147.11280	74.06004	1

LPGGYGLPYTTGK,
Y9-dichlorination (67.92206 Da)

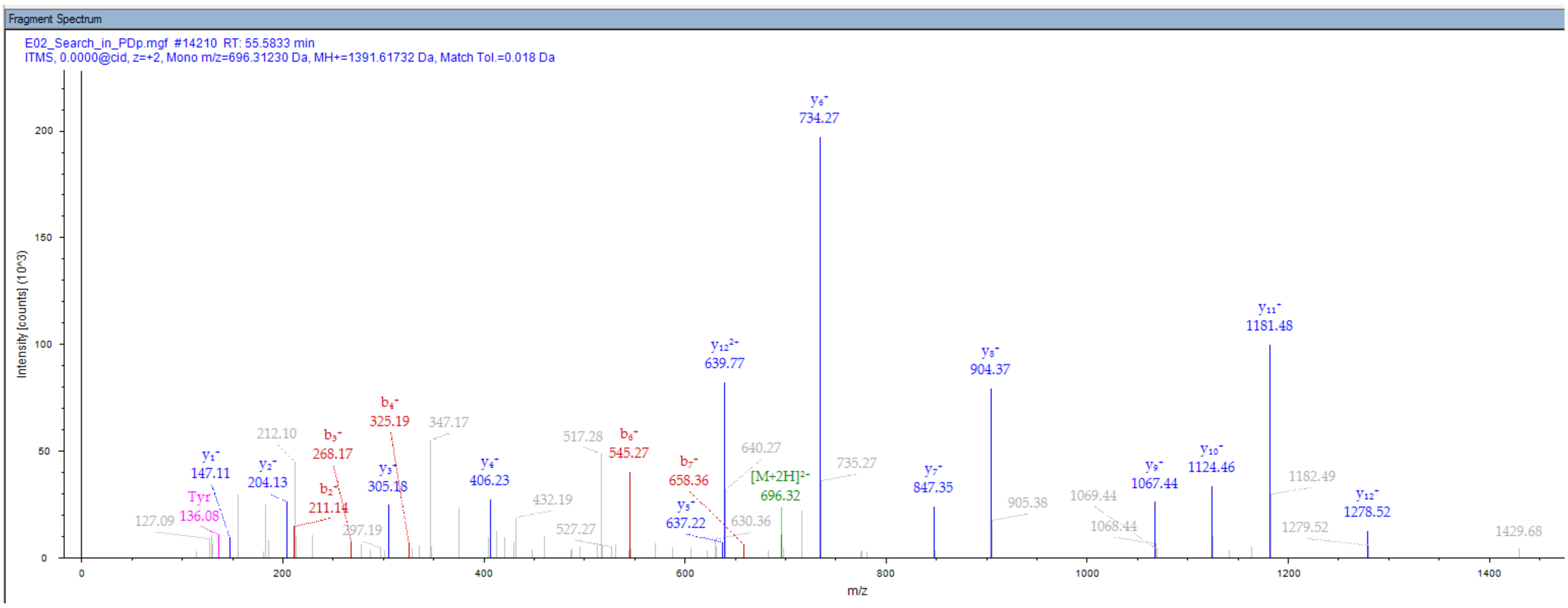

#1	Immonium	b^{+}	b^{2+}	Seq.	y^{+}	y^{2+}	#2
1	86.09643	114.09134	57.54931	L			13
2	70.06513	211.14410	106.07569	P	1278.53232	639.76980	12
3	30.03383	268.16557	134.58642	G	1181.47956	591.24342	11
4	30.03383	325.18703	163.09715	G	1124.45809	562.73268	10
5	136.07569	488.25036	244.62882	Y	1067.43663	534.22195	9
6	30.03383	545.27182	273.13955	G	904.37330	452.69029	8
7	86.09643	658.35589	329.68158	L	847.35184	424.17956	7
8	70.06513	755.40865	378.20796	P	734.26777	367.63752	6
9	203.99775	986.39404	493.70066	Y-dichlorin...	637.21501	319.11114	5
10	74.06004	1087.44171	544.22450	T	406.22962	203.61845	4
11	74.06004	1188.48939	594.74833	T	305.18195	153.09461	3
12	30.03383	1245.51086	623.25907	G	204.13427	102.57077	2
13	101.10732			K	147.11280	74.06004	1

LPGGYGLPYTTGK, Y5-Chlorination (33.96103 Da) and Y9-Chlorination (33.96103 Da)

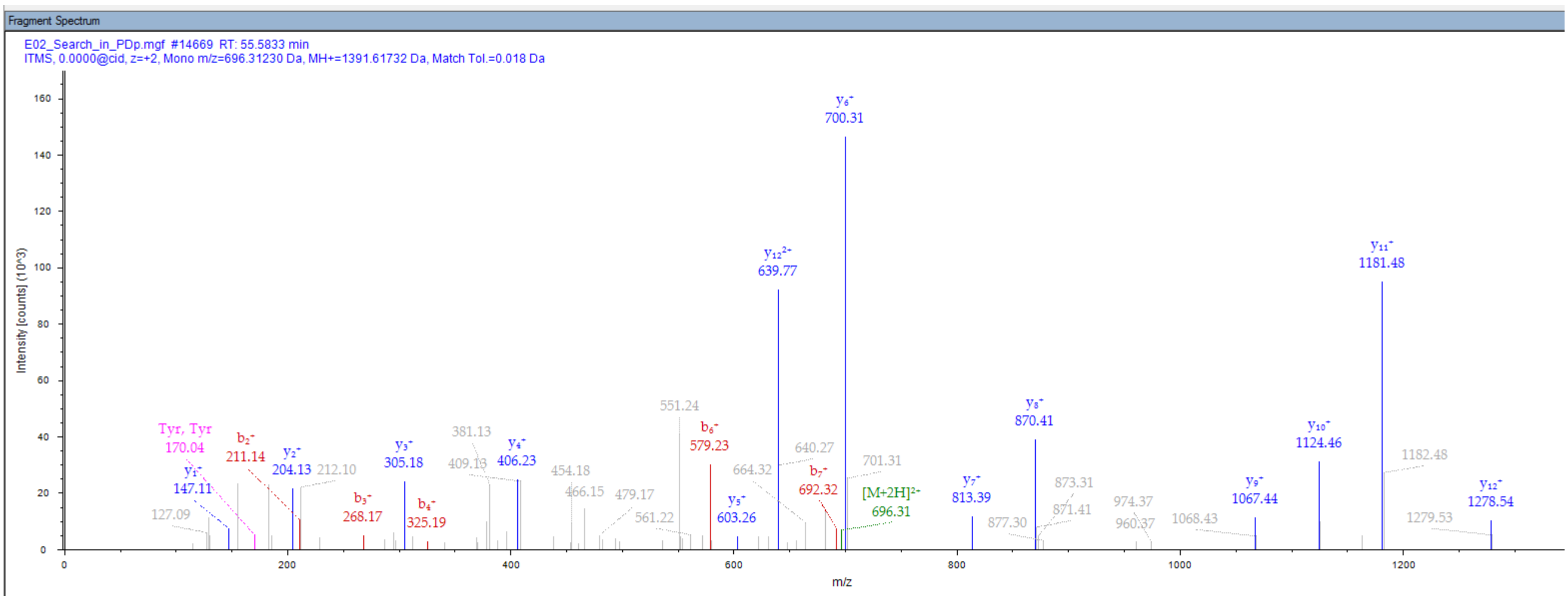

#1	Immonium	b+	b2+	Seq.	y+	y2+	#2
1	86.09643	114.09134	57.54931	L			13
2	70.06513	211.14410	106.07569	P	1278.53232	639.76980	12
3	30.03383	268.16557	134.58642	G	1181.47956	591.24342	11
4	30.03383	325.18703	163.09715	G	1124.45809	562.73268	10
5	170.03672	522.21139	261.60933	Y-Chlorinat...	1067.43663	534.22195	9
6	30.03383	579.23285	290.12006	G	870.41227	435.70977	8
7	86.09643	692.31692	346.66210	L	813.39081	407.19904	7
8	70.06513	789.36968	395.18848	P	700.30675	350.65701	6
9	170.03672	986.39404	493.70066	Y-Chlorinat...	603.25398	302.13063	5
10	74.06004	1087.44171	544.22450	T	406.22962	203.61845	4
11	74.06004	1188.48939	594.74833	T	305.18195	153.09461	3
12	30.03383	1245.51086	623.25907	G	204.13427	102.57077	2
13	101.10732			K	147.11280	74.06004	1

LPGGYGLPYTTGK,
Y5-Chlorination (33.96103 Da) and
Y9-dichlorination (67.92206 Da)

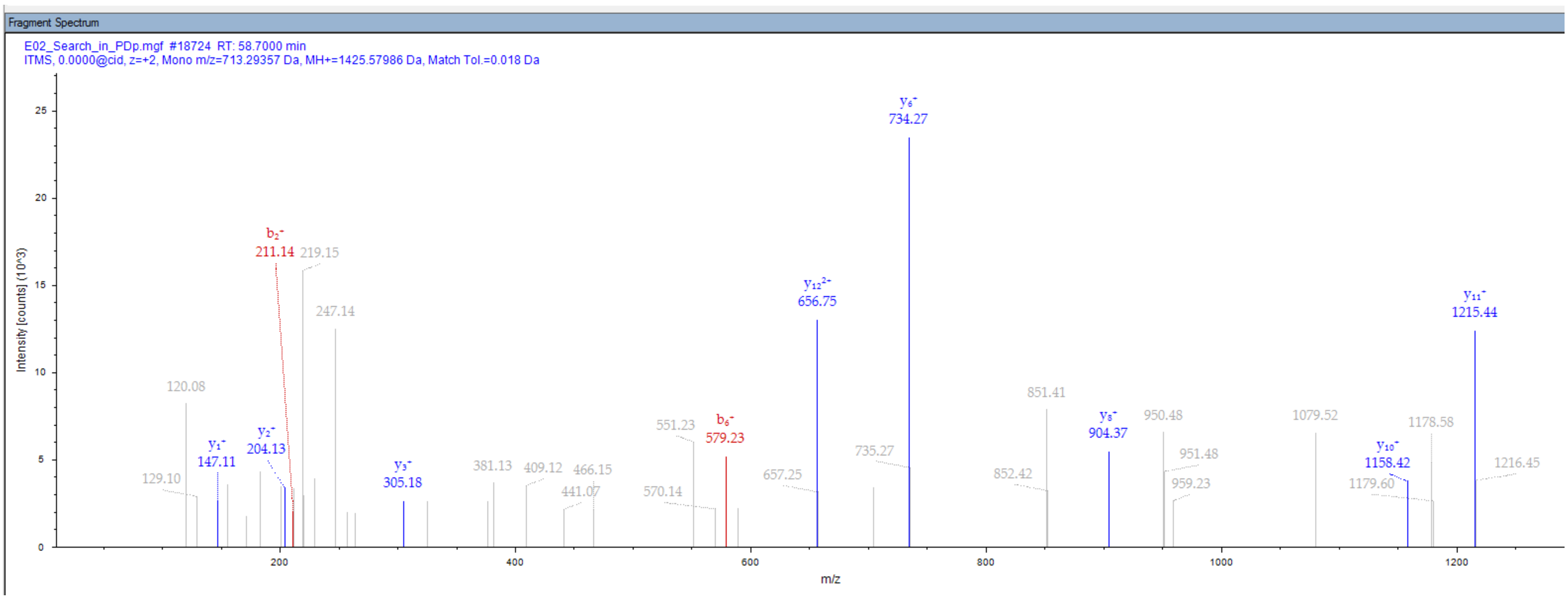

#1	Immonium	b⁺	b²⁺	Seq.	y⁺	y²⁺	#2
1	86.09643	114.09134	57.54931	L			13
2	70.06513	211.14410	106.07569	P	1312.49335	656.75031	12
3	30.03383	268.16557	134.58642	G	1215.44058	608.22393	11
4	30.03383	325.18703	163.09715	G	1158.41912	579.71320	10
5	170.03672	522.21139	261.60933	Y-Chlorinat...	1101.39766	551.20247	9
6	30.03383	579.23285	290.12006	G	904.37330	452.69029	8
7	86.09643	692.31692	346.66210	L	847.35184	424.17956	7
8	70.06513	789.36968	395.18848	P	734.26777	367.63752	6
9	203.99775	1020.35506	510.68117	Y-dichlorin...	637.21501	319.11114	5
10	74.06004	1121.40274	561.20501	T	406.22962	203.61845	4
11	74.06004	1222.45042	611.72885	T	305.18195	153.09461	3
12	30.03383	1279.47188	640.23958	G	204.13427	102.57077	2
13	101.10732			K	147.11280	74.06004	1

LPYGYGPGGVAGAAGK

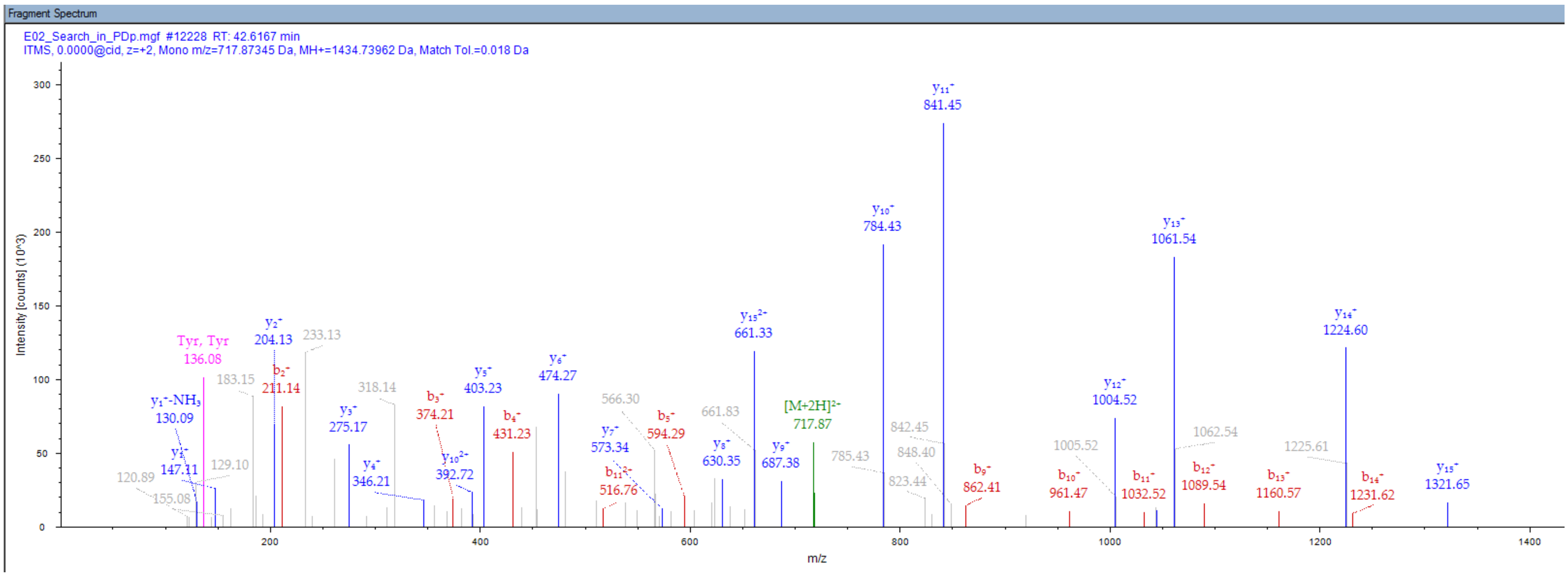

#1	Immonium	b+	b2+	Seq.	y+	y2+	#2
1	86.09643	114.09134	57.54931	L			16
2	70.06513	211.14410	106.07569	P	1321.65353	661.33040	15
3	136.07569	374.20743	187.60735	Y	1224.60076	612.80402	14
4	30.03383	431.22890	216.11809	G	1061.53743	531.27236	13
5	136.07569	594.29223	297.64975	Y	1004.51597	502.76162	12
6	30.03383	651.31369	326.16048	G	841.45264	421.22996	11
7	70.06513	748.36645	374.68686	P	784.43118	392.71923	10
8	30.03383	805.38792	403.19760	G	687.37841	344.19285	9
9	30.03383	862.40938	431.70833	G	630.35695	315.68211	8
10	72.08078	961.47779	481.24254	V	573.33549	287.17138	7
11	44.04948	1032.51491	516.76109	A	474.26707	237.63717	6
12	30.03383	1089.53637	545.27182	G	403.22996	202.11862	5
13	44.04948	1160.57348	580.79038	A	346.20850	173.60789	4
14	44.04948	1231.61060	616.30894	A	275.17138	138.08933	3
15	30.03383	1288.63206	644.81967	G	204.13427	102.57077	2
16	101.10732			K	147.11280	74.06004	1

LPYGYGPGGVAGAAGK, Y3-Chlorination (33.96103 Da)

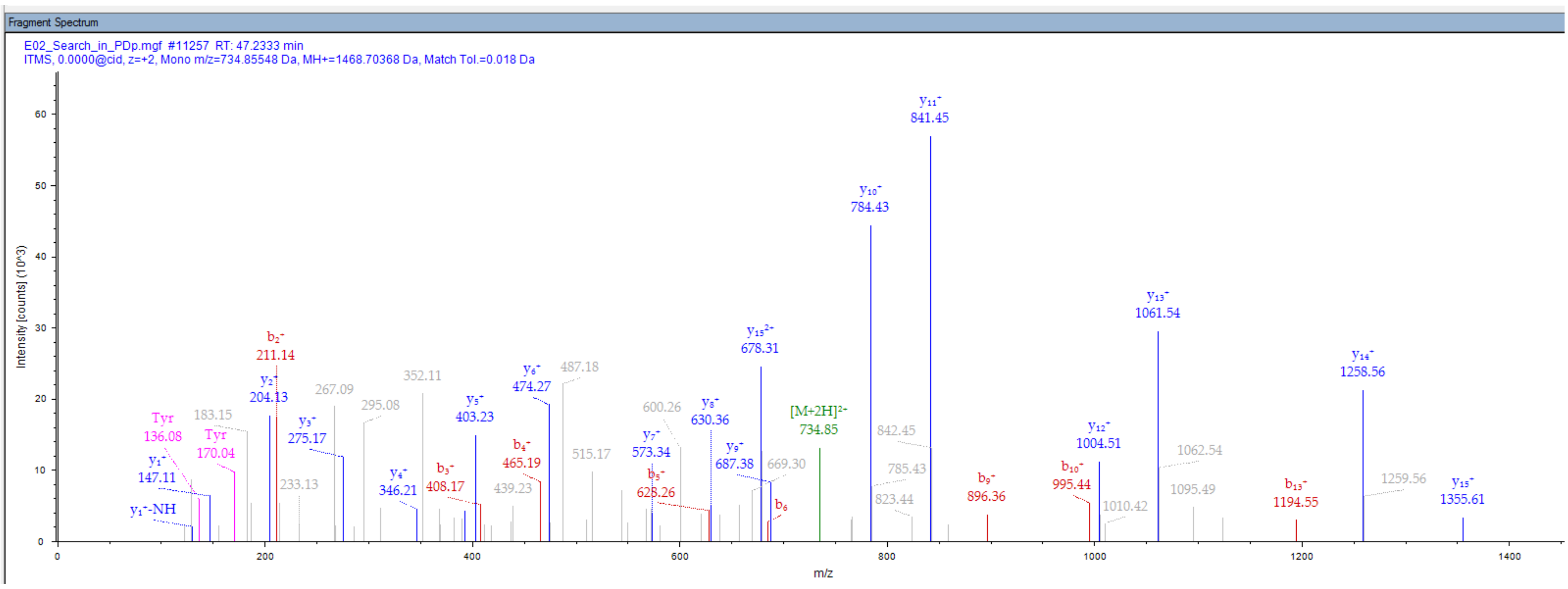

#1	Immonium	b⁺	b²⁺	Seq.	y⁺	y²⁺	#2
1	86.09643	114.09134	57.54931	L			16
2	70.06513	211.14410	106.07569	P	1355.61455	678.31092	15
3	170.03672	408.16846	204.58787	Y-Chlorinat...	1258.56179	629.78453	14
4	30.03383	465.18992	233.09860	G	1061.53743	531.27236	13
5	136.07569	628.25325	314.63026	Y	1004.51597	502.76162	12
6	30.03383	685.27472	343.14100	G	841.45264	421.22996	11
7	70.06513	782.32748	391.66738	P	784.43118	392.71923	10
8	30.03383	839.34894	420.17811	G	687.37841	344.19285	9
9	30.03383	896.37041	448.68884	G	630.35695	315.68211	8
10	72.08078	995.43882	498.22305	V	573.33549	287.17138	7
11	44.04948	1066.47594	533.74161	A	474.26707	237.63717	6
12	30.03383	1123.49740	562.25234	G	403.22996	202.11862	5
13	44.04948	1194.53451	597.77089	A	346.20850	173.60789	4
14	44.04948	1265.57163	633.28945	A	275.17138	138.08933	3
15	30.03383	1322.59309	661.80018	G	204.13427	102.57077	2
16	101.10732			K	147.11280	74.06004	1

LPYGYGPGGVAGAAGK, Y5-Chlorination (33.96103 Da)

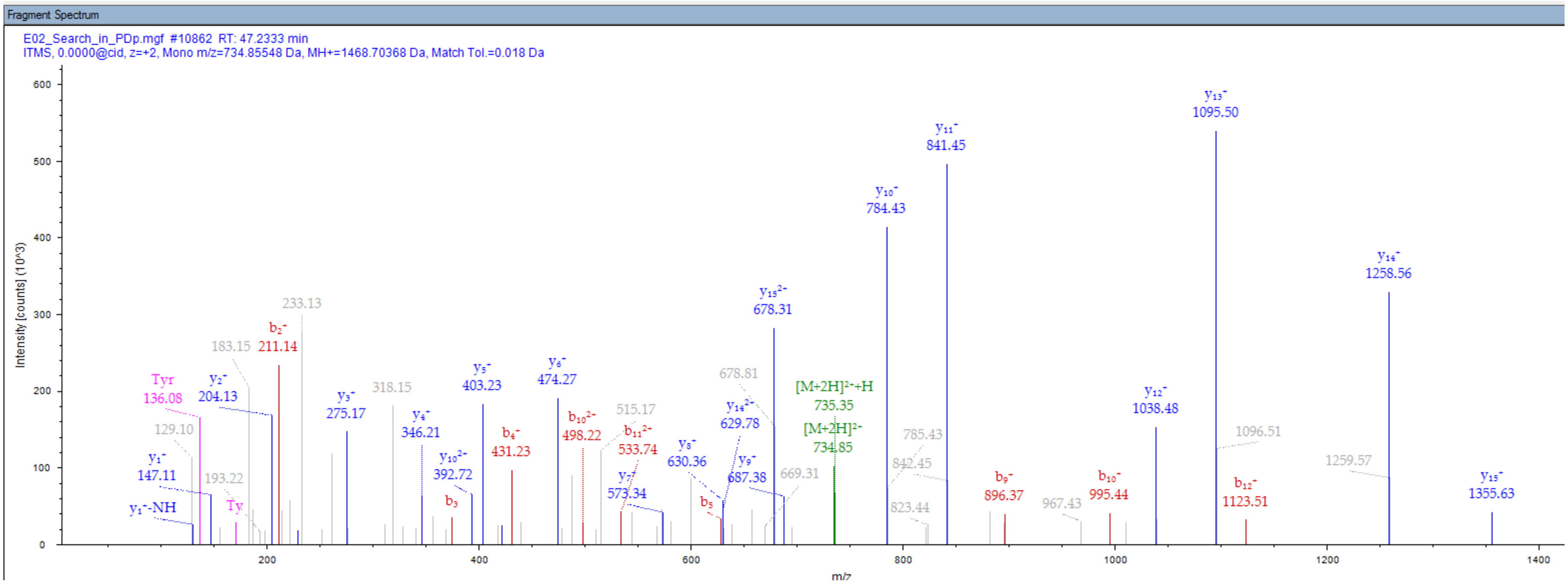

#1	Immonium	b^+	b^{2+}	Seq.	y^+	y^{2+}	#2
1	86.09643	114.09134	57.54931	L			16
2	70.06513	211.14410	106.07569	P	1355.61455	678.31092	15
3	136.07569	374.20743	187.60735	Y	1258.56179	629.78453	14
4	30.03383	431.22890	216.11809	G	1095.49846	548.25287	13
5	170.03672	628.25325	314.63026	Y-Chlorinat...	1038.47700	519.74214	12
6	30.03383	685.27472	343.14100	G	841.45264	421.22996	11
7	70.06513	782.32748	391.66738	P	784.43118	392.71923	10
8	30.03383	839.34894	420.17811	G	687.37841	344.19285	9
9	30.03383	896.37041	448.68884	G	630.35695	315.68211	8
10	72.08078	995.43882	498.22305	V	573.33549	287.17138	7
11	44.04948	1066.47594	533.74161	A	474.26707	237.63717	6
12	30.03383	1123.49740	562.25234	G	403.22996	202.11862	5
13	44.04948	1194.53451	597.77089	A	346.20850	173.60789	4
14	44.04948	1265.57163	633.28945	A	275.17138	138.08933	3
15	30.03383	1322.59309	661.80018	G	204.13427	102.57077	2
16	101.10732			K	147.11280	74.06004	1

LPYGYGPGGVAGAAGK, Y3-dichlorination (67.92206 Da)

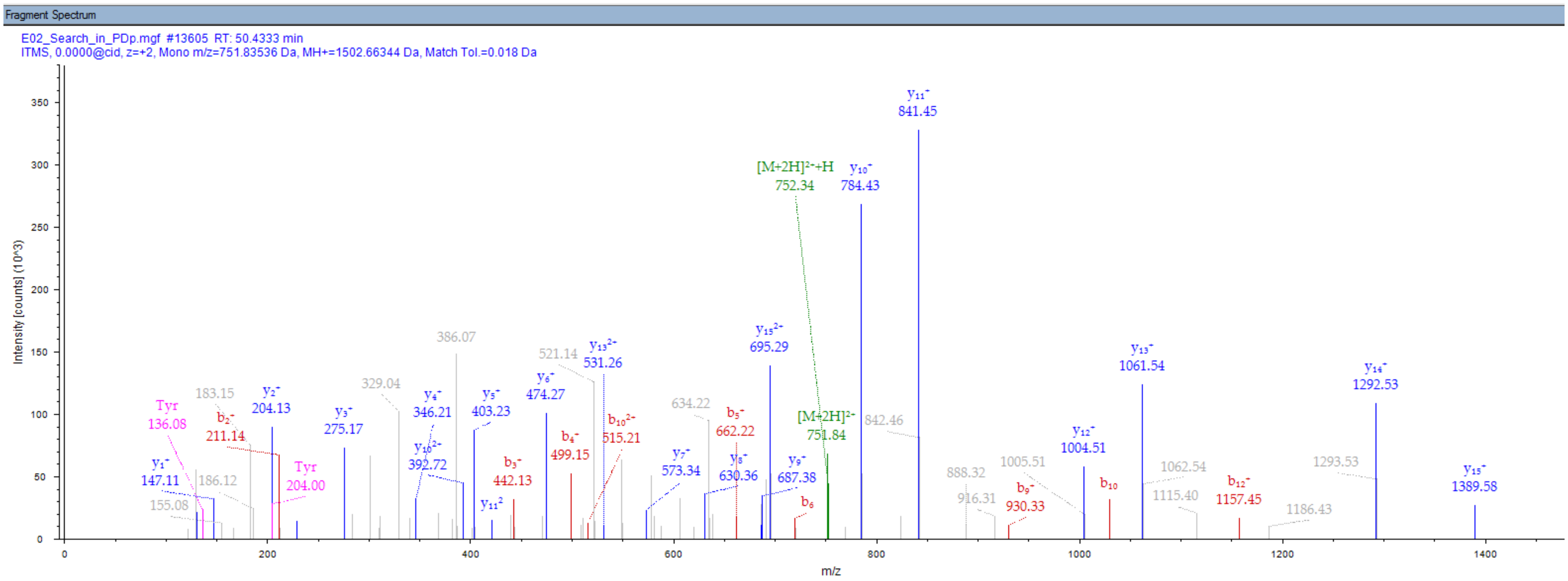

#1	Immonium	b+	b2+	Seq.	y+	y2+	#2
1	86.09643	114.09134	57.54931	L			16
2	70.06513	211.14410	106.07569	P	1389.57558	695.29143	15
3	203.99775	442.12949	221.56838	Y-dichlorin...	1292.52282	646.76505	14
4	30.03383	499.15095	250.07911	G	1061.53743	531.27236	13
5	136.07569	662.21428	331.61078	Y	1004.51597	502.76162	12
6	30.03383	719.23574	360.12151	G	841.45264	421.22996	11
7	70.06513	816.28851	408.64789	P	784.43118	392.71923	10
8	30.03383	873.30997	437.15862	G	687.37841	344.19285	9
9	30.03383	930.33143	465.66936	G	630.35695	315.68211	8
10	72.08078	1029.39985	515.20356	V	573.33549	287.17138	7
11	44.04948	1100.43696	550.72212	A	474.26707	237.63717	6
12	30.03383	1157.45843	579.23285	G	403.22996	202.11862	5
13	44.04948	1228.49554	614.75141	A	346.20850	173.60789	4
14	44.04948	1299.53265	650.26997	A	275.17138	138.08933	3
15	30.03383	1356.55412	678.78070	G	204.13427	102.57077	2
16	101.10732			K	147.11280	74.06004	1

LPYGYGPGGVAGAAGK, Y5-dichlorination (67.92206 Da)

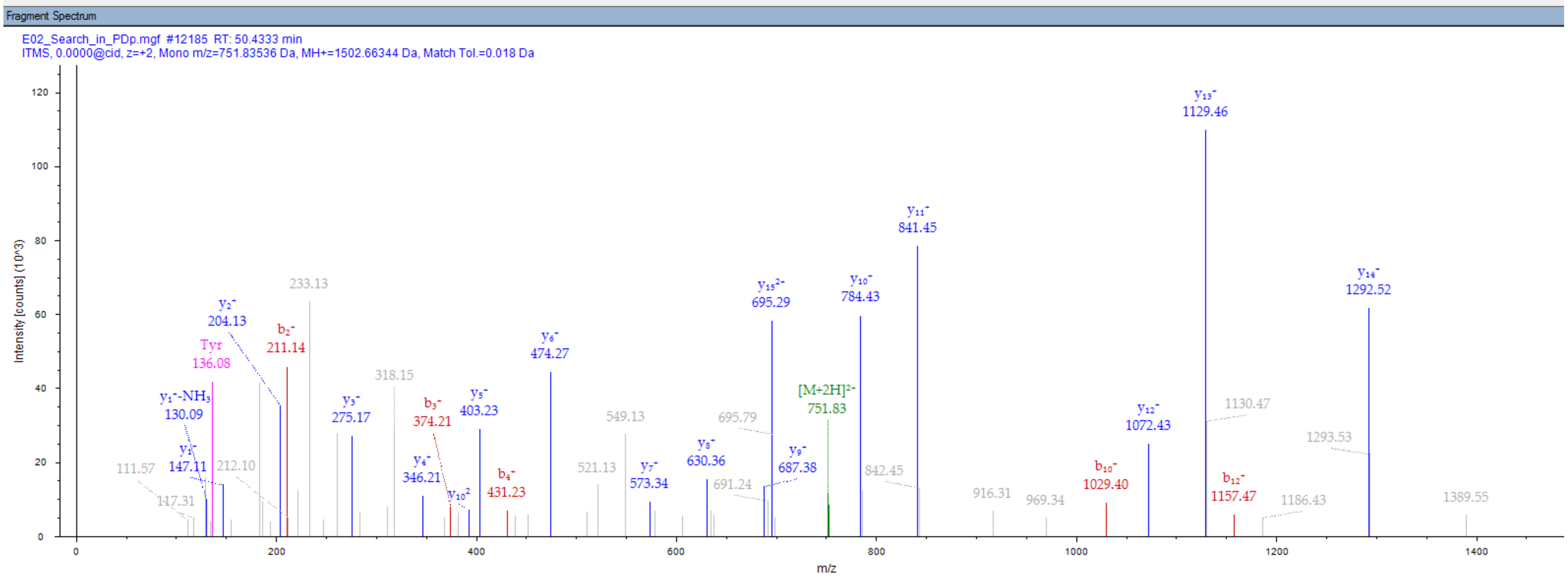

#1	Immonium	b⁺	b²⁺	Seq.	y⁺	y²⁺	#2
1	86.09643	114.09134	57.54931	L			16
2	70.06513	211.14410	106.07569	P	1389.57558	695.29143	15
3	136.07569	374.20743	187.60735	Y	1292.52282	646.76505	14
4	30.03383	431.22890	216.11809	G	1129.45949	565.23338	13
5	203.99775	662.21428	331.61078	Y-dichlorin...	1072.43803	536.72265	12
6	30.03383	719.23574	360.12151	G	841.45264	421.22996	11
7	70.06513	816.28851	408.64789	P	784.43118	392.71923	10
8	30.03383	873.30997	437.15862	G	687.37841	344.19285	9
9	30.03383	930.33143	465.66936	G	630.35695	315.68211	8
10	72.08078	1029.39985	515.20356	V	573.33549	287.17138	7
11	44.04948	1100.43696	550.72212	A	474.26707	237.63717	6
12	30.03383	1157.45843	579.23285	G	403.22996	202.11862	5
13	44.04948	1228.49554	614.75141	A	346.20850	173.60789	4
14	44.04948	1299.53265	650.26997	A	275.17138	138.08933	3
15	30.03383	1356.55412	678.78070	G	204.13427	102.57077	2
16	101.10732			K	147.11280	74.06004	1

LPYGYGPGGVAGAAGK, Y3-Chlorination (33.96103 Da) and Y5-Chlorination (33.96103 Da)

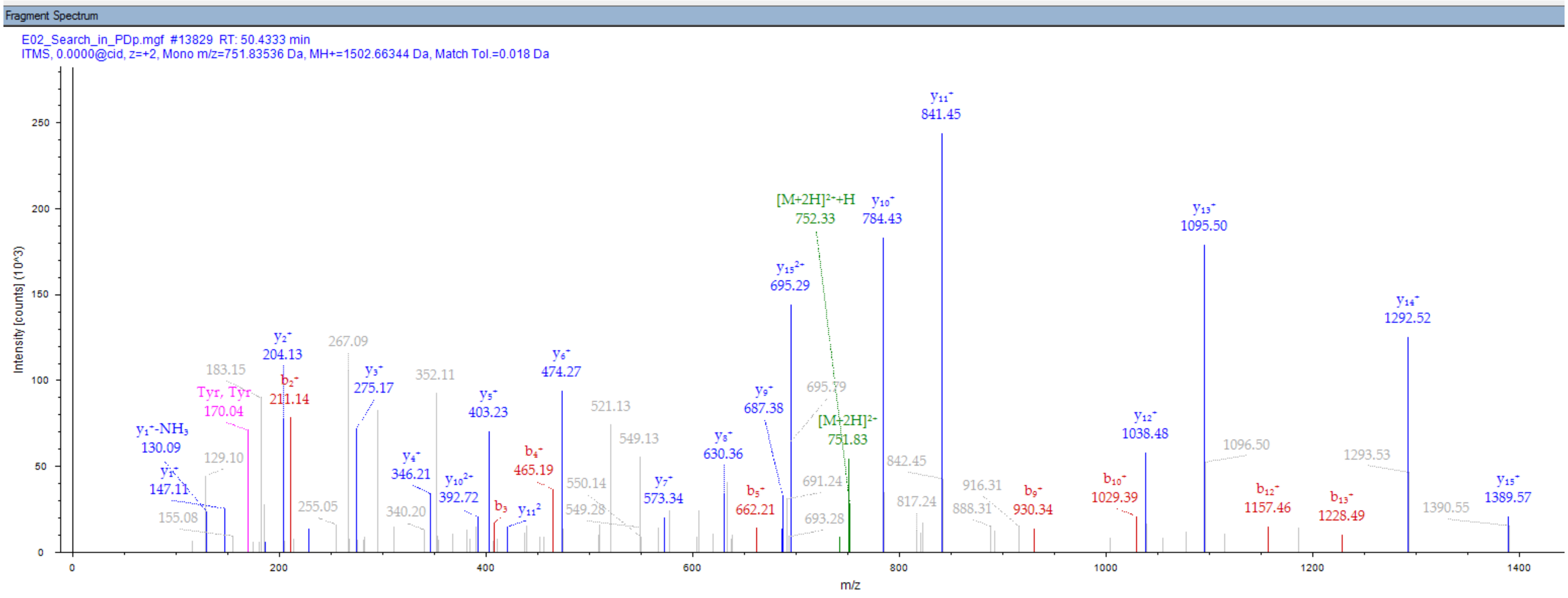

#1	Immonium	b^+	b^{2+}	Seq.	y^+	y^{2+}	#2
1	86.09643	114.09134	57.54931	L			16
2	70.06513	211.14410	106.07569	P	1389.57558	695.29143	15
3	170.03672	408.16846	204.58787	Y-Chlorinat...	1292.52282	646.76505	14
4	30.03383	465.18992	233.09860	G	1095.49846	548.25287	13
5	170.03672	662.21428	331.61078	Y-Chlorinat...	1038.47700	519.74214	12
6	30.03383	719.23574	360.12151	G	841.45264	421.22996	11
7	70.06513	816.28851	408.64789	P	784.43118	392.71923	10
8	30.03383	873.30997	437.15862	G	687.37841	344.19285	9
9	30.03383	930.33144	465.66936	G	630.35695	315.68211	8
10	72.08078	1029.39985	515.20356	V	573.33549	287.17138	7
11	44.04948	1100.43696	550.72212	A	474.26707	237.63717	6
12	30.03383	1157.45843	579.23285	G	403.22996	202.11862	5
13	44.04948	1228.49554	614.75141	A	346.20850	173.60789	4
14	44.04948	1299.53265	650.26997	A	275.17138	138.08933	3
15	30.03383	1356.55412	678.78070	G	204.13427	102.57077	2
16	101.10732			K	147.11280	74.06004	1

AGYPTGTGVGPQAAAAAAK

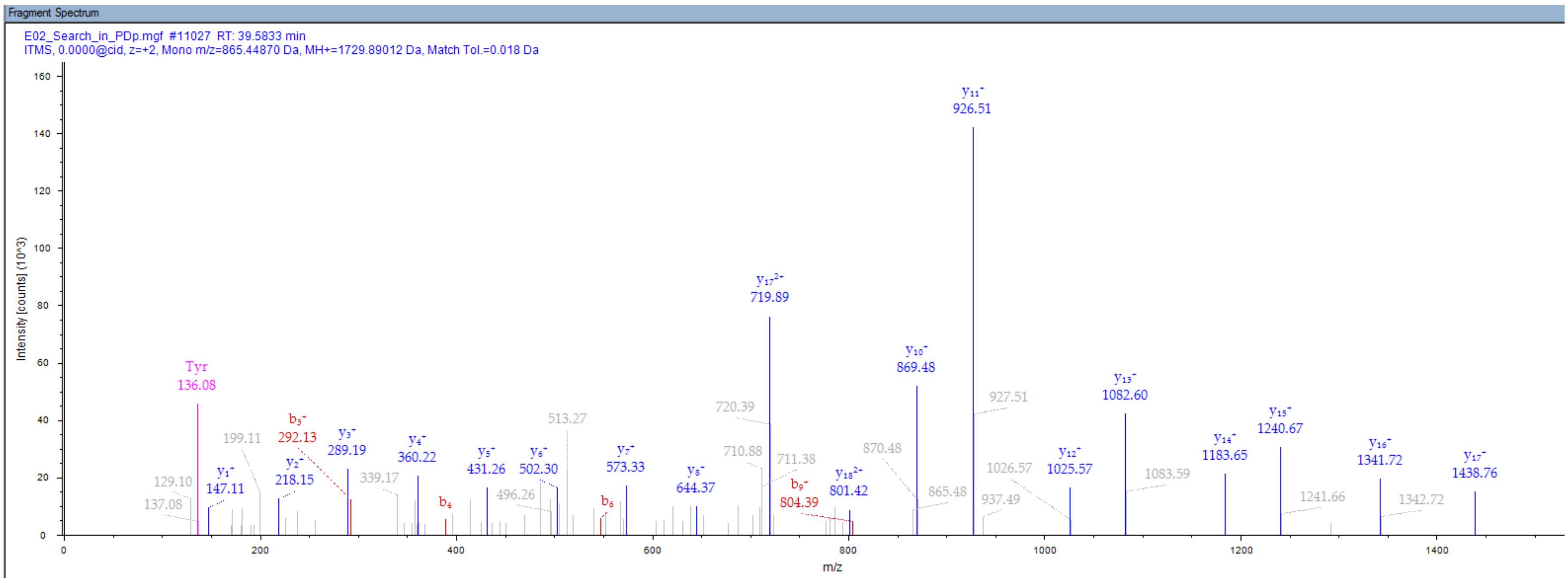

#1	Immonium	b+	b2+	Seq.	y+	y2+	#2
1	44.04948	72.04439	36.52583	A			20
2	30.03383	129.06585	65.03657	G	1658.84966	829.92847	19
3	136.07569	292.12918	146.56823	Y	1601.82820	801.41774	18
4	70.06513	389.18195	195.09461	P	1438.76487	719.88607	17
5	74.06004	490.22962	245.61845	T	1341.71210	671.35969	16
6	30.03383	547.25109	274.12918	G	1240.66443	620.83585	15
7	74.06004	648.29877	324.65302	T	1183.64296	592.32512	14
8	30.03383	705.32023	353.16375	G	1082.59528	541.80128	13
9	72.08078	804.38864	402.69796	V	1025.57382	513.29055	12
10	30.03383	861.41011	431.20869	G	926.50541	463.75634	11
11	70.06513	958.46287	479.73507	P	869.48394	435.24561	10
12	101.07094	1086.52145	543.76436	Q	772.43118	386.71923	9
13	44.04948	1157.55856	579.28292	A	644.37260	322.68994	8
14	44.04948	1228.59568	614.80148	A	573.33549	287.17138	7
15	44.04948	1299.63279	650.32003	A	502.29837	251.65282	6
16	44.04948	1370.66990	685.83859	A	431.26126	216.13427	5
17	44.04948	1441.70702	721.35715	A	360.22415	180.61571	4
18	44.04948	1512.74413	756.87570	A	289.18703	145.09715	3
19	44.04948	1583.78125	792.39426	A	218.14992	109.57860	2
20	101.10732			K	147.11280	74.06004	1

AGYPTGTGVGPQAAAAAAK, Y3-Chlorination (33.96103 Da)

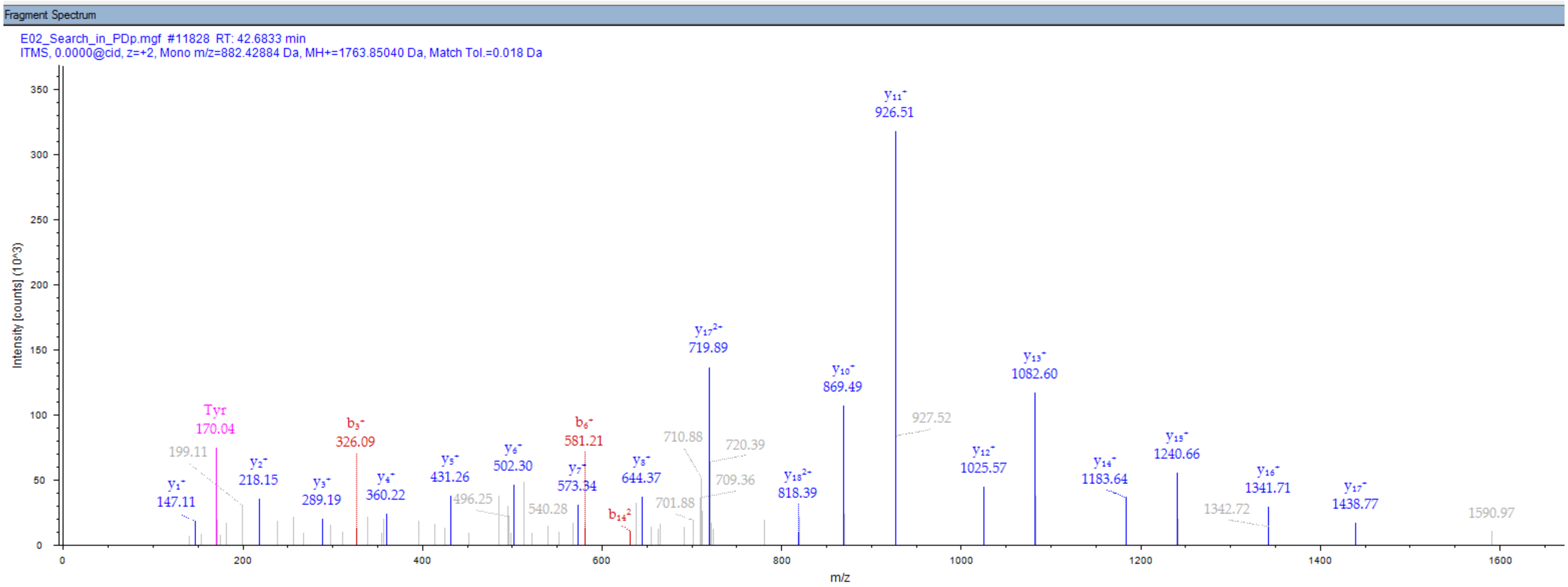

#1	Immonium	b^+	b^{2+}	Seq.	y^+	y^{2+}	#2
1	44.04948	72.04439	36.52583	A			20
2	30.03383	129.06585	65.03657	G	1692.81069	846.90898	19
3	170.03672	326.09021	163.54874	Y-Chlorinat...	1635.78922	818.39825	18
4	70.06513	423.14297	212.07513	P	1438.76487	719.88607	17
5	74.06004	524.19065	262.59896	T	1341.71210	671.35969	16
6	30.03383	581.21212	291.10970	G	1240.66443	620.83585	15
7	74.06004	682.25979	341.63354	T	1183.64296	592.32512	14
8	30.03383	739.28126	370.14427	G	1082.59528	541.80128	13
9	72.08078	838.34967	419.67847	V	1025.57382	513.29055	12
10	30.03383	895.37114	448.18921	G	926.50541	463.75634	11
11	70.06513	992.42390	496.71559	P	869.48394	435.24561	10
12	101.07094	1120.48248	560.74488	Q	772.43118	386.71923	9
13	44.04948	1191.51959	596.26343	A	644.37260	322.68994	8
14	44.04948	1262.55670	631.78199	A	573.33549	287.17138	7
15	44.04948	1333.59382	667.30055	A	502.29837	251.65282	6
16	44.04948	1404.63093	702.81910	A	431.26126	216.13427	5
17	44.04948	1475.66805	738.33766	A	360.22415	180.61571	4
18	44.04948	1546.70516	773.85622	A	289.18703	145.09715	3
19	44.04948	1617.74227	809.37478	A	218.14992	109.57860	2
20	101.10732			K	147.11280	74.06004	1

AGYPTGTGVGPQAAAAAAK, Y3-dichlorination (67.92206 Da)

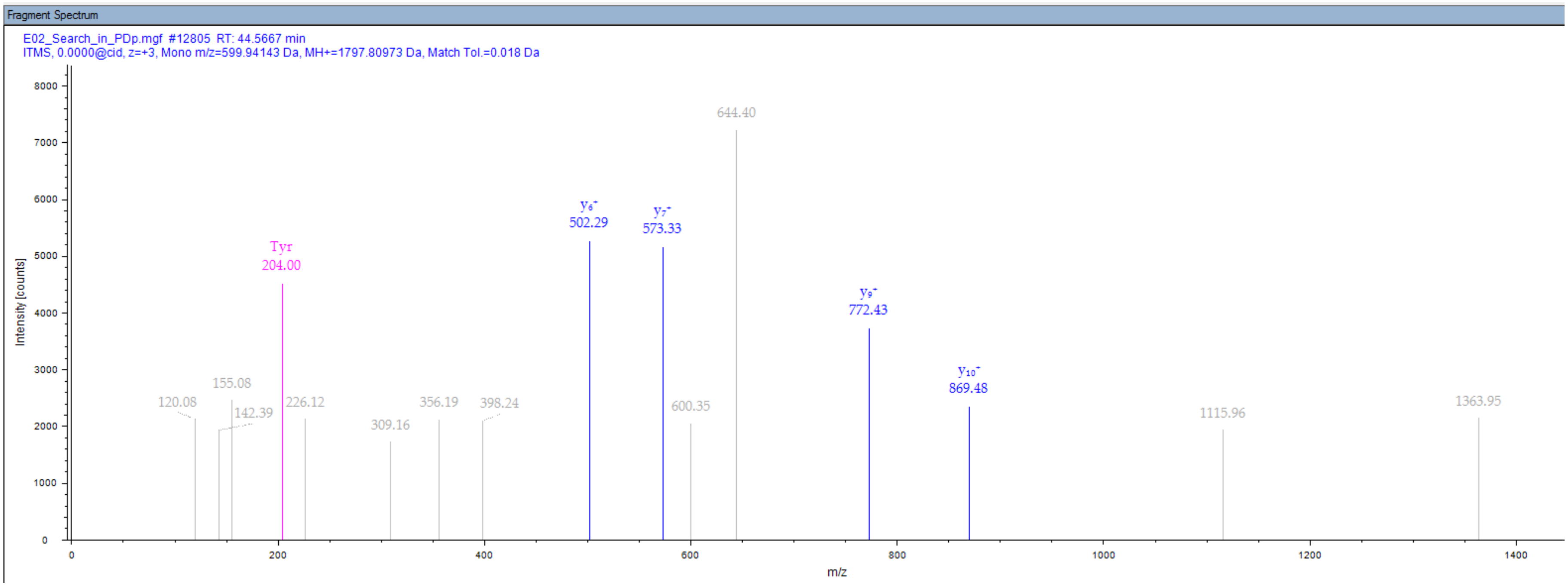

#1	Immonium	b+	b2+	b3+	Seq.	y+	y2+	y3+	#2
1	44.04948	72.04439	36.52583	24.68631	A				20
2	30.03383	129.06585	65.03657	43.69347	G	1726.77171	863.88950	576.26209	19
3	203.99775	360.05124	180.52926	120.68860	Y-dichlorin...	1669.75025	835.37876	557.25493	18
4	70.06513	457.10400	229.05564	153.03952	P	1438.76487	719.88607	480.25981	17
5	74.06004	558.15168	279.57948	186.72208	T	1341.71210	671.35969	447.90889	16
6	30.03383	615.17314	308.09021	205.72923	G	1240.66443	620.83585	414.22633	15
7	74.06004	716.22082	358.61405	239.41179	T	1183.64296	592.32512	395.21917	14
8	30.03383	773.24229	387.12478	258.41895	G	1082.59528	541.80128	361.53661	13
9	72.08078	872.31070	436.65899	291.44175	V	1025.57382	513.29055	342.52946	12
10	30.03383	929.33216	465.16972	310.44891	G	926.50541	463.75634	309.50665	11
11	70.06513	1026.38493	513.69610	342.79983	P	869.48394	435.24561	290.49950	10
12	101.07094	1154.44350	577.72539	385.48602	Q	772.43118	386.71923	258.14858	9
13	44.04948	1225.48062	613.24395	409.16506	A	644.37260	322.68994	215.46238	8
14	44.04948	1296.51773	648.76250	432.84410	A	573.33549	287.17138	191.78335	7
15	44.04948	1367.55485	684.28106	456.52313	A	502.29837	251.65282	168.10431	6
16	44.04948	1438.59196	719.79962	480.20217	A	431.26126	216.13427	144.42527	5
17	44.04948	1509.62907	755.31817	503.88121	A	360.22415	180.61571	120.74623	4
18	44.04948	1580.66619	790.83673	527.56025	A	289.18703	145.09715	97.06720	3
19	44.04948	1651.70330	826.35529	551.23928	A	218.14992	109.57860	73.38816	2
20	101.10732				K	147.11280	74.06004	49.70912	1

YGAAVPGVLGGLGALGGVGIPGGVVGA GPAAAAAAK

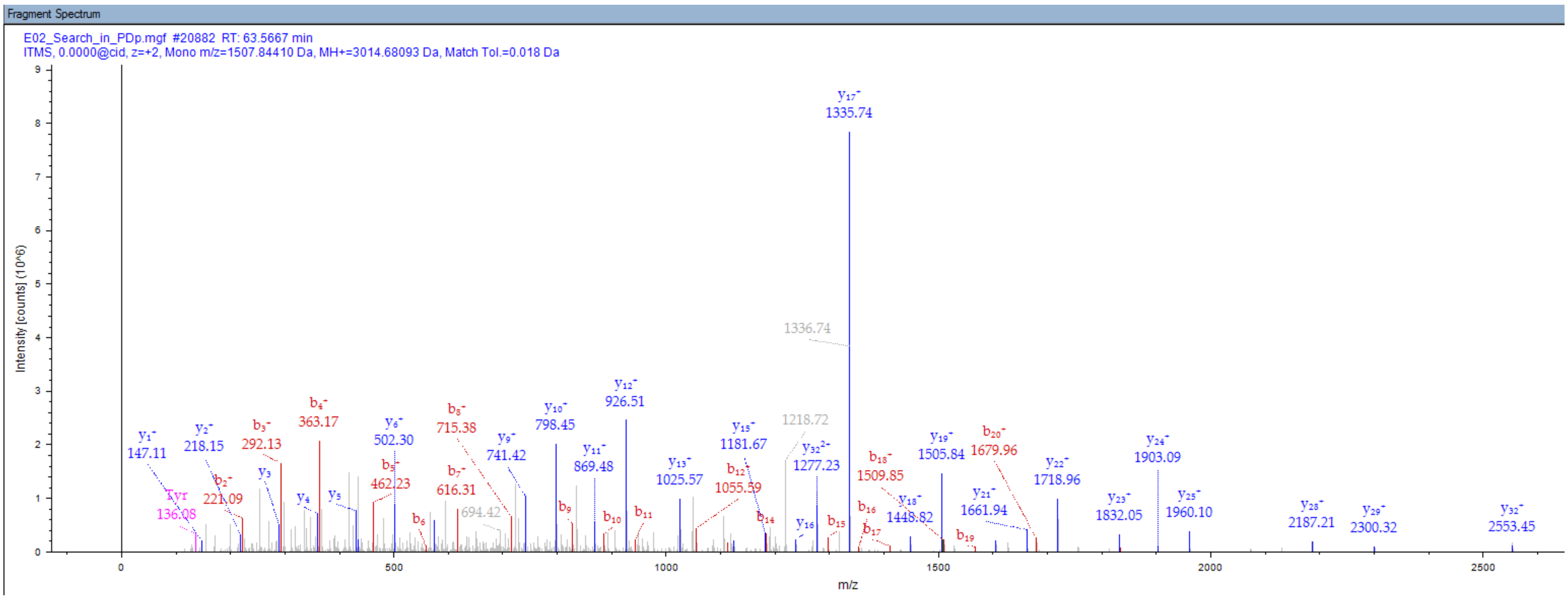

#1	Immonium	b⁺	b²⁺	Seq.	y⁺	y²⁺	#2
1	136.07569	164.07061	82.53894	Y			37
2	30.03383	221.09207	111.04967	G	2851.61524	1426.31126	36
3	44.04948	292.12918	146.56823	A	2794.59377	1397.80052	35
4	44.04948	363.16630	182.08679	A	2723.55666	1362.28197	34
5	72.08078	462.23471	231.62099	V	2652.51955	1326.76341	33
6	70.06513	559.28747	280.14738	P	2553.45113	1277.22920	32
7	30.03383	616.30894	308.65811	G	2456.39837	1228.70282	31
8	72.08078	715.37735	358.19231	V	2399.37690	1200.19209	30
9	86.09643	828.46142	414.73435	L	2300.30849	1150.65788	29
10	30.03383	885.48288	443.24508	G	2187.22443	1094.11585	28
11	30.03383	942.50434	471.75581	G	2130.20296	1065.60512	27
12	86.09643	1055.58841	528.29784	L	2073.18150	1037.09439	26
13	30.03383	1112.60987	556.80857	G	1960.09743	980.55236	25
14	44.04948	1183.64698	592.32713	A	1903.07597	952.04162	24
15	86.09643	1296.73105	648.86916	L	1832.03886	916.52307	23
16	30.03383	1353.75251	677.37989	G	1718.95479	859.98103	22
17	30.03383	1410.77398	705.89063	G	1661.93333	831.47030	21
18	72.08078	1509.84239	755.42483	V	1604.91187	802.95957	20
19	30.03383	1566.86385	783.93556	G	1505.84345	753.42536	19
20	86.09643	1679.94792	840.47760	I	1448.82199	724.91463	18
21	70.06513	1777.00068	889.00398	P	1335.73792	668.37260	17
22	30.03383	1834.02214	917.51471	G	1238.68516	619.84622	16
23	30.03383	1891.04361	946.02544	G	1181.66370	591.33549	15
24	72.08078	1990.11202	995.55965	V	1124.64223	562.82475	14
25	72.08078	2089.18044	1045.09386	V	1025.57382	513.29055	13
26	30.03383	2146.20190	1073.60459	G	926.50541	463.75634	12
27	44.04948	2217.23901	1109.12315	A	869.48394	435.24561	11
28	30.03383	2274.26048	1137.63388	G	798.44683	399.72705	10
29	70.06513	2371.31324	1186.16026	P	741.42536	371.21632	9
30	44.04948	2442.35035	1221.67882	A	644.37260	322.68994	8
31	44.04948	2513.38747	1257.19737	A	573.33549	287.17138	7
32	44.04948	2584.42458	1292.71593	A	502.29837	251.65282	6
33	44.04948	2655.46170	1328.23449	A	431.26126	216.13427	5
34	44.04948	2726.49881	1363.75304	A	360.22415	180.61571	4
35	44.04948	2797.53592	1399.27160	A	289.18703	145.09715	3
36	44.04948	2868.57304	1434.79016	A	218.14992	109.57860	2
37	101.10732			K	147.11280	74.06004	1

YGAAVPGVLGGLGALGGVGIPGGVVGA GPAAAAAAAK,
Y1-Chlorination (33.96103 Da)

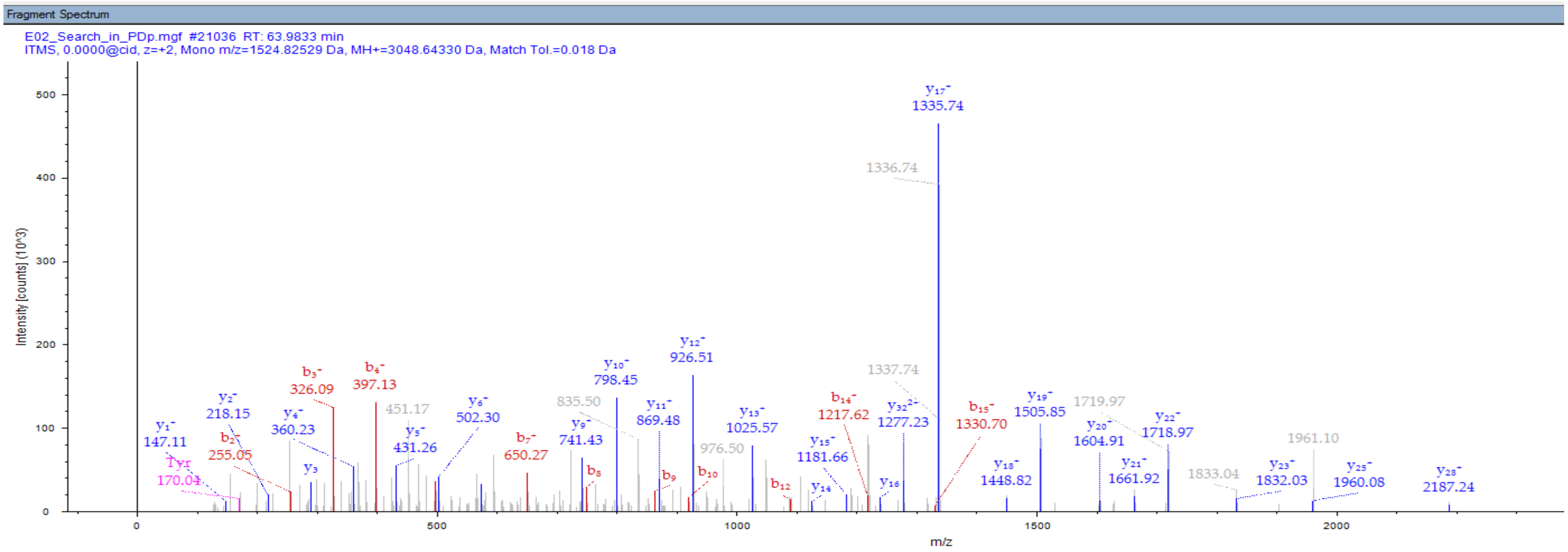

#1	Immonium	b⁺	b²⁺	Seq.	y⁺	y²⁺	#2
1	170.03672	198.03163	99.51945	Y-Chlorinat...			37
2	30.03383	255.05310	128.03019	G	2851.61524	1426.31126	36
3	44.04948	326.09021	163.54874	A	2794.59377	1397.80052	35
4	44.04948	397.12732	199.06730	A	2723.55666	1362.28197	34
5	72.08078	496.19574	248.60151	V	2652.51955	1326.76341	33
6	70.06513	593.24850	297.12789	P	2553.45113	1277.22920	32
7	30.03383	650.26997	325.63862	G	2456.39837	1228.70282	31
8	72.08078	749.33838	375.17283	V	2399.37690	1200.19209	30
9	86.09643	862.42244	431.71486	L	2300.30849	1150.65788	29
10	30.03383	919.44391	460.22559	G	2187.22443	1094.11585	28
11	30.03383	976.46537	488.73632	G	2130.20296	1065.60512	27
12	86.09643	1089.54943	545.27836	L	2073.18150	1037.09439	26
13	30.03383	1146.57090	573.78909	G	1960.09743	980.55236	25
14	44.04948	1217.60801	609.30764	A	1903.07597	952.04162	24
15	86.09643	1330.69208	665.84968	L	1832.03886	916.52307	23
16	30.03383	1387.71354	694.36041	G	1718.95479	859.98103	22
17	30.03383	1444.73500	722.87114	G	1661.93333	831.47030	21
18	72.08078	1543.80342	772.40535	V	1604.91187	802.95957	20
19	30.03383	1600.82488	800.91608	G	1505.84345	753.42536	19
20	86.09643	1713.90895	857.45811	I	1448.82199	724.91463	18
21	70.06513	1810.96171	905.98449	P	1335.73792	668.37260	17
22	30.03383	1867.98317	934.49522	G	1238.68516	619.84622	16
23	30.03383	1925.00464	963.00596	G	1181.66370	591.33549	15
24	72.08078	2024.07305	1012.54016	V	1124.64223	562.82475	14
25	72.08078	2123.14146	1062.07437	V	1025.57382	513.29055	13
26	30.03383	2180.16293	1090.58510	G	926.50541	463.75634	12
27	44.04948	2251.20004	1126.10366	A	869.48394	435.24561	11
28	30.03383	2308.22151	1154.61439	G	798.44683	399.72705	10
29	70.06513	2405.27427	1203.14077	P	741.42536	371.21632	9
30	44.04948	2476.31138	1238.65933	A	644.37260	322.68994	8
31	44.04948	2547.34850	1274.17789	A	573.33549	287.17138	7
32	44.04948	2618.38561	1309.69644	A	502.29837	251.65282	6
33	44.04948	2689.42272	1345.21500	A	431.26126	216.13427	5
34	44.04948	2760.45984	1380.73356	A	360.22415	180.61571	4
35	44.04948	2831.49695	1416.25211	A	289.18703	145.09715	3
36	44.04948	2902.53407	1451.77067	A	218.14992	109.57860	2
37	101.10732			K	147.11280	74.06004	1

YGAAVPGVLGGLGALGGVGIPGGVVGA GPAAAAAAK, Y1-dichlorination (67.92206 Da)

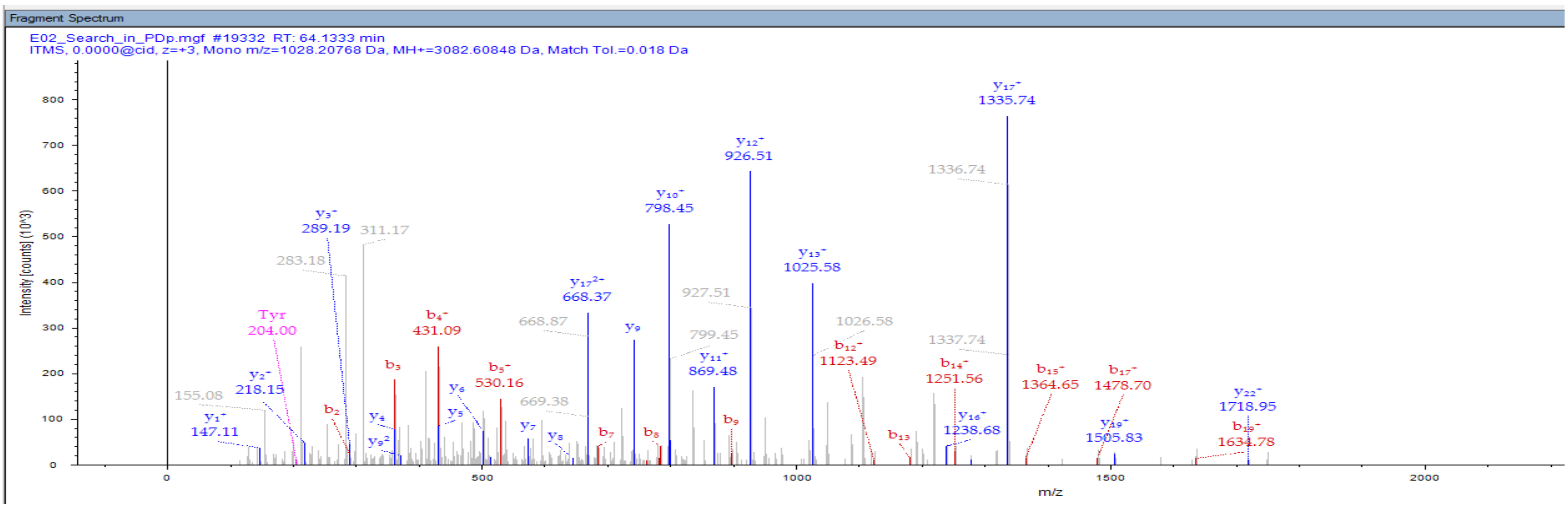

#1	Immonium	b⁺	b²⁺	b³⁺	Seq.	y⁺	y²⁺	y³⁺	#2
1	203.99775	231.99266	116.49997	78.00240	Y-dichlorin...				37
2	30.03383	289.01412	145.01070	97.00956	G	2851.61524	1426.31126	951.20993	36
3	44.04948	360.05124	180.52926	120.68860	A	2794.59377	1397.80052	932.20278	35
4	44.04948	431.08835	216.04781	144.36763	A	2723.55666	1362.28197	908.52374	34
5	72.08078	530.15677	265.58202	177.39044	V	2652.51955	1326.76341	884.84470	33
6	70.06513	627.20953	314.10840	209.74136	P	2553.45113	1277.22920	851.82189	32
7	30.03383	684.23099	342.61913	228.74852	G	2456.39837	1228.70282	819.47097	31
8	72.08078	783.29941	392.15334	261.77132	V	2399.37690	1200.19209	800.46382	30
9	86.09643	896.38347	448.69537	299.46601	L	2300.30849	1150.65788	767.44101	29
10	30.03383	953.40493	477.20611	318.47316	G	2187.22443	1094.11585	729.74633	28
11	30.03383	1010.42640	505.71684	337.48032	G	2130.20296	1065.60512	710.73917	27
12	86.09643	1123.51046	562.25887	375.17501	L	2073.18150	1037.09439	691.73202	26
13	30.03383	1180.53193	590.76960	394.18216	G	1960.09743	980.55236	654.03733	25
14	44.04948	1251.56904	626.28816	417.86120	A	1903.07597	952.04162	635.03017	24
15	86.09643	1364.65310	682.83019	455.55589	L	1832.03886	916.52307	611.35114	23
16	30.03383	1421.67457	711.34092	474.56304	G	1718.95479	859.98103	573.65645	22
17	30.03383	1478.69603	739.85165	493.57019	G	1661.93333	831.47030	554.64929	21
18	72.08078	1577.76444	789.38586	526.59300	V	1604.91187	802.95957	535.64214	20
19	30.03383	1634.78591	817.89659	545.60015	G	1505.84345	753.42536	502.61934	19
20	86.09643	1747.86997	874.43862	583.29484	I	1448.82199	724.91463	483.61218	18
21	70.06513	1844.92274	922.96501	615.64576	P	1335.73792	668.37260	445.91749	17
22	30.03383	1901.94420	951.47574	634.65292	G	1238.68516	619.84622	413.56657	16
23	30.03383	1958.96566	979.98647	653.66007	G	1181.66370	591.33549	394.55942	15
24	72.08078	2058.03408	1029.52068	686.68288	V	1124.64223	562.82475	375.55226	14
25	72.08078	2157.10249	1079.05488	719.70568	V	1025.57382	513.29055	342.52946	13
26	30.03383	2214.12395	1107.56562	738.71284	G	926.50541	463.75634	309.50665	12
27	44.04948	2285.16107	1143.08417	762.39187	A	869.48394	435.24561	290.49950	11
28	30.03383	2342.18253	1171.59490	781.39903	G	798.44683	399.72705	266.82046	10
29	70.06513	2439.23530	1220.12129	813.74995	P	741.42536	371.21632	247.81331	9
30	44.04948	2510.27241	1255.63984	837.42899	A	644.37260	322.68994	215.46238	8
31	44.04948	2581.30952	1291.15840	861.10803	A	573.33549	287.17138	191.78335	7
32	44.04948	2652.34664	1326.67696	884.78706	A	502.29837	251.65282	168.10431	6
33	44.04948	2723.38375	1362.19551	908.46610	A	431.26126	216.13427	144.42527	5
34	44.04948	2794.42086	1397.71407	932.14514	A	360.22415	180.61571	120.74623	4
35	44.04948	2865.45798	1433.23263	955.82418	A	289.18703	145.09715	97.06720	3
36	44.04948	2936.49509	1468.75118	979.50322	A	218.14992	109.57860	73.38816	2
37	101.10732				K	147.11280	74.06004	49.70912	1

YGVAARPGFGLSPIFPGGACLGK

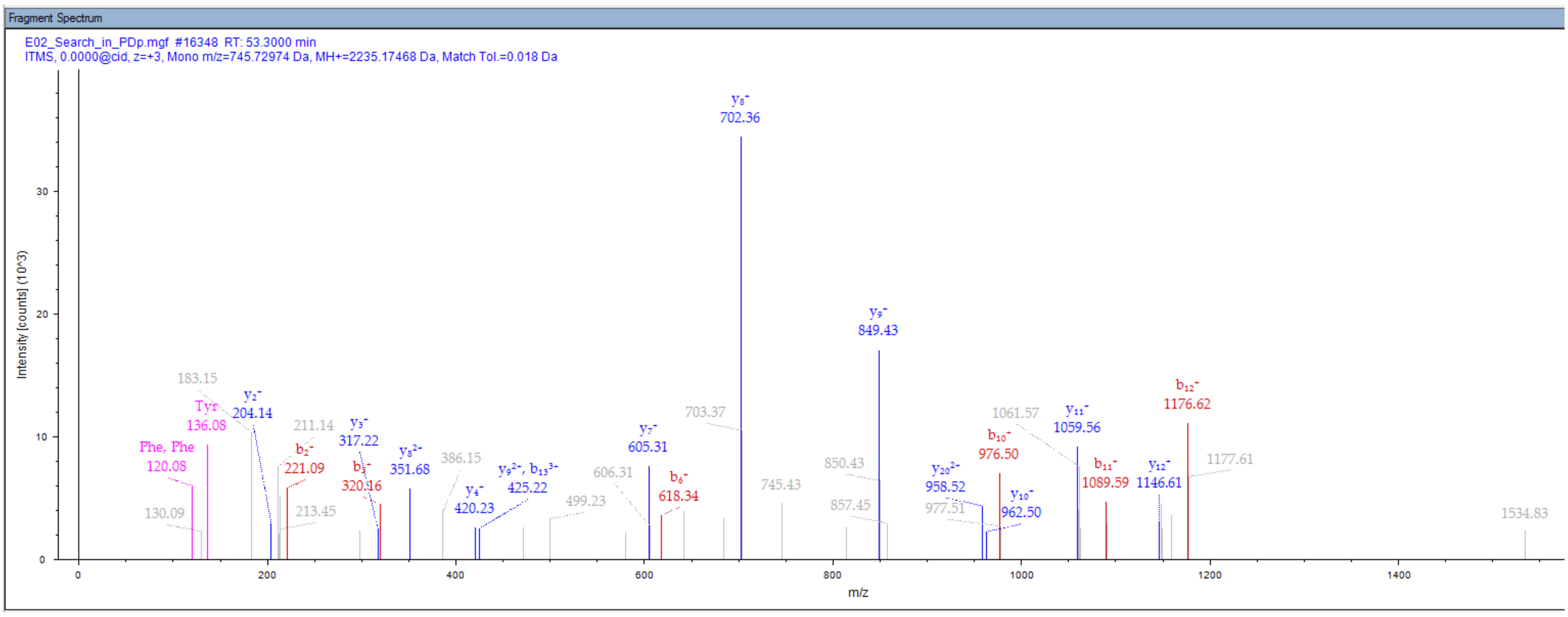

#1	Immonium	b⁺	b²⁺	b³⁺	Seq.	y⁺	y²⁺	y³⁺	#2
1	136.07569	164.07061	82.53894	55.36172	Y				23
2	30.03383	221.09207	111.04967	74.36887	G	2072.11098	1036.55913	691.37518	22
3	72.08078	320.16048	160.58388	107.39168	V	2015.08951	1008.04839	672.36802	21
4	44.04948	391.19760	196.10244	131.07072	A	1916.02110	958.51419	639.34522	20
5	44.04948	462.23471	231.62099	154.74975	A	1844.98398	922.99563	615.66618	19
6	129.11347	618.33582	309.67155	206.78346	R	1773.94687	887.47707	591.98714	18
7	70.06513	715.38858	358.19793	239.13438	P	1617.84576	809.42652	539.95344	17
8	30.03383	772.41005	386.70866	258.14153	G	1520.79300	760.90014	507.60252	16
9	120.08078	919.47846	460.24287	307.16434	F	1463.77153	732.38940	488.59536	15
10	30.03383	976.49993	488.75360	326.17149	G	1316.70312	658.85520	439.57256	14
11	86.09643	1089.58399	545.29563	363.86618	L	1259.68166	630.34447	420.56540	13
12	60.04439	1176.61602	588.81165	392.87686	S	1146.59759	573.80243	382.87071	12
13	70.06513	1273.66878	637.33803	425.22778	P	1059.56556	530.28642	353.86004	11
14	86.09643	1386.75285	693.88006	462.92247	I	962.51280	481.76004	321.50912	10
15	120.08078	1533.82126	767.41427	511.94527	F	849.42874	425.21801	283.81443	9
16	70.06513	1630.87402	815.94065	544.29619	P	702.36032	351.68380	234.79162	8
17	30.03383	1687.89549	844.45138	563.30335	G	605.30756	303.15742	202.44070	7
18	30.03383	1744.91695	872.96211	582.31050	G	548.28609	274.64669	183.43355	6
19	44.04948	1815.95407	908.48067	605.98954	A	491.26463	246.13595	164.42639	5
20	76.02155	1918.96325	959.98526	640.32593	C	420.22752	210.61740	140.74736	4
21	86.09643	2032.04731	1016.52730	678.02062	L	317.21833	159.11280	106.41096	3
22	30.03383	2089.06878	1045.03803	697.02778	G	204.13427	102.57077	68.71627	2
23	101.10732				K	147.11280	74.06004	49.70912	1

YGVAARPGFGLSPIFPGGACLGK, C20-Trioxidation (47.98474 Da)

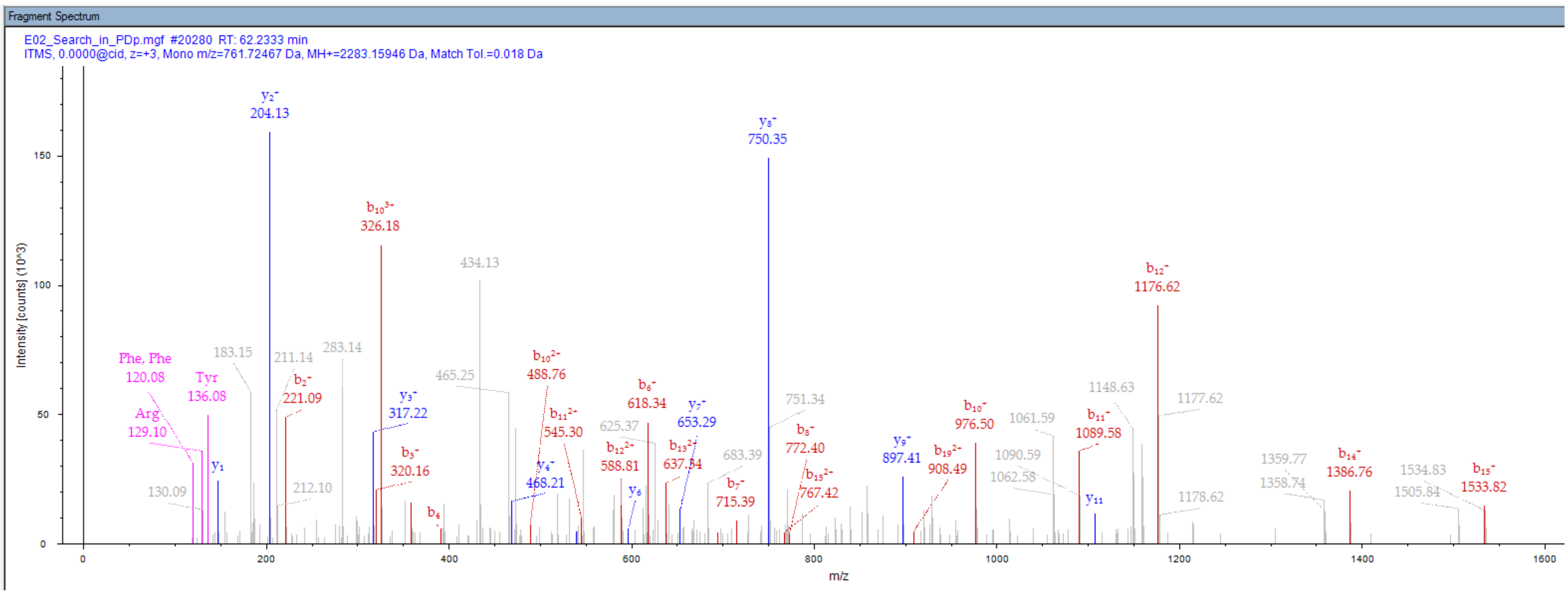

#1	Immonium	b⁺	b²⁺	b³⁺	Seq.	y⁺	y²⁺	y³⁺	#2
1	136.07569	164.07061	82.53894	55.36172	Y				23
2	30.03383	221.09207	111.04967	74.36887	G	2120.09572	1060.55150	707.37009	22
3	72.08078	320.16048	160.58388	107.39168	V	2063.07426	1032.04077	688.36294	21
4	44.04948	391.19760	196.10244	131.07072	A	1964.00584	982.50656	655.34013	20
5	44.04948	462.23471	231.62099	154.74975	A	1892.96873	946.98800	631.66109	19
6	129.11347	618.33582	309.67155	206.78346	R	1821.93162	911.46945	607.98206	18
7	70.06513	715.38858	358.19793	239.13438	P	1665.83050	833.41889	555.94835	17
8	30.03383	772.41005	386.70866	258.14153	G	1568.77774	784.89251	523.59743	16
9	120.08078	919.47846	460.24287	307.16434	F	1511.75628	756.38178	504.59028	15
10	30.03383	976.49993	488.75360	326.17149	G	1364.68786	682.84757	455.56747	14
11	86.09643	1089.58399	545.29563	363.86618	L	1307.66640	654.33684	436.56032	13
12	60.04439	1176.61602	588.81165	392.87686	S	1194.58234	597.79481	398.86563	12
13	70.06513	1273.66878	637.33803	425.22778	P	1107.55031	554.27879	369.85495	11
14	86.09643	1386.75285	693.88006	462.92247	I	1010.49754	505.75241	337.50403	10
15	120.08078	1533.82126	767.41427	511.94527	F	897.41348	449.21038	299.80934	9
16	70.06513	1630.87402	815.94065	544.29619	P	750.34507	375.67617	250.78654	8
17	30.03383	1687.89549	844.45138	563.30335	G	653.29230	327.14979	218.43562	7
18	30.03383	1744.91695	872.96211	582.31050	G	596.27084	298.63906	199.42846	6
19	44.04948	1815.95407	908.48067	605.98954	A	539.24937	270.12833	180.42131	5
20	124.00629	1966.94799	983.97764	656.32085	C-Trioxidat..	468.21226	234.60977	156.74227	4
21	86.09643	2080.03206	1040.51967	694.01554	L	317.21833	159.11280	106.41096	3
22	30.03383	2137.05352	1069.03040	713.02269	G	204.13427	102.57077	68.71627	2
23	101.10732				K	147.11280	74.06004	49.70912	1
